# Microdissecting the Hypoxia Landscape in Colon Cancer Reveals Three Distinct Subtypes and Their Potential Mechanism to Facilitate the Development of Cancer

**DOI:** 10.1155/2023/9346621

**Published:** 2023-03-07

**Authors:** Pingfei Tang, Yueming Wu, Chaojun Zhu, Qingyuan Li, Side Liu

**Affiliations:** Guangdong Provincial Key Laboratory of Gastroenterology, Department of Gastroenterology, Nanfang Hospital, Southern Medical University, Guangzhou 510515, China

## Abstract

**Background:**

Hypoxia contributes to tumor progression and confers drug resistance. We attempted to microdissect the hypoxia landscape in colon cancer (CC) and explore its correlation with immunotherapy response.

**Materials and Methods:**

The hypoxia landscape in CC patients was microdissected through unsupervised clustering. The “xCell” algorithms were applied to decipher the tumor immune infiltration characteristics. A hypoxia-related index signature was developed via the LASSO (least absolute shrinkage and selection operator) Cox regression in The Cancer Genome Atlas (TCGA)-colon adenocarcinoma (COAD) cohort and validated in an independent dataset from the Gene Expression Omnibus (GEO) database. The tumor immune dysfunction and exclusion (TIDE) algorithm was utilized to evaluate the correlation between the hypoxia-related index (HRI) signature and immunotherapy response. Quantitative reverse transcription polymerase chain reaction (qRT-PCR) and western blotting were performed to verify the mRNA expression levels of five key genes. The Cell Counting Kit-8 (CCK-8) assay and flow cytometry were performed to examine the cell viability and cell apoptosis.

**Results:**

Patients were classified into hypoxia-high, hypoxia-median, and hypoxia-low clusters in TCGA-COAD and verified in the GSE 17538 dataset. Compared with the hypoxia-low cluster, the hypoxia-high cluster consistently presented an unfavorable prognosis, higher immune scores, and stromal scores and elevated infiltration levels of several critical immune and stromal cells. Otherwise, we also found 600 hypoxia-related differentially expressed genes (HRDEGs) between the hypoxia-high cluster and the hypoxia-low cluster. Based on the 600 HRDEGs, we constructed the HRI signature which consists of 11 genes and shows a good prognostic value in both TCGA-COAD and GSE 17538 (AUC of 6-year survival prediction >0.75). Patients with low HRI scores were consistently predicted to be more responsive to immunotherapy. Of the 11 HRI signature genes, RGS16, SNAI1, CDR2L, FRMD5, and FSTL3 were differently expressed between tumors and adjacent tissues. Low expression of SNAI1, CDR2L, FRMD5, and FSTL3 could induce cell viability and promote tumor cell apoptosis.

**Conclusion:**

In our study, we discovered three hypoxia clusters which correlate with the clinical outcome and the tumor immune microenvironment in CC. Based on the hypoxia cluster and HRDEGs, we constructed a reliable HRI signature that could accurately predict the prognosis and immunotherapeutic responsiveness in CC patients and discovered four key genes that could affect tumor cell viability and apoptosis.

## 1. Introduction

Colon cancer (CC) is the fifth most frequent malignant disease with 1,148,515 new cases diagnosed in 2020 and accounting for 576,858 cancer-associated deaths around the world [1]. The 5-year survival probability for colorectal cancer ranges from 90% in early-stage patients to 14% in distant-stage patients [2]. The American Joint Committee on Cancer (AJCC) staging is a critical assessment system for the treatment management of CC [3], and patients with stage III or high-risk stage II may need to undergo a combination treatment of curative resection and adjuvant therapy [4]. However, most of the distant-stage patients miss the radical surgical opportunity and die due to metastasis or recurrence. Owing to the tumor heterogeneity and diverse molecular pattern, patients with the same AJCC stage exhibit tremendous survival differences. Thus, it is imperative to conduct in-depth microdissection and develop new prognostic biomarkers for patients with CC.

Hypoxia is a specific hallmark of solid tumors, owing to the unrestricted growth and abnormal vascularization during the tumor progression [5]. Hypoxia promotes the tumor metastatic cascade, including invasion, migration, and distant metastasis [6]. The hypoxia-inducible factor (HIF)-1*α* pathway contributes greatly to the modulation of hypoxia-related downstream gene expression and pathway activity in cancer cells under hypoxic conditions [7]. Hypoxia also promotes the epithelial-to-mesenchymal transition (EMT) process and facilitates the invasion of CC cells by activation of HIF1A, whereas treatment with HIF1A-specific small interfering RNAs (siRNAs) suppresses these processes [8]. Our previous study [9] has constructed a hypoxia-related long noncoding RNAs signature that is tightly associated with the prognosis and drug sensitivity in patients with hepatocellular carcinoma. The HIF-1*α* signaling pathway also confers drug resistance under hypoxic stress in colorectal carcinoma [10, 11]. Hence, we speculate that the hypoxic exposure level in tumor tissues probably has a critical impact on the prognosis and treatment effectiveness of CC.

Over the past decade, immunotherapeutic treatment based on immune checkpoint inhibitors (ICIs) has resulted in revolutionary long-term benefits in the therapy of several cancer types [12]. ICIs such as anti-PD-1 (programmed cell death 1) and anti-PD-L1 (programmed cell death 1 ligand 1) have achieved a durable response in a subset of microsatellite instability-high (MSI-H) patients [12], whereas the MSI-L/MSS (MSI-low/microsatellite stability) patients who constitute the majority of CC patients have not obtained satisfactory benefits from ICI treatment. Interestingly, hypoxia has been reported to affect tumor plasticity, heterogeneity, and the immune resistance phenotype [13]. Hypoxia not only recruits myeloid-derived suppressive cells (MDSCs), cancer-associated fibroblasts (CAFs), and regulatory T cells (Tregs) to induce tumor immunosuppression [14] but also augments the expression level of immune checkpoints such as PD-L1 to promote tumor immune evasion [15]. Hence, targeting the hypoxic microenvironment may improve the efficacy of cancer immunotherapy [16]. Nevertheless, there is still a deficiency in comprehensive delineation of the interplay among hypoxia, tumor immune infiltrating patterns, and immunotherapy response in patients with CC.

In the current study, we discovered the hypoxia cluster in CC patients using unsupervised clustering based on two publicly available datasets (TCGA-COAD and GSE17538) and investigated the intrinsic correlation between hypoxia and the tumor immune microenvironment by the xCell algorithm and TIDE. Additionally, we developed a reliable hypoxia-related index (HRI) prognostic signature that exhibited good performance in predicting clinical prognosis and immunotherapy response in two independent datasets by the LASSO cox regression model. Finally, *in vitro* experiments were supplied to explore the results at the cell level. Our findings may deepen the understanding of the hypoxia role in the tumor microenvironment and provide beneficial information for immunotherapy in CC.

## 2. Materials and Methods

### 2.1. Data Preprocessing

The fragments per kilobase per million mapped reads (FPKM) profiles of the level-3 sequencing transcriptomic data in TCGA-COAD cohort were obtained from TCGA database (https://portal.gdc.cancer.gov/). We subsequently converted the FPKM values into the log2-transformed TPM (transcripts per million) values for further analysis. The corresponding detailed clinical parameters were publicly acquired from the cBioPortal database [17] (https://cbioportal.org).

Another publicly available, independent microarray dataset, GSE17538, was downloaded from the Gene Expression Omnibus database (https://www.ncbi.nlm.nih.gov/geo/). The TCGA-COAD cohort consisted of 402 primary CC samples and 39 adjacent normal tissues. Only 348 patients with complete clinical data and overall survival (OS) time of ≥1 month and 39 normal samples were used as the discovery cohort. The GSE17538 dataset was composed of two subsets, GSE17536 (177 CC patients) and GSE17537 (55 CC patients), and the nonbiological batch was corrected using the “ComBat” function via the R “sva” package. In total, 210 patients with CC in GSE17538 with complete clinical and histopathological grade information were enrolled as the independent validation cohort. Detailed information on all enrolled patients in the previous two datasets is listed in Supplemental Table 1 (Table S1).

### 2.2. Microdissecting the Hypoxia-Specific Cluster of CC

The “HALLMARK_HYPOXIA” gene set (“h.all.v7.2.symbols.gmt”) includes 200 hypoxia-specific genes (Table S2), which have been demonstrated to typically represent the biological process under hypoxia conditions and was gathered from the molecular signatures database (MsigDB) [18]. TCGA-COAD cohort (348 patients) and GSE17538 dataset (210 patients) were assigned into different groups by the unsupervised clustering method according to the expression of the previous 200 hypoxia-specific genes, respectively, via the “km” method in the R “ConsensusClusterPlus” package. Survival analysis for hypoxia-specific clusters was performed by the R “survival” package, and the survival difference among these clusters was determined by the log-rank test.

### 2.3. Gene Set Variation Analysis (GSVA)

Overall, 50 hallmark gene sets (h.all.v7.2.symbols.gmt) were downloaded from the MSigDB database [18]. In addition, 13 typical metabolic pathways (Table S3) associated with “GLYCOLYSIS,” “OXIDATIVE_PHOSPHORYLATION,” and “CITRATE_CYCLE_TCA_CYCLE” were curated from the MSigDB database. The activity differences of these hallmark pathways and metabolic pathways among different hypoxia-specific clusters were explored by GSVA [19], which can calculate a specific pathway score for each sample using an unsupervised nonparametric algorithm.

### 2.4. Identifying Hypoxia-Related Differentially Expressed Genes (HRDEGs)

Analysis of differentially expressed genes (DEGs) between the 348 COAD cancer samples and 39 adjacent normal samples in TCGA-COAD cohort was carried out by the “limma” package, according to the standard of the absolute value of log2 (fold change) greater than 1 and an adjusted *p* value less than 0.05. With the same method and criteria, DEGs between the hypoxia-high and hypoxia-low clusters were further examined. HRDEGs were defined as the intersection of the previous two gene lists.

### 2.5. Development of the HRI Signature

A univariable Cox regression model was applied to screen the prognostic HRDEGs in TCGA-COAD cohort. The LASSO penalty Cox regression model, which can avoid overfitting and select the most contributive variables through tuning the penalty parameter, was employed to develop the optimal HRI signature using the “glmnet” package [20]. The final HRI score formula is defined as follows: risk score=∑_*k*=1_^*n*^*expk∗coefk*, where *k* is the sequence number of the prognostic gene in the HRI signature, exp*k* represents the corresponding gene expression of each patient, and coef*k* represents the corresponding LASSO coefficient.

### 2.6. Evaluation and Validation of the Prognostic Capability of the HRI Signature

HRI scores of CC patients in TCGA-COAD cohort (discovery dataset) and GSE17538 (validation dataset) were calculated using the previous formula. Patients in each dataset were assigned to the HRI high- or low-risk group according to their respective median HRI scores. Survival analysis for each dataset was carried out by the “survival” package, and the survival differences were determined by the log-rank test. Time-dependent receiver operating characteristic (ROC) curves were drawn to evaluate the performance for prognosis prediction using the “timeROC” package. Multivariable Cox regression was conducted to determine whether the HRI signature was independent of other clinical parameters (age, sex, AJCC stage, and histopathological grade) in prognostic prediction.

### 2.7. Single Sample Gene Set Enrichment Analysis (GSEA)

The gene list of critical immune function pathways (Table S4) was collected from the previous studies [21]. Single-sample GSEA (ssGSEA) [22], a particular kind of GSEA that can calculate the relative score for a predefined gene list at a single sample level, was utilized to calculate the relative scores of the previous immune function pathways using the “GSVA” package in R.

### 2.8. Analyzing the Immune Landscape of Hypoxia-Specific Clusters

The “xCell” algorithm, which can effectively infer immune and stromal cell abundance from the mixture transcriptomic profiles [23], was applied to comprehensively delineate the tumor immune microenvironment (TIME).

### 2.9. Evaluating the HRI Predictive Ability of Immunotherapy Response

The tumor immune dysfunction and exclusion (TIDE) algorithm, which can calculate the TIDE scores representing the dysregulation of tumor immune escape for tumor samples and function as a representative biomarker to predict responsiveness to immune checkpoint blockade [24], was employed to examine the HRI predictive capability of immunotherapy response in CC patients.

### 2.10. Quantitative Reverse Transcription Polymerase Chain Reaction (qRT-PCR)

Thirty pairs of clinical samples (including tumors and corresponding adjacent normal samples) of patients diagnosed with CC were gathered at Nanfang Hospital of Southern Medical University. The samples were immediately preserved at −80°C postcollection after surgical resection until RNA extraction. All patients gave informed consent for sample collection and usage. The present research was supported by the Institutional Ethical Committee Board of Nanfang Hospital (NFEC-201809-K3). Total RNA from 30 pairs of clinical tissues was isolated using an RNAex Pro Reagent (Accurate Biology, China). qRT-PCR reactions were performed using the Evo M-MLV RT Premix for qPCR (Accurate Biology, China) and SYBR® Green Premix Pro Taq HS qPCR Kit (Accurate Biology, China). GAPDH was utilized as the internal standard, and each sample was analyzed in triplicate. All PCR primer sequences are presented in Table S5. Relative quantification of mRNA expression levels of RGS16, SNAI1, CDR2L, FRMD5, and FSTL3 was analyzed via the 2^−ΔΔCt^ method.

### 2.11. Cell Culture and Cell Transfection

Human colon cell line HCT116 was obtained from ATCC. Then, the cells were cultured in DMEM with 10% FBS at 37°C in 5% CO_2_.

The plasmid and scramble were purchased from Biosystems (General Biosystems, Anhui, China). siRNA and siRNA scramble were obtained from the GenePharma Corporation (Shanghai, China). According to the introduction, all siRNA and vectors were transfected using a lipofectamine 3000 transfection kit (Invitrogen, USA). qRT-PCR was performed to test the transfection efficiency.

### 2.12. Western Blot

Proteins were extracted using RIPA (CWBIO, China), subjected to SDS-PAGE gel electrophoresis, and then transferred to a nitrocellulose membrane, incubated with primary antibodies, and incubated overnight at 4°C. The secondary antibody was then incubated for 1 h at room temperature. Immobilon ECL substrate was used for signal detection and image acquisition.

### 2.13. CCK-8 Assay

The Cell Counting Kit-8 (CCK-8, ImmunoWay Biotechnology Company, Plano, TX, USA) assay was used to monitor cell proliferation. In brief, the cells transfected with siRNA or plasmid were placed on 96-well plates and cultured for 24 h, 48 h, 72 h, and 96 h. Then, the OD450 value was detected using the Thermo Scientific Varioskan Flash spectrophotometer (Thermo Scientific, Finland).

### 2.14. Flow Cytometry

Stably transfected tumor cells were placed in 6-well plates, 3 × 10^5^ cells per well. The purified tumor cells were adjusted to 1 × 10^6^/L. The apoptosis rate of tumor cells was evaluated by flow cytometry (FACScan, BD Bioscience) with an Annexin-V-FITC/PI apoptosis kit (ads5001; Absin, Shanghai, China).

### 2.15. Statistical Analysis

Numerical variable differences with normal distribution were determined using Student's *t*-test or analysis of variance for two or more groups, respectively. The Wilcoxon rank-sum test or Kruskal–Wallis test were performed to determine the numerical variable differences with nonnormal distribution for two or more groups, respectively. Categorical variable differences were examined via the chi-square test. Spearman correlation analysis was conducted to investigate the correlation between the continual variables. Univariate Cox and LASSO penalty Cox regression analyses were utilized to perform survival analyses. Survival differences were examined by the Kaplan–Meier curve and log-rank test. A two-tailed *p* value of <0.05 was set to indicate statistical significance. For multiple testing, the *p* value was corrected by the Benjamini–Hochberg method. We utilized R software (version 3.6.3) to perform all the statistical analyses.

## 3. Results

### 3.1. The Discovery of Hypoxia-Related Cluster Using Unsupervised Clustering

**Figure 1:**
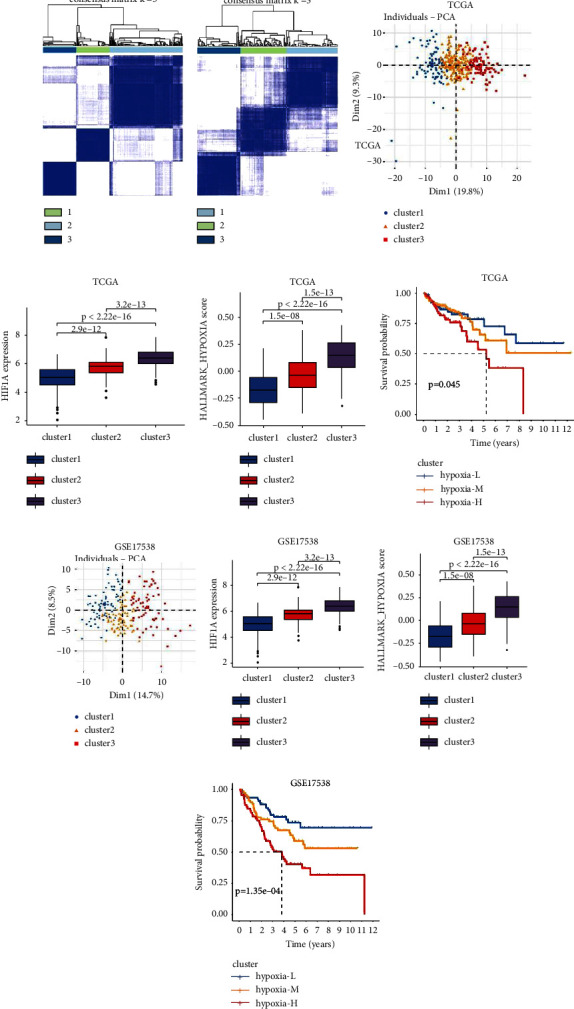
Microdissection of the hypoxia landscape in TCGA-COAD cohort and GSE17538 cohort. Consensus matrix plot of unsupervised clustering in TCGA-COAD cohort (a) and GSE17538 cohort (b), when *k* = 3 representing the optimal cluster number. (c) and (g) PCA plot of hypoxia-specific clusters. Comparison of HIF1A expression (d) and (h), HALLMARK_HYPOXIA pathway score (e) and (i), and the survival difference (f) and (j) among hypoxia-specific clusters. COAD: colon adenocarcinoma. PCA: principal component analysis. Hypoxia-L: hypoxia-low; hypoxia-M: hypoxia-median; hypoxia-H: hypoxia-high. OS: overall survival.

In total, 348 patients with complete clinical information in TCGA-COAD cohort were categorized into three different clusters by unsupervised clustering (Figure 1(a) and Figures S1A-S1B). Clusters 1, 2, and 3 consisted of 82, 182, and 84 patients, respectively. The detailed lists are shown in Table S6. The principal component analysis confirmed a clear distinction among the three clusters (Figure 1(c)). To clarify the relationship between the clusters and hypoxia, the HIF1A messenger RNA (mRNA) expression, which represents the mRNA level of the master regulator HIF-1*α* under hypoxic conditions, was compared among the three clusters. Notably, cluster 3 possessed the highest HIF1A mRNA level, while cluster 1 exhibited the lowest HIF1A mRNA level (Figure 1(d)). GSVA further showed that cluster 3 had the highest activity in the “HALLMARK_HYPOXIA” pathway, whereas cluster 1 displayed the lowest pathway activity (Figure 1(e)). These results demonstrated that the previous three clusters were strongly correlated with hypoxia exposure in CC tissues. Henceforth, we defined clusters 3, 2, and 1 as the hypoxia-high, hypoxia-median, and hypoxia-low subtypes, respectively. Survival analysis revealed a significant OS difference among the three hypoxia-specific clusters (global *p* value = 0.045, Figure 1(f)). The hypoxia-high subtype had the poorest OS outcome compared with the hypoxia-low (*p* = 0.031) and hypoxia-median (*p* = 0.046) subtypes.

To further verify the hypoxic landscape in CC, the independent microarray dataset GSE17538 was explored using the same unsupervised clustering method. Notably, 210 patients with complete clinical characteristics in GSE17538 were likewise classified into three different clusters (Figures 1(b), S1C-S1D, and 1(g)), with detailed lists shown in Table S7), namely, cluster 1 (77 patients), cluster 2 (68 patients), and cluster 3 (65 patients). Similarly, cluster 3 had the highest level of HIF1A mRNA expression and the activity of the “HALLMARK_HYPOXIA” pathway, while cluster 1 exhibited the lowest level for the previous two indices (Figures 1(h) and 1(i)). Thus, we also defined clusters 3, 2, and 1 in GSE17538 as the hypoxia-high, hypoxia-median, and hypoxia-low subtypes, respectively. In addition, there was a significant OS difference among the three clusters (global *p* value = 1.35*e* − 04, Figure 1(j)). The hypoxia-high cluster showed the poorest OS outcome compared with the hypoxia-low (*p* value 3.22*e* − 05) and hypoxia-median (*p* value 0.019) clusters. The previous results confirmed that the hypoxia exposure landscape is closely correlated with the clinical outcomes in patients with CC.

### 3.2. Distinct Molecular Patterns among the Hypoxia-Specific Clusters

**Figure 2:**
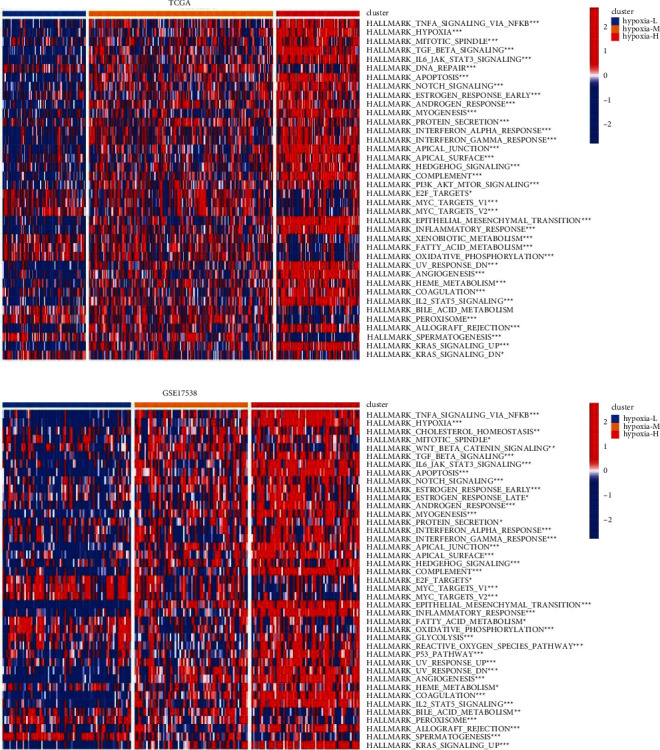
The distinct molecular pattern among the hypoxia-specific clusters. Comparison of the relative activities of the hallmark gene sets among hypoxia-specific clusters in TCGA-COAD (a) and GSE 17538 (b). COAD: colon adenocarcinoma. Hypoxia-L: hypoxia-low; hypoxia-M: hypoxia-median; hypoxia-H: hypoxia-high. ^*∗∗∗*^*p* < 0.001; ^*∗∗*^*p* < 0.01; ^*∗*^*p* < 0.05.

Owing to the close relationship between hypoxia-specific clusters and clinical outcomes, we continued to explore the underlying molecular mechanisms. GSVA results for the hallmark gene sets showed that the relative activities of several tumor aggression-associated pathways, including “EPITHELIAL_MESENCHYMAL_TRANSITION,” “ANGIOGENESIS,” “MYOGENESIS,” “APICAL_JUNCTION,” “APICAL_SURFACE,” “HYPOXIA,” and “IL6_JAK_STAT3_SIGNALING,” were elevated in the hypoxia-high group compared with those in the hypoxia-low group in both TCGA-COAD and GSE17538 datasets (Figures 2(a) and 2(b)).

### 3.3. Identification of HRDEGs

**Figure 3:**
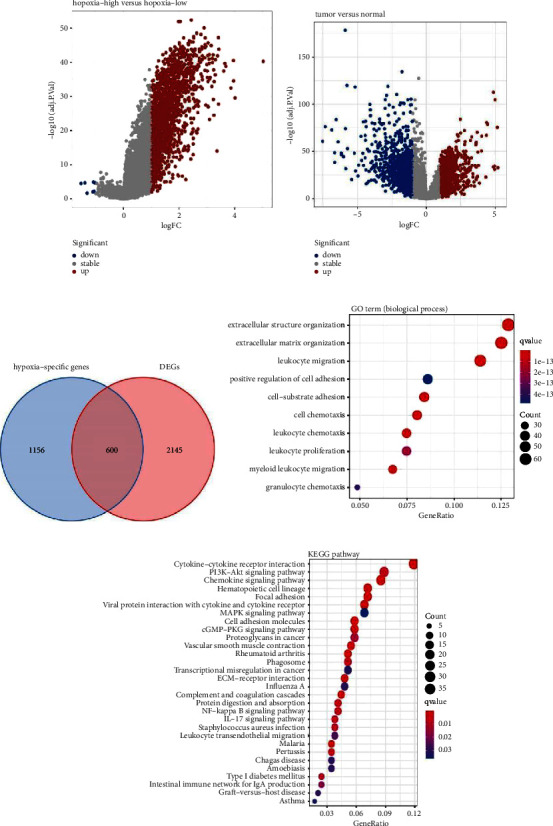
Identification of the hypoxia-related differentially expressed genes (HRDEGs) in TCGA-COAD cohort. (a) Volcano plot of hypoxia-specific genes between hypoxia-high and hypoxia-low clusters. (b) Volcano plot of differentially expressed genes (DEGs) between tumor and adjacent normal tissues. (c) Venn diagram of HRDEGs. Bubble plots for GO (d) and KEGG (e) functional annotation of the 600 HRDEGs. GO: gene ontology. KEGG: Kyoto encyclopedia of genes and genomes.

In total, 1756 DEGs (1748 upregulated and 8 downregulated genes, Figure 3(a)) between the hypoxia-high and hypoxia-low clusters (|log2FC| greater than 1 and adjusted *p* value less than 0.05) were identified. Using the same criteria, we acquired 2745 DEGs (1442 upregulated and 1303 downregulated genes, Figure 3(b)) between the tumor tissues and adjacent normal samples. Furthermore, 600 overlapping genes for the previous two gene lists (Figure 3(c), detailed lists shown in Table S8) were categorized as HRDEGs. Gene Ontology (GO) function enrichment analysis demonstrated that these HRDEGs were predominantly enriched in several biological process (BP) terms, including “extracellular matrix organization,” “positive regulation of cell adhesion,” and “cell-substrate adhesion” (Figure 3(d)). KEGG pathway analysis further showed a strong linkage between the HRDEGs and the following pathways: “cytokine-cytokine receptor interaction,” “PI3K−Akt signaling pathway,” and “focal adhesion” (Figure 3(e)). These enriched terms were closely associated with extracellular signal communication and cancer cell invasion, indicating that our defined HRDEGs probably participated in the tumor progression.

### 3.4. Development of HRI Signature

**Figure 4:**
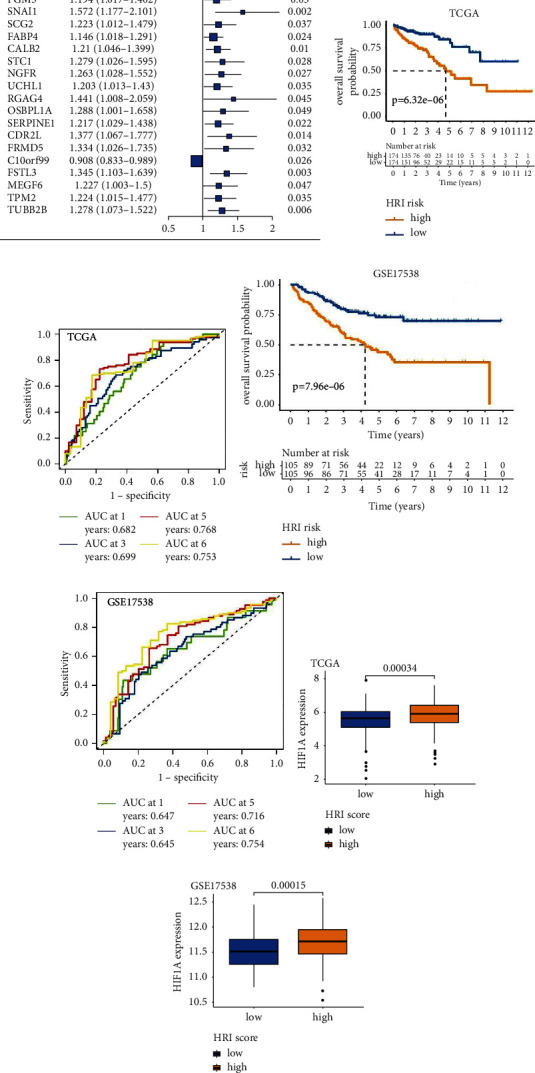
Development of the HRI signature. (a) Forest plot of twenty-two prognostic genes obtained by univariate Cox regression. Kaplan–Meier curves and the log-rank test *p* value for TCGA-COAD (b) and GSE17538 (d) datasets. The AUCs of the time-dependent ROC curves for TCGA-COAD (c) and GSE17538 (e) datasets. Comparison of HIF1A mRNA expression between HRI high-risk and low-risk groups in TCGA-COAD (f) and GSE17538 (g). AUC: area under the curve. ROC: receiver operating characteristic.

TCGA-COAD cohort with 348 patients was utilized as the discovery cohort to construct an HRI signature. The univariate Cox regression model yielded 22 prognostic genes out of the aforementioned 600 HRDEGs (Figure 4(a)). LASSO penalty Cox regression selected the optimal HRI signature according to the “lambda. min” standard, which represents the lambda (tuning parameter) with minimal cross-validation error. Ultimately, 11 selected optimal prognostic HRDEGs were incorporated to develop the HRI score signature (Figures S2A-S2B, detailed gene list shown in Table S9). The final HRI calculation formula was as follows (detailed formula development is described in the “Methods” section): HRI = (−0.140) *∗* CD177 expression + 0.045 *∗* CP expression + 0.006 *∗* RGS16 expression + 0.013 *∗* PGM5 expression + 0.206 *∗* SNAI1 expression + 0.010 *∗* CALB2 expression + 0.041 *∗* OSBPL1A expression + 0.043 *∗* CDR2L expression + 0.012 *∗* FRMD5 expression + 0.096 *∗* FSTL3 expression + 0.069 *∗* TUBB2B expression. The HRI scores for CC patients in TCGA-COAD cohort were calculated using the previous HRI calculation formula (Table S10). Patients were assigned into the HRI high- or low-risk groups based on the median HRI score. Survival analysis uncovered that the HRI high-risk group exhibited a significantly poorer OS outcome than the low-risk group (*p* = 6.321*e* − 06, Figure 4(b)). Time-dependent ROC curves showed that the areas under the curve (AUCs) of 1-, 3-, 5-, and 6-year survival predictions were 0.682, 0.699, 0.768, and 0.753, respectively (Figure 4(c)), indicating good prognostic prediction. To further validate the reliability of the signature, the HRI scores for 210 patients in the validation cohort GSE17538 were calculated using the same formula (Table S11). Because the data type of dataset GSE17538 (microarray data) was different from that of the TCGA-COAD cohort (sequencing data), we classified all patients in GSE17538 into HRI high-risk or low-risk groups according to the median HRI score of the dataset GSE17538. Similarly, the HRI high-risk group possessed a poorer OS prognosis than the low-risk counterpart (*p* = 7.956*e* − 06, Figure 4(d)). The AUCs for 1-, 3-, 5-, and 6-year survival predictions were 0.647, 0.645, 0.716, and 0.754, respectively (Figure 4(e)). These results verified the robustness and reliability of the HRI signature in different platform datasets.

Subsequently, we investigated the relationship between HRI scores and HIF1A mRNA expression. Notably, the HRI high-risk group consistently showed higher HIF1A expression than the low-risk counterpart in both TCGA-COAD (Figure 4(f)) and GSE17538 (Figure 4(g)), demonstrating that the HRI scores indeed reflected the hypoxic exposure level in CC tissues.

### 3.5. Correlation between the HRI Signature and Clinical Parameters

**Figure 5:**
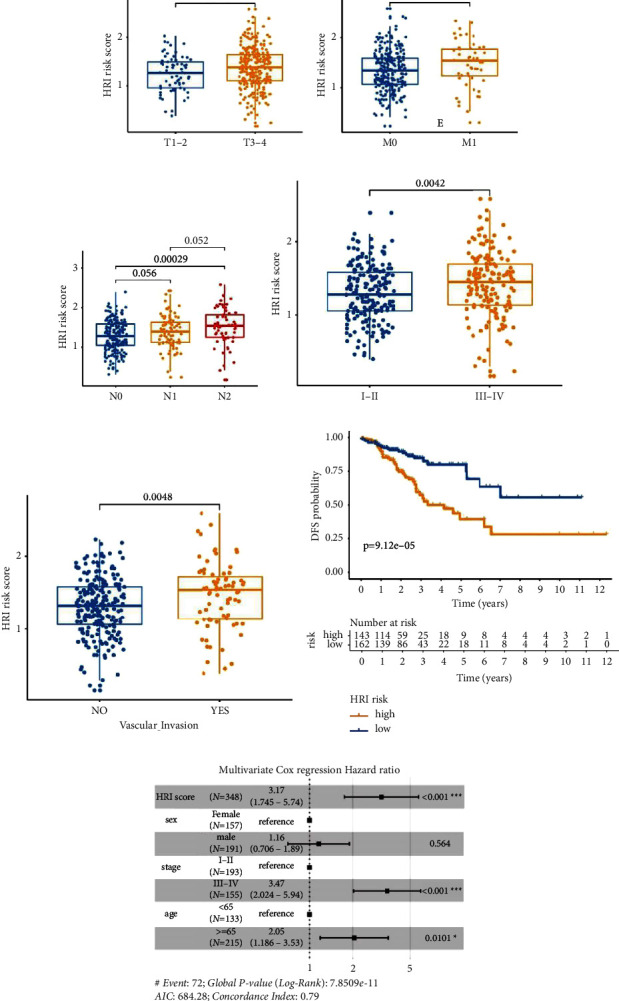
Correlation between the HRI score and clinical parameters in TCGA-COAD cohort. Comparison of HRI scores between different clinical subgroups, including T (a), M (b), N (c), stage (d), and vascular invasion (e). Disease-free survival (DFS) difference between HRI high-risk and low-risk groups (f). Forest plot for multivariate Cox regression analysis in TCGA-COAD (g). HRI: hypoxia-related index. ^*∗∗∗*^*p* < 0.001; ^*∗∗*^*p* < 0.01; ^*∗*^*p* < 0.05.

Owing to the remarkable impact of the HRI scores on the patient's clinical outcomes, we investigated the correlation between HRI scores and several critical clinical parameters. Results for TCGA-COAD cohort indicated that patients with T3-4, M1, N2, stage III-IV, and “Vascular Invasion” possessed higher HRI scores than patients with T1-2, M0, N0, stage I-II, and “nonvascular invasion,” respectively (Figures 5(a)–5(e)). Furthermore, patients with stage III-IV, histopathological grade 3, and recurrence in GSE17538 had elevated HRI scores compared with patients with stage I-II, grade 1 or 2, and nonrecurrence, respectively (Figures S3A–S3C). Additionally, patients with high HRI scores had poorer disease-free survival outcomes in TCGA-COAD (*p* = 9.12*e* − 05, Figure 5(f)) and poorer recurrence-free survival outcomes in GSE17538 (*p* = 0.006, Figure S3D) than patients with low HRI scores. 

To identify the independent predictive ability of the HRI signature, a multivariable Cox regression model was further performed on the TCGA-COAD and GSE17538 datasets. The results indicated that age, AJCC stage, and HRI scores were independent prognostic predictors after adjusting for other clinical parameters such as sex in TCGA-COAD cohort (Figure 5(g)). Similarly, stage and HRI risk scores were consistently independent of age, sex, and histopathological grade in GSE17538 (Figure S3E). The previous evidence demonstrated that the HRI signature can act as an independent indicator of prognosis in CC.

### 3.6. Different Molecular Patterns, TIME, and Immunotherapy Response between the High- and Low-Risk Groups

**Figure 6:**
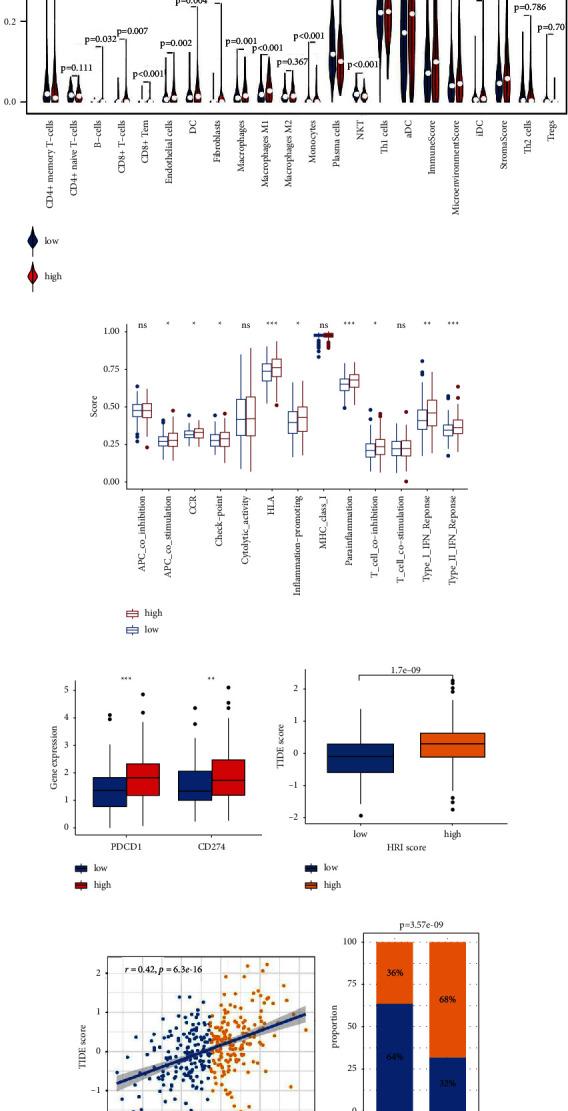
The distinct immune infiltration patterns in TCGA-COAD cohort. Comparison of the immune infiltration level (a), immune pathway activity (b), and immune checkpoint expression level (c) between HRI high-risk and low-risk groups. Comparison of TIDE scores between HRI high-risk and low-risk groups in TCGA-COAD (d). Correlation between TIDE scores and HRI scores in TCGA-COAD (e). Comparison of responder proportion predicted by the TIDE algorithm in TCGA-COAD (f). TIDE: tumor immune dysfunction and exclusion. HRI: hypoxia-related index. ^*∗∗∗*^*p* < 0.001; ^*∗∗*^*p* < 0.01; ^*∗*^*p* < 0.05; ns, no significance.

To further explore the underlying molecular mechanism, we investigated the different molecular patterns and TIME between the two HRI risk groups. GSEA results displayed that several critical hallmark pathways, including “APICAL_JUNCTION,” “APICAL_SURFACE,” “ANGIOGENESIS,” “HYPOXIA,” “EPITHELIAL_MESENCHYMAL_TRANSITION,” and “P53_PATHWAY,” were substantially enriched in the high-risk group in both TCGA-COAD (Figure S4A) and GSE17538 (Figure S4C) datasets. Furthermore, KEGG pathways such as “ADHERENS_JUNCTION,” “FOCAL_ADHESION,” and “PATHWAYS_IN_CANCER” were significantly enriched in the group with high HRI scores in both TCGA-COAD (Figure S4B) and GSE17538 (Figure S4D) datasets. These results suggest that hypoxia contributes to tumor aggression through the abovementioned oncogenic pathways. The “xCell” algorithm revealed that the high-risk group holds a higher abundance of macrophages, fibroblasts, and endothelial cells and higher stroma scores and microenvironment scores than the low-risk group in TCGA-COAD (Figure 6(a)). The high-risk group in GSE17538 possessed a higher infiltrating level of macrophages and higher immune scores and microenvironment scores than the low-risk counterpart (Figure S5A). The ssGSEA results displayed that the high-risk group consistently possessed higher scores in several critical immune pathways such as “check−point” and “T_cell_co−inhibition” than the low-risk group in both TCGA-COAD (Figure 6(b)) and GSE17538 (Figure S5B) cohorts. Moreover, the mRNA expression level of PD-L1 (CD274) was significantly elevated in the HRI high-risk group compared with the low counterpart in both TCGA-COAD (Figure 6(c)) and GSE17538 (Figure S5C) cohorts, suggesting distinct immune infiltration characteristics between the two groups.In addition, compared with the hypoxia-low cluster, the hypoxia-high clusterconsistently presented higher immune scores, stromal scores, and elevatedinfiltration levels of several critical immune and stromal cells (endothelialcells, fibroblasts, macrophages, dendritic cells, CD8+ T cells, CD4+ memory Tcells, B cells, and monocytes) in both TCGA-COAD and GSE17538 (Figure S7A-B). Theabove evidence demonstrated that elevated hypoxia exposure levels in CC tissuescorrelated with higher stromal and immune cell infiltration.

Using the TIDE algorithm, we estimated the TIDE scores for CC patients in the TCGA-COAD (Table S12) and GSE17538 (Table S13), respectively. Patients in the HRI high-risk group possessed higher TIDE scores than the corresponding low-risk patients in both TCGA-COAD (Figure 6(d)) and GSE17538 (Figure S5D). Moreover, HRI scores consistently displayed a positive correlation with the TIDE scores in TCGA-COAD (Figure 6(e)) and GSE17538 (Figure S5E), indicating that higher HRI scores represent greater immune evasion and immunotherapeutic resistance. Accordingly, the low-risk group was predicted to have a significantly higher ratio of immunotherapeutic responders than the high-risk group in both TCGA-COAD (Figure 6(f)) and GSE17538 (Figure S5F). The previous results demonstrated that HRI scores representing hypoxia levels in CC tissues have the potential to predict the immunotherapy response.

### 3.7. Correlation Analysis of MSI Status with HRI Signature

The MSI status information for CC patients in the TCGA-COAD cohort was curated from TCIA (The Cancer Immunome Atlas) database (https://tcia.at/home) [25]. There were 335 CC patients with complete MSI status in our TCGA-COAD dataset, including 46 MSI-H, 64 MSI-L, and 210 MSS (microsatellite stability), and 15 indeterminate cases, respectively. The chi-square test revealed a statistically significant difference in the constitutive proportion of MSI status between the two HRI risk groups (*p* = 0.019, Figure S6A). The HRI high-risk group presented an elevated ratio of MSI-H (17%) and MSI-L (25%) cases compared with the low-risk counterpart (11% and 15% of MSI-H and MSI-L cases, respectively). Subsequently, we stratified CC patients into different subgroups according to their MSI status and performed a subgroup survival analysis. Notably, patients with high HRI scores consistently exhibited poorer OS prognosis than those in the HRI low-risk group, irrespective of MSI status (Figures S6B–S6D).

### 3.8. Validating the mRNA Expression of Five Key Genes by qRT-PCR

**Figure 7:**
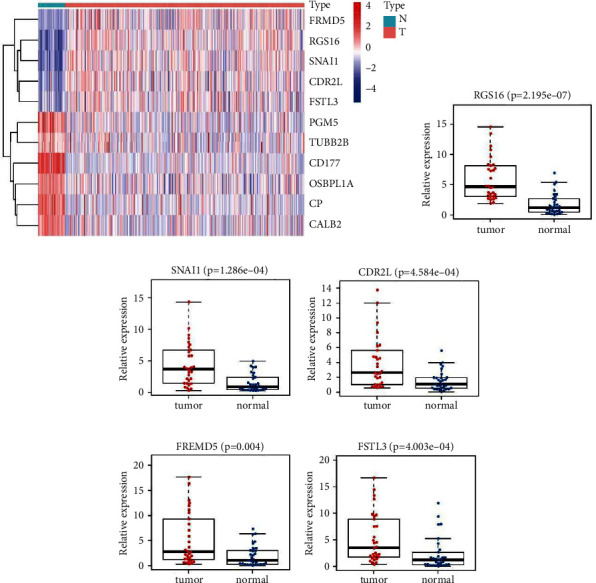
Verification of the mRNA expression levels of the five key genes in HRI signature by qRT-PCR. (a) Heatmap of the mRNA expression levels of all eleven genes in HRI signature in TCGA-COAD cohort. Comparison of relative mRNA expression levels of RGS16 (b), SNAI1 (c), CDR2L (d), FRMD5 (e), and FSTL3 (f) between 30 pairs of CC tumor and adjacent normal tissues using the qRT-PCR experiment. HRI: hypoxia-related index. qRT-PCR: quantitative reverse transcription polymerase chain reaction. N: normal; T: tumor; CC: colon cancer.

The HRI signature consisted of 11 HRDEGs, namely, CD177, CP, RGS16, PGM5, SNAI1, CALB2, OSBPL1A, CDR2L, FRMD5, FSTL3, and TUBB2B. Among the HRI prognostic signatures, CD177 was the only protective factor, and the other 10 genes were all risk factors for prognostic prediction in CC. The mRNA expression levels of RGS16, SNAI1, CDR2L, FRMD5, and FSTL3 were higher in tumor samples than that in adjacent normal tissues in TCGA-COAD cohort (Figure 7(a)), suggesting that these five key genes participate in the progression of CC. Thus, we experimentally investigated their mRNA expression levels in 30 pairs of clinical samples by qRT-PCR. The results demonstrated that RGS16 (Figure 7(b)), SNAI1 (Figure 7(c)), CDR2L (Figure 7(d)), FRMD5 (Figure 7(e)), and FSTL3 (Figure 7(f)) consistently exhibited significantly higher relative mRNA expression levels in CC tumor samples than in paired adjacent normal tissues.

### 3.9. The Validation Experiment *In Vitro*

**Figure 8:**
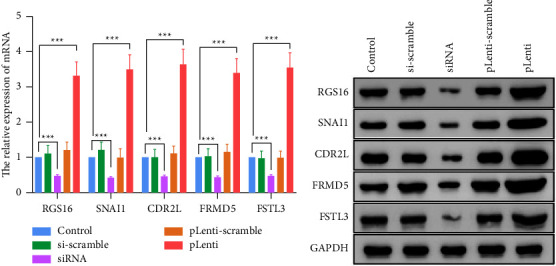
Transfection efficiency. Comparison of relative mRNA expression levels of RGS16, SNAI1, CDR2L, FRMD5, and FSTL3 of tumor cell line using the qRT-PCR experiment (a) and western blot experiment (b). ^*∗∗∗*^*p* < 0.001; ^*∗∗*^*p* < 0.01; ^*∗*^*p* < 0.05.

**Figure 9:**
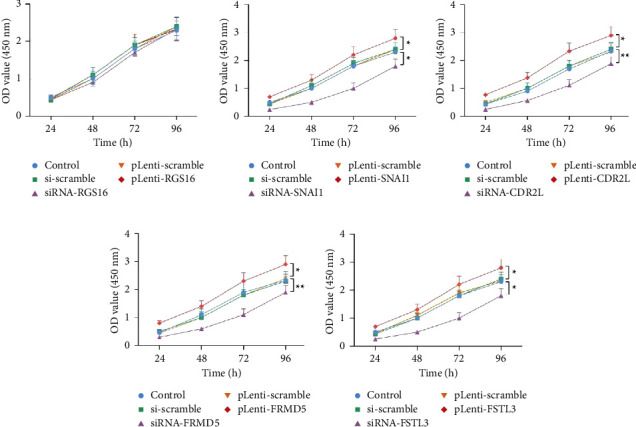
The CCK-8 assay. The effect of expression of RGS16 (a), SNAI1 (b), CDR2L (c), FRMD5 (d), and FSTL3 (e) on cell viability was examined using the CCK-8 assay. ^*∗∗∗*^*p* < 0.001; ^*∗∗*^*p* < 0.01; ^*∗*^*p* < 0.05.

**Figure 10:**
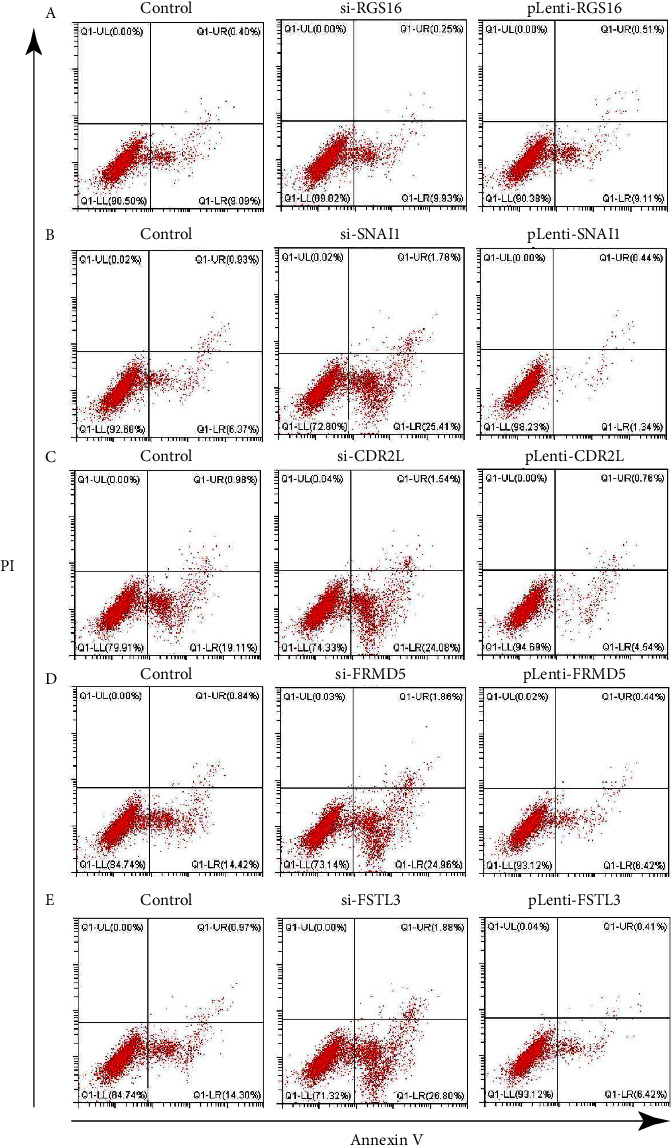
The flow cytometry assay. The effect of expression of RGS16 (a), SNAI1 (b), CDR2L (c), FRMD5 (d), and FSTL3 (e) on cell viability was examined using the flow cytometry assay.

To further determine the influence of the previously selected differentially expressed genes (RGS16, SNAI1, CDR2L, FRMD5, and FSTL3) on cell proliferation and apoptosis, we interfered with the expression of five differential genes and detected the cell proliferation and apoptosis levels. First, we tested the transfection level of the disruptor or plasmid. The results showed that the expression of mRNA (Figure 8(a)) and protein levels (Figure 8(b)) in the siRNA group was induced, while the pLenti group could significantly upregulate the expression of mRNA and protein levels of genes. Subsequently, we detected the cell activity by CCK-8 experiment. The results showed that the expression of RGS16 had no significant effect on cell proliferation and apoptosis (Figures 9(a) and 10(a)), while the high expression of SNAI1, CDR2L, FRMD5, and FSTL3 could promote the proliferation of cancer cells and inhibit the apoptosis of cancer cells, but inhibiting their expression could inhibit the proliferation of cancer cells and promote the apoptosis of cancer cells (Figures 9(b)–9(e) and 10(b)–10(e)).

## 4. Discussion

CC ranks the fifth most frequent malignant disease worldwide, and advanced-stage cases are associated with high mortality [2]. Thus, it is urgent to identify novel prognostic predictors and targeted biomarkers. Hypoxia in the tumor microenvironment is a specific hallmark of solid tumors [5] and contributes to the tumor metastatic cascade [6]. Several studies have constructed different hypoxia-related gene signatures for predicting the clinical outcomes of colorectal cancer [26–28]. However, these studies mainly aimed to establish a prognostic signature for CC patients and lacked comprehensive microdissection of the hypoxia landscape and its correlation with immunotherapy in CC. Compared with the previously published literature, we identified three hypoxia-specific clusters and developed a novel HRI prognostic signature. As far as we know, this is the first comprehensive investigation of the correlation of the hypoxia landscape with metabolic reprogramming, TIME, and immunotherapeutic response prediction in CC.

The hypoxic tumor microenvironment in solid tumors maintains a selective pressure for tumor cells to adapt to the hypoxia response and promotes their invasion, migration, and dissemination [6]. Moreover, the HIF-1*α* pathway plays a pivotal role in the modulation of hypoxia-related downstream gene expression and biological processes in cancer cells under hypoxic conditions [7]. Hence, we classified the CC patients into three different clusters based on the expression levels of the 200 genes in the “HALLMARK_HYPOXIA” gene set and verified the relationship between the clusters and the HIF1A mRNA expression level. Hypoxia stress can decrease the expression of DUSP2 and increase cancer stemness and tumor growth in CC cells [29]. Hypoxia may promote EMT, invasion, and migration of CC cells by activation of HIF1A, whereas treatment with HIF1A-specific siRNAs suppresses these processes [8]. In agreement with the findings of the abovementioned studies, the hypoxia-high cluster in our study possessed a higher HIF1A mRNA expression and elevated relative scores in tumor aggression-associated pathways including “EPITHELIAL_MESENCHYMAL_TRANSITION” and “ANGIOGENESIS.” Accordingly, the hypoxia-high cluster had more CC patients with “Vascular_Invasion” and “Recurred/Progressed.”

Cancer cells undergo metabolic reprogramming to reconcile themselves to hypoxic stress [30]. The HIF-1*α* pathway contributes greatly to metabolism alteration via glycolysis stimulation and oxidative phosphorylation (OXPHOS) suppression under hypoxic conditions during tumor development [14]. Upon hypoxic conditions, pancreatic ductal adenocarcinoma cells exhibited elevated HIF1A and HIF2A expression levels, increased expression of carbonic anhydrase 9, and activated glycolysis [31]. Our study also showed similar results in both TCGA-COAD and GSE17538 datasets. This phenomenon may be because of the cancer cells' metabolic plasticity and metabolic heterogeneity, depending on the complex tumor microenvironment [32].

Hypoxia stress also impacts TIME by inducing an immune suppression or immune evasion phenotype [33]. The local hypoxic microenvironment recruits immunosuppressive cells, such as MDSCs, tumor-associated macrophages, and CAFs, and upregulates immune checkpoint expression to induce antitumor resistance [15]. CAFs at the invasive front of tumor tissues boost tumor progression and metastasis in CC [34]. In our study, the hypoxia-high cluster consistently had a higher immune score, stromal score, and estimate score than the hypoxia-low cluster. Furthermore, macrophages and fibroblasts showed elevated infiltrating levels in the hypoxia-high group, supporting a positive correlation between hypoxia and tumor immune dysfunction. Accordingly, we speculate that the poor clinical outcomes of the patients in the hypoxia-high group partly depend on the immune suppression or evasion mechanism.

To further examine the clinical applicability, we developed a reliable HRI prognostic signature that is strongly correlated with critical clinical characteristics (T, N, M, AJCC stage, and tumor histological grade). Hypoxia-treated CC cells have been reported to strengthen the metastatic ability of normoxic cancer cells [35]. HIF-1*α* is a master regulator of the hypoxia-response process of tumor cells under hypoxic conditions [7]. Hypoxia can promote EMT, invasion, and migration of CC cells by the activation of HIF1A [8]. In our study, the high HRI score group consistently possessed a higher HIF1A expression level than the low-risk counterpart, indicating the effectiveness of the HRI score to reflect hypoxia exposure in CC tumor tissues. Additionally, patients with M1, N2, T3–4, stage 3–4, and tumor grade 3 had higher HRI risk scores than those with M0, N0, T1–2, stage 1–2, and grade 1, respectively. This further demonstrated that the HRI signature was strongly related to tumor progression and metastasis in CC.

Immunotherapy involving anti-PD-1/PD-L1 has resulted in revolutionary therapeutic benefits for various cancer types, and MSI-H status has proven to be an effective predictor of immunotherapeutic efficacy [36]. However, MSI-L/MSS patients, who represent most CC patients, have not acquired a satisfactory response from immunotherapy. High tumor mutational burden (TMB) in tumors is linked to favorable clinical outcomes; however, the TMB varies markedly among different cancer types, and there is a lack of a well-defined standard of high TMB [37]. Our study demonstrated that the HRI signature is positively correlated with the TIDE score, which represents the immune dysfunction and exclusion of tumor samples, and patients with a low HRI score are predicted to be more responsive to immunotherapy. The previous evidence suggests that the HRI score has the potential to be a complementary measure to MSI-H status and TMB in the personalized management of immunotherapy. Hypoxia gene sets were reported to be enriched in nonresponding pre-anti-PD-1 tumor samples with melanoma [38]. In agreement with these studies, CC patients with a high HRI score representing severe hypoxia exposure are predicted to have a lower response to immunotherapy. As targeting the hypoxic microenvironment may ameliorate the effects of cancer immunotherapy [16], we speculate that these patients with high HRI scores may acquire greater efficacy of immunotherapy in combination with antihypoxia drugs.

The HRI signature consists of 11 HRDEGs, and we focused on the 5 key genes (RGS16, SNAI1, CDR2L, FRMD5, and FSTL3), which exhibited elevated expression levels in tumor tissues and are prognostic risk factors for CC. RGS16 has already been reported to possess a higher expression level in colorectal cancer tissue than in the corresponding normal tissue and serves as an unfavorable prognostic marker [39]. Overexpression of SNAI1 (also known as SNAIL) is linked to increased stemness and decreased radiation sensitivity in CC cells [40]. A previously published study [41] reported the FRMD5 is a novel downstream gene targeted by the *β*-catenin/TCF7L2 complex in CC cells. CDR2L is widely present in ovarian cancer tissues and is abundantly expressed in testicular and prostate cancer tissues [42]. Knockdown of FSTL3 remarkably inhibited the aggression phenotype of lung cancer cells [43]. In the subsequent cell activity and apoptosis experiments, we found that the low expression of SNAI1, CDR2L, FRMD5, and FSTL3 could reduce the activity of cancer cells and increase the apoptosis rate of cancer cells. But RGS16 does not exhibit similar functions. According to previous literature reports, high expression of SNAI1 can promote the invasion ability of cancer cells [44], low expression of FRMD5 can weaken the metastatic ability of cancer cells [45, 46], and low expression of FSTL3 also has similar functions [47, 48]. The reason why RGS16 has no similar function may be that its mechanism of affecting prognosis is different from other genes. According to previous reports, the population with low expression of RGS16 presents a better prognosis than the population with high expression [39]. Therefore, RGS16 may affect the prognosis of patients by regulating the activity of immune cells and has no direct impact on the activity and apoptosis rate of cancer cells [49]. Collectively, these five key genes may act as oncogenic genes that contribute to the progression of CC, and their molecular mechanism is worth further studying to explore new therapeutic targets.

Nevertheless, there are still several limitations to our study. The HRI signature was identified in TCGA-COAD cohort and validated in another independent dataset, but these public datasets are mostly attributed to retrospective studies and may induce indispensable biases to some extent. Thus, prospective research will be required at a future date. Furthermore, although the HRI score is demonstrated to have a reliable predictive capability of immunotherapy response in CC by bioinformatical analysis, well-designed clinical trials should be performed to further prove its clinical effectiveness.

## 5. Conclusion

In conclusion, we discover three hypoxia clusters (hypoxia-H, hypoxia-L, and hypoxia-M) which correlate with the clinical outcome and the tumor immune microenvironment in CC. Furthermore, we found 600 HRDEs. Based on the 600 HRDEGs, we constructed a reliable HRI signature that could accurately predict the prognosis and immunotherapeutic responsiveness in CC patients. Finally, we discover five key genes which are differently expressed between tumors and adjacent tissues. Of them, four genes could affect tumor cell viability and apoptosis.

## Data Availability

The expression profiles of GSE17538 were downloaded from the Gene Expression Omnibus (GEO) database (https://www.ncbi.nlm.nih.gov/geo/), and the expression profile of The Cancer Genome Atlas Colon Adenocarcinoma (TCGA-COAD) project was downloaded from TCGA database (https://portal.gdc.cancer.gov/).

## Supplementary Materials

Supplementary Materials The following are available supplementary materials: Figure S1: microdissection of the hypoxia landscape in TCGA-COAD cohort and GSE17538. (A) Consensus CDF curve and (B) delta area curve, when *k* = 3 represents the optimal cluster number in TCGA-COAD cohort. (C) Consensus CDF curve and (D) delta area curve, when *k* = 3 represents the optimal cluster number in the GSE17538 cohort; Figure S2: construction of the hypoxia-related index signature. (A) The tuning parameters of the LASSO penalty Cox regression. (B) Cross-validation of the LASSO regression model, the left vertical dashed line means the “lambda. min” standard. LASSO: least absolute shrinkage and selection operator; Figure S3: correlation between the HRI score and clinical parameters in GSE17538. Comparison of HRI scores between different clinical subgroups, including stage (A), grade (B), and recurrence (C). Recurrence-free survival (RFS) difference between HRI high-risk and low-risk groups (D). Forest plot for multivariate Cox regression analysis in GSE17538 (E). HRI: hypoxia-related index. ^*∗∗∗*^*p* < 0.001; ^*∗∗*^*p* < 0.01; ^*∗*^*p* < 0.05; Figure S4: GSEA results of TCGA-COAD cohort and GSE17538 dataset. The significantly enriched pathway in the HRI high-risk group versus the low-risk group for hallmark gene sets (A) and KEGG pathways (B) in TCGA-COAD cohort. The significantly enriched pathway in the HRI high-risk group versus the low-risk group for hallmark gene sets (C) and KEGG pathways (D) in the GSE17538 dataset. GSEA: gene set enrichment analysis. HRI: hypoxia-related index. KEGG: Kyoto Encyclopedia of Genes and Genomes; Figure S5: the distinct immune infiltration patterns in GSE17538. Comparison of the immune infiltrating level (A), immune pathway activity (B), and immune checkpoint expression level (C) between HRI high-risk and low-risk groups. Comparison of TIDE scores between HRI high-risk and low-risk groups in GSE17538 (D). Correlation between TIDE scores and HRI scores in GSE17538 (E). Comparison of responder proportion predicted by the TIDE algorithm in GSE17538 (F). TIDE: tumor immune dysfunction and exclusion. HRI: hypoxia-related index. ^*∗∗∗*^*p* < 0.001; ^*∗∗*^*p* < 0.01; ^*∗*^*p* < 0.05; ns, no significance; Figure S6: relationship between HRI scores and MSI status in TCGA-COAD. (A) Comparison of distribution difference of MSI status between HRI high-risk and low-risk groups. Survival differences between HRI high-risk and low-risk groups according to MSI status, including MSI-H (B), MSI-L (C), and MSS (D). HRI: hypoxia-related index. MSI: microsatellite instability. MSS: microsatellite stability; Figure S7. The distinct tumor immune infiltration pattern from the “xCell” algorithm. Comparison of the tumor immune infiltration level among hypoxia-specific clusters in TCGA-COAD (A) and GSE17538 (B). hypoxia-L: hypoxia-low; hypoxia-M:hypoxia-median; hypoxia-H: hypoxia-high. ^*∗∗∗*^*p* < 0.001; ^*∗∗*^*p* < 0.01; ^∗^*p* < 0.05; ns, no significance; Table S1: clinicopathological characteristics of patients enrolled in the present study; Table S2: the detailed gene list of the HALLMARK_HYPOXIA gene set; Table S3: the detailed gene list of 13 metabolic pathway gene sets; Table S4: the detailed gene list of 13 immune function pathway gene sets; Table S5: the primer sequences used in the present study; Table S6: clinical parameters of 348 patients in TCGA-COAD cohort; Table S7: clinical parameters of 210 patients in the GSE17538 dataset; Table S8: the detailed gene list of the 600 hypoxia-related differentially expressed genes; Table S9: the optimal model of LASSO Cox regression; Table S10: HRI risk scores of 348 patients in TCGA-COAD cohort; Table S11: HRI risk scores of 210 patients in the GSE17538 dataset; Table S12: TIDE scores of 348 patients in TCGA-COAD cohort; Table S13: TIDE scores of 210 patients in GSE17538.
