# Supplementary material for: Microdissecting the Hypoxia Landscape in Colon Cancer Reveals Three Distinct Subtypes and Their Potential Mechanism to Facilitate the Development of Cancer
Source: J Oncol. 2023 Mar 7;2023:9346621. doi: 10.1155/2023/9346621 (PMC10014161; doi:10.1155/2023/9346621)
Supplement: Supplementary Materials — The following are available supplementary materials: Figure S1: microdissection of the hypoxia landscape in TCGA-COAD cohort and GSE17538. (A) Consensus CDF curve and (B) delta area curve, when k = 3 represents the optimal cluster number in TCGA-COAD cohort. (C) Consensus CDF curve and (D) delta area curve, when k = 3 represents the optimal cluster number in the GSE17538 cohort; Figure S2: construction of the hypoxia-related index signature. (A) The tuning parameters of the LASSO penalty Cox regression. (B) Cross-validation of the LASSO regression model, the left vertical dashed line means the “lambda. min” standard. LASSO: least absolute shrinkage and selection operator; Figure S3: correlation between the HRI score and clinical parameters in GSE17538. Comparison of HRI scores between different clinical subgroups, including stage (A), grade (B), and recurrence (C). Recurrence-free survival (RFS) difference between HRI high-risk and low-risk groups (D). Forest plot for multivariate Cox regression analysis in GSE17538 (E). HRI: hypoxia-related index. ∗∗∗p < 0.001; ∗∗p < 0.01; ∗p < 0.05; Figure S4: GSEA results of TCGA-COAD cohort and GSE17538 dataset. The significantly enriched pathway in the HRI high-risk group versus the low-risk group for hallmark gene sets (A) and KEGG pathways (B) in TCGA-COAD cohort. The significantly enriched pathway in the HRI high-risk group versus the low-risk group for hallmark gene sets (C) and KEGG pathways (D) in the GSE17538 dataset. GSEA: gene set enrichment analysis. HRI: hypoxia-related index. KEGG: Kyoto Encyclopedia of Genes and Genomes; Figure S5: the distinct immune infiltration patterns in GSE17538. Comparison of the immune infiltrating level (A), immune pathway activity (B), and immune checkpoint expression level (C) between HRI high-risk and low-risk groups. Comparison of TIDE scores between HRI high-risk and low-risk groups in GSE17538 (D). Correlation between TIDE scores and HRI scores in GSE17538 (E). Comparison of responde [file 9346621.f1.docx]

**Supplementary Materials**

**
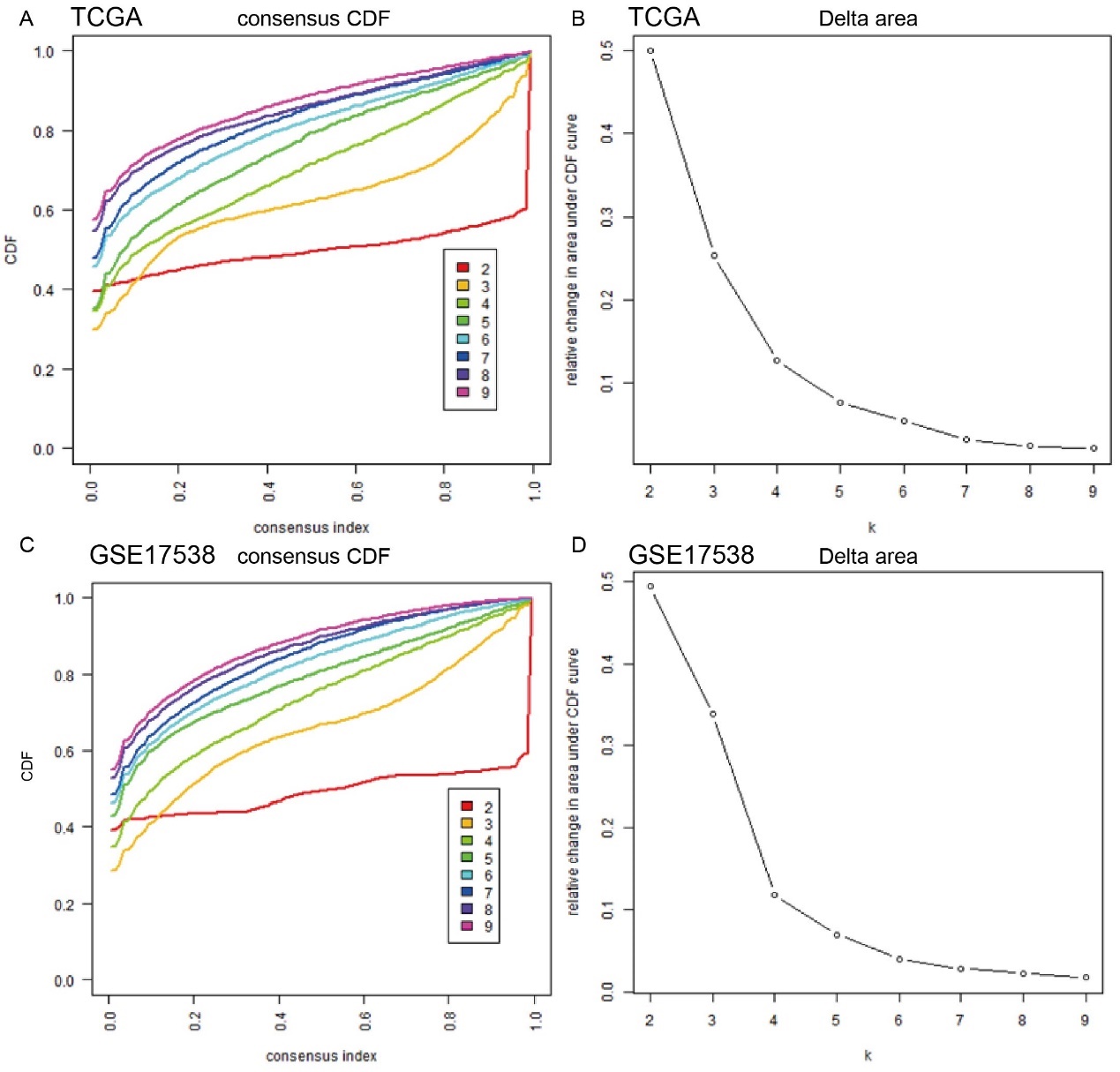
**

**Figure S1. Microdissection of the hypoxia landscape in** **TCGA-COAD cohort and GSE17538.** (A) Consensus CDF curve and (B) Delta area curve, when k = 3 representing the optimal cluster number in TCGA-COAD cohort. (C) Consensus CDF curve and (D) Delta area curve, when k = 3 representing the optimal cluster number in GSE17538 cohort.


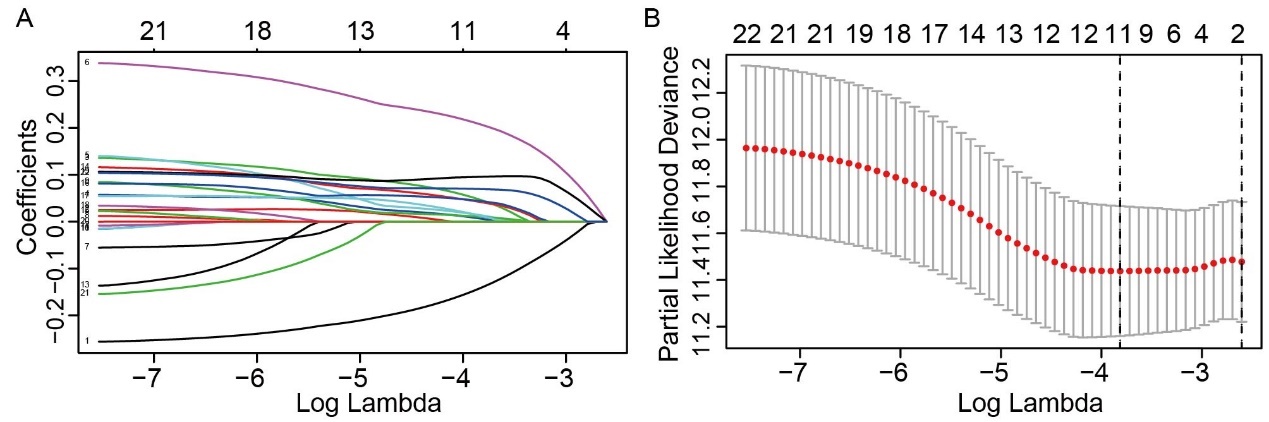


**Figure S2. Construction of the hypoxia-related index signature.** (A) The tuning parameters of the LASSO penalty Cox regression. (B) Cross-validation of the LASSO regression model, the left vertical dashed line means the “lambda. min” standard. LASSO: least absolute shrinkage and selection operator.


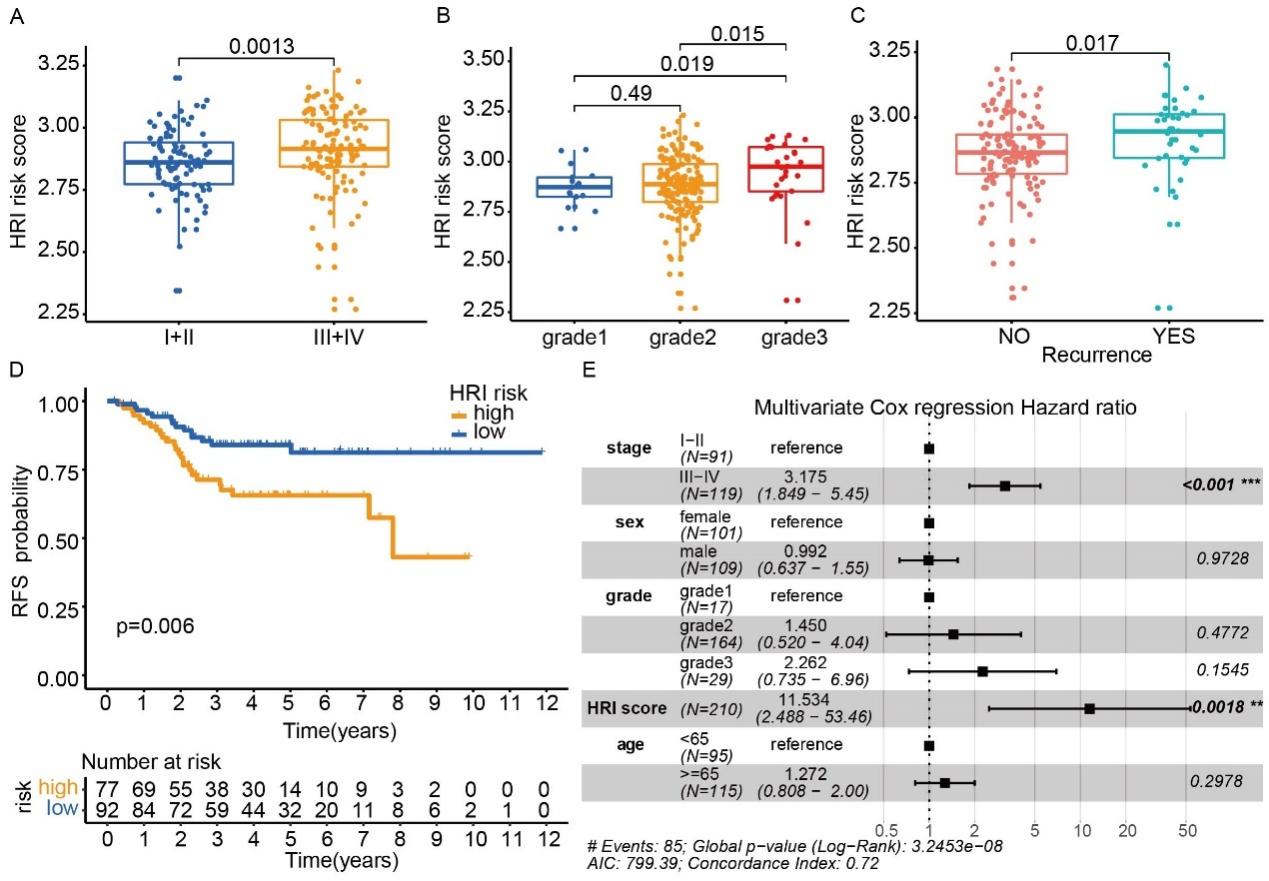


**Figure S3. Correlation between HRI score and clinical parameters in GSE17538.** Comparison of HRI scores between different clinical subgroups, including stage (A), grade (B), and recurrence (C). Recurrence-free survival (RFS) difference between HRI high-risk and low-risk groups (D). Forest plot for multivariate Cox regression analysis in GSE17538 (E). HRI: hypoxia-related index. ***, p<0.001; **, p<0.01; *, p<0.05.


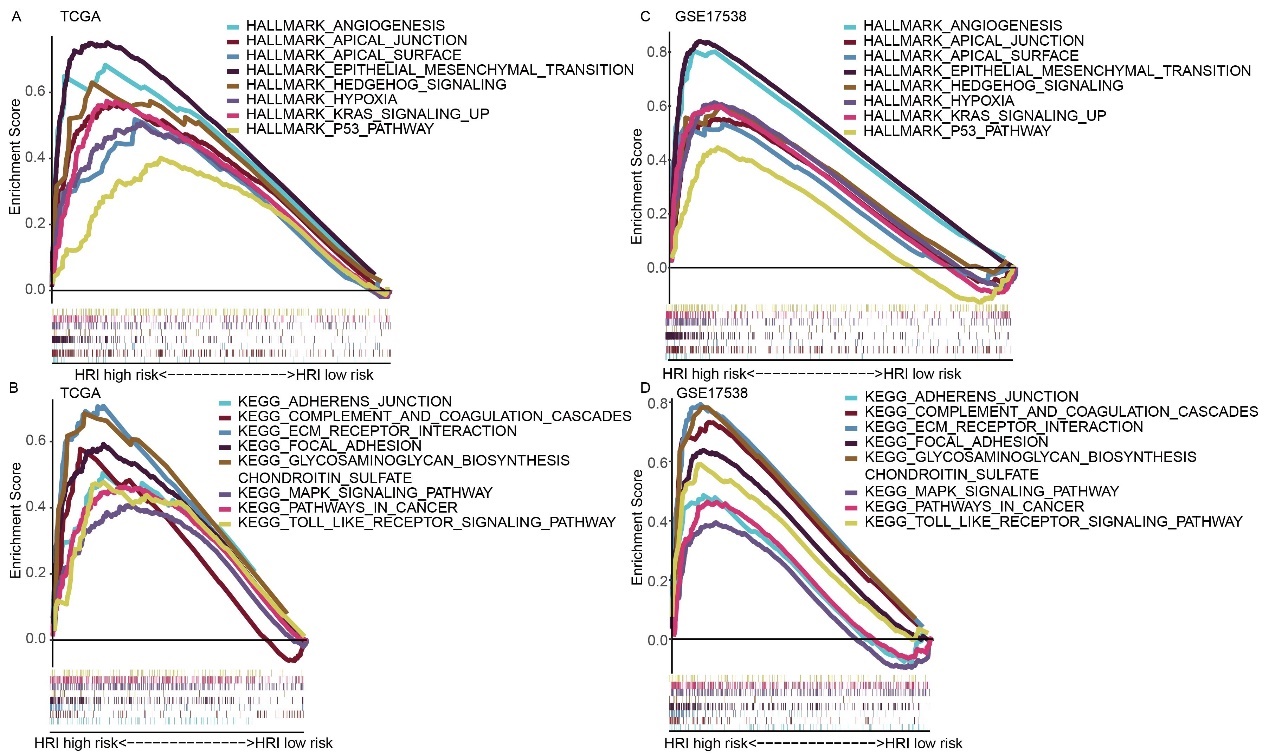


**Figure S4. GSEA results of the TCGA-COAD cohort and GSE17538 dataset.** Significantly enriched pathway in HRI high-risk group versus low-risk group for hallmark gene sets (A) and KEGG pathways (B) in TCGA-COAD cohort. Significantly enriched pathway in HRI high-risk group versus low-risk group for hallmark gene sets (C) and KEGG pathways (D) in GSE17538 dataset. GSEA: gene set enrichment analysis. HRI: hypoxia-related index. KEGG: Kyoto Encyclopedia of Genes and Genomes.


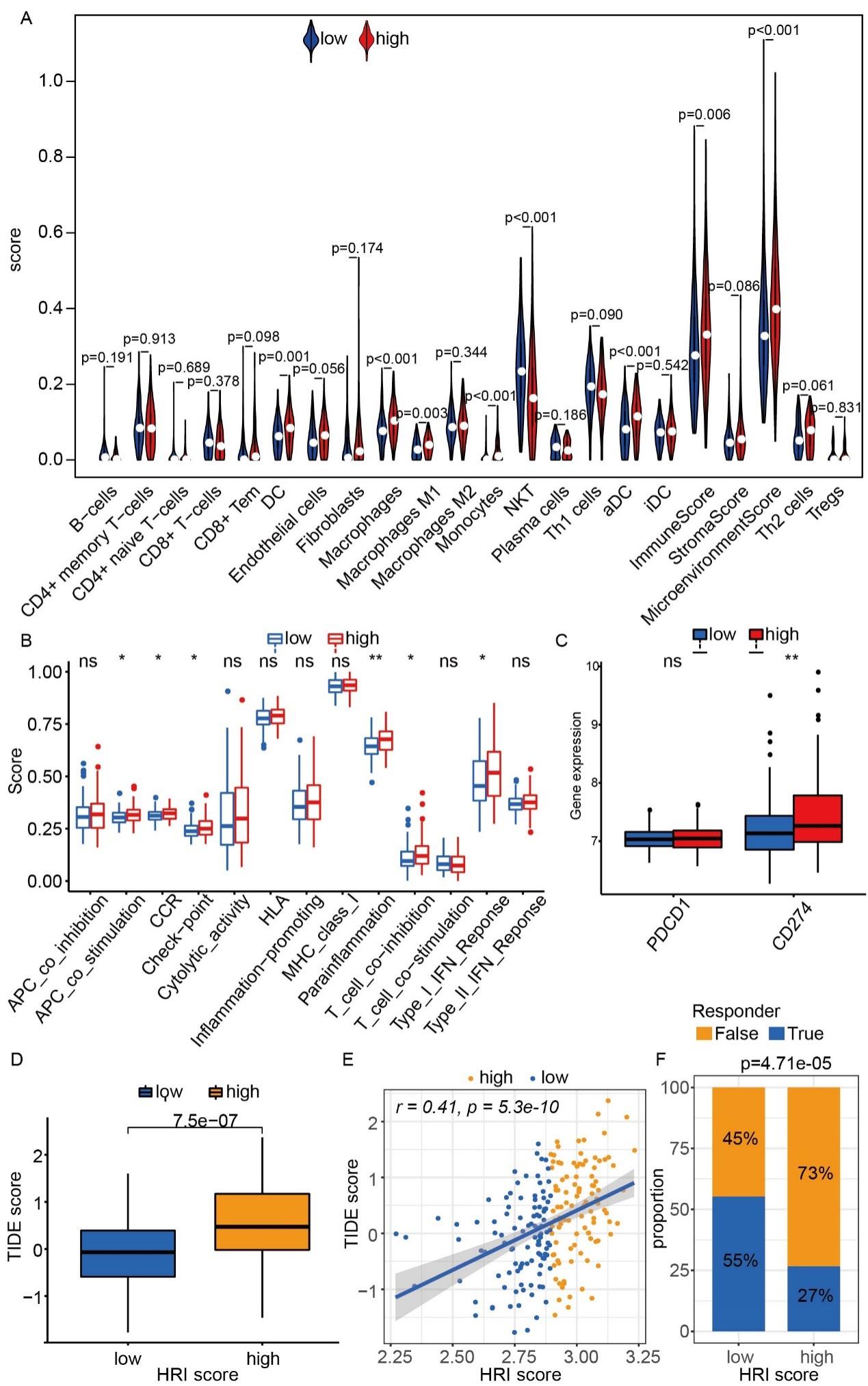


**Figure S5. The distinct immune infiltration patterns in GSE17538.** Comparison of the immune infiltrating level (A), immune pathway activity (B), and immune checkpoint expression level (C) between HRI high-risk and low-risk groups. Comparison of TIDE scores between HRI high-risk and low-risk groups in GSE17538 (D). Correlation between TIDE scores and HRI scores in GSE17538 (E). Comparison of responder proportion predicted by TIDE algorithm in GSE17538 (F). TIDE: Tumor Immune Dysfunction and Exclusion. HRI: hypoxia-related index. ***, p<0.001; **, p<0.01; *, p<0.05; ns, no significance.


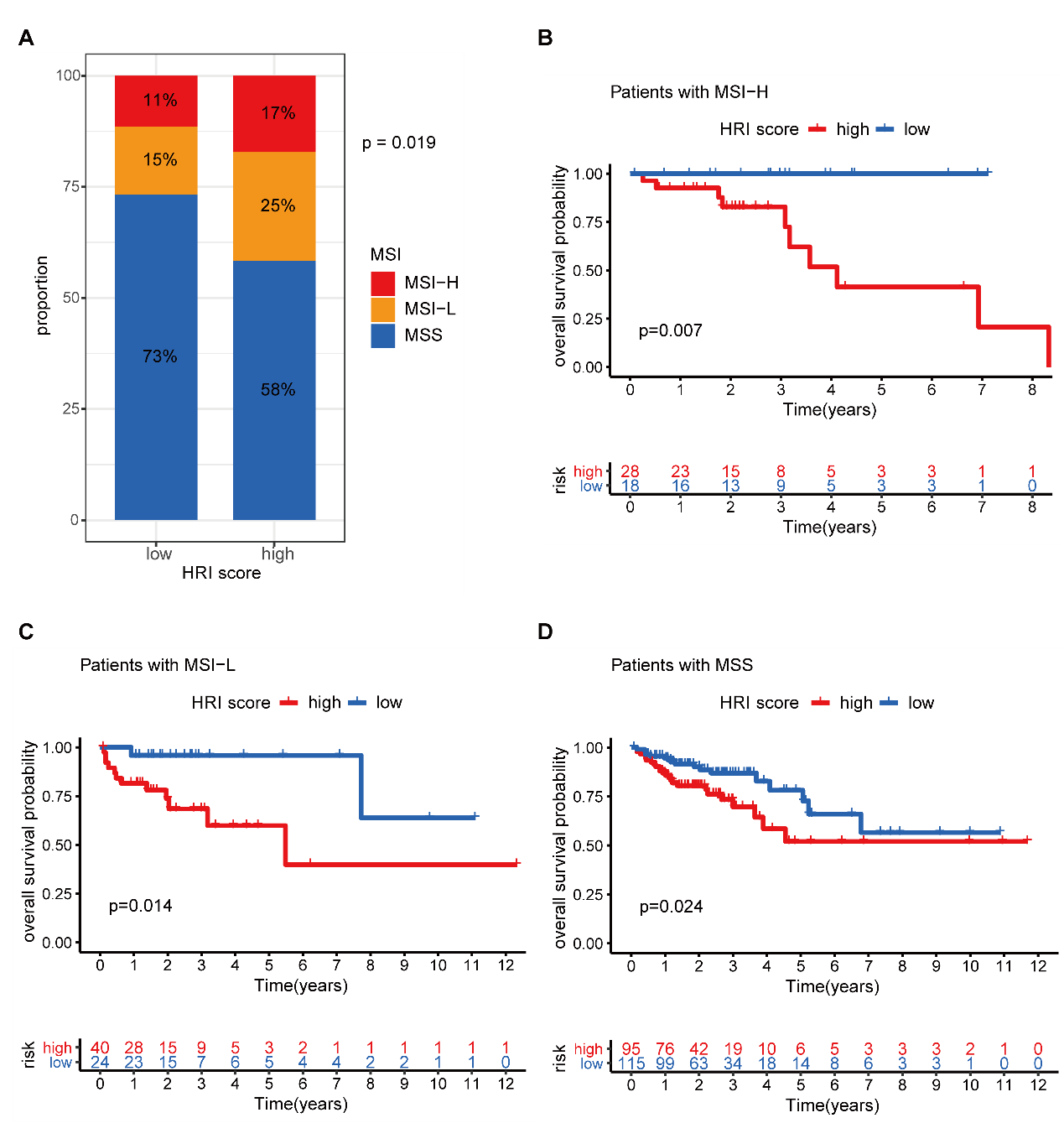


**Figure S6. Relationship between HRI scores and MSI status in TCGA-COAD.** (A) Comparison of distribution difference of MSI status between HRI high-risk and low-risk groups. Survival differences between HRI high-risk and low-risk groups according to MSI status, including MSI-H (B), MSI-L (C), and MSS (D). HRI: hypoxia-related index. MSI: microsatellite instability. MSS: microsatellite stability.

**Table S1 Clinicopathological characteristics of patients enrolled in the present study.**

| **Characteristic** | **dataset** | |
| --- | --- | --- |
|  | **TCGA-COAD** | **GSE17538** |
| **Total Patients** | 348 | 210 |
| **Age** |  |  |
| <65 years | 133 (38.2%) | 95 (45.2%) |
| >=65 years | 215 (61.8%) | 115 (54.8%) |
| **Sex** |  |  |
| Female | 157 (45.1%) | 101 (48.1%) |
| Male | 191 (54.9%) | 109 (51.9%) |
| **Tumor Grade** |  |  |
| grade 1 | NA | 17 (8.1%) |
| grage 2 | NA | 164 (78.1%) |
| grade 3 | NA | 29 (13.8%) |
| **Pathologic T Stage** |  |  |
| T1 | 8 (2.3%) | NA |
| T2 | 61 (17.5%) | NA |
| T3 | 242 (69.5%) | NA |
| T4 | 37 (10.6%) | NA |
| **Pathologic N Stage** |  |  |
| N0 | 201 (57.8%) | NA |
| N1 | 86 (24.7%) | NA |
| N2 | 61 (17.5%) | NA |
| **Pathologic M Stage** |  |  |
| M0 | 264 (75.9%) | NA |
| M1 | 53 (15.2%) | NA |
| unknown | 31 (8.9%) | NA |
| **Tumor Stage** |  |  |
| I | 59 (17.0%) | 27 (12.9%) |
| II | 134 (38.5%) | 64 (30.5%) |
| III | 102 (29.3%) | 69 (32.9%) |
| IV | 53 (15.2%) | 50 (23.8%) |
| **Survival Information Type** | OS, DFS | OS, RFS |
| OS: overall survival; DFS: disease-free survival; RFS: recurrence-free survival. | | |

**Table S2 The detailed gene list of the HALLMARK_HYPOXIA gene set.**

| ADM | DCN | HK1 | NDST2 | SAP30 |
| --- | --- | --- | --- | --- |
| ADORA2B | DDIT3 | HK2 | NEDD4L | SCARB1 |
| AK4 | DDIT4 | HMOX1 | NFIL3 | SDC2 |
| AKAP12 | DPYSL4 | HOXB9 | NR3C1 | SDC3 |
| ALDOA | DTNA | HS3ST1 | P4HA1 | SDC4 |
| ALDOB | DUSP1 | HSPA5 | P4HA2 | SELENBP1 |
| ALDOC | EDN2 | IDS | PAM | SERPINE1 |
| AMPD3 | EFNA1 | IER3 | PCK1 | SIAH2 |
| ANGPTL4 | EFNA3 | IGFBP1 | PDGFB | SLC25A1 |
| ANKZF1 | EGFR | IGFBP3 | PDK1 | SLC2A1 |
| ANXA2 | ENO1 | IL6 | PDK3 | SLC2A3 |
| ATF3 | ENO2 | ILVBL | PFKFB3 | SLC2A5 |
| ATP7A | ENO3 | INHA | PFKL | SLC37A4 |
| B3GALT6 | ERO1L | IRS2 | PFKP | SLC6A6 |
| B4GALNT2 | ERRFI1 | ISG20 | PGAM2 | SRPX |
| BCAN | ETS1 | JMJD6 | PGF | STBD1 |
| BCL2 | EXT1 | JUN | PGK1 | STC1 |
| BGN | F3 | KDELR3 | PGM1 | STC2 |
| BHLHE40 | FAM162A | KDM3A | PGM2 | SULT2B1 |
| BNIP3L | FBP1 | KIF5A | PHKG1 | TES |
| BRS3 | FOS | KLF6 | PIM1 | TGFB3 |
| BTG1 | FOSL2 | KLF7 | PKLR | TGFBI |
| CA12 | FOXO3 | KLHL24 | PKP1 | TGM2 |
| CASP6 | GAA | LALBA | PLAC8 | TIPARP |
| CAV1 | GALK1 | LARGE | PLAUR | TKTL1 |
| CCNG2 | GAPDH | LDHA | PLIN2 | TMEM45A |
| CCRN4L | GAPDHS | LDHC | PNRC1 | TNFAIP3 |
| CDKN1A | GBE1 | LOX | PPARGC1A | TPBG |
| CDKN1B | GCK | LXN | PPFIA4 | TPD52 |
| CDKN1C | GCNT2 | MAFF | PPP1R15A | TPI1 |
| CHST2 | GLRX | MAP3K1 | PPP1R3C | TPST2 |
| CHST3 | GPC1 | MIF | PRDX5 | UGP2 |
| CITED2 | GPC3 | MT1E | PRKCA | VEGFA |
| COL5A1 | GPC4 | MT2A | PRKCDBP | VHL |
| CP | GPI | MXI1 | PTRF | VLDLR |
| CSRP2 | GRHPR | MYH9 | PYGM | WISP2 |
| CTGF | GYS1 | NAGK | RBPJ | WSB1 |
| CXCR4 | HAS1 | NCAN | RORA | XPNPEP1 |
| ACKR3 | HDLBP | NDRG1 | RRAGD | ZFP36 |
| CYR61 | HEXA | NDST1 | S100A4 | ZNF292 |

**Table S3 The detailed gene list of 13 metabolic pathway gene sets.**

| REACTOME_GLYCOLYSIS | GO_NEGATIVE_REGULATION_OF_GLYCOLYTIC_PROCESS | GO_POSITIVE_REGULATION_OF_GLYCOLYTIC_PROCESS | HALLMARK_GLYCOLYSIS | HALLMARK_OXIDATIVE_PHOSPHORYLATION | GO_REGULATION_OF_OXIDATIVE_PHOSPHORYLATION | GO_OXIDATIVE_PHOSPHORYLATION | KEGG_OXIDATIVE_PHOSPHORYLATION | KEGG_CITRATE_CYCLE_TCA_CYCLE | REACTOME_CITRIC_ACID_CYCLE_TCA_CYCLE | REACTOME_PYRUVATE_METABOLISM_AND_CITRIC_ACID_TCA_CYCLE | GO_TRICARBOXYLIC_ACID_CYCLE | GO_TRICARBOXYLIC_ACID_CYCLE_ENZYME_COMPLEX |
| --- | --- | --- | --- | --- | --- | --- | --- | --- | --- | --- | --- | --- |
| NUP160 | PPARGC1A | PRXL2C | PGK1 | NDUFS3 | PPIF | UQCRFS1P1 | ATP6V1G1 | IDH3B | CS | PDK4 | ME3 | BCKDK |
| TPR | FBP1 | ESRRB | ALDOA | UQCRB | PARK7 | NDUFC2-KCTD14 | UQCR10 | DLST | IDH3G | PDK2 | MDH1B | DBT |
| NDC1 | NUPR1 | ZBTB20 | ENO1 | NDUFS2 | ATPSCKMT | PPIF | NDUFA5 | PCK2 | SDHA | MPC1 | CS | DLD |
| PFKP | MYOG | GAPDHS | TPI1 | SDHA | COX7A1 | ATP5PD | NDUFA4 | CS | ME2 | CS | DLAT | DLST |
| PKM | SIRT6 | GPD1 | PFKP | UQCRC1 | COX7A2 | ATP5MG | COX6CP3 | PDHB | DLD | HAGH | DLD | FH |
| NUP133 | PFKFB1 | HIF1A | ERO1A | NDUFA9 | COX7A2P2 | MTCO2P12 | PPA2 | PCK1 | FH | ME1 | DLST | KAT2A |
| PRKACA | DDIT4 | HTR2A | ALDOB | NDUFS4 | PDE12 | UQCR11 | ATP5MF | PDHA1 | ACO2 | IDH3G | FH | OGDH |
| ENO1 | PPARA | IFNG | VEGFA | NDUFS1 | AK4 | PARK7 | NDUFS7 | PDHA2 | IDH3B | PDK3 | NNT | OGDHL |
| NUP37 | TIGAR | IGF1 | MXI1 | NDUFA2 | ABCD1 | COX6B2 | MT-CYB | SUCLG2P2 | OGDH | SDHA | IDH1 | BCKDHA |
| GCKR | SLC4A1 | APP | PKM | NDUFS8 | ISCU | NDUFA11 | ATP6V0A1 | FH | NNT | ME2 | IDH2 | BCKDHB |
| SEH1L | STAT3 | INS | HK2 | SDHB | NUPR1 | COX4I1 | ATP6V1G2 | SDHD | SDHB | L2HGDH | IDH3A | SUCLG2 |
| NUP50 | CBFA2T3 | INSR | LDHA | NNT | DNAJC15 | COX5B | ATP6V0B | OGDH | DLST | PDPR | IDH3B | SUCLG1 |
| AAAS | ACTN3 | ARNT | EXT1 | ATP5PO | RHOA | COX6A1 | ATP5PO | SDHB | SUCLA2 | DLD | IDH3G | SUCLA2 |
| NUP188 | NCOR1 | P2RX7 | SLC25A10 | ATP5MC3 | MIR210 | COX6A2 | MT-CO2 | IDH3A | SDHC | FH | IREB2 | MRPS36 |
| RAE1 | HDAC4 | MLXIPL | GUSB | NDUFS7 | MYOG | COX6B1 | MT-CO1 | SDHC | MDH2 | SLC16A8 | MDH1 |  |
| PGK1 |  | PFKFB1 | PFKFB1 | ATP5F1A | MLXIPL | ATPSCKMT | ATP6AP1 | IDH2 | ME3 | ACO2 | MDH2 |  |
| NUP93 |  | PFKFB2 | PGAM1 | NDUFV1 | PGK1 | COX6C | COX8C | IDH1 | SUCLG1 | GSTZ1 | ME2 |  |
| PPP2CB |  | PFKFB3 | PYGB | COX5B | PGK2 | COX7A1 | MT-CO3 | ACO1 | IDH3A | IDH3B | ACO1 |  |
| GPI |  | PFKFB4 | AK4 | UQCRH | ATP7A | COX7A2 | COX5B | ACLY | SUCLG2 | OGDH | OGDH |  |
| PPP2R1A |  | PRKAA1 | P4HA1 | NDUFA1 | SHMT2 | COX7A2P2 | COX4I1 | MDH2 | FAHD1 | PDHX | ACO2 |  |
| GAPDHS |  | PRKAA2 | PMM2 | ATP5F1C | PINK1 | COX7B | ATP6V0A4 | DLD | IDH2 | LDHB | PDHA1 |  |
| GCK |  | PSEN1 | FAM162A | ATP5F1B | ANTKMT | COX7C | ATP12A | MDH1 | SDHD | PPARD | PDHA2 |  |
| ENO3 |  | SLC4A4 | SDC1 | COX7B | SNCA | COX8A | MT-ATP6 | DLAT |  | NNT | PDHB |  |
| NUP88 |  | ENTPD5 | EGLN3 | SDHD | VCP | COX10 | MT-ATP8 | OGDHL |  | SDHB | SDHAF2 |  |
| ALDOC |  |  | PC | CYCS | SLC25A23 | COX15 | ATP5PD | PC |  | DLST | DHTKD1 |  |
| NUP98 |  |  | B4GALT7 | NDUFA6 | SLC25A33 | CYC1 | NDUFA2 | SDHA |  | GLO1 | OGDHL |  |
| NUP107 |  |  | FBP2 | NDUFAB1 | DNAJC30 | COX7B2 | NDUFA3 | SUCLG1 |  | PDHA1 | SDHA |  |
| GAPDH |  |  | IGFBP3 | COX8A | UQCC2 | DLD | NDUFA1 | SUCLA2 |  | LDHA | SDHB |  |
| TPI1 |  |  | CHPF | ACO2 | ACTN3 | PDE12 | ATP5MC1P5 | SUCLG2 |  | SUCLA2 | SDHC |  |
| ENO2 |  |  | B3GAT3 | ATP5MC1 | CCNB1 | AK4 | UQCRQ | IDH3G |  | SLC16A3 | SDHD |  |
| PPP2R5D |  |  | CHST12 | CYC1 | COX7A2L | ABCD1 | ATP6V1H | ACO2 |  | MPC2 | FAHD1 |  |
| GNPDA1 |  |  | HS2ST1 | NDUFB6 | CDK1 | ISCU | ATP5F1D |  |  | SDHC | SUCLG2 |  |
| NUP155 |  |  | MPI | ATP5F1E |  | FXN | LHPP |  |  | MDH2 | SUCLG1 |  |
| PPP2CA |  |  | GNPDA1 | COX5A |  | AFG1L | ATP6V0D2 | |  | ADHFE1 | SUCLA2 |  |
| PFKFB4 |  |  | AKR1A1 | UQCRC2 |  | NIPSNAP2 | COX7A2L |  |  | DLAT |  |  |
| NUP43 |  |  | PPFIA4 | COX6A1 |  | NUPR1 | ATP5MC3 |  |  | ME3 |  |  |
| PFKFB2 |  |  | B3GAT1 | ATP5F1D |  | UQCRQ | ATP6V1C2 | |  | PDK1 |  |  |
| NUP153 |  |  | CHPF2 | COX6C |  | DMAC2L | ATP5MC2 |  |  | SLC16A1 |  |  |
| NUP85 |  |  | G6PD | ATP5PF |  | DNAJC15 | ATP5MC1 |  |  | PDHA2 |  |  |
| NUP214 |  |  | MDH2 | NDUFB3 |  | UQCR10 | ATP5PB |  |  | SUCLG1 |  |  |
| NUP210 |  |  | CHST6 | IDH3B |  | STOML2 | ATP5F1E |  |  | PDP1 |  |  |
| NUP42 |  |  | PGLS | OGDH |  | COX8C | COX5A |  |  | IDH3A |  |  |
| ALDOB |  |  | PGAM2 | NDUFB8 |  | NDUFS7 | TCIRG1 |  |  | LDHC |  |  |
| PPP2R1B |  |  | CHST1 | SURF1 |  | RHOA | UQCRHL |  |  | LDHAL6A |  |  |
| NUP54 |  |  | GPC1 | COX6B1 |  | COA6 | ATP4B |  |  | PDHB |  |  |
| NUP58 |  |  | TSTA3 | NDUFB5 |  | CHCHD10 | ATP6V1D |  |  | LDHAL6B |  |  |
| PFKL |  |  | ALG1 | NDUFA4 |  | MIR210 | ATP4A |  |  | BSG |  |  |
| PRKACB |  |  | GFPT1 | NDUFB1 |  | MECP2 | ATP5F1A |  |  | SUCLG2 |  |  |
| PKLR |  |  | PRPS1 | COX4I1 |  | UQCRHL | ATP5PF |  |  | PDP2 |  |  |
| ALDOA |  |  | GOT1 | COX7C |  | MSH2 | ATP5ME |  |  | FAHD1 |  |  |
| PFKM |  |  | MDH1 | UQCRFS1 |  | MT-ATP6 | SDHA |  |  | D2HGDH |  |  |
| RANBP2 |  |  | SLC35A3 | SDHC |  | MT-ATP8 | ATP6V1A |  |  | IDH2 |  |  |
| NUP205 |  |  | GALK1 | ATP6V1F |  | MT-CO1 | ATP6V1E1 |  |  | RXRA |  |  |
| HK1 |  |  | EGFR | COX7A2 |  | MT-CO2 | NDUFA4L2 | |  | SDHD |  |  |
| SEC13 |  |  | ANGPTL4 | SUCLG1 |  | MT-CO3 | ATP6V1B2 |  |  | VDAC1 |  |  |
| PFKFB1 |  |  | CITED2 | NDUFS6 |  | MT-CYB | ATP6V1B1 |  |  |  |  |  |
| ADPGK |  |  | PLOD2 | NDUFA7 |  | MT-ND1 | ATP6V1C1 | |  |  |  |  |
| HK2 |  |  | QSOX1 | FH |  | MT-ND2 | ATP6V0C |  |  |  |  |  |
| HK3 |  |  | ME2 | NDUFV2 |  | MT-ND3 | COX10 |  |  |  |  |  |
| NUP35 |  |  | SPAG4 | OXA1L |  | MT-ND4 | COX8A |  |  |  |  |  |
| GNPDA2 |  |  | P4HA2 | NDUFC1 |  | MT-ND4L | COX7C |  |  |  |  |  |
| PGAM2 |  |  | GAPDHS | UQCR11 |  | MT-ND5 | SDHD |  |  |  |  |  |
| PRKACG |  |  | ENO2 | NDUFA5 |  | MT-ND6 | SDHB |  |  |  |  |  |
| PGM2L1 |  |  | GOT2 | CS |  | MYOG | SDHC |  |  |  |  |  |
| PFKFB3 |  |  | EXT2 | ATP6V1G1 | | NDUFA1 | ATP6V1G3 | |  |  |  |  |
| PGK2 |  |  | SLC25A13 | ATP5PB |  | NDUFA2 | COX7B |  |  |  |  |  |
| PGAM1 |  |  | HMMR | HCCS |  | NDUFA3 | COX7A1 |  |  |  |  |  |
| BPGM |  |  | PDK3 | HADHB |  | NDUFA4 | COX7A2 |  |  |  |  |  |
| PGP |  |  | CXCR4 | ATP5PD |  | NDUFA5 | COX6C |  |  |  |  |  |
| POM121 |  |  | GPC4 | PDHA1 |  | NDUFA6 | COX17 |  |  |  |  |  |
| NUP62 |  |  | ECD | NDUFA8 |  | NDUFA7 | ATP5F1B |  |  |  |  |  |
| POM121C |  |  | GNE | DLD |  | NDUFA8 | COX6B1 |  |  |  |  |  |
|  |  |  | B4GALT2 | OPA1 |  | NDUFA9 | ATP5F1C |  |  |  |  |  |
|  |  |  | FUT8 | SLC25A11 |  | NDUFA10 | AL929410.2 | |  |  |  |  |
|  |  |  | MIOX | ATP5ME |  | NDUFAB1 | ATP6V1F |  |  |  |  |  |
|  |  |  | VCAN | PDHB |  | NDUFB1 | COX7B2 |  |  |  |  |  |
|  |  |  | GPC3 | ATP5MF |  | NDUFB2 | ATP6V0A2 | |  |  |  |  |
|  |  |  | B3GALT6 | NDUFB7 |  | NDUFB3 | UQCRB |  |  |  |  |  |
|  |  |  | HSPA5 | IDH2 |  | NDUFB4 | ATP6V0D1 | |  |  |  |  |
|  |  |  | ME1 | MTX2 |  | NDUFB5 | COX6A2 |  |  |  |  |  |
|  |  |  | ADORA2B | VDAC3 |  | NDUFB6 | COX6A1 |  |  |  |  |  |
|  |  |  | UGP2 | MDH1 |  | NDUFB7 | UQCRH |  |  |  |  |  |
|  |  |  | MIF | ATP5MC2 |  | NDUFB8 | UQCRFS1 |  |  |  |  |  |
|  |  |  | NANP | IMMT |  | NDUFB9 | UQCRC2 |  |  |  |  |  |
|  |  |  | ZNF292 | MDH2 |  | NDUFB10 | UQCRC1 |  |  |  |  |  |
|  |  |  | STC2 | SLC25A3 |  | NDUFC1 | NDUFB3 |  |  |  |  |  |
|  |  |  | TPST1 | ATP6V1D |  | NDUFC2 | NDUFB1 |  |  |  |  |  |
|  |  |  | PGM2 | VDAC2 |  | NDUFS1 | NDUFB2 |  |  |  |  |  |
|  |  |  | GYS1 | ACADM |  | NDUFS2 | NDUFA10 |  |  |  |  |  |
|  |  |  | TKTL1 | COX7A2L |  | NDUFS3 | NDUFAB1 |  |  |  |  |  |
|  |  |  | TGFA | TIMM17A |  | NDUFV1 | ATP6V0E2 |  |  |  |  |  |
|  |  |  | CHST2 | ATP6V1E1 |  | NDUFS4 | MT-ND6 |  |  |  |  |  |
|  |  |  | PHKA2 | NDUFA3 |  | NDUFS5 | NDUFA9 |  |  |  |  |  |
|  |  |  | STMN1 | SLC25A6 |  | NDUFS6 | MT-ND5 |  |  |  |  |  |
|  |  |  | GALE | IDH3G |  | NDUFS8 | NDUFA7 |  |  |  |  |  |
|  |  |  | MET | ACADVL |  | NDUFV2 | NDUFA8 |  |  |  |  |  |
|  |  |  | LCT | ETFA |  | NDUFV3 | UQCR11 |  |  |  |  |  |
|  |  |  | IRS2 | TIMM9 |  | ATP5F1A | NDUFA6 |  |  |  |  |  |
|  |  |  | POLR3K | IDH3A |  | ATP5F1B | NDUFV3 |  |  |  |  |  |
|  |  |  | B4GALT1 | TIMM8B |  | ATP5F1C | ATP6V1E2 |  |  |  |  |  |
|  |  |  | EFNA3 | ATP6AP1 |  | NDUFA13 | MT-ND4 |  |  |  |  |  |
|  |  |  | LHX9 | TIMM13 |  | MLXIPL | MT-ND4L |  |  |  |  |  |
|  |  |  | KDELR3 | UQCRQ |  | NDUFAF1 | MT-ND2 |  |  |  |  |  |
|  |  |  | TALDO1 | ABCB7 |  | ATP5F1D | COX6B2 |  |  |  |  |  |
|  |  |  | DPYSL4 | VDAC1 |  | ATP5F1E | PPA1 |  |  |  |  |  |
|  |  |  | VLDLR | ATP5MG |  | ATP5PB | MT-ND3 |  |  |  |  |  |
|  |  |  | CD44 | PHB2 |  | ATP5MC1 | COX4I2 |  |  |  |  |  |
|  |  |  | AGL | DECR1 |  | ATP5MC2 | MT-ND1 |  |  |  |  |  |
|  |  |  | SOX9 | SUCLA2 |  | ATP5MC3 | ATP5MG |  |  |  |  |  |
|  |  |  | DDIT4 | GOT2 |  | ATP5ME | ATP6V0E1 |  |  |  |  |  |
|  |  |  | IDUA | DLAT |  | ATP5PF | NDUFA11 |  |  |  |  |  |
|  |  |  | CASP6 | ATP6V1H |  | PGK1 | NDUFB10 |  |  |  |  |  |
|  |  |  | GLCE | NDUFB2 |  | PGK2 | NDUFS8 |  |  |  |  |  |
|  |  |  | COPB2 | FDX1 |  | ATP7A | NDUFC1 |  |  |  |  |  |
|  |  |  | DSC2 | HADHA |  | ATP5PO | NDUFC2 |  |  |  |  |  |
|  |  |  | HS6ST2 | ATP6V1C1 | | CYCS | NDUFS1 |  |  |  |  |  |
|  |  |  | CDK1 | MAOB |  | NDUFB11 | NDUFV2 |  |  |  |  |  |
|  |  |  | PLOD1 | NDUFB4 |  | SDHAF2 | NDUFB6 |  |  |  |  |  |
|  |  |  | SDC2 | UQCR10 |  | NDUFA12 | NDUFS4 |  |  |  |  |  |
|  |  |  | GMPPB | ETFDH |  | COQ9 | NDUFB7 |  |  |  |  |  |
|  |  |  | PAXIP1 | GPX4 |  | BID | NDUFV1 |  |  |  |  |  |
|  |  |  | NSDHL | PDHX |  | SDHA | NDUFB8 |  |  |  |  |  |
|  |  |  | RARS1 | MFN2 |  | SDHC | NDUFS6 |  |  |  |  |  |
|  |  |  | SLC16A3 | AIFM1 |  | SDHD | NDUFB9 |  |  |  |  |  |
|  |  |  | GLRX | ACAA2 |  | SHMT2 | NDUFS5 |  |  |  |  |  |
|  |  |  | SRD5A3 | ETFB |  | PINK1 | NDUFS2 |  |  |  |  |  |
|  |  |  | SDC3 | COX11 |  | ANTKMT | NDUFB4 |  |  |  |  |  |
|  |  |  | HDLBP | ECHS1 |  | SNCA | NDUFS3 |  |  |  |  |  |
|  |  |  | COL5A1 | PMPCA |  | SURF1 | NDUFB5 |  |  |  |  |  |
|  |  |  | CLDN9 | ATP6V0B |  | TAZ | CYC1 |  |  |  |  |  |
|  |  |  | TFF3 | SLC25A5 |  | UQCRB | COX11 |  |  |  |  |  |
|  |  |  | STC1 | DLST |  | UQCRC1 | COX15 |  |  |  |  |  |
|  |  |  | KIF20A | COX15 |  | UQCRC2 |  |  |  |  |  |  |
|  |  |  | GYS2 | CYB5A |  | UQCRFS1 |  |  |  |  |  |  |
|  |  |  | SLC37A4 | ALAS1 |  | UQCRH |  |  |  |  |  |  |
|  |  |  | LHPP | SLC25A4 |  | VCP |  |  |  |  |  |  |
|  |  |  | SDHC | CPT1A |  | SLC25A23 |  |  |  |  |  |  |
|  |  |  | NASP | SLC25A20 |  | UQCC3 |  |  |  |  |  |  |
|  |  |  | AURKA | MTRR |  | TEFM |  |  |  |  |  |  |
|  |  |  | B3GNT3 | COX17 |  | SLC25A33 |  |  |  |  |  |  |
|  |  |  | ISG20 | CYB5R3 |  | DNAJC30 |  |  |  |  |  |  |
|  |  |  | LDHC | TOMM22 |  | UQCC2 |  |  |  |  |  |  |
|  |  |  | ARPP19 | ACAT1 |  | COX4I2 |  |  |  |  |  |  |
|  |  |  | CENPA | MRPS11 |  | ACTN3 |  |  |  |  |  |  |
|  |  |  | HOMER1 | ATP6V0C |  | CCNB1 |  |  |  |  |  |  |
|  |  |  | BIK | PDK4 |  | COX7A2L |  |  |  |  |  |  |
|  |  |  | CYB5A | TIMM10 |  | COX5A |  |  |  |  |  |  |
|  |  |  | HAX1 | LDHA |  | ATP5MF |  |  |  |  |  |  |
|  |  |  | COG2 | ECI1 |  | CDK1 |  |  |  |  |  |  |
|  |  |  | IL13RA1 | MRPL11 |  |  |  |  |  |  |  |  |
|  |  |  | NOL3 | FXN |  |  |  |  |  |  |  |  |
|  |  |  | CLDN3 | MRPS12 |  |  |  |  |  |  |  |  |
|  |  |  | AGRN | COX10 |  |  |  |  |  |  |  |  |
|  |  |  | CLN6 | RHOT1 |  |  |  |  |  |  |  |  |
|  |  |  | TXN | ACAA1 |  |  |  |  |  |  |  |  |
|  |  |  | PAM | ACADSB |  |  |  |  |  |  |  |  |
|  |  |  | CAPN5 | LDHB |  |  |  |  |  |  |  |  |
|  |  |  | PKP2 | MRPS30 |  |  |  |  |  |  |  |  |
|  |  |  | ABCB6 | ATP1B1 |  |  |  |  |  |  |  |  |
|  |  |  | DCN | BDH2 |  |  |  |  |  |  |  |  |
|  |  |  | GMPPA | SLC25A12 |  |  |  |  |  |  |  |  |
|  |  |  | BPNT1 | TIMM50 |  |  |  |  |  |  |  |  |
|  |  |  | ANG | MRPL34 |  |  |  |  |  |  |  |  |
|  |  |  | GPR87 | ISCA1 |  |  |  |  |  |  |  |  |
|  |  |  | GAL3ST1 | MRPL35 |  |  |  |  |  |  |  |  |
|  |  |  | ALDH7A1 | IDH1 |  |  |  |  |  |  |  |  |
|  |  |  | NT5E | HSPA9 |  |  |  |  |  |  |  |  |
|  |  |  | IDH1 | MRPL15 |  |  |  |  |  |  |  |  |
|  |  |  | PYGL | MRPS15 |  |  |  |  |  |  |  |  |
|  |  |  | NDUFV3 | TOMM70 |  |  |  |  |  |  |  |  |
|  |  |  | NDST3 | TCIRG1 |  |  |  |  |  |  |  |  |
|  |  |  | PPP2CB | ISCU |  |  |  |  |  |  |  |  |
|  |  |  | PSMC4 | POLR2F |  |  |  |  |  |  |  |  |
|  |  |  | TPBG | NQO2 |  |  |  |  |  |  |  |  |
|  |  |  | TGFBI | NDUFC2 |  |  |  |  |  |  |  |  |
|  |  |  | GALK2 | MRPS22 |  |  |  |  |  |  |  |  |
|  |  |  | CTH | POR |  |  |  |  |  |  |  |  |
|  |  |  | KIF2A | ATP6V0E1 |  |  |  |  |  |  |  |  |
|  |  |  | CACNA1H | PHYH |  |  |  |  |  |  |  |  |
|  |  |  | ANKZF1 | MPC1 |  |  |  |  |  |  |  |  |
|  |  |  | SAP30 | GPI |  |  |  |  |  |  |  |  |
|  |  |  | RBCK1 | AFG3L2 |  |  |  |  |  |  |  |  |
|  |  |  | ELF3 | HSD17B10 | |  |  |  |  |  |  |  |
|  |  |  | RPE | CASP7 |  |  |  |  |  |  |  |  |
|  |  |  | B4GALT4 | PRDX3 |  |  |  |  |  |  |  |  |
|  |  |  | DEPDC1 | MGST3 |  |  |  |  |  |  |  |  |
|  |  |  | RRAGD | HTRA2 |  |  |  |  |  |  |  |  |
|  |  |  | IER3 | BCKDHA |  |  |  |  |  |  |  |  |
|  |  |  | ALDH9A1 | LRPPRC |  |  |  |  |  |  |  |  |
|  |  |  | DLD | RETSAT |  |  |  |  |  |  |  |  |
|  |  |  | MERTK | ECH1 |  |  |  |  |  |  |  |  |
|  |  |  | GCLC | RHOT2 |  |  |  |  |  |  |  |  |
|  |  |  | FKBP4 | BAX |  |  |  |  |  |  |  |  |
|  |  |  | SOD1 | MTRF1 |  |  |  |  |  |  |  |  |
|  |  |  | MED24 | GLUD1 |  |  |  |  |  |  |  |  |
|  |  |  | AK3 | SUPV3L1 |  |  |  |  |  |  |  |  |
|  |  |  | XYLT2 | GRPEL1 |  |  |  |  |  |  |  |  |
|  |  |  | ARTN | PDP1 |  |  |  |  |  |  |  |  |
|  |  |  | PPIA | ALDH6A1 |  |  |  |  |  |  |  |  |
|  |  |  | CHST4 | OAT |  |  |  |  |  |  |  |  |

**Table S4 The detailed gene list of 13 immune function pathway gene sets.**

| APC_co_inhibition | APC_co_stimulation | CCR | Check-point | Cytolytic_activity | HLA | Inflammation-promoting | MHC_class_I | Parainflammation | T_cell_co-inhibition | T_cell_co-stimulation | Type_I_IFN_Reponse | Type_II_IFN_Reponse |
| --- | --- | --- | --- | --- | --- | --- | --- | --- | --- | --- | --- | --- |
| C10orf54 | CD40 | CCL16 | IDO1 | PRF1 | HLA-E | CCL5 | B2M | CXCL10 | BTLA | CD2 | DDX4 | GPR146 |
| CD274 | CD58 | TPO | LAG3 | GZMA | HLA-DPB2 | CD19 | HLA-A | PLAT | C10orf54 | CD226 | IFIT1 | SELP |
| LGALS9 | CD70 | TGFBR2 | CTLA4 |  | HLA-C | CD8B | TAP1 | CCND1 | CD160 | CD27 | IFIT2 | AHR |
| PDCD1LG2 | ICOSLG | CXCL2 | TNFRSF9 |  | HLA-J | CXCL10 |  | LGMN | CD244 | CD28 | IFIT3 |  |
| PVRL3 | SLAMF1 | CCL14 | ICOS |  | HLA-DQB1 | CXCL13 |  | PLAUR | CD274 | CD40LG | IRF7 |  |
|  | TNFSF14 | TGFBR3 | CD80 |  | HLA-DQB2 | CXCL9 |  | AIM2 | CTLA4 | ICOS | ISG20 |  |
|  | TNFSF15 | IL11RA | PDCD1LG2 | | HLA-DQA2 | GNLY |  | MMP7 | HAVCR2 | SLAMF1 | MX1 |  |
|  | TNFSF18 | CCL11 | TIGIT |  | HLA-DQA1 | GZMB |  | ICAM1 | LAG3 | TNFRSF18 | MX2 |  |
|  | TNFSF4 | IL4I1 | CD70 |  | HLA-A | IFNG |  | MX2 | LAIR1 | TNFRSF25 | RSAD2 |  |
|  | TNFSF8 | IL33 | TNFSF9 |  | HLA-DMA | IL12A |  | CXCL9 | TIGIT | TNFRSF4 | TNFSF10 |  |
|  | TNFSF9 | CXCL12 | ICOSLG |  | HLA-DOB | IL12B |  | ANXA1 |  | TNFRSF8 |  |  |
|  |  | CXCL10 | KIR3DL1 |  | HLA-DRB1 | IRF1 |  | TLR2 |  | TNFRSF9 |  |  |
|  |  | BMPER | CD86 |  | HLA-H | PRF1 |  | PLA2G2D |  | TNFSF14 |  |  |
|  |  | BMP8A | PDCD1 |  | HLA-B | STAT1 |  | ITGA2 |  |  |  |  |
|  |  | CXCL11 | LAIR1 |  | HLA-DRB5 | TBX21 |  | MX1 |  |  |  |  |
|  |  | IL21R | TNFRSF8 |  | HLA-DOA |  |  | HMOX1 |  |  |  |  |
|  |  | IL17B | TNFSF15 |  | HLA-DPB1 | |  | CD276 |  |  |  |  |
|  |  | TNFRSF9 | TNFRSF14 |  | HLA-DRA |  |  | TIRAP |  |  |  |  |
|  |  | ILF2 | IDO2 |  | HLA-DRB6 | |  | IL33 |  |  |  |  |
|  |  | CX3CR1 | CD276 |  | HLA-L |  |  | PTGES |  |  |  |  |
|  |  | CCR8 | CD40 |  | HLA-F |  |  | TNFRSF12A | |  |  |  |
|  |  | TNFSF12 | TNFRSF4 |  | HLA-G |  |  | SCARB1 |  |  |  |  |
|  |  | CSF3 | TNFSF14 |  | HLA-DMB |  |  | CD14 |  |  |  |  |
|  |  | TNFSF4 | HHLA2 |  | HLA-DPA1 | |  | BLNK |  |  |  |  |
|  |  | BMP3 | CD244 |  |  |  |  | IFIT3 |  |  |  |  |
|  |  | CX3CL1 | CD274 |  |  |  |  | RETNLB |  |  |  |  |
|  |  | BMP5 | HAVCR2 |  |  |  |  | IFIT2 |  |  |  |  |
|  |  | CXCR2 | CD27 |  |  |  |  | ISG15 |  |  |  |  |
|  |  | TNFRSF10D | BTLA |  |  |  |  | OAS2 |  |  |  |  |
|  |  | BMP2 | LGALS9 |  |  |  |  | REL |  |  |  |  |
|  |  | CXCL14 | TMIGD2 |  |  |  |  | OAS3 |  |  |  |  |
|  |  | CCL28 | CD28 |  |  |  |  | CD44 |  |  |  |  |
|  |  | CXCL3 | CD48 |  |  |  |  | PPARG |  |  |  |  |
|  |  | BMP6 | TNFRSF25 |  |  |  |  | BST2 |  |  |  |  |
|  |  | CCL21 | CD40LG |  |  |  |  | OAS1 |  |  |  |  |
|  |  | CXCL9 | ADORA2A | |  |  |  | NOX1 |  |  |  |  |
|  |  | CCL23 | VTCN1 |  |  |  |  | PLA2G2A |  |  |  |  |
|  |  | IL6 | CD160 |  |  |  |  | IFIT1 |  |  |  |  |
|  |  | TNFRSF18 | CD44 |  |  |  |  | IFITM3 |  |  |  |  |
|  |  | IL17RD | TNFSF18 |  |  |  |  | IL1RN |  |  |  |  |
|  |  | IL17D | TNFRSF18 |  |  |  |  |  |  |  |  |  |
|  |  | IL27 | BTNL2 |  |  |  |  |  |  |  |  |  |
|  |  | CCL7 | C10orf54 |  |  |  |  |  |  |  |  |  |
|  |  | IL1R1 | CD200R1 |  |  |  |  |  |  |  |  |  |
|  |  | CXCR4 | TNFSF4 |  |  |  |  |  |  |  |  |  |
|  |  | CXCR2P1 | CD200 |  |  |  |  |  |  |  |  |  |
|  |  | TGFB1I1 | NRP1 |  |  |  |  |  |  |  |  |  |
|  |  | IFNGR1 |  |  |  |  |  |  |  |  |  |  |
|  |  | IL9R |  |  |  |  |  |  |  |  |  |  |
|  |  | IL1RAPL1 |  |  |  |  |  |  |  |  |  |  |
|  |  | IL11 |  |  |  |  |  |  |  |  |  |  |
|  |  | CSF1 |  |  |  |  |  |  |  |  |  |  |
|  |  | IL20RA |  |  |  |  |  |  |  |  |  |  |
|  |  | IL25 |  |  |  |  |  |  |  |  |  |  |
|  |  | TNFRSF4 |  |  |  |  |  |  |  |  |  |  |
|  |  | IL18 |  |  |  |  |  |  |  |  |  |  |
|  |  | ILF3 |  |  |  |  |  |  |  |  |  |  |
|  |  | CCL20 |  |  |  |  |  |  |  |  |  |  |
|  |  | TNFRSF12A | |  |  |  |  |  |  |  |  |  |
|  |  | IL6ST |  |  |  |  |  |  |  |  |  |  |
|  |  | CXCL13 |  |  |  |  |  |  |  |  |  |  |
|  |  | IL12B |  |  |  |  |  |  |  |  |  |  |
|  |  | TNFRSF8 |  |  |  |  |  |  |  |  |  |  |
|  |  | IL6R |  |  |  |  |  |  |  |  |  |  |
|  |  | BMPR2 |  |  |  |  |  |  |  |  |  |  |
|  |  | IFNE |  |  |  |  |  |  |  |  |  |  |
|  |  | IL1RAPL2 |  |  |  |  |  |  |  |  |  |  |
|  |  | IL3RA |  |  |  |  |  |  |  |  |  |  |
|  |  | BMP4 |  |  |  |  |  |  |  |  |  |  |
|  |  | CCL24 |  |  |  |  |  |  |  |  |  |  |
|  |  | TNFSF13B |  |  |  |  |  |  |  |  |  |  |
|  |  | CCR4 |  |  |  |  |  |  |  |  |  |  |
|  |  | IL2RA |  |  |  |  |  |  |  |  |  |  |
|  |  | IL32 |  |  |  |  |  |  |  |  |  |  |
|  |  | TNFRSF10C | |  |  |  |  |  |  |  |  |  |
|  |  | IL22RA1 |  |  |  |  |  |  |  |  |  |  |
|  |  | BMPR1A |  |  |  |  |  |  |  |  |  |  |
|  |  | CXCR5 |  |  |  |  |  |  |  |  |  |  |
|  |  | CXCR3 |  |  |  |  |  |  |  |  |  |  |
|  |  | IFNA8 |  |  |  |  |  |  |  |  |  |  |
|  |  | IL17REL |  |  |  |  |  |  |  |  |  |  |
|  |  | IFNB1 |  |  |  |  |  |  |  |  |  |  |
|  |  | IFNAR1 |  |  |  |  |  |  |  |  |  |  |
|  |  | TNFRSF1B |  |  |  |  |  |  |  |  |  |  |
|  |  | CCL17 |  |  |  |  |  |  |  |  |  |  |
|  |  | IFNL1 |  |  |  |  |  |  |  |  |  |  |
|  |  | IL16 |  |  |  |  |  |  |  |  |  |  |
|  |  | IL1RL1 |  |  |  |  |  |  |  |  |  |  |
|  |  | ILK |  |  |  |  |  |  |  |  |  |  |
|  |  | CCL25 |  |  |  |  |  |  |  |  |  |  |
|  |  | ILDR2 |  |  |  |  |  |  |  |  |  |  |
|  |  | CXCR1 |  |  |  |  |  |  |  |  |  |  |
|  |  | IL36RN |  |  |  |  |  |  |  |  |  |  |
|  |  | IL34 |  |  |  |  |  |  |  |  |  |  |
|  |  | TGFB1 |  |  |  |  |  |  |  |  |  |  |
|  |  | IFNG |  |  |  |  |  |  |  |  |  |  |
|  |  | IL19 |  |  |  |  |  |  |  |  |  |  |
|  |  | ILKAP |  |  |  |  |  |  |  |  |  |  |
|  |  | BMP2K |  |  |  |  |  |  |  |  |  |  |
|  |  | CCR10 |  |  |  |  |  |  |  |  |  |  |
|  |  | ILDR1 |  |  |  |  |  |  |  |  |  |  |
|  |  | EPO |  |  |  |  |  |  |  |  |  |  |
|  |  | CCR7 |  |  |  |  |  |  |  |  |  |  |
|  |  | IL17C |  |  |  |  |  |  |  |  |  |  |
|  |  | IL23A |  |  |  |  |  |  |  |  |  |  |
|  |  | CCR5 |  |  |  |  |  |  |  |  |  |  |
|  |  | IL7 |  |  |  |  |  |  |  |  |  |  |
|  |  | EPOR |  |  |  |  |  |  |  |  |  |  |
|  |  | CCL13 |  |  |  |  |  |  |  |  |  |  |
|  |  | IL2RG |  |  |  |  |  |  |  |  |  |  |
|  |  | IL31RA |  |  |  |  |  |  |  |  |  |  |
|  |  | TNFAIP6 |  |  |  |  |  |  |  |  |  |  |
|  |  | IFNL2 |  |  |  |  |  |  |  |  |  |  |
|  |  | BMP1 |  |  |  |  |  |  |  |  |  |  |
|  |  | IL12RB1 |  |  |  |  |  |  |  |  |  |  |
|  |  | TNFAIP8 |  |  |  |  |  |  |  |  |  |  |
|  |  | IL4R |  |  |  |  |  |  |  |  |  |  |
|  |  | TNFRSF6B |  |  |  |  |  |  |  |  |  |  |
|  |  | TNFAIP8L1 | |  |  |  |  |  |  |  |  |  |
|  |  | TNFRSF10B | |  |  |  |  |  |  |  |  |  |
|  |  | IFNL3 |  |  |  |  |  |  |  |  |  |  |
|  |  | CCL5 |  |  |  |  |  |  |  |  |  |  |
|  |  | CXCL6 |  |  |  |  |  |  |  |  |  |  |
|  |  | CXCL1 |  |  |  |  |  |  |  |  |  |  |
|  |  | CCR3 |  |  |  |  |  |  |  |  |  |  |
|  |  | TNFSF11 |  |  |  |  |  |  |  |  |  |  |
|  |  | CSF1R |  |  |  |  |  |  |  |  |  |  |
|  |  | IL21 |  |  |  |  |  |  |  |  |  |  |
|  |  | IL1RAP |  |  |  |  |  |  |  |  |  |  |
|  |  | IL12RB2 |  |  |  |  |  |  |  |  |  |  |
|  |  | CCL1 |  |  |  |  |  |  |  |  |  |  |
|  |  | IL17RA |  |  |  |  |  |  |  |  |  |  |
|  |  | CCR1 |  |  |  |  |  |  |  |  |  |  |
|  |  | IL1RN |  |  |  |  |  |  |  |  |  |  |
|  |  | TNFRSF11B | |  |  |  |  |  |  |  |  |  |
|  |  | TNFRSF14 |  |  |  |  |  |  |  |  |  |  |
|  |  | IL13 |  |  |  |  |  |  |  |  |  |  |
|  |  | IL2RB |  |  |  |  |  |  |  |  |  |  |
|  |  | BMP8B |  |  |  |  |  |  |  |  |  |  |
|  |  | CCL2 |  |  |  |  |  |  |  |  |  |  |
|  |  | IL24 |  |  |  |  |  |  |  |  |  |  |
|  |  | IL18RAP |  |  |  |  |  |  |  |  |  |  |
|  |  | TGFBI |  |  |  |  |  |  |  |  |  |  |
|  |  | TNFSF10 |  |  |  |  |  |  |  |  |  |  |
|  |  | TNFRSF11A | |  |  |  |  |  |  |  |  |  |
|  |  | CXCL5 |  |  |  |  |  |  |  |  |  |  |
|  |  | IL5RA |  |  |  |  |  |  |  |  |  |  |
|  |  | TNFSF9 |  |  |  |  |  |  |  |  |  |  |
|  |  | IL1RL2 |  |  |  |  |  |  |  |  |  |  |
|  |  | TNFRSF13C | |  |  |  |  |  |  |  |  |  |
|  |  | IL36G |  |  |  |  |  |  |  |  |  |  |
|  |  | IL15RA |  |  |  |  |  |  |  |  |  |  |
|  |  | TNFRSF21 |  |  |  |  |  |  |  |  |  |  |
|  |  | CXCL8 |  |  |  |  |  |  |  |  |  |  |
|  |  | IL22RA2 |  |  |  |  |  |  |  |  |  |  |
|  |  | TNFAIP8L2 | |  |  |  |  |  |  |  |  |  |
|  |  | IL18R1 |  |  |  |  |  |  |  |  |  |  |
|  |  | IFNLR1 |  |  |  |  |  |  |  |  |  |  |
|  |  | CXCR6 |  |  |  |  |  |  |  |  |  |  |
|  |  | CCL3L3 |  |  |  |  |  |  |  |  |  |  |
|  |  | TNFRSF1A | |  |  |  |  |  |  |  |  |  |
|  |  | IL17RE |  |  |  |  |  |  |  |  |  |  |
|  |  | IFNGR2 |  |  |  |  |  |  |  |  |  |  |
|  |  | IL17RC |  |  |  |  |  |  |  |  |  |  |
|  |  | TNFAIP8L3 | |  |  |  |  |  |  |  |  |  |
|  |  | ILVBL |  |  |  |  |  |  |  |  |  |  |
|  |  | TGFBRAP1 | |  |  |  |  |  |  |  |  |  |
|  |  | CCL4L1 |  |  |  |  |  |  |  |  |  |  |
|  |  | CSF2RA |  |  |  |  |  |  |  |  |  |  |
|  |  | CCRN4L |  |  |  |  |  |  |  |  |  |  |
|  |  | CCL26 |  |  |  |  |  |  |  |  |  |  |
|  |  | TNFAIP1 |  |  |  |  |  |  |  |  |  |  |
|  |  | CCRL2 |  |  |  |  |  |  |  |  |  |  |
|  |  | IFNA10 |  |  |  |  |  |  |  |  |  |  |
|  |  | TNFRSF17 |  |  |  |  |  |  |  |  |  |  |
|  |  | IFNA13 |  |  |  |  |  |  |  |  |  |  |
|  |  | IL20 |  |  |  |  |  |  |  |  |  |  |
|  |  | IL18BP |  |  |  |  |  |  |  |  |  |  |
|  |  | CCL3L1 |  |  |  |  |  |  |  |  |  |  |
|  |  | TNFSF12-TNFSF13 | |  |  |  |  |  |  |  |  |  |
|  |  | IL5 |  |  |  |  |  |  |  |  |  |  |
|  |  | IL23R |  |  |  |  |  |  |  |  |  |  |
|  |  | IL26 |  |  |  |  |  |  |  |  |  |  |
|  |  | TNF |  |  |  |  |  |  |  |  |  |  |
|  |  | TGFA |  |  |  |  |  |  |  |  |  |  |
|  |  | CSF2 |  |  |  |  |  |  |  |  |  |  |
|  |  | IL1F10 |  |  |  |  |  |  |  |  |  |  |
|  |  | CXCL17 |  |  |  |  |  |  |  |  |  |  |
|  |  | TNFSF13 |  |  |  |  |  |  |  |  |  |  |
|  |  | IFNA4 |  |  |  |  |  |  |  |  |  |  |
|  |  | IL37 |  |  |  |  |  |  |  |  |  |  |
|  |  | IL12A |  |  |  |  |  |  |  |  |  |  |
|  |  | IL7R |  |  |  |  |  |  |  |  |  |  |
|  |  | IFNA1 |  |  |  |  |  |  |  |  |  |  |
|  |  | IL1A |  |  |  |  |  |  |  |  |  |  |
|  |  | IL4 |  |  |  |  |  |  |  |  |  |  |
|  |  | IL2 |  |  |  |  |  |  |  |  |  |  |
|  |  | CCL22 |  |  |  |  |  |  |  |  |  |  |
|  |  | CSF3R |  |  |  |  |  |  |  |  |  |  |
|  |  | IL10 |  |  |  |  |  |  |  |  |  |  |
|  |  | IFNK |  |  |  |  |  |  |  |  |  |  |
|  |  | TGFB2 |  |  |  |  |  |  |  |  |  |  |
|  |  | IL1R2 |  |  |  |  |  |  |  |  |  |  |
|  |  | IL1B |  |  |  |  |  |  |  |  |  |  |
|  |  | IL17F |  |  |  |  |  |  |  |  |  |  |
|  |  | IL27RA |  |  |  |  |  |  |  |  |  |  |
|  |  | IL15 |  |  |  |  |  |  |  |  |  |  |
|  |  | TNFSF8 |  |  |  |  |  |  |  |  |  |  |
|  |  | IL36B |  |  |  |  |  |  |  |  |  |  |
|  |  | XCL1 |  |  |  |  |  |  |  |  |  |  |
|  |  | CXCL16 |  |  |  |  |  |  |  |  |  |  |
|  |  | TNFRSF19 |  |  |  |  |  |  |  |  |  |  |
|  |  | IL3 |  |  |  |  |  |  |  |  |  |  |
|  |  | CCL3 |  |  |  |  |  |  |  |  |  |  |
|  |  | IFNA2 |  |  |  |  |  |  |  |  |  |  |
|  |  | BMPR1B |  |  |  |  |  |  |  |  |  |  |
|  |  | IFNA21 |  |  |  |  |  |  |  |  |  |  |
|  |  | TNFSF18 |  |  |  |  |  |  |  |  |  |  |
|  |  | CCL8 |  |  |  |  |  |  |  |  |  |  |
|  |  | IL17RB |  |  |  |  |  |  |  |  |  |  |
|  |  | TNFRSF25 |  |  |  |  |  |  |  |  |  |  |
|  |  | IL22 |  |  |  |  |  |  |  |  |  |  |
|  |  | IL10RB |  |  |  |  |  |  |  |  |  |  |
|  |  | IFNAR2 |  |  |  |  |  |  |  |  |  |  |
|  |  | CCL18 |  |  |  |  |  |  |  |  |  |  |
|  |  | IFNA16 |  |  |  |  |  |  |  |  |  |  |
|  |  | CSF2RB |  |  |  |  |  |  |  |  |  |  |
|  |  | IL36A |  |  |  |  |  |  |  |  |  |  |
|  |  | TNFAIP3 |  |  |  |  |  |  |  |  |  |  |
|  |  | IL13RA2 |  |  |  |  |  |  |  |  |  |  |
|  |  | IL13RA1 |  |  |  |  |  |  |  |  |  |  |
|  |  | CCR9 |  |  |  |  |  |  |  |  |  |  |
|  |  | TNFRSF10A | |  |  |  |  |  |  |  |  |  |
|  |  | IFNA7 |  |  |  |  |  |  |  |  |  |  |
|  |  | IFNW1 |  |  |  |  |  |  |  |  |  |  |
|  |  | XCL2 |  |  |  |  |  |  |  |  |  |  |
|  |  | TNFSF14 |  |  |  |  |  |  |  |  |  |  |
|  |  | CCR2 |  |  |  |  |  |  |  |  |  |  |
|  |  | BMP15 |  |  |  |  |  |  |  |  |  |  |
|  |  | BMP10 |  |  |  |  |  |  |  |  |  |  |
|  |  | CCL15-CCL14 | |  |  |  |  |  |  |  |  |  |
|  |  | TGFBR1 |  |  |  |  |  |  |  |  |  |  |
|  |  | IFNA5 |  |  |  |  |  |  |  |  |  |  |
|  |  | BMP7 |  |  |  |  |  |  |  |  |  |  |
|  |  | IFNA14 |  |  |  |  |  |  |  |  |  |  |
|  |  | IL20RB |  |  |  |  |  |  |  |  |  |  |
|  |  | IL10RA |  |  |  |  |  |  |  |  |  |  |
|  |  | IFNA17 |  |  |  |  |  |  |  |  |  |  |
|  |  | CCR6 |  |  |  |  |  |  |  |  |  |  |
|  |  | TGFB3 |  |  |  |  |  |  |  |  |  |  |
|  |  | CCL15 |  |  |  |  |  |  |  |  |  |  |
|  |  | CCL4 |  |  |  |  |  |  |  |  |  |  |
|  |  | CCL27 |  |  |  |  |  |  |  |  |  |  |
|  |  | TNFRSF13B | |  |  |  |  |  |  |  |  |  |
|  |  | TNFAIP2 |  |  |  |  |  |  |  |  |  |  |
|  |  | IL31 |  |  |  |  |  |  |  |  |  |  |
|  |  | IL17A |  |  |  |  |  |  |  |  |  |  |
|  |  | TNFSF15 |  |  |  |  |  |  |  |  |  |  |
|  |  | CCL19 |  |  |  |  |  |  |  |  |  |  |
|  |  | IFNA6 |  |  |  |  |  |  |  |  |  |  |
|  |  | IL9 |  |  |  |  |  |  |  |  |  |  |

| **Table S5 The primer sequences used in the present study.** | | |
| --- | --- | --- |
| Gene | Forward primer (5′-3′) | Reverse primer (5′-3′) |
| CDR2L | AGCAGCACGCCAAAGTCTAT | AACTGTGTAGGCGGAAAGCA |
| FSTL3 | CTGTGACCTTAGCCCAGCAA | GCCTCATGAACTGACGTGGA |
| FRMD5 | ACAAGACGGAACTGAGTGGTC | GACACGTCCTTACATGGGTGA |
| SNAI1 | CGAGTGGTTCTTCTGCGCTA | GGGCTGCTGGAAGGTAAACT |
| RGS16 | AACACAGCAAAGAGAATAGAAACTT | CTTTAGGGGCCTCACTGCAA |
| GAPDH | CTGGGCTACACTGAGCACC | AAGTGGTCGTTGAGGGCAATG |

**Table S6 Clinical parameters of 348 patients in TCGA-COAD cohort.**

| id | OS time(months) | OS status | cluster | age | sex | T | N | M | stage | Vascular_Invasion | LymphoVascular_Invasion | Recurred/Progressed |
| --- | --- | --- | --- | --- | --- | --- | --- | --- | --- | --- | --- | --- |
| TCGA-AA-3667 | 13.99 | alive | cluster1 | <65 | Female | T2 | N0 | M0 | I | NO | NO | no |
| TCGA-G4-6307 | 54.99 | alive | cluster1 | <65 | Female | T3 | N1 | M0 | III | YES | YES | no |
| TCGA-SS-A7HO | 60.09 | alive | cluster1 | <65 | Female | T4 | N0 | M0 | II | YES | YES | yes |
| TCGA-AA-A01S | 1.02 | alive | cluster1 | <65 | Female | T3 | N1 | M0 | III | unknown | unknown | no |
| TCGA-CM-4747 | 25 | alive | cluster1 | <65 | Male | T4 | N1 | M1 | IV | NO | YES | no |
| TCGA-AZ-4308 | 109.2 | alive | cluster1 | <65 | Female | T3 | N1 | M0 | III | NO | YES | no |
| TCGA-AA-A01Q | 1.02 | alive | cluster1 | <65 | Female | T3 | N0 | M0 | II | unknown | YES | no |
| TCGA-AA-A02K | 13.99 | dead | cluster1 | <65 | Male | T4 | N2 | M1 | IV | YES | YES | yes |
| TCGA-DM-A28H | 116.98 | alive | cluster1 | <65 | Male | T3 | N2 | M0 | III | NO | NO | yes |
| TCGA-G4-6317 | 35.97 | alive | cluster1 | <65 | Female | T3 | N2 | unknown | III | unknown | unknown | yes |
| TCGA-RU-A8FL | 38.67 | alive | cluster1 | <65 | Male | T3 | N2 | unknown | III | unknown | unknown | yes |
| TCGA-AZ-4313 | 75.89 | alive | cluster1 | <65 | Female | T1 | N0 | M0 | I | YES | YES | no |
| TCGA-AZ-5407 | 88.14 | alive | cluster1 | <65 | Female | T1 | N0 | M0 | I | NO | NO | no |
| TCGA-NH-A8F7 | 17.84 | alive | cluster1 | <65 | Female | T3 | N0 | unknown | II | NO | NO | no |
| TCGA-CA-5256 | 12.45 | alive | cluster1 | <65 | Female | T3 | N0 | M0 | II | unknown | unknown | no |
| TCGA-AY-A71X | 19.32 | alive | cluster1 | <65 | Female | T2 | N0 | M0 | I | NO | NO | no |
| TCGA-F4-6808 | 33.64 | alive | cluster1 | <65 | Female | T1 | N0 | M0 | I | NO | NO | no |
| TCGA-AZ-6608 | 1.94 | dead | cluster1 | <65 | Female | T2 | N1 | M0 | III | YES | YES | unknown |
| TCGA-AA-A017 | 15.01 | alive | cluster1 | <65 | Female | T3 | N0 | M0 | II | NO | YES | no |
| TCGA-A6-2683 | 16.56 | dead | cluster1 | <65 | Female | T4 | N0 | M1 | IV | NO | NO | yes |
| TCGA-AA-3488 | 5.03 | dead | cluster1 | <65 | Male | T3 | N2 | M1 | IV | unknown | unknown | unknown |
| TCGA-AA-A01V | 1.02 | alive | cluster1 | <65 | Male | T2 | N0 | M0 | I | unknown | unknown | no |
| TCGA-A6-6652 | 24.67 | alive | cluster1 | <65 | Male | T3 | N0 | M1 | IV | NO | NO | no |
| TCGA-CM-5864 | 15.01 | alive | cluster1 | <65 | Male | T2 | N0 | M0 | I | NO | NO | no |
| TCGA-D5-6532 | 18.23 | alive | cluster1 | <65 | Male | T3 | N0 | M0 | II | NO | NO | no |
| TCGA-QG-A5YX | 32.95 | alive | cluster1 | <65 | Female | T3 | N0 | unknown | II | YES | NO | no |
| TCGA-CM-4746 | 36.99 | alive | cluster1 | <65 | Male | T2 | N0 | M0 | I | NO | NO | no |
| TCGA-AZ-4315 | 58.34 | alive | cluster1 | <65 | Male | T3 | N0 | M0 | II | NO | NO | no |
| TCGA-AA-3519 | 9.07 | alive | cluster1 | <65 | Male | T3 | N1 | M0 | III | NO | NO | no |
| TCGA-AA-A01T | 33.02 | alive | cluster1 | <65 | Female | T3 | N1 | M0 | III | unknown | YES | no |
| TCGA-DM-A28M | 95.11 | alive | cluster1 | <65 | Male | T3 | N0 | M0 | II | NO | NO | no |
| TCGA-DM-A0XD | 24.41 | dead | cluster1 | >=65 | Male | T3 | N0 | M0 | II | NO | NO | yes |
| TCGA-AA-A03J | 40.93 | alive | cluster1 | >=65 | Female | T2 | N0 | M0 | I | unknown | unknown | no |
| TCGA-AA-A00L | 38.01 | alive | cluster1 | >=65 | Male | T3 | N0 | M0 | II | NO | NO | no |
| TCGA-G4-6304 | 53.58 | alive | cluster1 | >=65 | Female | T4 | N0 | M0 | II | NO | NO | yes |
| TCGA-4N-A93T | 4.8 | alive | cluster1 | >=65 | Male | T4 | N1 | M0 | III | NO | NO | no |
| TCGA-DM-A1D9 | 140.28 | alive | cluster1 | >=65 | Female | T3 | N0 | M0 | II | NO | NO | no |
| TCGA-NH-A50T | 18.17 | alive | cluster1 | >=65 | Female | T3 | N0 | unknown | II | NO | NO | no |
| TCGA-AA-A01Z | 36.99 | alive | cluster1 | >=65 | Male | T3 | N0 | M0 | II | unknown | YES | yes |
| TCGA-DM-A1DB | 44.28 | dead | cluster1 | >=65 | Male | T3 | N0 | M0 | II | NO | NO | unknown |
| TCGA-AA-3542 | 12.98 | alive | cluster1 | >=65 | Male | T3 | N2 | M0 | III | NO | YES | no |
| TCGA-CM-4744 | 20.01 | alive | cluster1 | >=65 | Male | T2 | N0 | M0 | I | YES | YES | no |
| TCGA-A6-6650 | 20.6 | alive | cluster1 | >=65 | Female | T3 | N0 | M0 | II | NO | NO | no |
| TCGA-AZ-4614 | 5.65 | dead | cluster1 | >=65 | Female | T4 | N1 | M1 | IV | YES | YES | unknown |
| TCGA-DM-A1DA | 7.49 | dead | cluster1 | >=65 | Female | T3 | N2 | M0 | III | unknown | unknown | unknown |
| TCGA-AA-3548 | 33.97 | alive | cluster1 | >=65 | Female | T3 | N2 | M0 | III | NO | YES | no |
| TCGA-DM-A0X9 | 119.61 | alive | cluster1 | >=65 | Female | T3 | N0 | M0 | II | NO | NO | no |
| TCGA-AZ-6599 | 6.77 | dead | cluster1 | >=65 | Male | T2 | N0 | unknown | I | NO | NO | unknown |
| TCGA-AA-3561 | 13.93 | alive | cluster1 | >=65 | Male | T3 | N0 | M0 | II | NO | NO | no |
| TCGA-AA-3560 | 19.97 | alive | cluster1 | >=65 | Female | T3 | N2 | M0 | III | NO | YES | no |
| TCGA-AA-3861 | 30.03 | alive | cluster1 | >=65 | Male | T3 | N0 | M0 | II | YES | YES | no |
| TCGA-AA-A01F | 32 | alive | cluster1 | >=65 | Male | T3 | N1 | M0 | III | NO | YES | no |
| TCGA-AA-3972 | 50.95 | alive | cluster1 | >=65 | Male | T3 | N1 | M1 | IV | NO | NO | yes |
| TCGA-DM-A28E | 119.84 | alive | cluster1 | >=65 | Female | T3 | N0 | M0 | II | NO | NO | no |
| TCGA-AD-6888 | 15.51 | dead | cluster1 | >=65 | Male | T3 | N1 | M0 | III | YES | YES | yes |
| TCGA-AA-3502 | 34.99 | alive | cluster1 | >=65 | Male | T2 | N0 | M0 | I | unknown | unknown | no |
| TCGA-AA-A02Y | 39.95 | alive | cluster1 | >=65 | Male | T2 | N0 | M0 | I | YES | YES | no |
| TCGA-D5-5540 | 56.04 | alive | cluster1 | >=65 | Male | T3 | N0 | M0 | II | NO | NO | no |
| TCGA-AY-A54L | 17.25 | alive | cluster1 | >=65 | Female | T2 | N0 | M0 | I | NO | NO | yes |
| TCGA-A6-5656 | 32.88 | alive | cluster1 | >=65 | Male | T2 | N0 | M0 | I | NO | NO | no |
| TCGA-DM-A28C | 81.31 | dead | cluster1 | >=65 | Male | T3 | N0 | M0 | II | unknown | unknown | yes |
| TCGA-G4-6294 | 28.19 | dead | cluster1 | >=65 | Male | T3 | N1 | M1 | IV | NO | NO | yes |
| TCGA-AA-3531 | 34 | alive | cluster1 | >=65 | Female | T3 | N0 | M0 | II | NO | NO | no |
| TCGA-DM-A28G | 60.74 | dead | cluster1 | >=65 | Male | T3 | N0 | M0 | II | NO | NO | unknown |
| TCGA-AA-A004 | 13.93 | alive | cluster1 | >=65 | Male | T3 | N0 | M0 | II | NO | NO | no |
| TCGA-AA-A01I | 30.98 | alive | cluster1 | >=65 | Male | T2 | N0 | M0 | I | NO | NO | no |
| TCGA-5M-AATE | 39.42 | alive | cluster1 | >=65 | Male | T3 | N0 | M0 | II | NO | NO | yes |
| TCGA-AA-3844 | 14.91 | alive | cluster1 | >=65 | Female | T3 | N2 | M0 | III | YES | YES | yes |
| TCGA-D5-6538 | 17.12 | alive | cluster1 | >=65 | Female | T3 | N2 | M0 | III | YES | NO | no |
| TCGA-DM-A1D0 | 130.55 | alive | cluster1 | >=65 | Female | T3 | N0 | M0 | II | NO | NO | no |
| TCGA-CM-5862 | 5.03 | dead | cluster1 | >=65 | Male | T3 | N1 | M1 | IV | YES | NO | yes |
| TCGA-AA-A00W | 14.98 | alive | cluster1 | >=65 | Male | T1 | N0 | M0 | I | NO | NO | no |
| TCGA-AA-A01X | 25.99 | alive | cluster1 | >=65 | Female | T2 | N1 | M0 | III | unknown | YES | yes |
| TCGA-A6-5661 | 33.51 | alive | cluster1 | >=65 | Female | T3 | N0 | M0 | II | NO | NO | no |
| TCGA-DM-A1D4 | 92.67 | dead | cluster1 | >=65 | Male | T3 | N0 | M0 | II | NO | NO | yes |
| TCGA-AA-3514 | 1.02 | alive | cluster1 | >=65 | Female | T2 | N0 | M0 | I | NO | NO | no |
| TCGA-AA-3848 | 10.05 | dead | cluster1 | >=65 | Female | T3 | N2 | M0 | III | YES | YES | unknown |
| TCGA-A6-5659 | 30.42 | alive | cluster1 | >=65 | Male | T2 | N0 | M0 | I | NO | NO | no |
| TCGA-A6-2672 | 46.62 | alive | cluster1 | >=65 | Female | T3 | N1 | M0 | III | YES | YES | no |
| TCGA-DM-A1HA | 131.41 | alive | cluster1 | >=65 | Male | T3 | N2 | M0 | III | unknown | unknown | no |
| TCGA-QL-A97D | 21.88 | alive | cluster1 | >=65 | Female | T2 | N0 | unknown | I | unknown | unknown | no |
| TCGA-AA-3941 | 23.98 | alive | cluster1 | >=65 | Female | T4 | N1 | M1 | IV | NO | YES | no |
| TCGA-A6-6141 | 8.38 | alive | cluster2 | <65 | Male | T3 | N0 | M0 | II | NO | NO | no |
| TCGA-CM-4750 | 8.02 | alive | cluster2 | <65 | Female | T1 | N1 | M0 | III | NO | YES | no |
| TCGA-CM-6161 | 15.01 | alive | cluster2 | <65 | Female | T2 | N0 | M0 | I | NO | NO | no |
| TCGA-AA-3955 | 20.96 | alive | cluster2 | <65 | Male | T2 | N2 | M0 | III | NO | YES | no |
| TCGA-CM-6674 | 12.94 | alive | cluster2 | <65 | Male | T3 | N0 | M0 | II | NO | NO | no |
| TCGA-CM-5344 | 22.01 | alive | cluster2 | <65 | Female | T3 | N1 | M0 | III | NO | YES | no |
| TCGA-A6-5667 | 29.14 | alive | cluster2 | <65 | Female | T3 | N1 | unknown | III | NO | YES | no |
| TCGA-G4-6309 | 85.41 | alive | cluster2 | <65 | Female | T3 | N1 | M0 | III | NO | NO | yes |
| TCGA-AA-3819 | 25 | alive | cluster2 | <65 | Female | T3 | N0 | M0 | II | YES | YES | no |
| TCGA-AA-3663 | 6.96 | alive | cluster2 | <65 | Male | T3 | N0 | M0 | II | NO | NO | no |
| TCGA-CA-5254 | 12.68 | alive | cluster2 | <65 | Female | T3 | N0 | M0 | II | unknown | unknown | no |
| TCGA-A6-2678 | 42.25 | alive | cluster2 | <65 | Female | T3 | N1 | M0 | III | NO | YES | no |
| TCGA-AY-A8YK | 18.82 | alive | cluster2 | <65 | Male | T3 | N2 | M1 | IV | YES | YES | no |
| TCGA-CA-5255 | 12.35 | alive | cluster2 | <65 | Male | T3 | N0 | M0 | II | unknown | unknown | no |
| TCGA-CK-4948 | 147.9 | alive | cluster2 | <65 | Female | T3 | N1 | M0 | III | unknown | unknown | no |
| TCGA-CK-4947 | 17.54 | alive | cluster2 | <65 | Female | T4 | N1 | M0 | III | unknown | unknown | yes |
| TCGA-A6-5662 | 23.59 | alive | cluster2 | <65 | Male | T3 | N2 | M1 | IV | YES | YES | yes |
| TCGA-CM-6164 | 29.01 | alive | cluster2 | <65 | Female | T3 | N0 | M0 | II | YES | NO | no |
| TCGA-AA-A010 | 34.95 | alive | cluster2 | <65 | Female | T4 | N0 | M0 | II | NO | NO | no |
| TCGA-CM-6166 | 21.98 | alive | cluster2 | <65 | Female | T2 | N0 | M0 | I | NO | NO | no |
| TCGA-D5-6541 | 15.57 | alive | cluster2 | <65 | Male | T3 | N0 | M0 | II | NO | NO | no |
| TCGA-AZ-4684 | 64.95 | alive | cluster2 | <65 | Male | T3 | N2 | M1 | IV | unknown | YES | yes |
| TCGA-G4-6293 | 133.08 | alive | cluster2 | <65 | Female | T3 | N1 | M0 | III | NO | NO | no |
| TCGA-AA-A00U | 17.02 | alive | cluster2 | <65 | Male | T3 | N1 | M0 | III | NO | NO | no |
| TCGA-AD-A5EK | 16.43 | alive | cluster2 | <65 | Male | T2 | N0 | unknown | I | NO | unknown | no |
| TCGA-F4-6460 | 31.93 | dead | cluster2 | <65 | Female | T3 | N1 | M0 | III | NO | NO | yes |
| TCGA-AA-3842 | 36.99 | alive | cluster2 | <65 | Male | T2 | N1 | M0 | III | YES | YES | yes |
| TCGA-AA-3660 | 78.02 | alive | cluster2 | <65 | Female | T3 | N0 | M0 | II | NO | NO | no |
| TCGA-D5-6530 | 20.4 | alive | cluster2 | <65 | Male | T2 | N0 | M0 | I | NO | NO | no |
| TCGA-A6-3807 | 34.63 | alive | cluster2 | <65 | Female | T3 | N2 | M0 | III | YES | YES | no |
| TCGA-AA-3673 | 50 | alive | cluster2 | <65 | Female | T3 | N0 | M0 | II | NO | NO | no |
| TCGA-AA-3538 | 25.99 | alive | cluster2 | <65 | Female | T2 | N0 | M0 | I | NO | NO | no |
| TCGA-AA-3509 | 62.91 | alive | cluster2 | <65 | Female | T3 | N0 | M0 | II | NO | NO | no |
| TCGA-AA-3494 | 1.02 | alive | cluster2 | <65 | Male | T3 | N0 | M1 | IV | unknown | unknown | no |
| TCGA-AY-A69D | 17.84 | alive | cluster2 | <65 | Female | T3 | N0 | M0 | II | NO | NO | no |
| TCGA-AA-3968 | 21.98 | alive | cluster2 | <65 | Female | T2 | N0 | M0 | I | NO | NO | no |
| TCGA-A6-6137 | 27.07 | alive | cluster2 | <65 | Male | T3 | N1 | M0 | III | NO | NO | no |
| TCGA-QG-A5YW | 29.43 | alive | cluster2 | <65 | Female | T3 | N2 | unknown | III | NO | NO | no |
| TCGA-CA-5797 | 12.58 | alive | cluster2 | <65 | Male | T3 | N0 | M0 | II | unknown | unknown | no |
| TCGA-A6-6648 | 25.16 | alive | cluster2 | <65 | Male | T3 | N0 | M1 | IV | NO | NO | yes |
| TCGA-A6-A567 | 61.79 | dead | cluster2 | <65 | Male | T3 | N1 | M1 | IV | NO | YES | yes |
| TCGA-AA-3526 | 19.05 | alive | cluster2 | <65 | Male | T2 | N0 | M0 | I | NO | NO | no |
| TCGA-A6-4107 | 32.42 | alive | cluster2 | <65 | Female | T3 | N1 | M0 | III | NO | YES | no |
| TCGA-A6-A56B | 56.21 | dead | cluster2 | <65 | Male | T3 | N1 | M0 | III | NO | YES | yes |
| TCGA-NH-A6GA | 9.92 | dead | cluster2 | <65 | Male | T4 | N2 | unknown | III | YES | YES | yes |
| TCGA-CM-4752 | 13.01 | alive | cluster2 | <65 | Male | T3 | N0 | M0 | II | NO | NO | no |
| TCGA-AA-3971 | 16.06 | alive | cluster2 | <65 | Male | T3 | N1 | M0 | III | NO | NO | no |
| TCGA-G4-6588 | 26.15 | alive | cluster2 | <65 | Female | T3 | N0 | M0 | II | NO | NO | no |
| TCGA-A6-A5ZU | 9.63 | alive | cluster2 | <65 | Male | T3 | N1 | M0 | III | NO | YES | no |
| TCGA-AA-3679 | 15.01 | alive | cluster2 | <65 | Male | T3 | N2 | M1 | IV | NO | YES | no |
| TCGA-CM-5868 | 17.02 | alive | cluster2 | <65 | Female | T4 | N1 | M1 | IV | YES | YES | no |
| TCGA-F4-6806 | 41.39 | alive | cluster2 | <65 | Female | T2 | N0 | M0 | I | NO | NO | yes |
| TCGA-G4-6321 | 22.08 | alive | cluster2 | <65 | Female | T2 | N1 | unknown | III | NO | NO | no |
| TCGA-AA-3517 | 38.96 | alive | cluster2 | <65 | Male | T3 | N0 | M0 | II | NO | NO | yes |
| TCGA-AA-3678 | 46.98 | alive | cluster2 | <65 | Female | T2 | N1 | M0 | III | NO | YES | no |
| TCGA-AZ-4682 | 22.34 | dead | cluster2 | <65 | Male | T3 | N0 | M1 | IV | NO | NO | unknown |
| TCGA-AA-3553 | 23.98 | alive | cluster2 | <65 | Female | T2 | N0 | M0 | I | NO | NO | no |
| TCGA-QG-A5Z2 | 31.27 | alive | cluster2 | <65 | Male | T2 | N0 | M0 | I | NO | NO | no |
| TCGA-A6-6140 | 24.11 | alive | cluster2 | <65 | Male | T3 | N0 | M0 | II | NO | NO | no |
| TCGA-AD-6965 | 26.45 | alive | cluster2 | <65 | Male | T4 | N2 | M0 | III | YES | YES | yes |
| TCGA-CM-6678 | 11.01 | alive | cluster2 | <65 | Female | T4 | N1 | M1 | IV | NO | NO | yes |
| TCGA-CA-6715 | 12.58 | alive | cluster2 | <65 | Male | T3 | N1 | M0 | III | unknown | unknown | no |
| TCGA-CM-5861 | 15.01 | alive | cluster2 | <65 | Female | T3 | N0 | M0 | II | NO | NO | yes |
| TCGA-AA-3532 | 28.98 | alive | cluster2 | <65 | Male | T3 | N0 | M0 | II | NO | NO | no |
| TCGA-D5-6537 | 4.8 | dead | cluster2 | <65 | Male | T3 | N1 | unknown | III | NO | YES | yes |
| TCGA-AA-3511 | 6.96 | alive | cluster2 | <65 | Male | T4 | N0 | M0 | II | NO | YES | yes |
| TCGA-QG-A5YV | 42.74 | alive | cluster2 | <65 | Female | T4 | N1 | unknown | III | NO | NO | no |
| TCGA-CA-6716 | 12.19 | alive | cluster2 | >=65 | Male | T3 | N0 | M0 | II | unknown | unknown | no |
| TCGA-AA-A00E | 29.99 | alive | cluster2 | >=65 | Male | T3 | N0 | M0 | II | NO | NO | no |
| TCGA-AY-5543 | 32.98 | alive | cluster2 | >=65 | Female | T3 | N1 | M1 | IV | NO | NO | no |
| TCGA-AA-3815 | 33.02 | alive | cluster2 | >=65 | Female | T3 | N0 | M0 | II | unknown | YES | no |
| TCGA-AA-3956 | 34 | alive | cluster2 | >=65 | Male | T3 | N0 | M0 | II | NO | NO | no |
| TCGA-AA-3970 | 36.01 | alive | cluster2 | >=65 | Male | T3 | N0 | M0 | II | NO | NO | no |
| TCGA-AA-3930 | 2 | dead | cluster2 | >=65 | Male | T3 | N2 | M1 | IV | YES | YES | unknown |
| TCGA-AY-6386 | 17.81 | alive | cluster2 | >=65 | Female | T3 | N1 | M0 | III | NO | NO | no |
| TCGA-AA-3831 | 17.97 | alive | cluster2 | >=65 | Male | T3 | N0 | M0 | II | YES | YES | no |
| TCGA-AA-A00F | 34 | alive | cluster2 | >=65 | Male | T3 | N2 | M0 | III | NO | YES | no |
| TCGA-AA-3841 | 36.93 | alive | cluster2 | >=65 | Male | T3 | N0 | M0 | II | YES | YES | no |
| TCGA-AA-A00Q | 41.98 | alive | cluster2 | >=65 | Female | T4 | N1 | M0 | III | NO | YES | no |
| TCGA-G4-6315 | 61.86 | alive | cluster2 | >=65 | Male | T3 | N1 | M1 | IV | NO | NO | no |
| TCGA-AA-3680 | 11.01 | dead | cluster2 | >=65 | Female | T4 | N2 | M1 | IV | NO | YES | unknown |
| TCGA-AA-3858 | 31.04 | alive | cluster2 | >=65 | Male | T2 | N0 | M0 | I | YES | YES | no |
| TCGA-AA-3522 | 37.02 | alive | cluster2 | >=65 | Male | T3 | N0 | M0 | II | NO | YES | no |
| TCGA-AA-A029 | 51.94 | alive | cluster2 | >=65 | Male | T3 | N0 | M0 | II | unknown | unknown | no |
| TCGA-AA-3666 | 2 | dead | cluster2 | >=65 | Male | T3 | N1 | M0 | III | NO | NO | unknown |
| TCGA-AA-3952 | 2 | dead | cluster2 | >=65 | Male | T3 | N2 | M0 | III | YES | YES | unknown |
| TCGA-AA-3544 | 13.99 | alive | cluster2 | >=65 | Male | T2 | N0 | M0 | I | NO | NO | no |
| TCGA-AA-3713 | 19.02 | alive | cluster2 | >=65 | Male | T3 | N0 | M1 | IV | unknown | unknown | no |
| TCGA-A6-2677 | 24.31 | dead | cluster2 | >=65 | Female | T3 | N2 | M0 | III | NO | YES | unknown |
| TCGA-CM-5349 | 30.06 | alive | cluster2 | >=65 | Female | T3 | N0 | M0 | II | NO | NO | no |
| TCGA-DM-A0XF | 38.17 | dead | cluster2 | >=65 | Female | T3 | N2 | M0 | III | NO | NO | unknown |
| TCGA-AA-3655 | 60.97 | alive | cluster2 | >=65 | Male | T3 | N0 | M0 | II | NO | NO | no |
| TCGA-D5-6932 | 11.37 | alive | cluster2 | >=65 | Male | T3 | N0 | M0 | II | NO | NO | no |
| TCGA-AA-3973 | 13.04 | alive | cluster2 | >=65 | Male | T4 | N1 | M1 | IV | YES | YES | no |
| TCGA-NH-A50V | 19.32 | alive | cluster2 | >=65 | Male | T3 | N2 | M0 | III | NO | NO | no |
| TCGA-AA-3549 | 20.99 | alive | cluster2 | >=65 | Male | T2 | N0 | M0 | I | NO | NO | no |
| TCGA-CM-4743 | 23.03 | alive | cluster2 | >=65 | Male | T3 | N0 | M0 | II | NO | YES | no |
| TCGA-AA-3685 | 37.02 | alive | cluster2 | >=65 | Male | T3 | N0 | M0 | II | NO | YES | no |
| TCGA-G4-6310 | 63.57 | alive | cluster2 | >=65 | Male | T3 | N1 | M0 | III | NO | NO | no |
| TCGA-AA-A02J | 5.03 | dead | cluster2 | >=65 | Female | T3 | N0 | M1 | IV | YES | YES | unknown |
| TCGA-G4-6295 | 8.34 | alive | cluster2 | >=65 | Female | T3 | N0 | M0 | II | NO | NO | no |
| TCGA-CM-6172 | 11.01 | alive | cluster2 | >=65 | Female | T3 | N1 | M0 | III | NO | NO | no |
| TCGA-AA-A00D | 18.99 | alive | cluster2 | >=65 | Male | T2 | N0 | M0 | I | NO | NO | no |
| TCGA-AA-A00Z | 21.98 | alive | cluster2 | >=65 | Male | T3 | N0 | M0 | II | NO | YES | no |
| TCGA-AA-3976 | 25.99 | alive | cluster2 | >=65 | Male | T2 | N1 | M0 | III | NO | unknown | no |
| TCGA-AA-3510 | 63.93 | alive | cluster2 | >=65 | Male | T3 | N0 | M0 | II | NO | NO | no |
| TCGA-QG-A5Z1 | 8.41 | dead | cluster2 | >=65 | Male | T3 | N1 | unknown | III | YES | YES | unknown |
| TCGA-NH-A6GB | 15.64 | alive | cluster2 | >=65 | Female | T3 | N2 | unknown | III | NO | NO | no |
| TCGA-AA-3864 | 52.96 | alive | cluster2 | >=65 | Male | T3 | N0 | M0 | II | NO | NO | no |
| TCGA-AA-3855 | 32.03 | alive | cluster2 | >=65 | Male | T2 | N0 | M0 | I | YES | YES | no |
| TCGA-A6-2680 | 35.09 | alive | cluster2 | >=65 | Female | T3 | N0 | M0 | II | NO | NO | no |
| TCGA-CM-6170 | 15.01 | alive | cluster2 | >=65 | Female | T2 | N0 | M0 | I | NO | NO | no |
| TCGA-AA-3986 | 19.05 | alive | cluster2 | >=65 | Male | T2 | N0 | M0 | I | NO | NO | no |
| TCGA-G4-6320 | 26.41 | alive | cluster2 | >=65 | Male | T3 | N1 | unknown | III | YES | YES | no |
| TCGA-A6-5660 | 29.17 | alive | cluster2 | >=65 | Male | T3 | N2 | M0 | III | YES | YES | no |
| TCGA-G4-6586 | 35.78 | alive | cluster2 | >=65 | Female | T3 | N0 | M0 | II | YES | YES | no |
| TCGA-DM-A28F | 35.94 | dead | cluster2 | >=65 | Male | T3 | N1 | M0 | III | unknown | unknown | unknown |
| TCGA-AA-A02W | 40.97 | alive | cluster2 | >=65 | Female | T2 | N0 | M0 | I | unknown | unknown | yes |
| TCGA-5M-AAT4 | 1.61 | dead | cluster2 | >=65 | Male | T3 | N0 | M1 | IV | YES | unknown | unknown |
| TCGA-AA-A02H | 2 | dead | cluster2 | >=65 | Female | T3 | N2 | M1 | IV | YES | YES | unknown |
| TCGA-CM-6163 | 14.03 | alive | cluster2 | >=65 | Male | T1 | N0 | M0 | I | YES | NO | no |
| TCGA-CM-6165 | 16.03 | alive | cluster2 | >=65 | Male | T3 | N0 | M0 | II | NO | NO | no |
| TCGA-AA-3846 | 17.02 | alive | cluster2 | >=65 | Female | T3 | N0 | M0 | II | unknown | YES | no |
| TCGA-AA-3867 | 24.01 | alive | cluster2 | >=65 | Male | T3 | N2 | M1 | IV | NO | YES | yes |
| TCGA-AA-A01K | 30.98 | alive | cluster2 | >=65 | Female | T3 | N2 | M0 | III | NO | YES | no |
| TCGA-AA-3851 | 33.05 | alive | cluster2 | >=65 | Male | T3 | N0 | M0 | II | YES | YES | no |
| TCGA-AA-3664 | 53.98 | alive | cluster2 | >=65 | Female | T3 | N0 | M0 | II | NO | NO | no |
| TCGA-AA-3696 | 5.03 | dead | cluster2 | >=65 | Female | T3 | N1 | M1 | IV | NO | NO | unknown |
| TCGA-CM-6677 | 11.07 | alive | cluster2 | >=65 | Female | T3 | N0 | M0 | II | NO | NO | no |
| TCGA-AA-A01C | 15.01 | alive | cluster2 | >=65 | Male | T2 | N1 | M0 | III | NO | YES | no |
| TCGA-D5-6531 | 17.74 | alive | cluster2 | >=65 | Male | T3 | N0 | M0 | II | NO | NO | no |
| TCGA-AA-3982 | 27 | alive | cluster2 | >=65 | Male | T3 | N1 | M0 | III | YES | YES | no |
| TCGA-AA-3869 | 27 | dead | cluster2 | >=65 | Male | T4 | N2 | M1 | IV | NO | YES | unknown |
| TCGA-AD-6889 | 83.18 | dead | cluster2 | >=65 | Male | T3 | N0 | M0 | II | NO | NO | yes |
| TCGA-AA-3681 | 5.98 | alive | cluster2 | >=65 | Female | T3 | N1 | M0 | III | NO | NO | no |
| TCGA-CM-6171 | 14.03 | alive | cluster2 | >=65 | Female | T2 | N0 | M0 | I | NO | NO | no |
| TCGA-AZ-6598 | 49.38 | dead | cluster2 | >=65 | Female | T3 | N0 | unknown | II | NO | NO | unknown |
| TCGA-AA-3506 | 57.98 | alive | cluster2 | >=65 | Male | T2 | N0 | M0 | I | unknown | unknown | no |
| TCGA-AA-3697 | 84.99 | alive | cluster2 | >=65 | Male | T3 | N0 | M0 | II | unknown | unknown | no |
| TCGA-G4-6625 | 91.72 | alive | cluster2 | >=65 | Female | T3 | N0 | M0 | II | NO | NO | yes |
| TCGA-AA-3875 | 18.04 | alive | cluster2 | >=65 | Female | T1 | N0 | M0 | I | NO | NO | no |
| TCGA-AA-3534 | 28.98 | alive | cluster2 | >=65 | Female | T3 | N0 | M0 | II | NO | YES | no |
| TCGA-A6-5666 | 32.69 | alive | cluster2 | >=65 | Male | T4 | N0 | M0 | II | NO | NO | yes |
| TCGA-NH-A8F8 | 16.79 | dead | cluster2 | >=65 | Male | T4 | N2 | M1 | IV | YES | YES | unknown |
| TCGA-AA-A00K | 18.04 | alive | cluster2 | >=65 | Male | T3 | N0 | M0 | II | NO | NO | no |
| TCGA-AA-3495 | 37.02 | alive | cluster2 | >=65 | Male | T2 | N0 | M0 | I | unknown | unknown | no |
| TCGA-AA-3662 | 6.04 | alive | cluster2 | >=65 | Female | T4 | N2 | M1 | IV | NO | NO | no |
| TCGA-AU-3779 | 14.49 | alive | cluster2 | >=65 | Female | T3 | N0 | M0 | II | NO | NO | no |
| TCGA-D5-6535 | 15.11 | alive | cluster2 | >=65 | Female | T3 | N1 | unknown | III | NO | YES | no |
| TCGA-AA-3688 | 18.99 | alive | cluster2 | >=65 | Male | T3 | N1 | M1 | IV | NO | YES | no |
| TCGA-AA-3530 | 19.05 | alive | cluster2 | >=65 | Male | T2 | N0 | M0 | I | NO | NO | no |
| TCGA-AA-3710 | 26.97 | alive | cluster2 | >=65 | Female | T3 | N0 | M0 | II | unknown | YES | no |
| TCGA-AA-3975 | 34.03 | alive | cluster2 | >=65 | Male | T2 | N0 | M0 | I | NO | NO | no |
| TCGA-AA-A00A | 38.01 | alive | cluster2 | >=65 | Male | T3 | N0 | M0 | II | NO | NO | no |
| TCGA-AA-3518 | 1.02 | alive | cluster2 | >=65 | Female | T3 | N0 | M0 | II | NO | NO | no |
| TCGA-AZ-6606 | 11.73 | dead | cluster2 | >=65 | Male | T4 | N2 | M1 | IV | YES | YES | unknown |
| TCGA-CK-5914 | 21.98 | alive | cluster2 | >=65 | Male | T3 | N1 | unknown | III | NO | NO | no |
| TCGA-CK-5912 | 49.05 | dead | cluster2 | >=65 | Male | T2 | N0 | unknown | I | NO | NO | unknown |
| TCGA-AA-A02E | 2.96 | dead | cluster2 | >=65 | Female | T3 | N1 | M1 | IV | YES | YES | unknown |
| TCGA-AZ-4616 | 5.12 | dead | cluster2 | >=65 | Female | T3 | N2 | M1 | IV | YES | YES | unknown |
| TCGA-T9-A92H | 11.89 | alive | cluster2 | >=65 | Male | T3 | N0 | M0 | II | NO | NO | yes |
| TCGA-AA-3562 | 19.97 | alive | cluster2 | >=65 | Male | T3 | N2 | M0 | III | NO | YES | no |
| TCGA-CM-5341 | 29.04 | alive | cluster2 | >=65 | Female | T2 | N1 | M0 | III | NO | YES | no |
| TCGA-AA-3862 | 30.03 | alive | cluster2 | >=65 | Male | T3 | N0 | M0 | II | unknown | YES | no |
| TCGA-AA-3812 | 35.02 | alive | cluster2 | >=65 | Female | T3 | N0 | M0 | II | unknown | YES | yes |
| TCGA-AA-3496 | 1.02 | alive | cluster2 | >=65 | Female | T3 | N0 | M0 | II | unknown | unknown | no |
| TCGA-AA-3939 | 12.98 | alive | cluster2 | >=65 | Male | T3 | N0 | M0 | II | NO | NO | no |
| TCGA-AA-A00O | 27 | alive | cluster2 | >=65 | Female | T3 | N2 | M0 | III | NO | YES | no |
| TCGA-AA-3989 | 7.95 | dead | cluster2 | >=65 | Male | T3 | N2 | M1 | IV | NO | YES | unknown |
| TCGA-A6-5665 | 22.04 | alive | cluster2 | >=65 | Female | T3 | N0 | M0 | II | NO | YES | yes |
| TCGA-AA-3979 | 23.98 | alive | cluster2 | >=65 | Male | T3 | N0 | M0 | II | NO | NO | no |
| TCGA-AA-3675 | 47.01 | alive | cluster2 | >=65 | Male | T3 | N0 | M0 | II | NO | NO | no |
| TCGA-AA-3552 | 13.01 | dead | cluster2 | >=65 | Male | T3 | N2 | M0 | III | NO | YES | yes |
| TCGA-AA-3524 | 36.01 | alive | cluster2 | >=65 | Male | T3 | N0 | M0 | II | NO | NO | no |
| TCGA-AA-3520 | 24.01 | alive | cluster2 | >=65 | Female | T3 | N0 | M0 | II | NO | NO | no |
| TCGA-CK-6747 | 82.88 | alive | cluster2 | >=65 | Female | T3 | N0 | unknown | II | NO | NO | no |
| TCGA-AA-3980 | 27 | alive | cluster2 | >=65 | Female | T2 | N0 | M0 | I | YES | YES | no |
| TCGA-AA-3492 | 3.02 | dead | cluster2 | >=65 | Female | T3 | N0 | M0 | II | NO | NO | unknown |
| TCGA-AA-3525 | 8.05 | alive | cluster2 | >=65 | Male | T3 | N1 | M0 | III | NO | NO | no |
| TCGA-NH-A5IV | 19.32 | alive | cluster2 | >=65 | Female | T3 | N0 | unknown | II | NO | NO | no |
| TCGA-G4-6298 | 23.49 | dead | cluster2 | >=65 | Male | T4 | N1 | unknown | III | NO | NO | yes |
| TCGA-G4-6626 | 46.71 | dead | cluster2 | >=65 | Male | T3 | N0 | M0 | II | NO | NO | unknown |
| TCGA-D5-6927 | 9.43 | alive | cluster3 | <65 | Male | T3 | N0 | M0 | II | NO | NO | no |
| TCGA-CM-6675 | 13.04 | alive | cluster3 | <65 | Male | T3 | N2 | M1 | IV | YES | NO | yes |
| TCGA-AZ-4323 | 1.41 | dead | cluster3 | <65 | Male | T4 | N2 | M1 | IV | YES | unknown | unknown |
| TCGA-5M-AAT6 | 9.53 | dead | cluster3 | <65 | Female | T4 | N2 | M1 | IV | YES | YES | yes |
| TCGA-F4-6461 | 11.1 | dead | cluster3 | <65 | Female | T4 | N2 | M0 | III | NO | NO | yes |
| TCGA-AZ-5403 | 62.75 | dead | cluster3 | <65 | Male | T3 | N0 | unknown | II | NO | NO | yes |
| TCGA-CM-5860 | 32 | alive | cluster3 | <65 | Male | T3 | N0 | M0 | II | YES | NO | no |
| TCGA-CA-6718 | 10.05 | dead | cluster3 | <65 | Male | T3 | N0 | M0 | II | unknown | unknown | yes |
| TCGA-A6-2685 | 37.22 | alive | cluster3 | <65 | Female | T3 | N0 | M0 | II | NO | NO | yes |
| TCGA-D5-6929 | 13.4 | alive | cluster3 | <65 | Female | T3 | N1 | M1 | IV | NO | YES | no |
| TCGA-AY-4070 | 16.29 | dead | cluster3 | <65 | Female | T3 | N2 | M0 | III | YES | YES | yes |
| TCGA-D5-6898 | 7.52 | alive | cluster3 | <65 | Female | T2 | N0 | M0 | I | NO | NO | no |
| TCGA-F4-6807 | 43 | alive | cluster3 | <65 | Female | T3 | N2 | M0 | III | NO | NO | no |
| TCGA-F4-6809 | 13.24 | dead | cluster3 | <65 | Female | T3 | N1 | M1 | IV | NO | NO | unknown |
| TCGA-AA-3860 | 31.04 | alive | cluster3 | <65 | Female | T3 | N1 | M0 | III | YES | YES | no |
| TCGA-G4-6303 | 65.8 | dead | cluster3 | <65 | Female | T3 | N1 | M1 | IV | YES | YES | yes |
| TCGA-A6-6651 | 21.75 | alive | cluster3 | <65 | Female | T3 | N1 | unknown | III | NO | YES | no |
| TCGA-G4-6297 | 82.33 | alive | cluster3 | <65 | Female | T3 | N2 | M1 | IV | YES | YES | yes |
| TCGA-A6-6142 | 25.07 | alive | cluster3 | <65 | Female | T3 | N1 | M1 | IV | YES | YES | yes |
| TCGA-D5-6923 | 12.42 | alive | cluster3 | <65 | Male | T2 | N0 | M0 | I | NO | NO | no |
| TCGA-CM-6167 | 14.98 | alive | cluster3 | <65 | Female | T3 | N2 | M0 | III | YES | YES | no |
| TCGA-CM-6679 | 10.05 | alive | cluster3 | <65 | Male | T3 | N0 | M0 | II | NO | NO | no |
| TCGA-F4-6805 | 34.4 | alive | cluster3 | <65 | Female | T3 | N0 | M0 | II | NO | NO | no |
| TCGA-CK-5913 | 51.28 | alive | cluster3 | <65 | Female | T3 | N0 | unknown | II | NO | NO | no |
| TCGA-F4-6569 | 35.71 | alive | cluster3 | <65 | Male | T2 | N0 | M0 | I | NO | NO | no |
| TCGA-D5-5538 | 54.57 | dead | cluster3 | <65 | Female | T3 | N1 | M0 | III | NO | NO | yes |
| TCGA-F4-6459 | 8.61 | dead | cluster3 | <65 | Female | T3 | N2 | M0 | III | NO | NO | unknown |
| TCGA-A6-6138 | 22.5 | alive | cluster3 | <65 | Male | T2 | N0 | M0 | I | NO | NO | no |
| TCGA-AA-3554 | 17.94 | alive | cluster3 | <65 | Female | T3 | N0 | M0 | II | NO | NO | no |
| TCGA-CM-4751 | 27 | alive | cluster3 | <65 | Male | T3 | N1 | M0 | III | YES | YES | no |
| TCGA-A6-3810 | 36.5 | alive | cluster3 | <65 | Male | T3 | N0 | M0 | II | NO | NO | no |
| TCGA-AA-3833 | 15.93 | alive | cluster3 | <65 | Female | T3 | N0 | M0 | II | YES | YES | no |
| TCGA-D5-5541 | 55.88 | alive | cluster3 | <65 | Male | T3 | N1 | M0 | III | NO | NO | no |
| TCGA-AZ-6600 | 12.09 | dead | cluster3 | <65 | Male | T4 | N1 | M1 | IV | YES | YES | unknown |
| TCGA-F4-6703 | 47.83 | alive | cluster3 | <65 | Male | T3 | N0 | M0 | II | NO | NO | no |
| TCGA-D5-6926 | 9.03 | alive | cluster3 | >=65 | Male | T4 | N1 | M0 | III | NO | NO | no |
| TCGA-A6-6654 | 23.85 | alive | cluster3 | >=65 | Female | T3 | N1 | M0 | III | NO | YES | no |
| TCGA-A6-5657 | 31.6 | alive | cluster3 | >=65 | Male | T3 | N1 | M0 | III | NO | YES | no |
| TCGA-A6-6649 | 24.15 | alive | cluster3 | >=65 | Male | T3 | N1 | M0 | III | YES | YES | no |
| TCGA-CM-6169 | 13.01 | alive | cluster3 | >=65 | Male | T3 | N0 | M0 | II | YES | YES | no |
| TCGA-D5-6924 | 14.29 | alive | cluster3 | >=65 | Male | T3 | N0 | M0 | II | NO | NO | no |
| TCGA-AZ-6601 | 99.93 | dead | cluster3 | >=65 | Male | T3 | N0 | M0 | II | YES | YES | yes |
| TCGA-AZ-6607 | 3.19 | dead | cluster3 | >=65 | Male | T4 | N2 | M1 | IV | YES | YES | unknown |
| TCGA-D5-6529 | 20.17 | alive | cluster3 | >=65 | Male | T3 | N0 | M0 | II | NO | NO | yes |
| TCGA-AU-6004 | 27.07 | alive | cluster3 | >=65 | Female | T2 | N0 | M0 | I | NO | NO | no |
| TCGA-G4-6299 | 74.51 | alive | cluster3 | >=65 | Male | T3 | N2 | M0 | III | YES | YES | no |
| TCGA-A6-2682 | 13.93 | dead | cluster3 | >=65 | Male | T4 | N1 | M1 | IV | YES | YES | yes |
| TCGA-F4-6855 | 47.37 | alive | cluster3 | >=65 | Female | T3 | N0 | M0 | II | NO | NO | no |
| TCGA-CK-5916 | 21.12 | dead | cluster3 | >=65 | Female | T1 | N0 | M0 | I | NO | YES | yes |
| TCGA-AA-3870 | 29.96 | alive | cluster3 | >=65 | Female | T3 | N2 | M1 | IV | NO | YES | no |
| TCGA-CM-5348 | 22.96 | alive | cluster3 | >=65 | Male | T3 | N1 | M0 | III | NO | YES | no |
| TCGA-D5-6536 | 17.84 | alive | cluster3 | >=65 | Male | T3 | N0 | M0 | II | NO | NO | yes |
| TCGA-A6-3808 | 33.31 | alive | cluster3 | >=65 | Male | T3 | N0 | M0 | II | NO | NO | no |
| TCGA-A6-2681 | 45.57 | alive | cluster3 | >=65 | Female | T3 | N0 | M0 | II | NO | NO | yes |
| TCGA-AA-3489 | 7.03 | dead | cluster3 | >=65 | Male | T3 | N0 | M0 | II | unknown | unknown | unknown |
| TCGA-A6-2684 | 37.02 | alive | cluster3 | >=65 | Female | T2 | N0 | M0 | I | NO | NO | yes |
| TCGA-A6-2676 | 42.87 | dead | cluster3 | >=65 | Female | T4 | N0 | M0 | II | NO | NO | unknown |
| TCGA-D5-6922 | 10.12 | alive | cluster3 | >=65 | Male | T3 | N1 | M0 | III | NO | YES | no |
| TCGA-G4-6314 | 35.91 | alive | cluster3 | >=65 | Female | T3 | N2 | M1 | IV | YES | YES | yes |
| TCGA-AZ-6605 | 5.22 | dead | cluster3 | >=65 | Male | T4 | N1 | M0 | III | YES | YES | unknown |
| TCGA-D5-6931 | 11.99 | alive | cluster3 | >=65 | Male | T4 | N2 | M0 | III | NO | YES | no |
| TCGA-D5-6920 | 12.39 | alive | cluster3 | >=65 | Female | T3 | N0 | M0 | II | NO | NO | no |
| TCGA-CA-6719 | 14.29 | alive | cluster3 | >=65 | Male | T3 | N0 | M0 | II | unknown | unknown | yes |
| TCGA-F4-6570 | 6.18 | dead | cluster3 | >=65 | Female | T3 | N0 | M0 | II | NO | NO | unknown |
| TCGA-CM-6680 | 12.02 | alive | cluster3 | >=65 | Female | T3 | N2 | M0 | III | NO | NO | no |
| TCGA-AA-3866 | 17.02 | alive | cluster3 | >=65 | Female | T2 | N0 | M0 | I | NO | NO | no |
| TCGA-DM-A28A | 26.45 | dead | cluster3 | >=65 | Male | T3 | N2 | M0 | III | NO | NO | unknown |
| TCGA-A6-2675 | 43.4 | alive | cluster3 | >=65 | Male | T3 | N0 | unknown | II | NO | NO | no |
| TCGA-G4-6628 | 79.63 | alive | cluster3 | >=65 | Male | T2 | N0 | M0 | I | NO | NO | no |
| TCGA-A6-4105 | 14.52 | dead | cluster3 | >=65 | Male | T3 | N0 | M0 | II | NO | NO | yes |
| TCGA-A6-5664 | 22.08 | alive | cluster3 | >=65 | Male | T4 | N2 | unknown | III | YES | YES | yes |
| TCGA-AA-A01P | 38.04 | dead | cluster3 | >=65 | Female | T3 | N1 | M0 | III | unknown | YES | yes |
| TCGA-G4-6311 | 39.39 | alive | cluster3 | >=65 | Male | T3 | N1 | unknown | III | YES | YES | no |
| TCGA-AD-6548 | 21.35 | alive | cluster3 | >=65 | Female | T2 | N0 | M0 | I | unknown | NO | no |
| TCGA-A6-2686 | 36.99 | dead | cluster3 | >=65 | Female | T3 | N0 | M0 | II | NO | NO | unknown |
| TCGA-CM-6676 | 11.07 | alive | cluster3 | >=65 | Male | T2 | N0 | M0 | I | NO | YES | no |
| TCGA-A6-6782 | 20.27 | alive | cluster3 | >=65 | Male | T4 | N0 | unknown | II | unknown | NO | no |
| TCGA-A6-6653 | 24.38 | alive | cluster3 | >=65 | Male | T2 | N0 | M0 | I | NO | NO | no |
| TCGA-CM-6168 | 12.98 | alive | cluster3 | >=65 | Female | T3 | N0 | M0 | II | YES | NO | no |
| TCGA-AA-A02R | 22.01 | dead | cluster3 | >=65 | Female | T3 | N0 | M0 | II | YES | YES | unknown |
| TCGA-AD-6895 | 25.07 | alive | cluster3 | >=65 | Male | T3 | N1 | M0 | III | YES | YES | no |
| TCGA-AZ-4615 | 32.92 | alive | cluster3 | >=65 | Male | T3 | N1 | M0 | III | YES | YES | yes |
| TCGA-G4-6627 | 74.74 | alive | cluster3 | >=65 | Male | T3 | N0 | M0 | II | NO | NO | yes |
| TCGA-A6-2671 | 43.73 | dead | cluster3 | >=65 | Male | T3 | N2 | M1 | IV | YES | YES | yes |

**Table S7 Clinical parameters of 210 patients in the GSE17538 dataset.**

| id | OS time(months) | OS status | cluster | age | sex | stage | grade | Recurred |
| --- | --- | --- | --- | --- | --- | --- | --- | --- |
| GSM437093 | 142.55 | alive | cluster1 | >=65 | male | 1 | 2 | no |
| GSM437094 | 122.72 | alive | cluster1 | <65 | male | 1 | 1 | no |
| GSM437095 | 28.96 | dead | cluster1 | <65 | male | 1 | 2 | no |
| GSM437096 | 119.21 | alive | cluster1 | <65 | female | 1 | 1 | no |
| GSM437097 | 59.53 | alive | cluster1 | <65 | male | 1 | 2 | no |
| GSM437099 | 82.29 | alive | cluster1 | >=65 | male | 1 | 1 | no |
| GSM437101 | 65.88 | dead | cluster1 | >=65 | male | 1 | 2 | no |
| GSM437104 | 89.75 | alive | cluster1 | <65 | male | 1 | 2 | no |
| GSM437105 | 76.07 | alive | cluster1 | >=65 | male | 1 | 2 | no |
| GSM437107 | 52.24 | alive | cluster1 | >=65 | male | 1 | 1 | no |
| GSM437108 | 64.1 | alive | cluster1 | >=65 | female | 1 | 2 | no |
| GSM437110 | 57.79 | alive | cluster1 | <65 | male | 1 | 2 | no |
| GSM437114 | 3.64 | alive | cluster1 | >=65 | male | 1 | 2 | no |
| GSM437115 | 36.59 | alive | cluster1 | <65 | male | 1 | 1 | no |
| GSM437126 | 52.5 | dead | cluster1 | >=65 | female | 2 | 2 | no |
| GSM437128 | 88.99 | alive | cluster1 | >=65 | male | 2 | 2 | no |
| GSM437129 | 102.44 | alive | cluster1 | <65 | male | 2 | 2 | no |
| GSM437131 | 85.28 | alive | cluster1 | >=65 | female | 2 | 2 | no |
| GSM437136 | 70.65 | alive | cluster1 | >=65 | female | 2 | 2 | no |
| GSM437140 | 80.25 | alive | cluster1 | >=65 | female | 2 | 2 | no |
| GSM437141 | 40.6 | alive | cluster1 | >=65 | female | 2 | 1 | no |
| GSM437143 | 31.06 | dead | cluster1 | >=65 | female | 2 | 3 | yes |
| GSM437146 | 63.25 | alive | cluster1 | >=65 | male | 2 | 2 | no |
| GSM437147 | 34.09 | alive | cluster1 | >=65 | female | 2 | 3 | no |
| GSM437148 | 52.14 | alive | cluster1 | >=65 | female | 2 | 2 | no |
| GSM437150 | 59.96 | alive | cluster1 | >=65 | male | 2 | 1 | no |
| GSM437153 | 45.99 | alive | cluster1 | >=65 | male | 2 | 2 | no |
| GSM437154 | 60.42 | alive | cluster1 | >=65 | male | 2 | 2 | no |
| GSM437158 | 49.87 | alive | cluster1 | >=65 | female | 2 | 2 | no |
| GSM437160 | 44.97 | alive | cluster1 | <65 | male | 2 | 2 | no |
| GSM437164 | 41.52 | alive | cluster1 | >=65 | female | 2 | 2 | no |
| GSM437165 | 22.22 | alive | cluster1 | >=65 | female | 2 | 2 | unknown |
| GSM437167 | 39.68 | alive | cluster1 | >=65 | male | 2 | 2 | no |
| GSM437168 | 25.61 | alive | cluster1 | >=65 | male | 2 | 2 | no |
| GSM437179 | 112.33 | alive | cluster1 | <65 | female | 3 | 2 | no |
| GSM437185 | 99.51 | alive | cluster1 | <65 | female | 3 | 1 | no |
| GSM437186 | 20.44 | dead | cluster1 | <65 | male | 3 | 2 | no |
| GSM437193 | 22.25 | dead | cluster1 | >=65 | female | 3 | 1 | no |
| GSM437194 | 74.53 | alive | cluster1 | <65 | male | 3 | 2 | no |
| GSM437213 | 52.86 | alive | cluster1 | >=65 | male | 3 | 3 | no |
| GSM437216 | 56.67 | alive | cluster1 | >=65 | male | 3 | 3 | no |
| GSM437218 | 4.24 | dead | cluster1 | >=65 | female | 3 | 2 | no |
| GSM437219 | 49.11 | alive | cluster1 | >=65 | female | 3 | 2 | no |
| GSM437220 | 49.57 | alive | cluster1 | <65 | female | 3 | 2 | no |
| GSM437228 | 31.92 | alive | cluster1 | >=65 | female | 3 | 2 | no |
| GSM437230 | 24.62 | alive | cluster1 | <65 | female | 3 | 2 | no |
| GSM437232 | 130.75 | alive | cluster1 | >=65 | female | 4 | 2 | no |
| GSM437236 | 5.72 | dead | cluster1 | >=65 | male | 4 | 2 | unknown |
| GSM437238 | 95.07 | alive | cluster1 | <65 | male | 4 | 2 | no |
| GSM437240 | 28.89 | dead | cluster1 | <65 | male | 4 | 2 | unknown |
| GSM437246 | 5.12 | dead | cluster1 | >=65 | female | 4 | 2 | unknown |
| GSM437248 | 33.17 | dead | cluster1 | <65 | male | 4 | 3 | yes |
| GSM437249 | 50.53 | dead | cluster1 | <65 | male | 4 | 2 | no |
| GSM437252 | 34.02 | dead | cluster1 | >=65 | female | 4 | 2 | unknown |
| GSM437253 | 1.44 | dead | cluster1 | <65 | male | 4 | 3 | unknown |
| GSM437255 | 60.75 | alive | cluster1 | <65 | female | 4 | 2 | no |
| GSM437256 | 37.44 | dead | cluster1 | >=65 | female | 4 | 2 | unknown |
| GSM437258 | 19.85 | dead | cluster1 | >=65 | female | 4 | 2 | unknown |
| GSM437259 | 31.85 | alive | cluster1 | <65 | male | 4 | 2 | unknown |
| GSM437260 | 8.61 | dead | cluster1 | <65 | male | 4 | 2 | unknown |
| GSM437261 | 42.27 | alive | cluster1 | >=65 | male | 4 | 2 | yes |
| GSM437266 | 32.67 | alive | cluster1 | >=65 | female | 4 | 3 | yes |
| GSM437268 | 22.78 | dead | cluster1 | >=65 | male | 4 | 2 | unknown |
| GSM437269 | 22.35 | alive | cluster1 | <65 | male | 4 | 2 | unknown |
| GSM437273 | 60.03287671 | alive | cluster1 | <65 | female | 3 | 1 | no |
| GSM437275 | 60.03287671 | alive | cluster1 | <65 | female | 3 | 2 | no |
| GSM437277 | 57.0739726 | alive | cluster1 | <65 | male | 3 | 2 | no |
| GSM437283 | 59.76986301 | alive | cluster1 | >=65 | female | 1 | 2 | no |
| GSM437285 | 54.50958904 | alive | cluster1 | >=65 | female | 1 | 2 | no |
| GSM437299 | 68.05479452 | alive | cluster1 | >=65 | male | 2 | 2 | no |
| GSM437300 | 50.1369863 | alive | cluster1 | <65 | male | 3 | 2 | no |
| GSM437303 | 46.45479452 | alive | cluster1 | <65 | male | 3 | 2 | no |
| GSM437309 | 46.98082192 | alive | cluster1 | <65 | female | 3 | 3 | no |
| GSM437312 | 40.66849315 | alive | cluster1 | <65 | male | 3 | 2 | no |
| GSM437313 | 40.73424658 | alive | cluster1 | <65 | male | 2 | 2 | no |
| GSM437321 | 32.51506849 | dead | cluster1 | <65 | female | 4 | 2 | unknown |
| GSM437324 | 41.42465753 | alive | cluster1 | >=65 | male | 2 | 2 | no |
| GSM437098 | 68.81 | dead | cluster2 | <65 | female | 1 | 2 | yes |
| GSM437100 | 110.82 | alive | cluster2 | <65 | male | 1 | 2 | no |
| GSM437103 | 16.47 | dead | cluster2 | >=65 | female | 1 | 2 | no |
| GSM437106 | 67.82 | alive | cluster2 | >=65 | female | 1 | 2 | no |
| GSM437109 | 55.13 | alive | cluster2 | >=65 | female | 1 | 2 | no |
| GSM437112 | 43.79 | alive | cluster2 | >=65 | female | 1 | 2 | no |
| GSM437113 | 30.27 | alive | cluster2 | <65 | male | 1 | 2 | no |
| GSM437116 | 20.02 | alive | cluster2 | >=65 | male | 1 | 2 | no |
| GSM437117 | 118.58 | alive | cluster2 | >=65 | female | 2 | 2 | no |
| GSM437120 | 127.29 | alive | cluster2 | <65 | female | 2 | 2 | no |
| GSM437121 | 120.62 | alive | cluster2 | <65 | female | 2 | 2 | yes |
| GSM437122 | 106.94 | alive | cluster2 | >=65 | male | 2 | 2 | no |
| GSM437123 | 11.7 | dead | cluster2 | >=65 | female | 2 | 2 | unknown |
| GSM437132 | 26.53 | alive | cluster2 | >=65 | female | 2 | 2 | no |
| GSM437137 | 76.04 | alive | cluster2 | >=65 | male | 2 | 2 | no |
| GSM437138 | 79.13 | alive | cluster2 | <65 | female | 2 | 1 | no |
| GSM437139 | 83.99 | alive | cluster2 | >=65 | female | 2 | 2 | yes |
| GSM437142 | 95.37 | alive | cluster2 | <65 | female | 2 | 3 | yes |
| GSM437144 | 37.31 | dead | cluster2 | >=65 | male | 2 | 2 | no |
| GSM437151 | 47.86 | alive | cluster2 | <65 | male | 2 | 2 | no |
| GSM437152 | 58.45 | alive | cluster2 | <65 | female | 2 | 2 | no |
| GSM437159 | 25.24 | alive | cluster2 | >=65 | male | 2 | 2 | no |
| GSM437162 | 26.66 | alive | cluster2 | >=65 | male | 2 | 2 | no |
| GSM437163 | 36.78 | alive | cluster2 | <65 | female | 2 | 2 | no |
| GSM437166 | 33.2 | alive | cluster2 | <65 | male | 2 | 2 | no |
| GSM437169 | 83.99 | alive | cluster2 | >=65 | female | 2 | 2 | yes |
| GSM437170 | 15.05 | alive | cluster2 | <65 | male | 2 | 2 | no |
| GSM437172 | 18.18 | alive | cluster2 | >=65 | male | 2 | 2 | no |
| GSM437177 | 70.45 | dead | cluster2 | <65 | male | 3 | 2 | yes |
| GSM437182 | 54.04 | dead | cluster2 | >=65 | female | 3 | 2 | yes |
| GSM437183 | 103.98 | alive | cluster2 | <65 | male | 3 | 1 | yes |
| GSM437188 | 10.02 | dead | cluster2 | >=65 | male | 3 | 2 | yes |
| GSM437189 | 41.45 | dead | cluster2 | <65 | male | 3 | 2 | yes |
| GSM437191 | 85.61 | alive | cluster2 | >=65 | female | 3 | 2 | no |
| GSM437192 | 78.87 | alive | cluster2 | <65 | male | 3 | 2 | no |
| GSM437195 | 59.07 | dead | cluster2 | >=65 | male | 3 | 2 | no |
| GSM437198 | 79.39 | alive | cluster2 | <65 | male | 3 | 1 | no |
| GSM437199 | 74.07 | alive | cluster2 | >=65 | male | 3 | 2 | yes |
| GSM437205 | 38.72 | dead | cluster2 | >=65 | female | 3 | 2 | no |
| GSM437206 | 54.9 | dead | cluster2 | >=65 | female | 3 | 2 | no |
| GSM437207 | 67.92 | alive | cluster2 | >=65 | female | 3 | 2 | no |
| GSM437208 | 58.45 | alive | cluster2 | <65 | female | 3 | 2 | no |
| GSM437214 | 32.05 | dead | cluster2 | <65 | male | 3 | 2 | yes |
| GSM437215 | 55.95 | alive | cluster2 | >=65 | male | 3 | 2 | no |
| GSM437217 | 50.43 | alive | cluster2 | <65 | male | 3 | 2 | no |
| GSM437221 | 50.49 | alive | cluster2 | <65 | male | 3 | 2 | no |
| GSM437227 | 116.64 | alive | cluster2 | >=65 | male | 3 | 2 | yes |
| GSM437231 | 18.54 | dead | cluster2 | <65 | male | 4 | 2 | unknown |
| GSM437233 | 17.62 | dead | cluster2 | <65 | female | 4 | 1 | unknown |
| GSM437237 | 15.28 | dead | cluster2 | >=65 | male | 4 | 2 | unknown |
| GSM437241 | 3.25 | dead | cluster2 | >=65 | female | 4 | 3 | unknown |
| GSM437243 | 18.77 | dead | cluster2 | >=65 | male | 4 | 2 | yes |
| GSM437244 | 57.73 | dead | cluster2 | <65 | male | 4 | 2 | unknown |
| GSM437245 | 14.39 | dead | cluster2 | >=65 | female | 4 | 2 | unknown |
| GSM437247 | 4.99 | dead | cluster2 | <65 | male | 4 | 3 | unknown |
| GSM437251 | 8.02 | dead | cluster2 | <65 | male | 4 | 2 | unknown |
| GSM437254 | 36.95 | dead | cluster2 | >=65 | male | 4 | 3 | unknown |
| GSM437262 | 15.25 | dead | cluster2 | >=65 | male | 4 | 2 | unknown |
| GSM437264 | 9.96 | dead | cluster2 | >=65 | female | 4 | 2 | no |
| GSM437271 | 63.91232877 | alive | cluster2 | <65 | female | 3 | 2 | no |
| GSM437276 | 60.55890411 | alive | cluster2 | >=65 | female | 3 | 2 | yes |
| GSM437279 | 64.86575342 | alive | cluster2 | <65 | male | 1 | 2 | no |
| GSM437280 | 54.73972603 | alive | cluster2 | >=65 | male | 3 | 2 | no |
| GSM437302 | 74.30136986 | alive | cluster2 | <65 | female | 4 | 2 | unknown |
| GSM437304 | 76.76712329 | alive | cluster2 | <65 | female | 4 | 2 | no |
| GSM437305 | 23.40821918 | dead | cluster2 | >=65 | male | 4 | 2 | unknown |
| GSM437320 | 6.904109589 | dead | cluster2 | <65 | female | 4 | 2 | unknown |
| GSM437322 | 16.14246575 | dead | cluster2 | <65 | female | 4 | 2 | unknown |
| GSM437102 | 84.13 | alive | cluster3 | >=65 | male | 1 | 2 | no |
| GSM437111 | 50.1 | alive | cluster3 | >=65 | male | 1 | 2 | no |
| GSM437118 | 55.33 | alive | cluster3 | >=65 | female | 2 | 3 | no |
| GSM437119 | 134.86 | dead | cluster3 | <65 | male | 2 | 2 | yes |
| GSM437124 | 28.79 | dead | cluster3 | >=65 | male | 2 | 2 | yes |
| GSM437125 | 26.82 | dead | cluster3 | >=65 | male | 2 | 2 | no |
| GSM437127 | 24.03 | dead | cluster3 | <65 | female | 2 | 3 | yes |
| GSM437130 | 33.73 | dead | cluster3 | <65 | female | 2 | 2 | yes |
| GSM437133 | 37.31 | dead | cluster3 | >=65 | female | 2 | 1 | yes |
| GSM437134 | 86.43 | alive | cluster3 | >=65 | female | 2 | 2 | no |
| GSM437135 | 55.92 | alive | cluster3 | >=65 | female | 2 | 2 | no |
| GSM437145 | 22.02 | dead | cluster3 | >=65 | female | 2 | 2 | yes |
| GSM437149 | 66.04 | alive | cluster3 | >=65 | female | 2 | 2 | no |
| GSM437155 | 37.67 | alive | cluster3 | <65 | female | 2 | 2 | no |
| GSM437156 | 15.71 | alive | cluster3 | >=65 | male | 2 | 2 | no |
| GSM437157 | 39.25 | alive | cluster3 | <65 | female | 2 | 3 | no |
| GSM437161 | 35.9 | alive | cluster3 | <65 | male | 2 | 2 | no |
| GSM437171 | 8.38 | alive | cluster3 | <65 | female | 2 | 1 | no |
| GSM437173 | 27.15 | dead | cluster3 | <65 | female | 2 | 2 | no |
| GSM437174 | 24.26 | dead | cluster3 | <65 | male | 3 | 2 | yes |
| GSM437175 | 105.17 | alive | cluster3 | <65 | male | 3 | 2 | no |
| GSM437176 | 11.76 | dead | cluster3 | <65 | male | 3 | 2 | yes |
| GSM437178 | 95.07 | alive | cluster3 | >=65 | female | 3 | 2 | no |
| GSM437180 | 27.51 | dead | cluster3 | <65 | male | 3 | 1 | yes |
| GSM437181 | 45.92 | dead | cluster3 | <65 | female | 3 | 2 | yes |
| GSM437184 | 6.11 | dead | cluster3 | >=65 | male | 3 | 3 | yes |
| GSM437187 | 76.6 | dead | cluster3 | <65 | male | 3 | 3 | yes |
| GSM437190 | 96.26 | alive | cluster3 | <65 | male | 3 | 2 | no |
| GSM437196 | 47.8 | dead | cluster3 | <65 | male | 3 | 3 | no |
| GSM437197 | 5.68 | dead | cluster3 | >=65 | female | 3 | 2 | yes |
| GSM437200 | 50.76 | dead | cluster3 | >=65 | male | 3 | 2 | yes |
| GSM437201 | 67.82 | dead | cluster3 | <65 | female | 3 | 2 | yes |
| GSM437202 | 70.84 | alive | cluster3 | <65 | male | 3 | 3 | no |
| GSM437203 | 50.59 | alive | cluster3 | >=65 | female | 3 | 3 | no |
| GSM437204 | 34.58 | dead | cluster3 | <65 | male | 3 | 2 | unknown |
| GSM437209 | 59.34 | alive | cluster3 | >=65 | male | 3 | 3 | no |
| GSM437210 | 54.54 | alive | cluster3 | >=65 | female | 3 | 2 | no |
| GSM437211 | 19.03 | dead | cluster3 | <65 | female | 3 | 3 | yes |
| GSM437212 | 26.26 | dead | cluster3 | >=65 | female | 3 | 3 | no |
| GSM437222 | 63.05 | alive | cluster3 | <65 | male | 3 | 2 | yes |
| GSM437223 | 49.24 | dead | cluster3 | >=65 | female | 3 | 2 | yes |
| GSM437225 | 31.36 | alive | cluster3 | >=65 | female | 3 | 2 | no |
| GSM437226 | 40.3 | alive | cluster3 | <65 | female | 3 | 2 | no |
| GSM437229 | 19.98 | alive | cluster3 | >=65 | female | 3 | 3 | no |
| GSM437234 | 5.32 | dead | cluster3 | <65 | male | 4 | 2 | unknown |
| GSM437235 | 2.36 | dead | cluster3 | >=65 | male | 4 | 3 | unknown |
| GSM437239 | 4.96 | dead | cluster3 | >=65 | male | 4 | 2 | unknown |
| GSM437250 | 5.72 | dead | cluster3 | <65 | male | 4 | 3 | unknown |
| GSM437257 | 23.07 | dead | cluster3 | <65 | female | 4 | 2 | unknown |
| GSM437263 | 14.56 | dead | cluster3 | <65 | male | 4 | 2 | unknown |
| GSM437265 | 8.94 | dead | cluster3 | >=65 | male | 4 | 2 | no |
| GSM437267 | 2.07 | dead | cluster3 | >=65 | female | 4 | 3 | unknown |
| GSM437272 | 46.29041096 | dead | cluster3 | >=65 | male | 2 | 2 | no |
| GSM437278 | 55.85753425 | alive | cluster3 | >=65 | female | 2 | 2 | no |
| GSM437297 | 7.824657534 | dead | cluster3 | >=65 | female | 3 | 3 | no |
| GSM437301 | 70.84931507 | alive | cluster3 | <65 | male | 4 | 2 | yes |
| GSM437306 | 21.63287671 | dead | cluster3 | >=65 | male | 2 | 2 | no |
| GSM437307 | 33.10684932 | dead | cluster3 | >=65 | female | 2 | 2 | no |
| GSM437308 | 69.17260274 | alive | cluster3 | >=65 | female | 4 | 2 | unknown |
| GSM437314 | 13.84109589 | dead | cluster3 | >=65 | female | 3 | 2 | yes |
| GSM437315 | 70.45479452 | alive | cluster3 | <65 | female | 3 | 2 | no |
| GSM437316 | 36 | dead | cluster3 | >=65 | female | 4 | 2 | unknown |
| GSM437317 | 1.545205479 | dead | cluster3 | >=65 | female | 4 | 3 | unknown |
| GSM437318 | 17.65479452 | dead | cluster3 | <65 | male | 4 | 2 | unknown |
| GSM437319 | 12.65753425 | dead | cluster3 | <65 | female | 4 | 2 | unknown |

**Table S8 Detailed gene list of the 600 hypoxia-related differentially expressed genes.**

| CFH | CSF2RB | JAZF1 | ARMCX1 | ENPP2 | NDUFA4L2 |
| --- | --- | --- | --- | --- | --- |
| WISP1 | ARHGEF6 | EVI2B | AKAP12 | SSPN | IL1B |
| COL5A2 | CNRIP1 | COL12A1 | CD14 | CYS1 | ACHE |
| MAF | DCN | GALNT15 | ATP2B4 | SCG2 | KRT7 |
| CCDC80 | SPARCL1 | CD4 | SLIT2 | KIAA1644 | KRT17 |
| CLMP | CLIP4 | COL4A1 | LMOD1 | HAND2 | TUBB2B |
| CALD1 | ADAMTS1 | TAGLN | CECR1 | BVES | SLC4A11 |
| COLEC12 | THBS2 | SLC9A9 | STOM | PLAU | HPGD |
| FAM49A | DNAJB5 | NAP1L3 | SLIT3 | RGAG4 | TNFRSF17 |
| FLI1 | LCP1 | ARHGEF25 | LY86 | NDN | IGFL2 |
| RASSF2 | A2M | CCL21 | AQP9 | KAL1 | RAI2 |
| INHBA | OLFML1 | LOXL2 | TAGAP | MS4A6A | S100A9 |
| FERMT2 | TNS1 | TEK | PRELP | SRPX | MMP7 |
| GPNMB | TSPYL5 | HAPLN3 | IL16 | FCN1 | FKBP10 |
| PLEKHO1 | RGS2 | COL11A1 | NCF1 | PPAPDC1A | GJB4 |
| EHD2 | GNG2 | PTPRC | HLA-DQA1 | MEF2C | SEPP1 |
| SULF1 | BTK | PDLIM3 | NOTCH3 | MMP9 | CST2 |
| COL1A2 | NEXN | HSPB8 | PTGIS | CFL2 | FGFR2 |
| ZEB2 | ADAMTS12 | CILP | GIMAP7 | TLR7 | KLK10 |
| FAM129A | ADAMTS4 | ZEB1 | GPR183 | RNF150 | IL11 |
| DDR2 | IL10RA | CLIC2 | CDKN2B | IL6ST | LY6E |
| VCAN | OLR1 | CLIP3 | FLNA | SPP1 | PLA2G2A |
| MSRB3 | APBB1IP | JAM2 | HTRA3 | RSPO3 | DUSP4 |
| COL8A1 | TMEM200B | DACT3 | TREM1 | PDE2A | CFD |
| ADAMTS2 | TMEM140 | COL14A1 | TCEAL7 | SPON2 | ADRA2A |
| SYNC | BOC | SERPINE1 | SLC22A17 | MMRN1 | IGJ |
| ADAM12 | GIMAP8 | MFAP5 | HLA-DOA | HLA-DPB1 | CLC |
| COL5A1 | SPG20 | AIF1 | EVI2A | RGS16 | CPE |
| SPARC | THY1 | PLN | ITGAL | TMTC1 | MT1M |
| JAM3 | SFRP2 | MRVI1 | MPEG1 | HLA-DPA1 | CST1 |
| LSP1 | ARHGAP30 | STC1 | CXCL12 | PRKCB | MMP3 |
| HCLS1 | NR3C1 | MS4A4A | CALU | TWIST1 | ANPEP |
| S1PR1 | CMKLR1 | CYBRD1 | C10orf128 | CITED2 | CXCL5 |
| FAP | MRGPRF | CD37 | SASH3 | CHRNA3 | KRT6A |
| FBLN5 | EFEMP1 | CD180 | SDPR | NPR1 | SELENBP1 |
| MMP14 | CSF1R | SHE | FBLN1 | CNN1 | C10orf99 |
| CDH11 | HCK | SGCE | SIGLEC1 | EDIL3 | SERPINB5 |
| AOC3 | GIMAP6 | MYLK | C1QA | SGK1 | MT1E |
| GIMAP4 | PDPN | CLEC5A | MEIS1 | P2RY8 | GCNT3 |
| OLFML2B | DOCK2 | MS4A7 | MYL9 | CRYAB | TACSTD2 |
| AMOTL1 | MGP | GIMAP1 | FXYD6 | P3H1 | CD177 |
| BGN | WAS | ACTA2 | MAP1B | C1QC | SAA1 |
| C20orf194 | CD53 | ITGA11 | DAAM2 | CADM3 | CES1 |
| GAS7 | GYPC | SPHK1 | MFAP4 | PIK3CG | KRT6B |
| DPYD | SSC5D | MRC1 | FYB | TMEM47 | PPBP |
| CTHRC1 | FBLN2 | SLC11A1 | PPP1R16B | LYNX1 | DPEP1 |
| COL1A1 | COL10A1 | DOK2 | TNXB | C1QB | KLK6 |
| AXL | LYVE1 | KANK2 | IKZF1 | FHL1 | XXbac-BPG32J3.19 |
| CD163 | NCKAP1L | VSIG4 | CPXM2 | GLIPR2 | CEL |
| CAV1 | RCSD1 | LY96 | CCDC69 | RASL12 | REG3A |
| TMOD1 | PJA2 | BHLHE40 | FLNC | CPVL | EDNRB |
| FGL2 | HCST | C16orf54 | CDR2L | CHI3L1 | APOD |
| SYNPO | RARRES2 | PODNL1 | NTN1 | LAMC2 | HSPB6 |
| DUSP1 | CCL13 | P2RY13 | PSD | FOXS1 | FAS |
| SFRP4 | TRANK1 | CBX6 | ZNF304 | CTSW | LXN |
| KCNMB1 | PDE5A | SLAMF7 | CCL23 | SECTM1 | ITGB7 |
| MEOX1 | EMP1 | PLSCR4 | LRRN2 | SORBS1 | FAM46C |
| LRRC15 | IGFBP6 | CD209 | LAX1 | MXD1 | DERL3 |
| SLC37A2 | CD69 | ANGPTL1 | NGFR | VIP | TP53INP2 |
| FAM107A | STAB1 | EDNRA | IL6R | TMCC3 | IGLL5 |
| DPT | LMO2 | MFAP2 | MATN3 | MT2A | MMP1 |
| CHRDL1 | TSPAN11 | CTSG | SGCA | CD22 | SCARA3 |
| PRKAR2B | SYNPO2 | KDELC1 | WNT9A | CD79A | C16orf62 |
| JPH2 | PLA2G5 | KCNMA1 | ASB2 | PI16 | PLS3 |
| SALL2 | FLVCR2 | CD96 | TPSB2 | ADAM28 | RNF125 |
| FBXO32 | RASGRP2 | SFRP1 | CXCL10 | DES | PGM5 |
| CCR2 | SERPINH1 | SVIL | ACTG2 | EBI3 | POU2AF1 |
| F13A1 | DIO2 | SAMD9 | FOLR2 | OLFML3 | KITLG |
| PDGFRA | C10orf54 | PTGS1 | ARRDC4 | LIMS2 | CR2 |
| HMOX1 | SVEP1 | UCHL1 | PCSK5 | GRP | EGFL6 |
| CPA3 | BMP6 | CALB2 | CPNE8 | FAM46A | OSBPL1A |
| RGL1 | C8orf88 | ADAMTSL4 | CLDN5 | CLU | GABARAP |
| EML1 | CCL8 | PPAP2A | CCL5 | LPAR1 | MAOB |
| PTN | APBB1 | PPAP2B | CPED1 | MMP11 | ST6GALNAC6 |
| SEMA3G | PLCG2 | MMP12 | OMD | STMN2 | GZMA |
| CD36 | PAMR1 | EPB41L3 | TCEAL2 | CD1C | PLIN4 |
| KCTD12 | P2RY14 | OSM | CD7 | IRF4 | NDRG1 |
| RERG | TPM2 | RAB27A | TSPAN2 | TBC1D9 | FOXF2 |
| ACKR1 | POPDC2 | IL1RN | ADH1B | PAG1 | HAND1 |
| PPP1R3C | MAP4K1 | CD3E | KAT2B | IBSP | MZB1 |
| C7 | GSN | OLFM1 | DDX60 | CCL11 | MS4A1 |
| CPQ | GNAO1 | ADAM33 | FAIM3 | CXCL8 | FADS2 |
| CPXM1 | GAP43 | TIMP1 | AQP1 | SHISA3 | BMP2 |
| CAP2 | WNT2 | CD27 | ICAM3 | FDCSP | RARRES1 |
| PALM | THBS4 | UST | COX7A1 | AC131263.1 | FRMD5 |
| HSPB7 | EPAS1 | CXCL13 | PPP1R12B | WNT5A | KLRB1 |
| COMP | ANTXR2 | ITM2A | ADAMDEC1 | MEGF6 | PTPRR |
| BEX4 | CACNA1H | UBTD1 | MAMDC2 | KIAA1211 | L1CAM |
| MAB21L2 | TCN2 | AOX1 | BHLHE41 | CLEC3B | SNCG |
| RBPMS2 | TXNIP | AHNAK | CASQ2 | SLCO2A1 | LEF1 |
| FSTL3 | FABP4 | GPX3 | OGN | GZMM | IFI6 |
| RCAN2 | RSAD2 | CD79B | CP | FAM46B | CXCL11 |
| RGMA | FOXF1 | SNAI1 | PCDHGC3 | ITGA7 | RP11-812E19.9 |
| TCF21 | CD52 | LRRN4CL | FAM107B | SYNM | CDIP1 |
| ABI3BP | FNBP1 | KIF26B | S100B | TMEM132A | PTGER2 |
| CD48 | P2RY10 | PTGDS | ITIH5 | PDZRN3 | PDE3A |
| KLF9 | CCL19 | GPR34 | COL7A1 | SHISA2 | CXCL6 |
| MYH11 | TPSAB1 | CLEC10A | CPM | UBD | KRT80 |
| RTN1 | AMICA1 | SLC2A5 | PLCL2 | GREM2 | FOSL1 |
| SETBP1 | SPON1 | PLA2G2D | MYOCD | FADS1 | KIT |

**Table S9 The optimal model of LASSO Cox regression.**

| Gene | Coefficient |
| --- | --- |
| CD177 | -0.140180722 |
| CP | 0.045369908 |
| RGS16 | 0.006073836 |
| PGM5 | 0.01320997 |
| SNAI1 | 0.206420162 |
| CALB2 | 0.010108696 |
| OSBPL1A | 0.041273069 |
| CDR2L | 0.043393759 |
| FRMD5 | 0.011784582 |
| FSTL3 | 0.095763476 |
| TUBB2B | 0.069419349 |

**Table S10 HRI risk scores of 348 patients in TCGA-COAD cohort.**

| id | OS time(year) | OS status | CD177 | CP | RGS16 | PGM5 | SNAI1 | CALB2 | OSBPL1A | CDR2L | FRMD5 | FSTL3 | TUBB2B | riskScore | risk |
| --- | --- | --- | --- | --- | --- | --- | --- | --- | --- | --- | --- | --- | --- | --- | --- |
| TCGA-G4-6294 | 2.349166667 | 1 | 0.911558 | 0.113205 | 3.644866 | 0.543135 | 2.545811 | 0.097533 | 1.939602 | 1.943518 | 0.602056 | 3.319359 | 0.547414 | 0.960518 | low |
| TCGA-AZ-4313 | 6.324166667 | 0 | 4.997611 | 0.169674 | 2.838552 | 0.575775 | 2.577421 | 1.650189 | 0.562415 | 5.116153 | 1.797502 | 5.753883 | 0.871888 | 0.758631 | low |
| TCGA-CA-5256 | 1.0375 | 0 | 0.132894 | 0.482244 | 3.146528 | 2.33975 | 2.324164 | 0.19119 | 1.016364 | 2.676172 | 1.163502 | 2.978868 | 0.731146 | 1.042768 | low |
| TCGA-G4-6317 | 2.9975 | 0 | 0.423365 | 0.049732 | 3.630756 | 0.243402 | 4.432338 | 0.808423 | 2.65181 | 3.894375 | 1.556769 | 3.062662 | 0.513644 | 1.517007 | high |
| TCGA-AA-3488 | 0.419166667 | 1 | 4.07511 | 0.395435 | 4.730061 | 1.806026 | 4.17773 | 0.110322 | 2.635296 | 4.580509 | 2.080982 | 3.959232 | 1.034666 | 1.14579 | low |
| TCGA-A6-5661 | 2.7925 | 0 | 0.625598 | 0.282259 | 3.47318 | 0.106575 | 2.095247 | 0 | 1.655822 | 3.277203 | 2.019199 | 0.972377 | 0 | 0.707578 | low |
| TCGA-G4-6307 | 4.5825 | 0 | 1.680241 | 0.090558 | 4.606047 | 2.192063 | 3.643145 | 0.065237 | 1.011749 | 4.198939 | 1.866546 | 2.618177 | 1.162668 | 1.155582 | low |
| TCGA-A6-5656 | 2.74 | 0 | 0.527793 | 1.009952 | 3.090944 | 2.470053 | 1.762594 | 0.190328 | 2.405745 | 1.840335 | 2.150596 | 2.525067 | 0.456465 | 0.866989 | low |
| TCGA-DM-A28M | 7.925833333 | 0 | 0.989202 | 0.076244 | 4.276569 | 0.169172 | 2.906398 | 1.841315 | 0.330857 | 3.012036 | 1.549287 | 2.485791 | 2.210845 | 1.065695 | low |
| TCGA-AZ-4614 | 0.470833333 | 1 | 1.345518 | 1.119154 | 3.750338 | 0.236864 | 3.30146 | 5.688542 | 4.699665 | 4.847868 | 2.795318 | 4.689411 | 0.777139 | 1.567361 | high |
| TCGA-QG-A5YX | 2.745833333 | 0 | 4.196213 | 0.080684 | 2.66348 | 0.522006 | 1.969301 | 0.098159 | 2.397061 | 3.713972 | 1.395226 | 1.884753 | 1.385256 | 0.399195 | low |
| TCGA-A6-6650 | 1.716666667 | 0 | 1.509978 | 0.288467 | 3.834105 | 0.558198 | 1.77024 | 0 | 1.943401 | 1.921239 | 1.040195 | 3.568453 | 0.640056 | 0.75949 | low |
| TCGA-NH-A50T | 1.514166667 | 0 | 0.942743 | 0.137807 | 3.744184 | 0.277513 | 1.780573 | 0.944774 | 3.044526 | 3.315648 | 1.568284 | 5.067574 | 1.054402 | 1.124103 | low |
| TCGA-CM-5862 | 0.419166667 | 1 | 0.442437 | 0.093703 | 4.610845 | 3.738879 | 3.634838 | 0.309849 | 2.953094 | 3.271109 | 2.029769 | 2.972523 | 0.771173 | 1.399005 | high |
| TCGA-DM-A28G | 5.061666667 | 1 | 0.44191 | 0.086007 | 3.391184 | 2.142732 | 2.471345 | 0.33705 | 1.904259 | 3.29037 | 1.736063 | 2.500328 | 2.551334 | 1.162787 | low |
| TCGA-5M-AATE | 3.285 | 0 | 0.364273 | 0.069332 | 4.309789 | 2.487256 | 3.555633 | 0.882567 | 3.606745 | 3.298279 | 1.691058 | 4.280237 | 0.40863 | 1.504163 | high |
| TCGA-DM-A28H | 9.748333333 | 0 | 0.672752 | 0.061625 | 4.583778 | 0.904443 | 4.285941 | 0.391336 | 1.77448 | 3.769389 | 1.421157 | 2.386244 | 0.394442 | 1.346389 | low |
| TCGA-CM-4747 | 2.083333333 | 0 | 0.70565 | 0.22429 | 4.059614 | 3.371397 | 2.834906 | 2.202592 | 1.785622 | 3.310976 | 0.556994 | 5.065067 | 2.249626 | 1.453052 | high |
| TCGA-AZ-4315 | 4.861666667 | 0 | 0.877821 | 0.193533 | 3.142395 | 0.735467 | 2.832624 | 2.308548 | 1.752268 | 2.466721 | 2.420321 | 4.240822 | 0.690462 | 1.184507 | low |
| TCGA-A6-2672 | 3.885 | 0 | 1.914122 | 1.300562 | 3.82745 | 0.347081 | 2.805249 | 0.885295 | 3.049206 | 3.47134 | 3.326603 | 2.697535 | 0.974103 | 1.048159 | low |
| TCGA-AA-A01I | 2.581666667 | 0 | 0.282706 | 0.555784 | 3.563051 | 0.473874 | 1.998949 | 0.145313 | 3.357001 | 2.022272 | 2.384822 | 2.613101 | 0.898152 | 0.99458 | low |
| TCGA-AA-A01Z | 3.0825 | 0 | 0.422558 | 0.876573 | 4.221165 | 0.581731 | 3.285387 | 2.501936 | 0.858694 | 4.15124 | 2.39536 | 3.847425 | 1.893532 | 1.461018 | high |
| TCGA-AA-3519 | 0.755833333 | 0 | 3.040687 | 0.337023 | 3.418271 | 2.796274 | 3.037744 | 0.460263 | 1.945625 | 2.489339 | 0.35417 | 2.995806 | 0.594991 | 0.79914 | low |
| TCGA-A6-5659 | 2.535 | 0 | 0.043054 | 0.266023 | 2.656898 | 1.594568 | 2.097716 | 0 | 2.162043 | 0.687631 | 0.869075 | 1.323552 | 0.27165 | 0.751167 | low |
| TCGA-DM-A0X9 | 9.9675 | 0 | 0.151237 | 0.105371 | 4.648452 | 1.516578 | 3.178997 | 0.158253 | 3.081062 | 2.880755 | 1.780228 | 3.65093 | 1.117607 | 1.390017 | high |
| TCGA-F4-6808 | 2.803333333 | 0 | 3.709693 | 0.154533 | 4.502065 | 0.458004 | 3.666509 | 0.387628 | 3.714191 | 3.86732 | 2.243276 | 3.473175 | 1.990464 | 1.099468 | low |
| TCGA-AA-A017 | 1.250833333 | 0 | 0.881602 | 0.725558 | 3.161891 | 1.291873 | 2.994883 | 0.983101 | 1.933541 | 3.189389 | 0.690297 | 4.272319 | 0.728613 | 1.259797 | low |
| TCGA-AA-A01X | 2.165833333 | 0 | 3.780485 | 0.662081 | 4.890252 | 3.130006 | 4.295401 | 2.030645 | 3.218359 | 3.346282 | 2.051961 | 4.286394 | 3.725874 | 1.44967 | high |
| TCGA-D5-6532 | 1.519166667 | 0 | 1.241083 | 0.027831 | 5.277902 | 1.716741 | 3.724893 | 1.223544 | 2.473247 | 2.82147 | 0.800305 | 3.16849 | 0.90647 | 1.26358 | low |
| TCGA-DM-A0XD | 2.034166667 | 1 | 0.766934 | 0.16743 | 3.533141 | 0.28667 | 3.738188 | 3.587753 | 2.711537 | 4.212781 | 2.922427 | 5.07302 | 0.832873 | 1.606027 | high |
| TCGA-AA-3844 | 1.2425 | 0 | 2.340117 | 0.462912 | 2.977371 | 1.180881 | 3.012478 | 0 | 1.852757 | 2.47954 | 1.436122 | 3.042634 | 0.15623 | 0.851691 | low |
| TCGA-AY-A71X | 1.61 | 0 | 0.966999 | 0.167014 | 2.291171 | 1.101345 | 1.971265 | 0.121195 | 3.511712 | 3.875283 | 1.354629 | 3.22513 | 1.6044 | 1.057914 | low |
| TCGA-DM-A28C | 6.775833333 | 1 | 2.575126 | 0.049601 | 2.725779 | 1.909275 | 3.413767 | 0.124215 | 1.712743 | 2.091617 | 0.682782 | 2.336236 | 4.626792 | 1.103385 | low |
| TCGA-AA-3560 | 1.664166667 | 0 | 5.605961 | 0.984013 | 3.820393 | 1.742539 | 2.565831 | 1.560096 | 0.869791 | 2.485136 | 1.107238 | 3.125235 | 1.531458 | 0.412813 | low |
| TCGA-G4-6304 | 4.465 | 0 | 5.948839 | 0.025526 | 4.793036 | 3.692181 | 3.032784 | 2.482459 | 3.26615 | 3.476214 | 1.69795 | 2.880173 | 2.415031 | 0.645378 | low |
| TCGA-CM-5864 | 1.250833333 | 0 | 0.191025 | 0.395554 | 3.354398 | 1.96222 | 2.667953 | 0.603868 | 3.196229 | 2.454452 | 2.165043 | 3.070528 | 1.791237 | 1.276618 | low |
| TCGA-AA-A01V | 0.085 | 0 | 0.466386 | 0.849036 | 4.4749 | 0.519845 | 2.704816 | 0 | 3.143734 | 4.112479 | 2.598225 | 2.957451 | 3.278123 | 1.415125 | high |
| TCGA-AZ-6599 | 0.564166667 | 1 | 0.951604 | 0.263165 | 2.619029 | 3.7299 | 1.494926 | 0.598731 | 3.129801 | 5.038938 | 0.391349 | 1.367184 | 1.022506 | 0.812713 | low |
| TCGA-AA-A01Q | 0.085 | 0 | 0.33144 | 0.235193 | 2.739358 | 0.213463 | 2.533297 | 0.088433 | 2.983914 | 3.658802 | 0.140286 | 3.154855 | 0.622694 | 1.136409 | low |
| TCGA-AY-A54L | 1.4375 | 0 | 0.472072 | 0.031559 | 4.173289 | 2.080764 | 3.353245 | 0.52823 | 3.022748 | 3.553217 | 2.063626 | 2.19529 | 2.798484 | 1.39337 | high |
| TCGA-AD-6888 | 1.2925 | 1 | 3.027991 | 0.68298 | 4.554775 | 1.325643 | 3.169317 | 1.007628 | 3.294331 | 4.10636 | 3.691327 | 3.360788 | 1.173192 | 1.077035 | low |
| TCGA-AA-3514 | 0.085 | 0 | 1.651709 | 0.237084 | 2.918853 | 3.934702 | 3.552726 | 0.952509 | 1.506413 | 3.859252 | 1.328186 | 5.261567 | 2.925891 | 1.544181 | high |
| TCGA-SS-A7HO | 5.0075 | 0 | 1.773315 | 1.306084 | 4.55023 | 1.539461 | 4.722868 | 0.059056 | 1.279838 | 3.554106 | 0.664492 | 2.686156 | 4.044644 | 1.58703 | high |
| TCGA-AA-A00L | 3.1675 | 0 | 1.718787 | 0.181568 | 3.277555 | 0.293828 | 3.385681 | 1.20997 | 0.623109 | 2.461193 | 0.655004 | 3.352224 | 0.327519 | 0.986184 | low |
| TCGA-AA-A01S | 0.085 | 0 | 0.066196 | 0.023035 | 3.92378 | 1.604026 | 3.939958 | 0.42736 | 2.197362 | 5.046457 | 1.258482 | 2.409295 | 1.086769 | 1.485067 | high |
| TCGA-AA-A02K | 1.165833333 | 1 | 1.497063 | 0.067004 | 4.304663 | 0.214295 | 3.251366 | 0.551415 | 2.54353 | 3.400085 | 1.838885 | 4.541574 | 1.059374 | 1.281529 | low |
| TCGA-DM-A1D9 | 11.69 | 0 | 0.768558 | 0.053134 | 4.39856 | 2.132843 | 2.927874 | 0.451411 | 2.758399 | 3.521478 | 2.365822 | 2.828343 | 3.399565 | 1.359885 | high |
| TCGA-A6-2683 | 1.38 | 1 | 0.291491 | 0 | 3.106705 | 3.035892 | 3.570161 | 0.655754 | 1.66968 | 3.590491 | 0.809454 | 5.589155 | 2.381663 | 1.696522 | high |
| TCGA-DM-A1DA | 0.624166667 | 1 | 0.070721 | 0.01647 | 5.74513 | 2.935432 | 4.187123 | 1.991295 | 4.009431 | 4.487222 | 2.712794 | 4.462844 | 0.080346 | 1.774065 | high |
| TCGA-DM-A28E | 9.986666667 | 0 | 0.235586 | 0.012478 | 3.090961 | 0.27901 | 2.462267 | 0.495044 | 2.392432 | 1.924497 | 0.214524 | 2.683339 | 0.759817 | 0.997761 | low |
| TCGA-4N-A93T | 0.4 | 0 | 0.208636 | 0.156128 | 2.401452 | 0.71704 | 2.240592 | 2.259015 | 2.151915 | 3.167778 | 0.594334 | 4.260942 | 0.876691 | 1.189418 | low |
| TCGA-D5-6538 | 1.426666667 | 0 | 0.597039 | 0.296819 | 3.51176 | 1.787228 | 2.950384 | 0.713598 | 2.435867 | 3.838569 | 2.343163 | 3.573837 | 1.848118 | 1.356202 | low |
| TCGA-AA-A02Y | 3.329166667 | 0 | 2.075065 | 0.255338 | 3.888348 | 0.282693 | 2.232077 | 0.11877 | 2.822151 | 2.861548 | 0.995283 | 2.068381 | 0.865102 | 0.72051 | low |
| TCGA-AZ-6608 | 0.161666667 | 1 | 0.202869 | 0.052372 | 3.782229 | 0.149214 | 3.789761 | 0.866778 | 2.244548 | 3.752677 | 1.745865 | 3.408935 | 1.057865 | 1.465871 | high |
| TCGA-AA-A01T | 2.751666667 | 0 | 3.405404 | 0 | 2.851094 | 0.151602 | 3.582633 | 0.402836 | 0.67656 | 2.77741 | 0.644983 | 3.764424 | 0.700484 | 0.850716 | low |
| TCGA-AA-A03J | 3.410833333 | 0 | 3.640461 | 0.44422 | 3.136554 | 0.758441 | 3.679673 | 2.248424 | 2.383066 | 3.280275 | 0.934397 | 5.234113 | 0.752909 | 1.126404 | low |
| TCGA-AA-A004 | 1.160833333 | 0 | 4.34258 | 1.174708 | 3.234802 | 1.211115 | 3.527583 | 1.030475 | 2.400411 | 4.98336 | 3.077199 | 5.458966 | 1.988271 | 1.231155 | low |
| TCGA-AZ-5407 | 7.345 | 0 | 1.663006 | 0.296941 | 3.736827 | 1.217083 | 2.49778 | 0.42512 | 4.352923 | 4.579986 | 2.237887 | 3.272774 | 1.841548 | 1.18504 | low |
| TCGA-AA-3548 | 2.830833333 | 0 | 1.328576 | 0.434666 | 3.490042 | 1.408434 | 2.904275 | 2.087623 | 2.269184 | 3.89709 | 0.61697 | 4.666243 | 1.712328 | 1.329648 | low |
| TCGA-DM-A1DB | 3.69 | 1 | 2.089417 | 0.043103 | 4.264208 | 1.706873 | 3.65552 | 0.831528 | 2.83813 | 3.097468 | 1.364145 | 2.741504 | 0.664426 | 1.096771 | low |
| TCGA-AA-A01F | 2.666666667 | 0 | 0.067453 | 0.113752 | 4.151401 | 0.154251 | 2.068715 | 0.826383 | 3.138262 | 3.195193 | 0.988545 | 3.643866 | 0.408578 | 1.115475 | low |
| TCGA-D5-5540 | 4.67 | 0 | 0.089242 | 0.793084 | 4.167137 | 1.22847 | 4.798841 | 1.523181 | 2.632814 | 2.183636 | 0.187483 | 3.080032 | 0.661677 | 1.617503 | high |
| TCGA-AA-3861 | 2.5025 | 0 | 1.659936 | 0.454405 | 3.36406 | 1.064338 | 1.61978 | 0.552374 | 3.382385 | 3.352137 | 1.636982 | 2.858252 | 0.367012 | 0.765905 | low |
| TCGA-AA-3531 | 2.833333333 | 0 | 3.133861 | 0.742239 | 2.693353 | 0.984952 | 4.502925 | 0.51299 | 2.114821 | 2.72335 | 0.545852 | 4.672308 | 1.317014 | 1.309176 | low |
| TCGA-AA-3561 | 1.160833333 | 0 | 0.1983 | 0.182298 | 4.559081 | 0.394282 | 2.659599 | 0.444295 | 2.06523 | 2.426833 | 1.46848 | 2.942151 | 0.533964 | 1.09353 | low |
| TCGA-CM-4744 | 1.6675 | 0 | 0.251322 | 0.414335 | 4.421802 | 0.764332 | 2.387994 | 1.694098 | 3.466784 | 4.586735 | 3.391783 | 5.325697 | 1.344925 | 1.516039 | high |
| TCGA-AA-3941 | 1.998333333 | 0 | 0.540999 | 0.187303 | 2.248175 | 1.531002 | 2.925838 | 2.117987 | 2.738533 | 4.185764 | 2.910077 | 4.595301 | 3.260993 | 1.587298 | high |
| TCGA-A6-6652 | 2.055833333 | 0 | 0.215726 | 0.064984 | 4.183854 | 1.698324 | 2.852119 | 0.306689 | 3.863944 | 3.095622 | 1.63934 | 4.312417 | 0.661945 | 1.38444 | high |
| TCGA-DM-A1HA | 10.95083333 | 0 | 0.233876 | 0.042874 | 1.71792 | 0.854055 | 4.744834 | 5.744917 | 2.184547 | 3.194283 | 2.00663 | 5.018181 | 1.551356 | 1.869055 | high |
| TCGA-AA-3542 | 1.081666667 | 0 | 1.75489 | 0.057563 | 2.984401 | 0.286841 | 3.873015 | 1.20662 | 0.332459 | 3.715542 | 1.415178 | 3.842015 | 0.910643 | 1.212963 | low |
| TCGA-QL-A97D | 1.823333333 | 0 | 2.264766 | 0.207255 | 3.590119 | 0.532988 | 3.314841 | 0.287616 | 0.787398 | 2.357904 | 0.501633 | 3.631658 | 0.192456 | 0.909799 | low |
| TCGA-DM-A1D4 | 7.7225 | 1 | 0.656672 | 0.106809 | 3.433133 | 0.696144 | 2.697513 | 0.033533 | 3.733957 | 2.490769 | 1.888908 | 2.002445 | 1.002815 | 1.045833 | low |
| TCGA-RU-A8FL | 3.2225 | 0 | 0.462629 | 0.034636 | 3.479406 | 0.089039 | 2.545196 | 0.392097 | 0.261706 | 4.669583 | 2.646178 | 1.922202 | 0.997525 | 0.986313 | low |
| TCGA-AZ-4308 | 9.1 | 0 | 4.079541 | 0.076349 | 4.012513 | 1.656996 | 3.58195 | 2.397408 | 1.491778 | 4.087289 | 1.417767 | 5.357471 | 0.680249 | 1.057386 | low |
| TCGA-AA-3972 | 4.245833333 | 0 | 3.115369 | 0.101644 | 3.464573 | 2.067444 | 2.616608 | 1.524247 | 1.05577 | 2.82013 | 1.614917 | 3.757942 | 0 | 0.716636 | low |
| TCGA-AA-3848 | 0.8375 | 1 | 3.350274 | 0.442432 | 4.368164 | 1.143361 | 4.449929 | 0.909667 | 1.482988 | 3.539999 | 0.558886 | 4.970614 | 2.902819 | 1.418738 | high |
| TCGA-AA-3667 | 1.165833333 | 0 | 2.227973 | 0.160272 | 2.930332 | 1.145434 | 4.067143 | 1.004873 | 0.911981 | 3.574069 | 1.256551 | 5.040077 | 1.119151 | 1.345467 | low |
| TCGA-AA-A00W | 1.248333333 | 0 | 1.348426 | 0.243043 | 3.72875 | 0.356624 | 2.788901 | 0 | 2.411522 | 2.636036 | 1.584463 | 3.238341 | 0.687214 | 1.015459 | low |
| TCGA-CM-4746 | 3.0825 | 0 | 0.259964 | 0.139503 | 4.185325 | 3.344859 | 2.698613 | 0.283896 | 1.636159 | 3.982167 | 1.230685 | 4.363936 | 0.644036 | 1.316859 | low |
| TCGA-AA-3502 | 2.915833333 | 0 | 0.334463 | 0.721081 | 2.44261 | 2.095316 | 1.403198 | 0.56458 | 3.606989 | 3.367264 | 1.094125 | 2.90146 | 2.094769 | 1.054856 | low |
| TCGA-DM-A1D0 | 10.87916667 | 0 | 0.202128 | 0.0866 | 2.734821 | 0.685101 | 2.351889 | 1.691871 | 2.201948 | 3.074652 | 0.873856 | 3.027287 | 0.49718 | 1.062853 | low |
| TCGA-NH-A8F7 | 1.486666667 | 0 | 0.273291 | 0.087539 | 2.123712 | 4.004432 | 2.475951 | 1.272975 | 3.159109 | 1.96843 | 0.377344 | 3.648913 | 0.776723 | 1.179016 | low |
| TCGA-A6-3807 | 2.885833333 | 0 | 1.485281 | 1.286582 | 4.845068 | 1.025358 | 3.981351 | 2.547343 | 3.060215 | 3.947346 | 1.648422 | 5.103885 | 1.391845 | 1.643126 | high |
| TCGA-AA-3846 | 1.418333333 | 0 | 5.027342 | 0.404916 | 4.917484 | 0.614065 | 3.304532 | 1.159561 | 1.287293 | 2.570422 | 2.620466 | 3.245312 | 0.504894 | 0.586842 | low |
| TCGA-QG-A5Z2 | 2.605833333 | 0 | 4.129513 | 0.291021 | 4.127062 | 0.599229 | 1.766095 | 0.787777 | 3.021789 | 3.907854 | 1.065918 | 2.879813 | 0.843591 | 0.481028 | low |
| TCGA-CM-5341 | 2.42 | 0 | 1.53754 | 1.244819 | 4.30215 | 0.923926 | 2.669815 | 2.026508 | 2.365423 | 1.844389 | 1.346285 | 3.903622 | 6.118832 | 1.442987 | high |
| TCGA-AA-3662 | 0.503333333 | 0 | 3.716675 | 1.030785 | 3.463301 | 2.692242 | 3.705972 | 0.635995 | 3.765543 | 3.9473 | 2.170065 | 4.110289 | 2.131876 | 1.247663 | low |
| TCGA-A6-2678 | 3.520833333 | 0 | 3.907244 | 0.391187 | 4.58303 | 1.117744 | 3.183833 | 0.703063 | 2.075908 | 3.96278 | 2.295694 | 3.369526 | 0.686417 | 0.831965 | low |
| TCGA-AA-3976 | 2.165833333 | 0 | 2.025014 | 0.057242 | 5.186167 | 3.679274 | 3.312194 | 2.318544 | 2.120982 | 3.730814 | 2.6605 | 4.313264 | 0 | 1.199813 | low |
| TCGA-CA-5797 | 1.048333333 | 0 | 1.069895 | 0.170704 | 4.169281 | 4.334313 | 3.874455 | 1.971321 | 1.675834 | 3.666732 | 3.442713 | 4.558066 | 0.899358 | 1.527819 | high |
| TCGA-AA-A02H | 0.166666667 | 1 | 2.812583 | 0.363865 | 3.969053 | 2.967296 | 5.034733 | 0.987833 | 2.624324 | 3.35527 | 1.60958 | 5.360213 | 1.337049 | 1.613809 | high |
| TCGA-AA-3862 | 2.5025 | 0 | 3.172331 | 0.39021 | 4.324273 | 0.883638 | 2.763333 | 1.016883 | 1.918882 | 3.102914 | 0.658768 | 3.374719 | 0.398123 | 0.76405 | low |
| TCGA-CA-6716 | 1.015833333 | 0 | 0.452615 | 0.580706 | 4.420827 | 3.166945 | 2.902226 | 2.998913 | 2.390164 | 3.470565 | 2.397981 | 4.18597 | 5.457644 | 1.718217 | high |
| TCGA-D5-6535 | 1.259166667 | 0 | 1.305038 | 0.559696 | 5.805571 | 0.89683 | 4.024451 | 2.039352 | 3.504056 | 3.8563 | 1.97075 | 4.358632 | 0.930116 | 1.558057 | high |
| TCGA-A6-6137 | 2.255833333 | 0 | 1.549119 | 0.49725 | 3.483433 | 1.71583 | 3.301976 | 0.453131 | 2.667446 | 3.705481 | 1.562661 | 2.577209 | 0.623893 | 1.114819 | low |
| TCGA-AA-3986 | 1.5875 | 0 | 3.890053 | 2.21664 | 2.807139 | 3.121773 | 2.464706 | 1.554319 | 2.458071 | 2.542305 | 1.865356 | 3.620585 | 2.579428 | 0.897561 | low |
| TCGA-CM-6165 | 1.335833333 | 0 | 0.973362 | 0.885682 | 3.919403 | 2.495664 | 3.083902 | 1.747639 | 3.37869 | 2.85272 | 1.14993 | 4.086128 | 0.937482 | 1.347928 | low |
| TCGA-CM-4750 | 0.668333333 | 0 | 1.12992 | 0.70316 | 4.298417 | 0.834993 | 4.507362 | 2.814765 | 2.058282 | 4.224007 | 3.100597 | 4.967722 | 0.645688 | 1.694848 | high |
| TCGA-AA-3520 | 2.000833333 | 0 | 3.42589 | 0.620105 | 5.102733 | 1.609625 | 4.639585 | 2.221524 | 2.507139 | 3.66157 | 1.468065 | 4.566425 | 0.895138 | 1.359411 | high |
| TCGA-G4-6625 | 7.643333333 | 0 | 6.359605 | 1.512542 | 4.648742 | 3.095151 | 3.692547 | 2.449775 | 3.250856 | 3.296709 | 0.920926 | 4.062006 | 1.933806 | 0.84455 | low |
| TCGA-AA-A02E | 0.246666667 | 1 | 1.008809 | 0.316069 | 4.093916 | 4.17519 | 3.796574 | 0.232527 | 2.836864 | 3.236196 | 2.622057 | 4.622453 | 2.801796 | 1.664562 | high |
| TCGA-AA-3710 | 2.2475 | 0 | 2.649777 | 1.502114 | 4.051791 | 1.473425 | 3.833085 | 0.785031 | 3.733686 | 5.361103 | 3.774359 | 5.069951 | 1.288855 | 1.546144 | high |
| TCGA-G4-6320 | 2.200833333 | 0 | 0.673209 | 0.118788 | 2.225422 | 0.970222 | 2.346327 | 0.351358 | 3.039163 | 4.73152 | 1.045602 | 3.370318 | 0.54969 | 1.129221 | low |
| TCGA-AA-A00Z | 1.831666667 | 0 | 6.074406 | 0.844651 | 2.905675 | 2.470526 | 3.724385 | 0 | 2.756167 | 4.073332 | 0.523206 | 3.746268 | 2.401843 | 0.828048 | low |
| TCGA-CM-6674 | 1.078333333 | 0 | 1.763912 | 0.601779 | 4.994688 | 2.641552 | 3.625867 | 1.924446 | 3.509326 | 5.106644 | 2.187867 | 4.221636 | 1.383468 | 1.505712 | high |
| TCGA-A6-2680 | 2.924166667 | 0 | 2.994207 | 0.641375 | 3.501034 | 1.415964 | 3.730556 | 2.535238 | 2.025695 | 4.19888 | 0.49465 | 4.36431 | 1.41979 | 1.233172 | low |
| TCGA-AA-3930 | 0.166666667 | 1 | 2.352983 | 0.345802 | 4.090906 | 1.331624 | 4.62507 | 4.238043 | 3.118681 | 3.977667 | 2.189602 | 3.925009 | 2.436565 | 1.597977 | high |
| TCGA-AA-3982 | 2.25 | 0 | 2.783108 | 0.954932 | 3.195924 | 1.890378 | 3.602498 | 2.839825 | 1.910898 | 3.63851 | 1.885532 | 3.589029 | 0.179898 | 1.085069 | low |
| TCGA-AA-3510 | 5.3275 | 0 | 3.404802 | 0.353709 | 4.330398 | 1.11619 | 3.417576 | 1.131148 | 3.19258 | 2.674121 | 2.906685 | 3.683992 | 1.429694 | 1.0308 | low |
| TCGA-G4-6310 | 5.2975 | 0 | 0.278867 | 0.677528 | 3.52953 | 6.882178 | 3.102943 | 1.323764 | 3.918455 | 3.425363 | 1.398233 | 4.38535 | 3.529778 | 1.749725 | high |
| TCGA-AA-A00K | 1.503333333 | 0 | 0.981078 | 0.282173 | 4.874696 | 2.713019 | 3.707943 | 0.945231 | 2.728209 | 3.186546 | 2.407129 | 3.671811 | 2.178422 | 1.497765 | high |
| TCGA-G4-6588 | 2.179166667 | 0 | 2.796258 | 0.382485 | 4.757161 | 0.38838 | 3.571098 | 3.445462 | 3.35473 | 5.838436 | 2.689963 | 5.649647 | 1.17756 | 1.477659 | high |
| TCGA-CK-5912 | 4.0875 | 1 | 1.270881 | 0.017808 | 3.673716 | 3.05265 | 3.5742 | 0.564526 | 1.423927 | 3.605684 | 3.003652 | 2.416251 | 2.18652 | 1.262594 | low |
| TCGA-CM-5344 | 1.834166667 | 0 | 5.721353 | 0.372582 | 5.203032 | 1.606022 | 3.773384 | 4.707835 | 2.858646 | 4.042333 | 2.287814 | 4.599308 | 1.636111 | 0.968573 | low |
| TCGA-AA-3831 | 1.4975 | 0 | 2.394606 | 0.412586 | 4.126556 | 0.982453 | 3.894417 | 0.255095 | 3.037891 | 2.733615 | 1.583167 | 3.252717 | 0.433282 | 1.13178 | low |
| TCGA-D5-6541 | 1.2975 | 0 | 3.073089 | 0.736554 | 4.36248 | 0.952975 | 3.487552 | 1.054186 | 2.571159 | 4.25904 | 1.155001 | 3.949944 | 0.78159 | 1.109337 | low |
| TCGA-A6-4107 | 2.701666667 | 0 | 1.399337 | 1.260845 | 3.953941 | 1.602485 | 3.072638 | 1.077323 | 2.619511 | 3.129687 | 1.565325 | 3.97623 | 0.820435 | 1.251476 | low |
| TCGA-AA-3875 | 1.503333333 | 0 | 2.939661 | 0.580308 | 4.248272 | 1.678447 | 3.472713 | 1.442187 | 3.16979 | 3.578556 | 2.787066 | 3.670417 | 0.47722 | 1.097215 | low |
| TCGA-G4-6295 | 0.695 | 0 | 1.048253 | 0.711705 | 5.740745 | 5.010075 | 3.800116 | 0.895409 | 1.419873 | 4.618469 | 4.444606 | 3.583715 | 2.242132 | 1.590098 | high |
| TCGA-DM-A28F | 2.995 | 1 | 0.799776 | 0.117229 | 5.022911 | 0.280779 | 4.412776 | 2.171748 | 3.428246 | 3.021033 | 1.402555 | 3.44696 | 2.183384 | 1.631041 | high |
| TCGA-CM-4743 | 1.919166667 | 0 | 1.729463 | 0.244439 | 4.445339 | 0.137202 | 4.058312 | 0.480783 | 3.164775 | 3.696233 | 2.745832 | 5.712369 | 1.522379 | 1.616134 | high |
| TCGA-AA-A00U | 1.418333333 | 0 | 1.867886 | 0.236006 | 5.033867 | 0.949563 | 4.499542 | 0.117166 | 2.650843 | 4.581352 | 1.800299 | 3.786772 | 1.805468 | 1.53936 | high |
| TCGA-CM-5861 | 1.250833333 | 0 | 0.38459 | 4.841564 | 4.579054 | 0.935918 | 3.314681 | 3.903403 | 3.798788 | 5.451377 | 2.509922 | 4.382125 | 3.795422 | 2.035646 | high |
| TCGA-CK-4948 | 12.325 | 0 | 1.430782 | 0.434575 | 4.104012 | 0.977948 | 5.016087 | 2.486647 | 2.753243 | 4.683798 | 3.278303 | 4.792523 | 3.892133 | 2.002207 | high |
| TCGA-AA-3968 | 1.831666667 | 0 | 4.863025 | 0.850196 | 3.451552 | 3.09257 | 3.671833 | 0.576574 | 2.099442 | 3.636528 | 1.321471 | 4.106925 | 0.398126 | 0.863414 | low |
| TCGA-D5-6530 | 1.7 | 0 | 2.359934 | 0.422297 | 4.35415 | 0.90488 | 2.541885 | 0.855496 | 3.043063 | 4.408116 | 0.817817 | 3.136802 | 0.784491 | 0.941455 | low |
| TCGA-G4-6315 | 5.155 | 0 | 4.068499 | 0.325578 | 2.966241 | 0.813532 | 3.400699 | 0.714495 | 3.112821 | 3.875445 | 0.675669 | 2.922025 | 0.819956 | 0.823757 | low |
| TCGA-AA-3562 | 1.664166667 | 0 | 5.680452 | 0.522217 | 3.567612 | 1.520231 | 4.737103 | 0.923665 | 1.72281 | 3.879241 | 1.491201 | 4.208408 | 0.550049 | 0.954535 | low |
| TCGA-AA-A010 | 2.9125 | 0 | 3.032146 | 2.269903 | 3.713923 | 2.980856 | 2.467905 | 0.924047 | 2.726884 | 3.301658 | 2.131871 | 4.757913 | 3.966103 | 1.270538 | low |
| TCGA-AA-3952 | 0.166666667 | 1 | 1.217856 | 0.241007 | 4.971496 | 1.06855 | 3.740273 | 1.612887 | 2.537874 | 3.933816 | 0.704153 | 5.561537 | 0.995163 | 1.558321 | high |
| TCGA-AA-3532 | 2.415 | 0 | 5.457202 | 1.633378 | 4.792217 | 1.350037 | 3.456584 | 1.60115 | 2.464185 | 3.952629 | 1.968673 | 4.454695 | 0.696172 | 0.857096 | low |
| TCGA-AA-3989 | 0.6625 | 1 | 0.144215 | 1.006511 | 2.842996 | 1.627132 | 3.071683 | 1.256722 | 2.243808 | 3.192535 | 0.680559 | 4.580798 | 2.218817 | 1.542839 | high |
| TCGA-AA-3530 | 1.5875 | 0 | 0.303699 | 0.6436 | 3.958591 | 2.534527 | 3.140817 | 2.064501 | 3.028351 | 2.61763 | 3.39182 | 3.061598 | 1.303925 | 1.375606 | high |
| TCGA-NH-A5IV | 1.61 | 0 | 2.282604 | 0.740646 | 5.203621 | 0.813981 | 3.337609 | 1.407497 | 3.905092 | 4.824814 | 0.389092 | 4.487096 | 0.475546 | 1.297002 | low |
| TCGA-CK-4947 | 1.461666667 | 0 | 2.314473 | 0.552181 | 4.227158 | 1.460324 | 2.866117 | 1.466079 | 4.209156 | 3.310831 | 1.492646 | 3.72055 | 0.957953 | 1.109796 | low |
| TCGA-AA-3492 | 0.251666667 | 1 | 0.801021 | 0.302477 | 4.78235 | 1.222427 | 3.772254 | 1.397951 | 3.110593 | 3.654763 | 1.874298 | 4.678195 | 2.636514 | 1.679522 | high |
| TCGA-AZ-4682 | 1.861666667 | 1 | 5.069765 | 0.403984 | 4.426696 | 1.706602 | 3.600108 | 1.488428 | 1.060872 | 3.172475 | 1.293106 | 3.060708 | 0.529943 | 0.64184 | low |
| TCGA-G4-6293 | 11.09 | 0 | 0.255687 | 0.382784 | 3.093548 | 0.886717 | 2.453887 | 0.406477 | 3.483108 | 3.419309 | 1.708107 | 3.671676 | 2.011266 | 1.326166 | low |
| TCGA-AA-3552 | 1.084166667 | 1 | 5.489052 | 0.735987 | 3.890303 | 1.547545 | 4.051904 | 0.811269 | 2.916769 | 4.013101 | 0.686747 | 4.13169 | 0.257351 | 0.868751 | low |
| TCGA-CM-6166 | 1.831666667 | 0 | 1.302241 | 0.191248 | 4.033808 | 3.494153 | 4.658651 | 0.471403 | 3.525195 | 3.856063 | 2.820651 | 4.28051 | 4.221524 | 1.912228 | high |
| TCGA-AA-3496 | 0.085 | 0 | 1.598966 | 0.554787 | 4.351035 | 2.044523 | 4.831055 | 0.487298 | 3.199909 | 3.730371 | 0.871408 | 4.111424 | 0.628366 | 1.598174 | high |
| TCGA-AY-A8YK | 1.568333333 | 0 | 5.96958 | 0.400694 | 3.112238 | 1.963049 | 3.398887 | 0.493401 | 0.982445 | 3.456792 | 0.49489 | 2.984244 | 1.645805 | 0.529197 | low |
| TCGA-CM-6677 | 0.9225 | 0 | 1.99127 | 0.274541 | 5.066709 | 1.404642 | 3.915496 | 3.043335 | 1.526558 | 4.223016 | 1.703407 | 4.205485 | 1.393104 | 1.387422 | high |
| TCGA-A6-2677 | 2.025833333 | 1 | 0.378523 | 1.575158 | 3.732162 | 3.134238 | 1.94001 | 1.249048 | 3.742167 | 2.375977 | 2.994475 | 2.708228 | 1.344234 | 1.141066 | low |
| TCGA-AA-3544 | 1.165833333 | 0 | 4.330745 | 0.407876 | 4.218661 | 1.324662 | 3.642461 | 0.608732 | 2.143184 | 4.455565 | 2.111481 | 3.836624 | 0.561061 | 0.925611 | low |
| TCGA-A6-A567 | 5.149166667 | 1 | 0.534342 | 0.40767 | 3.018363 | 2.883379 | 3.422946 | 2.666531 | 3.26756 | 3.099295 | 1.634561 | 4.326881 | 1.015795 | 1.507022 | high |
| TCGA-AA-3867 | 2.000833333 | 0 | 4.15176 | 0.836865 | 3.62575 | 2.81697 | 4.905257 | 4.867686 | 2.28733 | 4.668158 | 1.725088 | 5.966311 | 1.274538 | 1.554092 | high |
| TCGA-NH-A50V | 1.61 | 0 | 0.757651 | 0.272713 | 5.695979 | 0.374152 | 3.186485 | 0.822516 | 2.985002 | 3.924374 | 1.252287 | 4.801075 | 1.179251 | 1.461655 | high |
| TCGA-AA-A01C | 1.250833333 | 0 | 0.919588 | 0.506127 | 3.962114 | 2.01445 | 3.512875 | 1.919293 | 1.970347 | 4.441698 | 1.320949 | 5.055691 | 1.771372 | 1.586009 | high |
| TCGA-AA-A00O | 2.25 | 0 | 0.545376 | 0.253957 | 3.745646 | 3.745462 | 3.762281 | 1.137117 | 3.383637 | 3.245548 | 0.646297 | 4.789864 | 0.661631 | 1.588134 | high |
| TCGA-AA-A00D | 1.5825 | 0 | 0.767266 | 0.465749 | 4.458116 | 1.698744 | 3.83571 | 0.426178 | 2.784786 | 4.460786 | 2.114757 | 4.708063 | 1.793903 | 1.66799 | high |
| TCGA-AA-3522 | 3.085 | 0 | 3.083418 | 0.306336 | 4.107519 | 1.339202 | 2.29989 | 1.515035 | 3.54892 | 2.716469 | 2.016911 | 2.526799 | 1.435343 | 0.744097 | low |
| TCGA-AA-3979 | 1.998333333 | 0 | 2.750577 | 0.470392 | 4.666236 | 0.239216 | 4.144171 | 1.96709 | 2.389727 | 4.638053 | 1.899761 | 4.354214 | 0.6317 | 1.3257 | low |
| TCGA-A6-5667 | 2.428333333 | 0 | 1.961161 | 1.533818 | 4.232917 | 3.757544 | 4.390622 | 1.423878 | 3.552969 | 4.374196 | 2.678963 | 4.745223 | 1.953856 | 1.748805 | high |
| TCGA-AA-3842 | 3.0825 | 0 | 0.251339 | 0.50999 | 4.236215 | 1.34385 | 3.443703 | 3.589738 | 1.621361 | 5.142604 | 2.30764 | 4.815248 | 0.814996 | 1.613496 | high |
| TCGA-A6-A5ZU | 0.8025 | 0 | 0.600148 | 0.258802 | 3.712453 | 0.974694 | 3.069931 | 1.980476 | 2.657061 | 2.872729 | 3.020905 | 4.097744 | 0.781348 | 1.333331 | low |
| TCGA-AA-3511 | 0.58 | 0 | 5.326882 | 0.305527 | 4.288595 | 1.124177 | 3.370303 | 1.565493 | 2.234319 | 3.331989 | 1.409916 | 4.434302 | 1.082112 | 0.772741 | low |
| TCGA-NH-A8F8 | 1.399166667 | 1 | 0.75206 | 0.197343 | 5.352798 | 3.194003 | 3.814267 | 3.492793 | 1.935973 | 3.722362 | 2.031635 | 5.340918 | 1.682489 | 1.694518 | high |
| TCGA-AA-3973 | 1.086666667 | 0 | 1.222334 | 0.391334 | 3.482755 | 2.022015 | 3.715453 | 2.581095 | 2.054933 | 2.503886 | 1.613911 | 3.959787 | 1.483427 | 1.381975 | high |
| TCGA-AA-3680 | 0.9175 | 1 | 1.90182 | 0.773964 | 4.008304 | 3.193171 | 2.487598 | 2.090474 | 3.937431 | 3.815519 | 2.154228 | 2.869487 | 2.76935 | 1.190171 | low |
| TCGA-QG-A5YW | 2.4525 | 0 | 9.278107 | 0.429709 | 3.938938 | 0.755293 | 2.543972 | 1.130381 | 2.850739 | 5.233744 | 1.793627 | 3.901461 | 2.059253 | 0.171817 | low |
| TCGA-AY-A69D | 1.486666667 | 0 | 1.501675 | 0.53557 | 3.81381 | 1.52544 | 2.56301 | 0.915701 | 2.189884 | 2.662086 | 1.99555 | 3.673557 | 2.111648 | 1.123222 | low |
| TCGA-AA-3673 | 4.166666667 | 0 | 1.583246 | 0.284218 | 3.891424 | 3.095751 | 4.12847 | 1.180187 | 3.115553 | 3.638887 | 2.856705 | 3.951703 | 2.817936 | 1.613821 | high |
| TCGA-AA-3855 | 2.669166667 | 0 | 4.052425 | 0.815062 | 2.863491 | 1.568341 | 2.598261 | 0.907577 | 2.97575 | 2.942844 | 3.451367 | 6.29685 | 1.64927 | 1.061217 | low |
| TCGA-CM-6172 | 0.9175 | 0 | 4.247422 | 0.662179 | 2.728629 | 1.850355 | 2.562215 | 0.776507 | 3.388106 | 3.339295 | 1.349222 | 3.936089 | 2.040185 | 0.831599 | low |
| TCGA-AA-3971 | 1.338333333 | 0 | 7.103003 | 0.85272 | 2.750976 | 2.772358 | 2.649322 | 1.178341 | 2.088704 | 2.658332 | 1.445304 | 3.176392 | 0.835425 | 0.235872 | low |
| TCGA-AA-3666 | 0.166666667 | 1 | 1.916717 | 0.422025 | 4.022786 | 0.638241 | 3.237374 | 0 | 4.146453 | 4.485951 | 1.894403 | 3.181808 | 0.29396 | 1.164816 | low |
| TCGA-D5-6537 | 0.4 | 1 | 2.198253 | 0.14609 | 3.788089 | 2.535354 | 3.494566 | 1.294349 | 2.939224 | 3.402574 | 4.507587 | 3.460928 | 2.131822 | 1.29091 | low |
| TCGA-G4-6321 | 1.84 | 0 | 5.255264 | 0.998596 | 1.770631 | 1.02052 | 2.253543 | 3.10225 | 4.512859 | 2.794047 | 0.570794 | 1.750208 | 1.006265 | 0.381082 | low |
| TCGA-T9-A92H | 0.990833333 | 0 | 3.119325 | 0.035003 | 4.193813 | 0.375836 | 2.930656 | 0.934984 | 2.419038 | 2.457612 | 1.956107 | 3.791427 | 3.874402 | 1.070731 | low |
| TCGA-AD-6965 | 2.204166667 | 0 | 1.455693 | 0.217776 | 4.710677 | 1.591883 | 3.781425 | 1.759179 | 3.622001 | 3.702565 | 2.678896 | 4.252433 | 2.147852 | 1.551866 | high |
| TCGA-A6-6140 | 2.009166667 | 0 | 4.318308 | 2.127166 | 3.75322 | 1.047525 | 2.96129 | 0.472714 | 1.105897 | 3.147873 | 2.758045 | 2.574133 | 0.553993 | 0.643558 | low |
| TCGA-AA-3553 | 1.998333333 | 0 | 3.300741 | 0.639868 | 4.447435 | 1.020256 | 4.371657 | 4.563368 | 3.14617 | 4.340245 | 3.747914 | 4.772391 | 0.857657 | 1.434267 | high |
| TCGA-A6-A56B | 4.684166667 | 1 | 0.721075 | 0.859958 | 6.727629 | 1.681693 | 3.39391 | 2.200809 | 3.290194 | 4.096143 | 2.007094 | 5.365682 | 2.904582 | 1.776499 | high |
| TCGA-AA-A00E | 2.499166667 | 0 | 2.126006 | 0.934079 | 5.565517 | 0.562854 | 3.973837 | 1.028852 | 3.609877 | 4.602883 | 1.459847 | 5.035856 | 0.68318 | 1.511881 | high |
| TCGA-AA-3956 | 2.833333333 | 0 | 2.090373 | 0.581544 | 3.965848 | 3.004272 | 2.977221 | 1.315464 | 3.015938 | 3.43746 | 2.725109 | 3.502315 | 0.114428 | 1.074078 | low |
| TCGA-AA-3713 | 1.585 | 0 | 5.953201 | 1.095178 | 4.538853 | 1.47661 | 3.139936 | 1.739815 | 4.199283 | 4.503645 | 0.543272 | 4.484719 | 0.792743 | 0.787625 | low |
| TCGA-AA-3955 | 1.746666667 | 0 | 1.573616 | 0.26016 | 3.404598 | 0.916517 | 3.237148 | 1.143681 | 1.345124 | 2.130413 | 2.246368 | 2.663322 | 0.357983 | 0.958109 | low |
| TCGA-AZ-6598 | 4.115 | 1 | 0.394742 | 3.122413 | 5.328468 | 3.302267 | 3.578867 | 1.899575 | 2.276303 | 4.608357 | 2.15393 | 3.664835 | 2.518257 | 1.765348 | high |
| TCGA-CM-6163 | 1.169166667 | 0 | 6.822682 | 1.709151 | 3.558956 | 3.6388 | 3.039652 | 1.304665 | 2.178103 | 3.164493 | 0.767031 | 3.889992 | 0.332703 | 0.463325 | low |
| TCGA-AA-3869 | 2.25 | 1 | 0.595353 | 2.047713 | 4.715237 | 1.724833 | 3.356834 | 0.378607 | 1.25435 | 3.422154 | 2.76923 | 4.069716 | 1.832776 | 1.507483 | high |
| TCGA-AA-3538 | 2.165833333 | 0 | 4.916816 | 0.576758 | 4.019078 | 1.45145 | 3.470283 | 2.725763 | 2.829684 | 3.943503 | 3.289419 | 4.842713 | 0.569759 | 0.954385 | low |
| TCGA-AA-3494 | 0.085 | 0 | 3.450444 | 0.437155 | 4.451167 | 4.158411 | 3.039213 | 0.100581 | 2.774038 | 4.211544 | 3.481271 | 3.199169 | 2.455155 | 1.06156 | low |
| TCGA-AA-3851 | 2.754166667 | 0 | 1.835107 | 1.179766 | 4.283092 | 0.912946 | 3.328712 | 1.070362 | 3.085605 | 3.858865 | 1.359203 | 3.45973 | 3.253684 | 1.400292 | high |
| TCGA-A6-5665 | 1.836666667 | 0 | 0.679673 | 1.12236 | 3.696523 | 1.403388 | 2.968331 | 3.067293 | 3.404686 | 3.628416 | 3.019799 | 3.903223 | 0.646094 | 1.392562 | high |
| TCGA-AA-3509 | 5.2425 | 0 | 3.976461 | 1.114181 | 4.14558 | 1.360001 | 3.142694 | 1.756938 | 3.211423 | 2.694202 | 0.696073 | 3.232869 | 0.61945 | 0.813 | low |
| TCGA-AA-3970 | 3.000833333 | 0 | 4.983567 | 0.620521 | 5.394495 | 3.216352 | 3.378457 | 1.764854 | 2.783639 | 3.336606 | 2.618308 | 3.290565 | 0.532862 | 0.762668 | low |
| TCGA-CK-5914 | 1.831666667 | 0 | 0.242246 | 0.533729 | 4.87753 | 0.819916 | 3.286332 | 0.343129 | 1.086606 | 3.233518 | 0.810591 | 3.983762 | 1.679537 | 1.405353 | high |
| TCGA-A6-5666 | 2.724166667 | 0 | 0.688508 | 0.062313 | 5.16578 | 0.998127 | 3.666638 | 0.850928 | 0.586688 | 2.891535 | 2.532548 | 2.510581 | 0.632658 | 1.180217 | low |
| TCGA-CM-6171 | 1.169166667 | 0 | 1.404533 | 0.807082 | 4.165608 | 3.113104 | 2.428054 | 0 | 4.478135 | 5.191282 | 2.827458 | 2.735392 | 2.425137 | 1.281071 | low |
| TCGA-G4-6309 | 7.1175 | 0 | 3.479374 | 0.190421 | 4.489001 | 0.326709 | 2.818635 | 3.527091 | 2.771506 | 4.998062 | 2.435101 | 4.449033 | 0.98369 | 1.024269 | low |
| TCGA-AA-3697 | 7.0825 | 0 | 5.154657 | 1.107577 | 4.375651 | 1.233817 | 3.737047 | 1.682845 | 3.013087 | 3.100879 | 2.950742 | 4.166485 | 1.044196 | 0.924132 | low |
| TCGA-G4-6586 | 2.981666667 | 0 | 4.532326 | 0.291578 | 4.722734 | 0.355359 | 3.172315 | 0.600928 | 3.361286 | 4.86481 | 2.663618 | 2.974931 | 1.58751 | 0.848484 | low |
| TCGA-AZ-4616 | 0.426666667 | 1 | 1.468177 | 0.958892 | 5.130261 | 3.287313 | 4.751417 | 4.267771 | 2.129735 | 5.116464 | 4.302011 | 4.702769 | 4.118776 | 2.033107 | high |
| TCGA-AA-A02J | 0.419166667 | 1 | 4.077757 | 0.211123 | 4.256062 | 1.947014 | 3.714089 | 0.926145 | 2.502354 | 4.301209 | 1.732385 | 2.983893 | 0.949832 | 0.927577 | low |
| TCGA-NH-A6GB | 1.303333333 | 0 | 1.735861 | 0.999071 | 3.565801 | 0.245305 | 3.360073 | 0.067007 | 2.753316 | 5.337279 | 4.012821 | 3.799856 | 0.970444 | 1.344943 | low |
| TCGA-CK-6747 | 6.906666667 | 0 | 1.529675 | 0.074034 | 5.066865 | 1.300616 | 3.144866 | 0.215536 | 3.717799 | 3.642195 | 1.376616 | 2.816822 | 1.202119 | 1.169142 | low |
| TCGA-AD-A5EK | 1.369166667 | 0 | 0.547988 | 0.182218 | 4.635423 | 3.240275 | 3.303907 | 0.457546 | 3.326536 | 3.821454 | 1.545152 | 3.318952 | 1.718586 | 1.447497 | high |
| TCGA-AA-3506 | 4.831666667 | 0 | 3.351735 | 0.313619 | 3.56411 | 4.579105 | 3.567802 | 2.923822 | 3.629187 | 4.377851 | 3.524025 | 5.254558 | 2.846137 | 1.4746 | high |
| TCGA-G4-6298 | 1.9575 | 1 | 0.085669 | 0.462047 | 5.38292 | 0.842604 | 4.355208 | 4.643245 | 2.738659 | 3.637558 | 3.020111 | 5.493364 | 2.036842 | 1.97265 | high |
| TCGA-AA-3678 | 3.915 | 0 | 4.985846 | 0.940484 | 3.988106 | 0.827063 | 4.095918 | 0.247952 | 2.967277 | 3.034733 | 2.092544 | 3.681622 | 0.661949 | 0.904219 | low |
| TCGA-F4-6806 | 3.449166667 | 0 | 2.068311 | 1.187346 | 4.495083 | 1.842689 | 2.947107 | 0.78737 | 2.350959 | 3.243596 | 2.222969 | 3.444106 | 1.996649 | 1.164284 | low |
| TCGA-AA-3812 | 2.918333333 | 0 | 8.13567 | 1.475752 | 4.437488 | 2.90865 | 4.474549 | 2.866424 | 2.403754 | 3.796617 | 2.157245 | 6.437459 | 2.875629 | 1.049959 | low |
| TCGA-AA-3815 | 2.751666667 | 0 | 2.566515 | 1.173157 | 4.84681 | 0.81996 | 2.844025 | 1.953767 | 4.05197 | 4.093675 | 3.739365 | 3.958407 | 1.328331 | 1.200761 | low |
| TCGA-CA-6715 | 1.048333333 | 0 | 0.205262 | 2.661858 | 5.022879 | 3.819905 | 3.727642 | 2.089443 | 2.570846 | 3.639593 | 0.539461 | 3.08418 | 2.791993 | 1.723115 | high |
| TCGA-NH-A6GA | 0.826666667 | 1 | 0.306438 | 0.155193 | 4.465613 | 2.117528 | 2.885388 | 4.250767 | 5.026148 | 4.499897 | 4.300542 | 5.469652 | 1.75443 | 1.756728 | high |
| TCGA-AD-6889 | 6.931666667 | 1 | 0.721208 | 0.164045 | 4.812267 | 2.477368 | 3.113782 | 0.600194 | 3.327695 | 4.019203 | 2.122751 | 2.976245 | 2.384118 | 1.4044 | high |
| TCGA-AA-A029 | 4.328333333 | 0 | 0.903163 | 0.136753 | 4.414478 | 0.257463 | 4.220394 | 0.19155 | 3.288603 | 3.960321 | 1.752875 | 5.931117 | 1.071855 | 1.753556 | high |
| TCGA-AA-3655 | 5.080833333 | 0 | 2.662845 | 0.171298 | 4.906554 | 4.638412 | 3.568735 | 1.618448 | 3.24419 | 4.050015 | 3.272762 | 3.269823 | 0.932332 | 1.204649 | low |
| TCGA-CM-6678 | 0.9175 | 0 | 1.476446 | 0.061635 | 3.863803 | 3.05885 | 2.709402 | 1.456958 | 3.416781 | 3.231219 | 2.474182 | 3.733688 | 2.598356 | 1.282026 | low |
| TCGA-AA-3660 | 6.501666667 | 0 | 6.663669 | 0.698965 | 5.11688 | 2.082151 | 3.171211 | 1.223581 | 1.8568 | 4.617823 | 2.676436 | 3.792941 | 2.169275 | 0.645525 | low |
| TCGA-DM-A0XF | 3.180833333 | 1 | 0.556418 | 0.263081 | 5.013006 | 0.43833 | 3.856724 | 5.121445 | 2.379099 | 3.602202 | 2.693983 | 4.142052 | 0.515179 | 1.536726 | high |
| TCGA-QG-A5Z1 | 0.700833333 | 1 | 1.315843 | 0.156019 | 4.831193 | 3.295476 | 3.042462 | 0.396818 | 2.683878 | 3.78312 | 2.067912 | 4.313467 | 1.501791 | 1.344168 | low |
| TCGA-AA-3526 | 1.5875 | 0 | 1.835051 | 3.024967 | 4.991776 | 1.610161 | 4.064938 | 1.099466 | 2.787262 | 3.513535 | 3.510933 | 3.732645 | 1.409125 | 1.545943 | high |
| TCGA-CA-5255 | 1.029166667 | 0 | 1.97655 | 0.086496 | 4.907033 | 3.289307 | 2.357643 | 4.230633 | 4.247231 | 4.276262 | 3.997065 | 3.602736 | 0.555474 | 1.121072 | low |
| TCGA-5M-AAT4 | 0.134166667 | 1 | 0.806753 | 0.163318 | 4.684128 | 0.637065 | 4.412964 | 4.691065 | 3.060526 | 4.868455 | 2.832478 | 5.149275 | 0.676158 | 1.800538 | high |
| TCGA-AA-3864 | 4.413333333 | 0 | 2.521305 | 0.245065 | 4.988038 | 2.583107 | 3.741374 | 1.101476 | 1.253713 | 4.3918 | 2.158523 | 4.128089 | 0.269309 | 1.187303 | low |
| TCGA-CM-6161 | 1.250833333 | 0 | 1.064924 | 0.716429 | 3.80877 | 1.810873 | 3.393969 | 0.318156 | 4.269727 | 3.354636 | 0.583278 | 4.223435 | 2.373364 | 1.531955 | high |
| TCGA-AA-3819 | 2.083333333 | 0 | 6.077921 | 0.493527 | 3.134193 | 1.147988 | 3.399955 | 1.290523 | 2.913938 | 3.01972 | 2.166134 | 3.46287 | 0.408607 | 0.556263 | low |
| TCGA-AA-3685 | 3.085 | 0 | 4.266273 | 0.706284 | 4.391964 | 0.969102 | 3.318402 | 1.048928 | 3.180048 | 2.708096 | 1.785137 | 4.794302 | 0.674813 | 0.944827 | low |
| TCGA-AA-3664 | 4.498333333 | 0 | 2.613721 | 1.216159 | 6.226454 | 1.150891 | 3.270221 | 1.109009 | 4.189209 | 4.47444 | 3.362207 | 3.605964 | 1.054962 | 1.253296 | low |
| TCGA-A6-6648 | 2.096666667 | 0 | 6.485887 | 0.748839 | 4.424465 | 2.229167 | 2.292846 | 0.205929 | 3.962739 | 3.538053 | 2.718921 | 2.664483 | 0.637624 | 0.305019 | low |
| TCGA-AZ-4684 | 5.4125 | 0 | 4.65692 | 0.64768 | 5.632309 | 1.879412 | 4.49884 | 1.242172 | 1.462294 | 5.152354 | 2.615174 | 4.572238 | 1.557402 | 1.237539 | low |
| TCGA-AA-A00A | 3.1675 | 0 | 0.757266 | 0.541063 | 5.18813 | 0.441125 | 3.52054 | 1.265222 | 0.958736 | 3.234606 | 2.920631 | 4.403234 | 0.16486 | 1.342697 | low |
| TCGA-CM-5349 | 2.505 | 0 | 0.16176 | 2.906392 | 4.193849 | 3.81936 | 3.818893 | 2.033128 | 3.135322 | 4.037428 | 2.145641 | 4.61163 | 2.196673 | 1.917969 | high |
| TCGA-AA-3534 | 2.415 | 0 | 3.250482 | 0.8434 | 2.753488 | 4.585583 | 2.907409 | 1.910368 | 3.456385 | 2.793966 | 2.774823 | 4.153152 | 2.683781 | 1.159992 | low |
| TCGA-AA-3663 | 0.58 | 0 | 0.434061 | 0.177942 | 5.725884 | 0.391416 | 3.605463 | 0.45935 | 3.206003 | 4.583606 | 2.196493 | 3.675307 | 1.70283 | 1.563334 | high |
| TCGA-AA-3549 | 1.749166667 | 0 | 3.729006 | 0.855605 | 4.618174 | 0.816806 | 4.005033 | 0.528813 | 0.782904 | 3.494502 | 0.028382 | 4.435761 | 0.653304 | 1.041412 | low |
| TCGA-G4-6626 | 3.8925 | 1 | 1.146681 | 0.648459 | 4.586813 | 0.176446 | 3.964567 | 0.856323 | 3.158864 | 4.337915 | 2.73131 | 3.30292 | 0.91125 | 1.456251 | high |
| TCGA-AY-6386 | 1.484166667 | 0 | 2.386396 | 0.231357 | 4.502771 | 0.898373 | 2.870727 | 0.659551 | 3.542497 | 3.707402 | 2.769475 | 3.307366 | 1.887252 | 1.101891 | low |
| TCGA-AU-3779 | 1.2075 | 0 | 1.29038 | 3.619145 | 4.07278 | 2.411002 | 3.210349 | 0.566361 | 2.558433 | 3.26395 | 1.952144 | 4.301914 | 1.45499 | 1.491512 | high |
| TCGA-AA-3517 | 3.246666667 | 0 | 2.8889 | 0.818125 | 4.161701 | 2.825677 | 2.942072 | 0.3994 | 2.623647 | 3.204529 | 1.829815 | 4.526017 | 0.695056 | 1.056679 | low |
| TCGA-AA-3518 | 0.085 | 0 | 1.431436 | 1.405088 | 5.034375 | 2.195951 | 3.412859 | 0.66135 | 3.482419 | 4.830299 | 1.907438 | 4.164456 | 1.521121 | 1.514055 | high |
| TCGA-CM-4752 | 1.084166667 | 0 | 4.663846 | 0.153854 | 4.24692 | 1.28533 | 3.334938 | 1.337288 | 1.845164 | 3.933626 | 0.7026 | 4.17748 | 0.642798 | 0.797693 | low |
| TCGA-A6-5660 | 2.430833333 | 0 | 1.1286 | 0.105231 | 4.439595 | 1.64954 | 3.744355 | 1.273964 | 3.682218 | 3.829061 | 2.323112 | 4.426183 | 0.461986 | 1.482559 | high |
| TCGA-CM-5868 | 1.418333333 | 0 | 0.843047 | 0.255014 | 4.250955 | 1.211429 | 3.766847 | 3.233601 | 3.287831 | 3.937123 | 1.88797 | 3.93257 | 1.322073 | 1.542623 | high |
| TCGA-CM-6164 | 2.4175 | 0 | 1.01903 | 0.142699 | 4.716572 | 4.080611 | 4.255529 | 2.429134 | 3.217137 | 3.615208 | 2.481557 | 3.839492 | 1.448215 | 1.636281 | high |
| TCGA-AZ-6606 | 0.9775 | 1 | 3.127433 | 0.108048 | 6.17447 | 1.541111 | 3.071045 | 0.103047 | 3.877566 | 4.225318 | 2.703111 | 4.888912 | 1.974886 | 1.239845 | low |
| TCGA-QG-A5YV | 3.561666667 | 0 | 0.584078 | 0.589463 | 4.579662 | 1.386049 | 2.695652 | 0.051925 | 2.622474 | 3.314479 | 2.606792 | 3.779004 | 1.309864 | 1.283561 | low |
| TCGA-AA-3688 | 1.5825 | 0 | 2.719246 | 0.314266 | 2.968765 | 3.334354 | 3.553684 | 0.980518 | 2.574903 | 3.092992 | 0.398541 | 3.414736 | 2.689201 | 1.197492 | low |
| TCGA-AA-3980 | 2.25 | 0 | 3.489268 | 3.164339 | 4.159212 | 0.851124 | 4.191443 | 0.280505 | 3.839888 | 4.115338 | 1.752423 | 3.142292 | 0.687804 | 1.265356 | low |
| TCGA-AA-3525 | 0.670833333 | 0 | 3.970988 | 0.538414 | 4.547327 | 0.73698 | 3.546619 | 2.465231 | 3.624286 | 4.722271 | 1.218036 | 5.490631 | 1.757823 | 1.278826 | low |
| TCGA-AA-3495 | 3.085 | 0 | 2.389496 | 0.853926 | 3.500257 | 1.008222 | 3.147551 | 1.579661 | 2.730329 | 3.559147 | 2.482067 | 3.092987 | 0.488258 | 1.03052 | low |
| TCGA-D5-6531 | 1.478333333 | 0 | 1.994576 | 0.288344 | 4.785318 | 5.509117 | 4.539731 | 0.774732 | 4.332038 | 5.049873 | 3.06871 | 4.50872 | 2.055693 | 1.788814 | high |
| TCGA-CA-5254 | 1.056666667 | 0 | 4.340486 | 2.203077 | 4.51806 | 0.710223 | 3.190276 | 2.5087 | 2.866923 | 5.87252 | 4.20718 | 4.220862 | 0.82438 | 1.096392 | low |
| TCGA-AA-3696 | 0.419166667 | 1 | 2.481117 | 0.164599 | 4.704233 | 2.957565 | 3.380419 | 2.209254 | 4.137298 | 3.520213 | 2.686368 | 4.575246 | 1.931024 | 1.374788 | high |
| TCGA-A6-5662 | 1.965833333 | 0 | 0.369102 | 0.79872 | 3.558968 | 3.136901 | 3.535528 | 1.886523 | 2.700256 | 3.502226 | 2.229294 | 5.356583 | 3.111684 | 1.815096 | high |
| TCGA-AA-3975 | 2.835833333 | 0 | 5.501226 | 0.551635 | 3.520155 | 1.600133 | 3.521107 | 0.309691 | 2.209759 | 3.2472 | 1.181358 | 4.025543 | 0.580742 | 0.698187 | low |
| TCGA-F4-6460 | 2.660833333 | 1 | 1.525224 | 0.717095 | 3.330852 | 4.978104 | 3.487531 | 1.300312 | 2.747175 | 3.780494 | 0.909007 | 4.69489 | 2.607228 | 1.556498 | high |
| TCGA-AA-3858 | 2.586666667 | 0 | 4.38441 | 0.605374 | 4.084375 | 1.457032 | 4.401807 | 1.799855 | 3.137136 | 4.49029 | 0.884741 | 4.710107 | 0.174316 | 1.18164 | low |
| TCGA-AA-3679 | 1.250833333 | 0 | 2.742713 | 0.917881 | 3.443227 | 1.065029 | 4.905487 | 0.145138 | 2.112793 | 3.077585 | 2.067496 | 3.984844 | 0.317828 | 1.35499 | low |
| TCGA-AY-5543 | 2.748333333 | 0 | 1.846631 | 0.506799 | 5.58378 | 0.977766 | 2.037488 | 0.198875 | 2.667429 | 2.894348 | 0.925532 | 2.764138 | 1.086996 | 0.82031 | low |
| TCGA-AA-A00F | 2.833333333 | 0 | 6.802429 | 1.073178 | 2.785365 | 1.147732 | 3.044213 | 1.715219 | 2.878492 | 3.745404 | 1.979058 | 5.097653 | 0.650502 | 0.610906 | low |
| TCGA-AA-A01K | 2.581666667 | 0 | 0.970767 | 1.300061 | 3.673544 | 2.521407 | 3.988381 | 4.175736 | 3.259844 | 5.10825 | 2.537956 | 5.299873 | 2.610286 | 1.918872 | high |
| TCGA-D5-6932 | 0.9475 | 0 | 0.790304 | 0.222429 | 5.266178 | 1.809748 | 3.709603 | 1.169786 | 2.535672 | 4.227339 | 0.830875 | 5.206654 | 3.763781 | 1.790534 | high |
| TCGA-AA-3675 | 3.9175 | 0 | 1.386077 | 0.194466 | 4.867724 | 1.050454 | 2.793319 | 1.767297 | 3.736534 | 3.610574 | 2.915931 | 3.994474 | 2.344492 | 1.342962 | low |
| TCGA-AA-3681 | 0.498333333 | 0 | 3.578253 | 0.700678 | 4.88611 | 1.123996 | 3.025609 | 1.050683 | 3.995026 | 4.87692 | 2.650675 | 4.120446 | 2.390047 | 1.178137 | low |
| TCGA-AA-A00Q | 3.498333333 | 0 | 0.559848 | 0.607561 | 3.067437 | 1.980078 | 2.711104 | 2.681557 | 2.689895 | 2.565183 | 2.431668 | 4.50091 | 0.378792 | 1.288914 | low |
| TCGA-A6-6141 | 0.698333333 | 0 | 1.088387 | 1.995391 | 3.189813 | 1.969005 | 1.791724 | 0.334777 | 3.122193 | 3.841345 | 2.383881 | 3.402782 | 3.545625 | 1.25222 | low |
| TCGA-AA-3939 | 1.081666667 | 0 | 0.655343 | 0.61558 | 5.46774 | 0.843155 | 3.131215 | 2.014327 | 2.660866 | 2.494736 | 2.489391 | 4.125575 | 2.263514 | 1.446744 | high |
| TCGA-CM-6170 | 1.250833333 | 0 | 3.344351 | 0.873144 | 4.100683 | 1.134146 | 3.44713 | 1.318545 | 1.719645 | 2.912996 | 0.330997 | 3.634953 | 0.561499 | 0.923932 | low |
| TCGA-AA-A02W | 3.414166667 | 0 | 1.189428 | 0.396236 | 4.500967 | 2.857032 | 3.868056 | 1.139895 | 1.275814 | 3.946733 | 2.595389 | 3.580527 | 1.549402 | 1.431237 | high |
| TCGA-AA-3524 | 3.000833333 | 0 | 3.984488 | 0.142399 | 4.789929 | 3.210349 | 4.156598 | 1.874455 | 2.80555 | 4.118923 | 2.391794 | 3.967285 | 0.601663 | 1.140772 | low |
| TCGA-AA-3841 | 3.0775 | 0 | 5.879465 | 0.438232 | 4.386655 | 1.628715 | 4.36642 | 2.431082 | 2.572867 | 4.735141 | 1.845218 | 5.18499 | 1.124552 | 1.077755 | low |
| TCGA-F4-6703 | 3.985833333 | 0 | 7.700741 | 3.811352 | 6.053897 | 2.512571 | 4.986706 | 5.850999 | 3.388924 | 5.239276 | 2.235271 | 5.080442 | 2.034014 | 1.273175 | low |
| TCGA-F4-6461 | 0.925 | 1 | 2.946347 | 1.202496 | 4.759738 | 2.194155 | 3.596193 | 1.991433 | 2.89244 | 4.955203 | 1.904087 | 4.468325 | 3.347083 | 1.478986 | high |
| TCGA-G4-6303 | 5.483333333 | 1 | 1.250656 | 1.237839 | 4.828994 | 3.26547 | 3.778679 | 1.098874 | 3.645075 | 3.55466 | 3.120374 | 4.742572 | 3.310414 | 1.769852 | high |
| TCGA-CM-6675 | 1.086666667 | 0 | 0.929943 | 0.696518 | 2.978823 | 0.579265 | 3.542202 | 3.804546 | 4.469639 | 7.144216 | 2.942907 | 4.227862 | 1.943554 | 1.765593 | high |
| TCGA-A6-5664 | 1.84 | 0 | 1.431164 | 2.949691 | 4.719808 | 3.828527 | 3.136506 | 2.788195 | 3.670265 | 4.459464 | 3.125306 | 4.472831 | 5.055496 | 1.84918 | high |
| TCGA-A6-4105 | 1.21 | 1 | 2.966117 | 0.875778 | 5.075845 | 4.019112 | 5.317522 | 0.446 | 3.228503 | 3.87948 | 3.036608 | 4.781226 | 2.548556 | 1.782183 | high |
| TCGA-AA-3860 | 2.586666667 | 0 | 3.9397 | 0.851458 | 6.214371 | 0.964867 | 3.852788 | 1.564569 | 1.966984 | 3.387813 | 1.245262 | 5.337817 | 0.448621 | 1.13314 | low |
| TCGA-D5-6920 | 1.0325 | 0 | 7.1661 | 1.213569 | 4.70814 | 1.183972 | 3.164052 | 0.573749 | 2.332311 | 3.565195 | 2.112702 | 3.047082 | 1.858596 | 0.450359 | low |
| TCGA-A6-6138 | 1.875 | 0 | 1.072827 | 4.56422 | 5.316303 | 2.000959 | 4.060111 | 0.468678 | 3.593716 | 3.551163 | 2.12584 | 4.00237 | 1.153717 | 1.749083 | high |
| TCGA-A6-2685 | 3.101666667 | 0 | 4.903386 | 1.308734 | 5.432562 | 4.850417 | 4.379698 | 4.614455 | 2.929219 | 4.223327 | 2.7777 | 6.164288 | 2.848395 | 1.544737 | high |
| TCGA-CK-5916 | 1.76 | 1 | 0.435066 | 1.291506 | 4.343115 | 2.435553 | 3.561663 | 4.24703 | 2.056698 | 4.536442 | 3.340548 | 4.296068 | 2.205083 | 1.71988 | high |
| TCGA-AA-3833 | 1.3275 | 0 | 5.154788 | 0.799573 | 4.834724 | 2.805974 | 4.492237 | 3.185715 | 4.181133 | 5.000184 | 1.92984 | 6.835939 | 1.389701 | 1.502991 | high |
| TCGA-G4-6314 | 2.9925 | 0 | 0.452645 | 1.778003 | 5.644474 | 5.091003 | 3.911307 | 4.382629 | 3.277558 | 4.77653 | 2.616863 | 5.291725 | 3.39247 | 2.086069 | high |
| TCGA-CA-6718 | 0.8375 | 1 | 0.189648 | 0.387588 | 5.410056 | 2.890596 | 4.454361 | 5.668049 | 4.131002 | 5.112084 | 4.763624 | 6.474045 | 4.085955 | 2.390901 | high |
| TCGA-CM-6680 | 1.001666667 | 0 | 3.589511 | 1.578067 | 5.320635 | 2.09128 | 3.552185 | 1.923485 | 3.232812 | 3.814154 | 1.291163 | 4.040486 | 4.131315 | 1.368924 | high |
| TCGA-A6-6142 | 2.089166667 | 0 | 0.707099 | 0.100673 | 6.610053 | 2.531378 | 5.785528 | 3.487739 | 1.755886 | 4.521754 | 2.972721 | 5.750771 | 3.927915 | 2.335646 | high |
| TCGA-AD-6895 | 2.089166667 | 0 | 0.092305 | 1.872331 | 5.772205 | 3.570631 | 4.794773 | 2.77018 | 4.312429 | 5.251928 | 3.575383 | 6.496444 | 2.604998 | 2.422958 | high |
| TCGA-F4-6807 | 3.583333333 | 0 | 1.358901 | 1.424598 | 6.312332 | 3.957524 | 4.418292 | 2.620101 | 3.277107 | 4.498611 | 2.050823 | 5.253091 | 6.718644 | 2.227366 | high |
| TCGA-D5-6529 | 1.680833333 | 0 | 0.824541 | 0.373368 | 5.296404 | 2.384939 | 3.899724 | 2.147311 | 3.323896 | 4.15057 | 2.632372 | 5.219492 | 0.499351 | 1.674536 | high |
| TCGA-CM-6679 | 0.8375 | 0 | 2.12466 | 0.269914 | 5.155215 | 2.603503 | 3.328471 | 1.93377 | 2.194612 | 3.874703 | 0.684853 | 5.024871 | 1.673245 | 1.350867 | low |
| TCGA-AZ-6607 | 0.265833333 | 1 | 3.747799 | 4.005462 | 7.203064 | 1.888512 | 5.372401 | 5.404714 | 5.287573 | 5.877642 | 3.942288 | 7.576241 | 2.394138 | 2.300134 | high |
| TCGA-A6-3810 | 3.041666667 | 0 | 1.107603 | 0.914494 | 5.356598 | 2.023552 | 3.964583 | 5.113588 | 2.484386 | 3.909811 | 0.93607 | 4.96827 | 1.591405 | 1.685038 | high |
| TCGA-A6-2675 | 3.616666667 | 0 | 7.426305 | 1.623595 | 5.214771 | 2.26386 | 4.301785 | 3.58752 | 3.60992 | 3.885261 | 2.791075 | 4.639007 | 1.519292 | 0.918653 | low |
| TCGA-AA-3554 | 1.495 | 0 | 3.406958 | 1.534425 | 5.119889 | 2.417217 | 4.842497 | 4.024115 | 3.780205 | 6.768051 | 2.814506 | 5.945544 | 1.556185 | 1.855598 | high |
| TCGA-D5-5538 | 4.5475 | 1 | 1.641412 | 2.770571 | 5.73766 | 2.028338 | 3.670466 | 3.310451 | 3.115028 | 3.564439 | 1.982709 | 4.546131 | 1.288404 | 1.579773 | high |
| TCGA-AA-A01P | 3.17 | 1 | 3.827205 | 5.5 | 4.962007 | 3.308004 | 3.002939 | 3.016588 | 3.063096 | 4.957628 | 3.585576 | 6.186143 | 2.139246 | 1.561952 | high |
| TCGA-AZ-5403 | 5.229166667 | 1 | 5.629175 | 0.267196 | 4.903587 | 2.900512 | 4.323255 | 2.987312 | 3.260995 | 4.245526 | 2.688201 | 4.43542 | 1.56666 | 1.097732 | low |
| TCGA-G4-6299 | 6.209166667 | 0 | 4.603971 | 2.35498 | 4.99907 | 2.349011 | 4.118973 | 3.733711 | 5.29858 | 5.751681 | 3.754586 | 5.421656 | 1.665394 | 1.558162 | high |
| TCGA-CK-5913 | 4.273333333 | 0 | 1.665723 | 0.6528 | 6.222939 | 0.792232 | 4.346108 | 2.748494 | 2.345066 | 4.899291 | 3.362158 | 4.276591 | 1.274769 | 1.616329 | high |
| TCGA-AZ-6605 | 0.435 | 1 | 3.164406 | 0.690564 | 5.426487 | 2.075153 | 4.521488 | 2.816678 | 3.472538 | 4.267794 | 2.519836 | 4.804171 | 1.04717 | 1.500885 | high |
| TCGA-CM-4751 | 2.25 | 0 | 3.388526 | 0.541597 | 5.251823 | 1.716713 | 3.342064 | 2.092494 | 3.869959 | 4.167659 | 2.010394 | 4.743033 | 0.847054 | 1.192443 | low |
| TCGA-G4-6627 | 6.228333333 | 0 | 5.939262 | 1.634735 | 5.899393 | 5.031667 | 4.7996 | 3.781835 | 3.626869 | 3.929558 | 2.379738 | 5.508645 | 2.476269 | 1.420544 | high |
| TCGA-CM-6676 | 0.9225 | 0 | 0.473454 | 0.204355 | 4.858931 | 3.745143 | 4.537583 | 0.651171 | 3.283401 | 4.852278 | 2.360641 | 4.618821 | 1.655651 | 1.896261 | high |
| TCGA-CA-6719 | 1.190833333 | 0 | 3.106731 | 1.025322 | 6.474632 | 3.982186 | 5.288563 | 2.128673 | 3.862635 | 4.420027 | 3.087823 | 6.0627 | 1.991656 | 1.922587 | high |
| TCGA-AZ-4615 | 2.743333333 | 0 | 2.310813 | 0.622866 | 5.095941 | 1.271791 | 2.825594 | 3.955119 | 4.285169 | 5.062173 | 3.494044 | 6.487487 | 1.852655 | 1.5629 | high |
| TCGA-A6-5657 | 2.633333333 | 0 | 0.807312 | 0.880258 | 5.199998 | 4.277761 | 3.953464 | 3.823424 | 3.772268 | 4.937 | 3.667818 | 6.673871 | 3.487025 | 2.163917 | high |
| TCGA-D5-6923 | 1.035 | 0 | 2.672952 | 0.548752 | 5.348676 | 0.991197 | 5.326385 | 4.627921 | 3.193795 | 5.348586 | 2.765309 | 5.75358 | 0.68735 | 1.837236 | high |
| TCGA-D5-6922 | 0.843333333 | 0 | 5.140317 | 0.553075 | 4.582529 | 4.459014 | 3.811325 | 1.30485 | 3.116814 | 4.455027 | 2.790206 | 4.592639 | 1.981419 | 1.123379 | low |
| TCGA-AA-3870 | 2.496666667 | 0 | 1.96891 | 0.841386 | 4.718756 | 2.526086 | 5.192524 | 5.894175 | 2.238968 | 5.737583 | 3.102535 | 7.768486 | 1.494677 | 2.181268 | high |
| TCGA-CM-6167 | 1.248333333 | 0 | 2.558996 | 1.788739 | 4.914414 | 6.704491 | 4.753939 | 3.506152 | 4.637378 | 4.819406 | 1.361901 | 5.634309 | 3.897596 | 2.08431 | high |
| TCGA-CM-5348 | 1.913333333 | 0 | 1.858956 | 2.263879 | 5.123427 | 3.927138 | 3.260397 | 3.879666 | 4.357145 | 5.316768 | 3.620861 | 6.352581 | 3.845196 | 1.965842 | high |
| TCGA-A6-2671 | 3.644166667 | 1 | 0.191934 | 0.525059 | 4.315825 | 2.916379 | 4.598039 | 3.079511 | 2.88811 | 4.652746 | 1.343642 | 5.627098 | 1.950697 | 2.053135 | high |
| TCGA-G4-6628 | 6.635833333 | 0 | 3.046621 | 2.10454 | 5.337487 | 2.257073 | 4.062757 | 1.809366 | 4.173303 | 4.889579 | 2.907863 | 2.514685 | 1.874044 | 1.377166 | high |
| TCGA-AZ-6600 | 1.0075 | 1 | 2.558781 | 0.616535 | 5.623301 | 2.206733 | 4.325819 | 2.97322 | 3.3906 | 4.753702 | 2.355247 | 5.77436 | 2.347533 | 1.745492 | high |
| TCGA-D5-6898 | 0.626666667 | 0 | 1.756657 | 0.831949 | 5.167377 | 4.089617 | 4.688243 | 3.863753 | 3.140862 | 5.107364 | 3.174252 | 4.891949 | 1.833762 | 1.868148 | high |
| TCGA-AA-3489 | 0.585833333 | 1 | 1.502291 | 1.576927 | 5.445524 | 3.980544 | 4.939183 | 4.0865 | 3.700481 | 4.370206 | 1.307852 | 5.375334 | 3.214447 | 2.103155 | high |
| TCGA-D5-6931 | 0.999166667 | 0 | 1.744576 | 1.366871 | 4.826261 | 2.96638 | 2.889333 | 1.495495 | 3.748506 | 4.298153 | 3.548811 | 5.485172 | 1.414623 | 1.504021 | high |
| TCGA-D5-6927 | 0.785833333 | 0 | 1.116586 | 0.733689 | 6.595969 | 1.989673 | 4.41811 | 1.895926 | 2.305658 | 5.338627 | 3.234372 | 5.21237 | 1.382591 | 1.834336 | high |
| TCGA-A6-2676 | 3.5725 | 1 | 0.796295 | 1.293925 | 5.950799 | 1.095644 | 3.598045 | 0.783421 | 3.372758 | 4.787402 | 2.537561 | 6.931445 | 0.76533 | 1.842086 | high |
| TCGA-F4-6570 | 0.515 | 1 | 6.05233 | 3.894467 | 4.663547 | 5.543358 | 4.04348 | 6.089061 | 2.889371 | 6.286499 | 3.106744 | 7.092143 | 2.8995 | 1.635142 | high |
| TCGA-CM-6168 | 1.081666667 | 0 | 4.786764 | 1.864897 | 6.10724 | 3.968968 | 4.802736 | 2.753915 | 4.120874 | 4.689383 | 3.495525 | 5.614759 | 2.908391 | 1.676694 | high |
| TCGA-A6-6654 | 1.9875 | 0 | 3.986999 | 1.793842 | 7.484907 | 4.076689 | 4.980931 | 4.089601 | 3.695595 | 4.722146 | 2.975434 | 6.892792 | 2.387865 | 1.909652 | high |
| TCGA-A6-2682 | 1.160833333 | 1 | 2.312891 | 0.274174 | 6.32695 | 2.15755 | 4.448361 | 3.380625 | 2.246024 | 4.079541 | 2.461201 | 5.33282 | 3.842008 | 1.783682 | high |
| TCGA-A6-6782 | 1.689166667 | 0 | 0.195183 | 0.848611 | 4.874165 | 1.400857 | 3.964685 | 4.570742 | 2.342144 | 3.560388 | 0.418504 | 4.40564 | 1.289824 | 1.691382 | high |
| TCGA-CM-5860 | 2.666666667 | 0 | 2.105595 | 0.565251 | 5.444138 | 1.440856 | 4.273114 | 2.385142 | 3.824963 | 4.761094 | 1.677056 | 5.834457 | 0.735171 | 1.682746 | high |
| TCGA-A6-6653 | 2.031666667 | 0 | 2.321884 | 0.586525 | 4.772906 | 0.468138 | 3.707044 | 0.252208 | 1.231683 | 5.622881 | 2.479654 | 4.233632 | 2.75398 | 1.424721 | high |
| TCGA-A6-3808 | 2.775833333 | 0 | 1.55066 | 0.869297 | 5.896879 | 4.867817 | 4.528571 | 2.083539 | 3.944338 | 4.2431 | 3.176052 | 5.307223 | 3.466609 | 2.011273 | high |
| TCGA-A6-6651 | 1.8125 | 0 | 2.215071 | 1.228025 | 5.156041 | 5.605087 | 4.847854 | 3.277083 | 4.100087 | 4.09358 | 1.221862 | 5.820375 | 3.544425 | 2.049076 | high |
| TCGA-F4-6809 | 1.103333333 | 1 | 2.345481 | 1.4979 | 5.236247 | 3.949926 | 4.553288 | 3.900769 | 3.133613 | 3.591046 | 1.736632 | 5.159335 | 3.754751 | 1.86283 | high |
| TCGA-CM-6169 | 1.084166667 | 0 | 1.888937 | 0.582358 | 5.853206 | 3.490334 | 4.313162 | 0.569692 | 3.42832 | 3.846577 | 2.090619 | 4.75536 | 1.562863 | 1.636305 | high |
| TCGA-F4-6855 | 3.9475 | 0 | 1.043987 | 0.63534 | 6.324442 | 2.817894 | 3.923831 | 5.704655 | 3.72644 | 4.346822 | 2.095995 | 5.139093 | 1.577845 | 1.794538 | high |
| TCGA-D5-6536 | 1.486666667 | 0 | 0.738544 | 2.257318 | 4.420071 | 1.456724 | 4.083112 | 1.547236 | 2.676428 | 3.952349 | 1.419054 | 5.118313 | 4.507773 | 2.005221 | high |
| TCGA-A6-6649 | 2.0125 | 0 | 6.055356 | 1.823622 | 3.624706 | 3.706216 | 2.974056 | 3.933103 | 4.757303 | 5.349144 | 3.255814 | 5.041471 | 2.835941 | 1.105026 | low |
| TCGA-G4-6311 | 3.2825 | 0 | 3.179653 | 1.032911 | 6.14505 | 1.226912 | 4.450489 | 3.730809 | 3.528394 | 4.70626 | 3.116592 | 5.287425 | 2.10092 | 1.649817 | high |
| TCGA-G4-6297 | 6.860833333 | 0 | 4.522011 | 0.984575 | 6.524253 | 1.64546 | 4.901402 | 3.874093 | 3.969245 | 4.460183 | 3.166553 | 5.826365 | 1.625023 | 1.58849 | high |
| TCGA-5M-AAT6 | 0.794166667 | 1 | 1.191851 | 3.064977 | 5.435562 | 1.7458 | 4.556308 | 3.583173 | 3.144724 | 4.928173 | 2.805325 | 5.34745 | 1.421969 | 1.992301 | high |
| TCGA-D5-6926 | 0.7525 | 0 | 1.06681 | 1.013379 | 6.353632 | 2.904851 | 4.241119 | 2.820426 | 3.32771 | 4.964473 | 1.744171 | 5.612155 | 0.77664 | 1.842037 | high |
| TCGA-D5-6929 | 1.116666667 | 0 | 0.048574 | 1.686377 | 5.18556 | 2.068497 | 3.399094 | 2.729495 | 2.534403 | 3.80796 | 2.13702 | 4.601534 | 2.917943 | 1.796005 | high |
| TCGA-F4-6569 | 2.975833333 | 0 | 2.638607 | 0.97784 | 5.27063 | 2.799479 | 4.952692 | 3.171608 | 3.960962 | 5.005134 | 2.554053 | 5.948965 | 3.635536 | 2.030714 | high |
| TCGA-A6-2684 | 3.085 | 0 | 1.976412 | 0.740502 | 4.728015 | 4.098156 | 3.015398 | 3.291311 | 2.264193 | 4.313797 | 2.263148 | 5.611768 | 2.224614 | 1.494251 | high |
| TCGA-A6-2681 | 3.7975 | 0 | 2.158511 | 0.631795 | 5.622851 | 3.709971 | 3.857998 | 2.066769 | 2.607472 | 3.9763 | 1.420419 | 5.9003 | 2.735247 | 1.678321 | high |
| TCGA-D5-5541 | 4.656666667 | 0 | 2.27783 | 1.723818 | 4.959244 | 2.096734 | 4.729918 | 3.596446 | 3.417265 | 4.304121 | 2.760557 | 3.914291 | 0.795681 | 1.619854 | high |
| TCGA-A6-2686 | 3.0825 | 1 | 0.366341 | 1.841849 | 3.6855 | 1.625318 | 4.791497 | 1.883126 | 4.167207 | 4.614497 | 2.263239 | 4.278764 | 1.477651 | 1.995396 | high |
| TCGA-D5-6924 | 1.190833333 | 0 | 1.493452 | 0.416976 | 5.401134 | 3.01279 | 4.233969 | 2.271624 | 2.397024 | 4.295509 | 1.3484 | 4.826353 | 1.592926 | 1.653099 | high |
| TCGA-AY-4070 | 1.3575 | 1 | 1.244408 | 3.909803 | 5.415277 | 1.243024 | 5.825522 | 1.689858 | 3.59679 | 5.500532 | 2.176051 | 5.866431 | 4.803426 | 2.579868 | high |
| TCGA-AA-A02R | 1.834166667 | 1 | 4.873484 | 2.582898 | 5.048509 | 2.219887 | 3.72869 | 3.271356 | 4.250405 | 6.089736 | 2.328045 | 6.406295 | 1.861837 | 1.506607 | high |
| TCGA-F4-6805 | 2.866666667 | 0 | 4.512677 | 1.039728 | 6.656561 | 4.667319 | 4.936129 | 4.44825 | 3.824903 | 5.241096 | 3.921155 | 4.979932 | 2.720671 | 1.677819 | high |
| TCGA-AZ-6601 | 8.3275 | 1 | 1.59372 | 0.954756 | 4.819509 | 3.490894 | 3.952078 | 5.12361 | 4.537913 | 5.308907 | 2.926582 | 6.337123 | 1.531552 | 1.928217 | high |
| TCGA-AU-6004 | 2.255833333 | 0 | 1.069736 | 1.210649 | 4.958886 | 1.741693 | 3.625246 | 1.720512 | 3.037529 | 4.180811 | 2.10528 | 4.544129 | 1.786593 | 1.614599 | high |
| TCGA-DM-A28A | 2.204166667 | 1 | 0.925387 | 1.038412 | 5.013262 | 1.335692 | 4.839141 | 3.386368 | 3.082309 | 4.340885 | 3.097342 | 5.391691 | 1.879026 | 1.997466 | high |
| TCGA-AA-3866 | 1.418333333 | 0 | 1.48288 | 3.042711 | 4.584908 | 2.845318 | 3.708327 | 1.636432 | 2.207586 | 3.806597 | 2.162852 | 4.629208 | 1.32226 | 1.594511 | high |
| TCGA-AZ-4323 | 0.1175 | 1 | 2.875628 | 1.674814 | 4.663826 | 3.981792 | 4.335699 | 4.578539 | 3.425873 | 4.63726 | 2.261266 | 5.401841 | 7.009794 | 2.068251 | high |
| TCGA-AD-6548 | 1.779166667 | 0 | 5.824335 | 1.247803 | 5.953419 | 2.327046 | 4.483621 | 2.89047 | 3.558695 | 3.877309 | 2.479162 | 4.220131 | 2.041175 | 1.151959 | low |
| TCGA-F4-6459 | 0.7175 | 1 | 2.198044 | 2.42357 | 5.048278 | 4.951945 | 4.492345 | 3.846774 | 3.68332 | 4.426945 | 2.777996 | 5.963283 | 2.854999 | 2.010226 | high |

**Table S11 HRI risk scores of 210 patients in the GSE17538 dataset.**

| id | OS time(year) | OS status | CD177 | CP | RGS16 | PGM5 | SNAI1 | CALB2 | OSBPL1A | CDR2L | FRMD5 | FSTL3 | TUBB2B | riskScore | risk |
| --- | --- | --- | --- | --- | --- | --- | --- | --- | --- | --- | --- | --- | --- | --- | --- |
| GSM437093 | 11.87916667 | 0 | 7.430793 | 6.136 | 7.405342 | 6.3611 | 7.26685 | 7.202695 | 7.483623 | 7.045572 | 5.959117 | 7.6791 | 5.019387 | 2.70723 | low |
| GSM437094 | 10.22666667 | 0 | 7.637985 | 6.444664 | 6.746091 | 6.532566 | 7.707668 | 7.111636 | 7.675804 | 7.293147 | 5.687152 | 7.412958 | 5.079248 | 2.774663 | low |
| GSM437095 | 2.413333333 | 1 | 7.707222 | 7.3598 | 7.301243 | 7.863086 | 7.287399 | 7.180193 | 7.534387 | 7.148077 | 6.81777 | 7.411325 | 4.845771 | 2.726194 | low |
| GSM437096 | 9.934166667 | 0 | 9.068898 | 6.523849 | 7.250362 | 8.122776 | 7.730921 | 6.830055 | 7.80045 | 7.630786 | 5.487141 | 8.034567 | 4.869688 | 2.666111 | low |
| GSM437097 | 4.960833333 | 0 | 7.146444 | 6.384102 | 7.64029 | 7.516311 | 7.324411 | 7.080364 | 8.932026 | 7.50184 | 7.6575 | 7.731192 | 4.904691 | 2.8823 | low |
| GSM437098 | 5.734166667 | 1 | 7.180739 | 6.557424 | 8.058485 | 6.399887 | 7.454513 | 7.1966 | 8.186196 | 7.015237 | 8.281061 | 7.803225 | 5.396251 | 2.897651 | high |
| GSM437099 | 6.8575 | 0 | 7.082547 | 6.288588 | 7.078962 | 5.982957 | 7.355539 | 7.238774 | 8.257929 | 7.693606 | 7.410277 | 7.599282 | 4.90566 | 2.836306 | low |
| GSM437100 | 9.235 | 0 | 7.338882 | 6.074596 | 7.566672 | 7.505709 | 7.475041 | 7.011107 | 7.982498 | 7.352703 | 7.040005 | 7.76727 | 5.347118 | 2.852317 | low |
| GSM437101 | 5.49 | 1 | 6.957428 | 7.053232 | 7.116688 | 5.884598 | 7.441821 | 7.124529 | 7.47985 | 7.058401 | 8.289916 | 7.372579 | 7.514866 | 3.014231 | high |
| GSM437102 | 7.010833333 | 0 | 7.252938 | 6.581585 | 9.711371 | 5.759853 | 7.332978 | 7.609851 | 8.622938 | 7.553887 | 7.617276 | 8.207416 | 5.296506 | 2.934661 | high |
| GSM437103 | 1.3725 | 1 | 8.949036 | 6.912689 | 8.139094 | 6.609905 | 7.566325 | 7.149444 | 7.906611 | 7.275583 | 6.608629 | 7.728583 | 4.870249 | 2.628142 | low |
| GSM437104 | 7.479166667 | 0 | 7.10439 | 6.293529 | 7.342787 | 5.943995 | 7.113468 | 7.352785 | 6.815111 | 7.607515 | 6.645634 | 7.851202 | 4.807053 | 2.730724 | low |
| GSM437105 | 6.339166667 | 0 | 7.402041 | 6.422108 | 7.378527 | 6.137033 | 7.242948 | 7.303447 | 8.110598 | 7.262175 | 6.335339 | 7.517506 | 5.361716 | 2.765203 | low |
| GSM437106 | 5.651666667 | 0 | 8.000272 | 6.101017 | 7.38371 | 6.503736 | 7.262679 | 7.806043 | 7.460137 | 7.637227 | 7.309329 | 7.602325 | 5.052245 | 2.668349 | low |
| GSM437107 | 4.353333333 | 0 | 7.234914 | 6.202129 | 7.085997 | 7.1869 | 7.167453 | 7.301491 | 7.454386 | 6.854597 | 6.933077 | 7.803933 | 5.169409 | 2.751492 | low |
| GSM437108 | 5.341666667 | 0 | 6.926127 | 6.278592 | 7.32277 | 6.519545 | 7.256225 | 6.925674 | 7.205844 | 7.552613 | 7.057273 | 7.22844 | 5.263662 | 2.778322 | low |
| GSM437109 | 4.594166667 | 0 | 7.222064 | 6.683517 | 7.987188 | 8.837532 | 7.449993 | 7.40021 | 8.024756 | 7.115116 | 7.174517 | 7.649087 | 8.061383 | 3.085354 | high |
| GSM437110 | 4.815833333 | 0 | 7.387346 | 6.607421 | 7.251778 | 6.729559 | 7.529296 | 7.190077 | 8.21342 | 7.186566 | 6.599016 | 7.415948 | 6.338746 | 2.902859 | high |
| GSM437111 | 4.175 | 0 | 7.466364 | 6.209677 | 8.726689 | 7.041471 | 7.889034 | 7.525553 | 9.130096 | 7.45449 | 6.259957 | 8.711237 | 5.054026 | 3.044785 | high |
| GSM437112 | 3.649166667 | 0 | 7.263042 | 6.864061 | 8.013928 | 5.584118 | 7.589593 | 7.044864 | 7.756037 | 6.558895 | 6.295355 | 7.399029 | 4.760892 | 2.771558 | low |
| GSM437113 | 2.5225 | 0 | 7.505946 | 7.148915 | 7.320244 | 7.333915 | 7.489349 | 7.118198 | 8.353947 | 6.368655 | 6.598921 | 7.725567 | 4.72353 | 2.798058 | low |
| GSM437114 | 0.303333333 | 0 | 7.119573 | 6.352178 | 7.124854 | 7.239392 | 7.134372 | 7.097259 | 8.199317 | 6.844791 | 8.958687 | 7.581907 | 5.844227 | 2.846279 | low |
| GSM437115 | 3.049166667 | 0 | 7.239261 | 6.528638 | 6.73738 | 7.726329 | 7.586462 | 7.255925 | 8.668067 | 6.788472 | 6.76076 | 7.839011 | 7.314618 | 3.054207 | high |
| GSM437116 | 1.668333333 | 0 | 7.353976 | 6.647552 | 7.644611 | 6.632479 | 7.732317 | 7.22646 | 7.946159 | 7.185071 | 6.625811 | 7.885053 | 5.124075 | 2.902558 | high |
| GSM437117 | 9.881666667 | 0 | 7.14312 | 6.514794 | 9.132502 | 7.31747 | 7.704186 | 7.58961 | 7.881918 | 7.457631 | 7.152095 | 7.633609 | 5.143694 | 2.934704 | high |
| GSM437118 | 4.610833333 | 0 | 7.147025 | 8.012764 | 8.248233 | 6.106669 | 7.848455 | 7.280999 | 8.423528 | 8.001971 | 7.591619 | 8.053293 | 5.305316 | 3.109979 | high |
| GSM437119 | 11.23833333 | 1 | 6.928793 | 6.44654 | 8.720221 | 7.192714 | 7.816143 | 7.063026 | 9.096793 | 7.922516 | 6.89501 | 8.498073 | 6.212004 | 3.199517 | high |
| GSM437120 | 10.6075 | 0 | 9.326397 | 6.626926 | 7.247028 | 9.083343 | 7.3888 | 7.050667 | 8.02909 | 7.123075 | 7.524894 | 7.7121 | 8.247483 | 2.793993 | low |
| GSM437121 | 10.05166667 | 0 | 7.42696 | 6.478059 | 8.166023 | 7.16659 | 7.391968 | 7.392998 | 8.32444 | 7.32008 | 7.118907 | 7.577914 | 5.385341 | 2.842295 | low |
| GSM437122 | 8.911666667 | 0 | 6.945621 | 6.128433 | 7.806308 | 8.125856 | 7.4235 | 7.010565 | 7.379343 | 7.276698 | 7.17218 | 7.62885 | 4.900837 | 2.838019 | low |
| GSM437123 | 0.975 | 1 | 8.900925 | 6.917171 | 7.553244 | 6.983802 | 7.198726 | 7.397054 | 7.717856 | 7.100268 | 7.627548 | 7.500477 | 4.642066 | 2.522018 | low |
| GSM437124 | 2.399166667 | 1 | 6.993808 | 5.895468 | 7.988351 | 6.558701 | 7.684368 | 7.788866 | 8.315385 | 7.76435 | 9.064555 | 7.860574 | 4.729796 | 2.955227 | high |
| GSM437125 | 2.235 | 1 | 7.219566 | 6.250022 | 8.655158 | 7.153828 | 7.7129 | 7.555449 | 9.276413 | 7.756097 | 8.491447 | 8.511858 | 4.966516 | 3.066462 | high |
| GSM437126 | 4.375 | 1 | 7.315092 | 6.438967 | 7.497238 | 6.133676 | 7.492805 | 7.514484 | 8.6891 | 7.391074 | 7.994351 | 7.751027 | 4.967523 | 2.876561 | low |
| GSM437127 | 2.0025 | 1 | 8.578874 | 6.433949 | 8.015486 | 6.535266 | 7.709975 | 7.411758 | 9.039518 | 7.549954 | 8.357998 | 7.778821 | 5.469735 | 2.814585 | low |
| GSM437128 | 7.415833333 | 0 | 6.955284 | 6.444899 | 7.130743 | 7.238527 | 7.338134 | 7.141113 | 8.547578 | 7.07571 | 7.422705 | 7.548193 | 6.694243 | 2.978117 | high |
| GSM437129 | 8.536666667 | 0 | 7.158782 | 6.314616 | 7.716447 | 7.177176 | 7.180334 | 7.117416 | 8.223372 | 6.976144 | 7.181613 | 7.55529 | 6.074679 | 2.850741 | low |
| GSM437130 | 2.810833333 | 1 | 7.226478 | 6.42002 | 8.562621 | 6.148426 | 7.536144 | 8.198021 | 8.364969 | 7.914773 | 8.338292 | 8.414903 | 5.230913 | 3.005905 | high |
| GSM437131 | 7.106666667 | 0 | 7.558491 | 6.451439 | 6.78926 | 6.876124 | 7.530228 | 7.304339 | 8.366286 | 6.991771 | 6.517247 | 7.400543 | 4.790663 | 2.760216 | low |
| GSM437132 | 2.210833333 | 0 | 6.875604 | 6.862675 | 7.085238 | 8.079555 | 7.364946 | 7.037195 | 8.152942 | 7.128027 | 6.366308 | 7.872759 | 4.938229 | 2.906272 | high |
| GSM437133 | 3.109166667 | 1 | 7.20555 | 6.831997 | 7.991659 | 5.943583 | 7.445781 | 7.681479 | 8.23739 | 7.906587 | 7.662932 | 7.902625 | 6.026153 | 2.990049 | high |
| GSM437134 | 7.2025 | 0 | 7.921142 | 6.72077 | 8.603632 | 6.75903 | 7.792463 | 8.290028 | 8.614676 | 7.409354 | 8.262472 | 8.805514 | 5.347239 | 3.017288 | high |
| GSM437135 | 4.66 | 0 | 7.254755 | 6.33049 | 9.44889 | 6.895507 | 7.769871 | 7.989284 | 8.210837 | 7.726194 | 7.238039 | 8.405398 | 6.072825 | 3.089291 | high |
| GSM437136 | 5.8875 | 0 | 7.141626 | 6.746563 | 7.115766 | 8.91634 | 7.697053 | 7.226001 | 8.472057 | 7.097577 | 7.23525 | 7.722298 | 5.114224 | 2.965312 | high |
| GSM437137 | 6.336666667 | 0 | 7.054748 | 6.498157 | 7.451552 | 6.94278 | 7.430652 | 6.844099 | 7.699023 | 7.197841 | 7.11517 | 7.651613 | 5.329829 | 2.862567 | low |
| GSM437138 | 6.594166667 | 0 | 7.025706 | 6.18851 | 7.452728 | 6.526488 | 7.308674 | 7.66763 | 7.627266 | 7.74829 | 5.677815 | 7.902406 | 4.927416 | 2.830311 | low |
| GSM437139 | 6.999166667 | 0 | 7.025277 | 6.505798 | 7.370722 | 7.723572 | 7.532272 | 6.943892 | 7.798243 | 7.172023 | 5.978671 | 7.506325 | 4.939285 | 2.847411 | low |
| GSM437140 | 6.6875 | 0 | 7.321675 | 6.491291 | 7.461537 | 6.382986 | 7.477212 | 7.162603 | 7.451615 | 7.358357 | 7.246859 | 7.417277 | 4.850922 | 2.772954 | low |
| GSM437141 | 3.383333333 | 0 | 7.233948 | 8.97611 | 7.318909 | 7.003954 | 7.299621 | 7.188492 | 7.918195 | 7.161107 | 8.099402 | 7.646 | 5.000497 | 2.921959 | high |
| GSM437142 | 7.9475 | 0 | 7.025191 | 6.090229 | 7.519623 | 6.254586 | 7.69107 | 7.219272 | 7.106403 | 7.250825 | 9.282605 | 7.356578 | 5.04404 | 2.852363 | low |
| GSM437143 | 2.588333333 | 1 | 8.391357 | 6.537297 | 7.595325 | 7.472286 | 7.143063 | 7.17194 | 7.607695 | 7.338379 | 6.869662 | 7.579229 | 4.882695 | 2.590258 | low |
| GSM437144 | 3.109166667 | 1 | 8.528401 | 6.28564 | 8.120364 | 7.917565 | 7.610406 | 7.411951 | 7.616027 | 7.865771 | 6.531241 | 7.64151 | 5.684838 | 2.748486 | low |
| GSM437145 | 1.835 | 1 | 7.033882 | 6.616275 | 8.879198 | 6.92108 | 7.506172 | 7.934533 | 8.324584 | 7.742776 | 8.19708 | 8.11031 | 5.485607 | 3.022803 | high |
| GSM437146 | 5.270833333 | 0 | 7.788663 | 6.445307 | 7.063362 | 6.38046 | 7.262855 | 7.105411 | 7.533554 | 7.210906 | 5.866559 | 7.515979 | 4.989516 | 2.657917 | low |
| GSM437147 | 2.840833333 | 0 | 6.863913 | 7.808346 | 7.507992 | 6.189247 | 7.248736 | 6.893836 | 8.596365 | 7.995167 | 8.56013 | 7.971088 | 5.607472 | 3.040633 | high |
| GSM437148 | 4.345 | 0 | 10.45694 | 6.341788 | 7.988717 | 6.123047 | 7.326342 | 7.044069 | 8.087463 | 7.47363 | 6.069332 | 7.578296 | 5.102236 | 2.344329 | low |
| GSM437149 | 5.503333333 | 0 | 7.605098 | 6.724552 | 7.781815 | 6.673589 | 7.434831 | 7.83886 | 8.64522 | 7.726294 | 6.743872 | 7.930964 | 5.465755 | 2.898854 | high |
| GSM437150 | 4.996666667 | 0 | 7.611226 | 7.192352 | 7.9466 | 6.626036 | 7.739156 | 7.454208 | 7.893281 | 7.244356 | 6.399087 | 8.340871 | 4.984733 | 2.928374 | high |
| GSM437151 | 3.988333333 | 0 | 7.648201 | 6.525051 | 7.605048 | 5.916389 | 7.251599 | 7.096609 | 7.176485 | 7.244356 | 7.194395 | 7.672817 | 4.8354 | 2.682656 | low |
| GSM437152 | 4.870833333 | 0 | 8.462493 | 6.849657 | 7.545911 | 8.45039 | 7.669999 | 7.647113 | 9.134362 | 7.33922 | 7.781548 | 8.608535 | 5.262178 | 2.919359 | high |
| GSM437153 | 3.8325 | 0 | 7.566825 | 7.29895 | 7.737506 | 6.321757 | 7.288883 | 7.523356 | 6.337242 | 6.609163 | 6.842418 | 8.424056 | 4.840057 | 2.753259 | low |
| GSM437154 | 5.035 | 0 | 7.409394 | 6.780006 | 7.363619 | 8.650074 | 7.228453 | 7.270573 | 7.696708 | 6.61044 | 6.668405 | 8.037314 | 5.295954 | 2.813967 | low |
| GSM437155 | 3.139166667 | 0 | 7.108779 | 6.89166 | 8.200101 | 7.78907 | 7.371441 | 7.62708 | 8.683937 | 7.497406 | 6.730765 | 7.833292 | 5.426809 | 2.957515 | high |
| GSM437156 | 1.309166667 | 0 | 7.436393 | 6.670371 | 7.812076 | 5.601316 | 7.605362 | 8.015852 | 9.31533 | 7.716324 | 8.158449 | 7.587622 | 5.010638 | 2.922476 | high |
| GSM437157 | 3.270833333 | 0 | 7.687338 | 6.3708 | 7.838116 | 8.453538 | 7.434831 | 7.240751 | 8.489283 | 7.344721 | 7.641478 | 7.784809 | 4.966112 | 2.827988 | low |
| GSM437158 | 4.155833333 | 0 | 7.314731 | 6.650178 | 7.898857 | 7.439479 | 7.478088 | 7.017231 | 7.53884 | 8.000207 | 7.46841 | 7.717818 | 4.93957 | 2.865458 | low |
| GSM437159 | 2.103333333 | 0 | 7.298191 | 6.504757 | 8.003428 | 6.331153 | 7.610204 | 7.239642 | 7.360057 | 6.849948 | 6.860749 | 7.884297 | 5.177746 | 2.844715 | low |
| GSM437160 | 3.7475 | 0 | 7.30349 | 6.236628 | 7.826735 | 5.736482 | 7.509703 | 7.151201 | 7.96544 | 8.290913 | 7.354527 | 7.567978 | 4.958612 | 2.849069 | low |
| GSM437161 | 2.991666667 | 0 | 7.084593 | 6.399722 | 8.846811 | 7.150895 | 7.22303 | 7.250036 | 8.296369 | 7.496147 | 6.598838 | 7.813419 | 5.284005 | 2.870215 | low |
| GSM437162 | 2.221666667 | 0 | 7.121023 | 6.607457 | 7.498986 | 8.50982 | 7.330097 | 7.239395 | 8.369339 | 7.37583 | 6.942939 | 7.634413 | 4.935829 | 2.866825 | low |
| GSM437163 | 3.065 | 0 | 7.548049 | 6.904313 | 7.323128 | 8.066823 | 7.66359 | 7.799613 | 8.667594 | 7.118718 | 6.501521 | 8.354255 | 5.241026 | 2.974087 | high |
| GSM437164 | 3.46 | 0 | 7.496123 | 6.480925 | 7.112614 | 8.365397 | 7.298629 | 7.24815 | 8.468628 | 6.788472 | 7.456305 | 8.024211 | 5.075997 | 2.82956 | low |
| GSM437165 | 1.851666667 | 0 | 7.801205 | 6.615726 | 7.317761 | 5.446255 | 7.311084 | 7.054358 | 7.410339 | 6.192948 | 6.251849 | 7.433667 | 4.703505 | 2.59008 | low |
| GSM437166 | 2.766666667 | 0 | 8.662629 | 6.498289 | 7.668342 | 6.830196 | 7.600282 | 7.301574 | 7.713076 | 6.887267 | 7.500318 | 7.903579 | 6.479181 | 2.772206 | low |
| GSM437167 | 3.306666667 | 0 | 7.029616 | 6.640342 | 7.733536 | 6.415733 | 7.292127 | 7.055832 | 8.440921 | 7.256375 | 7.600712 | 7.75743 | 4.73597 | 2.848628 | low |
| GSM437168 | 2.134166667 | 0 | 7.196196 | 6.46382 | 7.613739 | 6.038418 | 7.573546 | 7.028655 | 7.911026 | 7.089315 | 5.776425 | 7.597862 | 7.049341 | 2.964065 | high |
| GSM437169 | 6.999166667 | 0 | 7.023612 | 6.68281 | 7.190048 | 6.882088 | 7.718469 | 7.236379 | 8.461762 | 7.205932 | 6.532012 | 7.668904 | 5.094698 | 2.946589 | high |
| GSM437170 | 1.254166667 | 0 | 7.255643 | 6.350622 | 7.127088 | 7.466035 | 7.40565 | 7.148552 | 7.386097 | 7.174733 | 6.026714 | 7.872105 | 6.301465 | 2.89239 | low |
| GSM437171 | 0.698333333 | 0 | 8.117091 | 6.459246 | 7.85548 | 7.729944 | 7.376112 | 7.027136 | 8.384007 | 7.799641 | 6.886589 | 7.828225 | 5.136401 | 2.770503 | low |
| GSM437172 | 1.515 | 0 | 7.136194 | 6.178843 | 7.858307 | 7.432001 | 7.40019 | 7.075755 | 8.300152 | 7.161021 | 6.908116 | 7.967951 | 5.300332 | 2.890668 | low |
| GSM437173 | 2.2625 | 1 | 7.080917 | 6.255897 | 7.798011 | 7.701014 | 7.342212 | 8.596391 | 7.765649 | 7.908562 | 7.531229 | 8.21974 | 5.997387 | 2.998727 | high |
| GSM437174 | 2.021666667 | 1 | 7.724029 | 6.430326 | 7.705071 | 5.830541 | 7.839248 | 7.932484 | 8.663359 | 8.177081 | 6.964556 | 8.870694 | 5.17373 | 3.034287 | high |
| GSM437175 | 8.764166667 | 0 | 7.095765 | 6.108127 | 8.640668 | 7.332074 | 7.461003 | 7.940079 | 8.125142 | 7.743357 | 8.170076 | 8.259973 | 7.704125 | 3.145602 | high |
| GSM437176 | 0.98 | 1 | 7.764374 | 6.586671 | 7.608459 | 6.509717 | 7.531708 | 6.758468 | 7.941638 | 8.183037 | 7.985046 | 8.046406 | 8.218714 | 3.083701 | high |
| GSM437177 | 5.870833333 | 1 | 7.019788 | 6.267931 | 8.204376 | 7.124773 | 8.020442 | 6.970261 | 7.948661 | 7.563602 | 6.875687 | 7.821451 | 5.089565 | 3.009957 | high |
| GSM437178 | 7.9225 | 0 | 7.306873 | 6.195361 | 8.353918 | 7.94222 | 7.475824 | 7.516264 | 8.235083 | 7.350914 | 7.233501 | 7.738967 | 5.362666 | 2.889095 | low |
| GSM437179 | 9.360833333 | 0 | 7.299967 | 6.400818 | 7.635575 | 6.928986 | 7.66025 | 7.228371 | 7.919107 | 7.423495 | 7.52792 | 7.674613 | 4.804599 | 2.865471 | low |
| GSM437180 | 2.2925 | 1 | 7.263042 | 6.219005 | 7.703052 | 6.023338 | 7.551138 | 7.240127 | 7.551859 | 7.834675 | 7.132388 | 8.01503 | 5.133073 | 2.881866 | low |
| GSM437181 | 3.826666667 | 1 | 7.823919 | 6.647552 | 8.574764 | 7.326757 | 7.648616 | 7.601639 | 7.934358 | 7.352703 | 6.344124 | 8.189152 | 5.736357 | 2.913111 | high |
| GSM437182 | 4.503333333 | 1 | 7.13949 | 6.385178 | 8.158001 | 6.989898 | 7.74437 | 7.260424 | 8.525389 | 7.602182 | 6.627645 | 8.390989 | 4.785728 | 2.998383 | high |
| GSM437183 | 8.665 | 0 | 7.351523 | 6.300524 | 7.748431 | 6.911312 | 7.520125 | 7.117172 | 7.757797 | 7.581264 | 7.023458 | 7.661392 | 4.919909 | 2.825079 | low |
| GSM437184 | 0.509166667 | 1 | 6.784337 | 7.06401 | 7.978808 | 6.09205 | 7.511346 | 8.331822 | 9.266737 | 7.797841 | 9.162195 | 8.055176 | 4.921484 | 3.07497 | high |
| GSM437185 | 8.2925 | 0 | 7.614366 | 6.835779 | 6.956638 | 8.860809 | 7.806934 | 7.161162 | 7.682743 | 7.563864 | 6.401304 | 7.693149 | 4.996133 | 2.890257 | low |
| GSM437186 | 1.703333333 | 1 | 7.267612 | 6.110183 | 7.263394 | 7.212964 | 7.418402 | 6.919551 | 7.888772 | 7.646477 | 7.078085 | 7.996099 | 5.656448 | 2.898311 | high |
| GSM437187 | 6.383333333 | 1 | 9.569508 | 6.524476 | 8.055577 | 6.664447 | 7.859666 | 7.529906 | 7.734406 | 7.640194 | 7.378937 | 8.32641 | 5.324699 | 2.694751 | low |
| GSM437188 | 0.835 | 1 | 7.264682 | 6.199226 | 8.266044 | 6.788937 | 7.390184 | 7.494643 | 8.430451 | 7.344453 | 6.395386 | 7.819598 | 4.947993 | 2.838361 | low |
| GSM437189 | 3.454166667 | 1 | 7.032211 | 6.48479 | 8.13667 | 5.911917 | 7.648026 | 7.914138 | 8.352986 | 7.51954 | 7.875301 | 7.774711 | 4.75997 | 2.933488 | high |
| GSM437190 | 8.021666667 | 0 | 7.026401 | 6.413864 | 8.784879 | 6.825249 | 7.841269 | 7.995606 | 8.161953 | 7.412622 | 7.333889 | 8.255774 | 4.935028 | 3.027116 | high |
| GSM437191 | 7.134166667 | 0 | 7.551317 | 6.359447 | 7.688667 | 7.456696 | 7.384074 | 7.088499 | 8.269364 | 7.3003 | 5.816874 | 7.943474 | 4.974848 | 2.803742 | low |
| GSM437192 | 6.5725 | 0 | 7.266067 | 6.099929 | 7.521782 | 6.671733 | 7.385124 | 7.528453 | 7.991917 | 7.724386 | 7.23142 | 7.925612 | 4.916774 | 2.843115 | low |
| GSM437193 | 1.854166667 | 1 | 7.015373 | 6.439786 | 7.300476 | 5.912915 | 7.207803 | 6.834912 | 8.067929 | 7.218138 | 8.218317 | 7.68291 | 5.440405 | 2.844603 | low |
| GSM437194 | 6.210833333 | 0 | 9.163908 | 6.393422 | 7.3779 | 6.727236 | 7.225919 | 7.314046 | 8.098374 | 7.085861 | 6.650477 | 7.790459 | 6.12919 | 2.596281 | low |
| GSM437195 | 4.9225 | 1 | 7.327558 | 6.541848 | 8.358089 | 6.87465 | 7.493009 | 7.431429 | 8.15559 | 6.886727 | 7.041027 | 7.915274 | 7.459807 | 3.027302 | high |
| GSM437196 | 3.983333333 | 1 | 7.054016 | 6.625087 | 7.822683 | 7.042639 | 7.598265 | 7.783801 | 8.252724 | 7.454063 | 6.727217 | 7.734403 | 4.963143 | 2.927972 | high |
| GSM437197 | 0.473333333 | 1 | 9.100565 | 7.429488 | 7.420346 | 6.16791 | 7.493507 | 7.798956 | 9.21158 | 8.04798 | 8.039169 | 7.992995 | 5.145441 | 2.760338 | low |
| GSM437198 | 6.615833333 | 0 | 6.981359 | 5.928536 | 7.746939 | 8.176098 | 7.117091 | 7.120281 | 8.490184 | 7.62695 | 7.577871 | 7.924665 | 5.573873 | 2.902981 | high |
| GSM437199 | 6.1725 | 0 | 7.496391 | 6.457869 | 7.609206 | 8.65079 | 7.455464 | 7.770564 | 8.295957 | 7.718089 | 5.68385 | 8.015151 | 6.10957 | 2.956125 | high |
| GSM437200 | 4.23 | 1 | 7.168058 | 6.390647 | 8.199229 | 6.430442 | 7.833381 | 7.810588 | 8.964415 | 7.96711 | 8.048247 | 8.575528 | 4.851518 | 3.084357 | high |
| GSM437201 | 5.651666667 | 1 | 7.309747 | 6.759023 | 10.06167 | 7.494195 | 7.409171 | 8.007697 | 9.642557 | 7.997033 | 6.673443 | 8.059293 | 5.28203 | 3.014535 | high |
| GSM437202 | 5.903333333 | 0 | 7.181687 | 6.553685 | 7.653897 | 6.560778 | 7.485655 | 7.565781 | 9.338772 | 7.905708 | 8.432338 | 8.069934 | 5.046335 | 2.99642 | high |
| GSM437203 | 4.215833333 | 0 | 7.596354 | 7.080573 | 8.260336 | 6.251502 | 7.38783 | 7.02949 | 8.436699 | 7.527719 | 6.521379 | 7.778602 | 7.034132 | 2.97012 | high |
| GSM437204 | 2.881666667 | 1 | 6.844218 | 6.974187 | 7.989429 | 11.75112 | 7.069085 | 7.411591 | 9.808846 | 7.433706 | 6.908794 | 7.986017 | 8.102999 | 3.230981 | high |
| GSM437205 | 3.226666667 | 1 | 7.915342 | 6.467858 | 7.846812 | 7.177578 | 7.546546 | 7.359747 | 8.091434 | 7.215816 | 5.692363 | 8.709759 | 5.030654 | 2.855964 | low |
| GSM437206 | 4.575 | 1 | 7.116187 | 6.366098 | 7.589595 | 7.196008 | 7.583119 | 7.23843 | 7.886159 | 7.23807 | 7.279416 | 7.604961 | 5.740492 | 2.92305 | high |
| GSM437207 | 5.66 | 0 | 6.963363 | 6.371308 | 8.201906 | 6.515162 | 7.499853 | 7.060978 | 7.514236 | 7.397889 | 6.163629 | 7.969123 | 5.043531 | 2.88538 | low |
| GSM437208 | 4.870833333 | 0 | 7.760109 | 6.2278 | 6.944505 | 8.253767 | 7.360088 | 7.010812 | 8.628973 | 6.901557 | 8.339375 | 7.673 | 5.127959 | 2.780767 | low |
| GSM437209 | 4.945 | 0 | 7.234834 | 9.340413 | 7.636469 | 5.997914 | 7.638755 | 8.578504 | 8.960321 | 7.580703 | 8.567917 | 7.746969 | 5.318629 | 3.109552 | high |
| GSM437210 | 4.545 | 0 | 7.779978 | 6.198136 | 8.420723 | 6.810369 | 7.69352 | 7.396887 | 8.724667 | 8.173145 | 6.93492 | 7.91758 | 5.356761 | 2.921148 | high |
| GSM437211 | 1.585833333 | 1 | 7.119745 | 7.634079 | 7.840065 | 7.557723 | 7.436423 | 7.843139 | 8.567929 | 7.465483 | 7.84165 | 7.826662 | 5.298517 | 2.997393 | high |
| GSM437212 | 2.188333333 | 1 | 7.255466 | 9.73263 | 7.446956 | 5.826987 | 7.374297 | 8.145533 | 8.735119 | 7.565656 | 8.383535 | 7.904357 | 5.051995 | 3.046521 | high |
| GSM437213 | 4.405 | 0 | 7.570932 | 6.280468 | 7.113126 | 8.837216 | 7.499954 | 7.194044 | 7.642191 | 7.483555 | 7.359104 | 7.471199 | 7.618365 | 2.975663 | high |
| GSM437214 | 2.670833333 | 1 | 8.054672 | 6.611575 | 7.748325 | 7.478018 | 7.457026 | 7.349507 | 7.957471 | 7.097892 | 5.361164 | 7.878355 | 4.889192 | 2.723753 | low |
| GSM437215 | 4.6625 | 0 | 7.783539 | 6.573251 | 7.951303 | 7.543077 | 7.626472 | 7.608745 | 8.676557 | 7.110007 | 6.832668 | 8.467425 | 7.49333 | 3.084447 | high |
| GSM437216 | 4.7225 | 0 | 9.596289 | 6.511335 | 7.055479 | 6.790101 | 6.89715 | 7.014729 | 7.805874 | 7.038614 | 6.64078 | 7.220005 | 4.818227 | 2.309129 | low |
| GSM437217 | 4.2025 | 0 | 7.275894 | 6.372136 | 7.447132 | 6.921486 | 7.288588 | 6.910493 | 7.600693 | 6.623656 | 7.200209 | 7.42129 | 5.340201 | 2.747579 | low |
| GSM437218 | 0.353333333 | 1 | 7.528203 | 6.490977 | 7.486858 | 7.12536 | 7.486244 | 7.269408 | 9.437636 | 7.543555 | 7.767197 | 8.1414 | 6.309633 | 3.023638 | high |
| GSM437219 | 4.0925 | 0 | 7.074239 | 6.460967 | 7.024478 | 7.774914 | 7.30062 | 7.032187 | 7.854303 | 7.593384 | 6.631838 | 7.641418 | 4.736109 | 2.817291 | low |
| GSM437220 | 4.130833333 | 0 | 8.236275 | 6.658256 | 7.094646 | 5.593769 | 7.532191 | 7.209702 | 7.495151 | 7.077023 | 6.96894 | 7.549655 | 4.965066 | 2.658405 | low |
| GSM437221 | 4.2075 | 0 | 7.374625 | 6.499583 | 7.31928 | 6.39424 | 7.881865 | 7.050821 | 8.1181 | 7.136353 | 6.690852 | 7.903355 | 5.154898 | 2.926563 | high |
| GSM437222 | 5.254166667 | 0 | 11.76061 | 6.839909 | 7.716971 | 6.140468 | 7.624113 | 6.865736 | 8.718366 | 7.265832 | 6.631322 | 7.735182 | 4.941806 | 2.269954 | low |
| GSM437223 | 4.103333333 | 1 | 7.163774 | 6.539132 | 8.580505 | 6.480736 | 7.578625 | 7.567879 | 8.738694 | 7.873059 | 7.804241 | 8.179917 | 5.562818 | 3.034855 | high |
| GSM437225 | 2.613333333 | 0 | 7.288373 | 9.05672 | 8.117093 | 8.138492 | 7.594302 | 7.348082 | 8.582607 | 7.191253 | 8.533149 | 8.072082 | 5.860214 | 3.134589 | high |
| GSM437226 | 3.358333333 | 0 | 9.110868 | 7.740729 | 7.93167 | 7.394433 | 7.631733 | 7.294307 | 8.906326 | 6.729272 | 6.922243 | 8.671346 | 5.815576 | 2.844251 | low |
| GSM437227 | 9.72 | 0 | 6.908759 | 6.684572 | 7.689006 | 5.947747 | 7.499075 | 7.410835 | 7.798134 | 7.222053 | 7.320292 | 8.35464 | 4.835678 | 2.94022 | high |
| GSM437228 | 2.66 | 0 | 6.992324 | 6.344456 | 7.351383 | 5.972581 | 7.494282 | 6.905057 | 8.312021 | 7.328317 | 7.522823 | 7.587807 | 4.859558 | 2.86168 | low |
| GSM437229 | 1.665 | 0 | 7.440091 | 6.699763 | 8.003812 | 6.678387 | 7.809928 | 7.258826 | 8.102006 | 7.690809 | 7.564197 | 8.222489 | 5.649031 | 3.020184 | high |
| GSM437230 | 2.051666667 | 0 | 8.457535 | 6.254322 | 6.94553 | 5.533327 | 7.493265 | 6.977876 | 7.763943 | 7.336046 | 7.623371 | 7.655367 | 4.619407 | 2.613153 | low |
| GSM437231 | 1.545 | 1 | 7.789496 | 6.670303 | 7.699526 | 7.721778 | 7.584197 | 7.256697 | 8.849597 | 7.181462 | 7.358136 | 7.752098 | 5.198156 | 2.865164 | low |
| GSM437232 | 10.89583333 | 0 | 8.432254 | 6.702521 | 7.178844 | 7.06241 | 7.45767 | 7.065588 | 8.340218 | 7.158512 | 6.685094 | 7.952488 | 7.807359 | 2.90697 | high |
| GSM437233 | 1.468333333 | 1 | 7.058968 | 6.209481 | 7.650449 | 7.443932 | 7.06675 | 7.338316 | 8.122111 | 7.194974 | 7.545247 | 7.68291 | 8.892018 | 3.059274 | high |
| GSM437234 | 0.443333333 | 1 | 7.634773 | 6.518344 | 9.099639 | 7.344173 | 7.50557 | 7.55286 | 8.090667 | 7.602725 | 7.64953 | 8.00182 | 5.335525 | 2.89408 | high |
| GSM437235 | 0.196666667 | 1 | 7.01095 | 6.019942 | 9.602521 | 7.103248 | 7.825965 | 8.090797 | 8.426914 | 7.703656 | 7.901657 | 8.352862 | 5.904004 | 3.12467 | high |
| GSM437236 | 0.476666667 | 1 | 7.131658 | 6.210633 | 7.425251 | 5.900624 | 7.564687 | 7.181127 | 8.605992 | 7.481614 | 8.158213 | 7.737977 | 6.03318 | 2.975025 | high |
| GSM437237 | 1.273333333 | 1 | 7.362143 | 6.442313 | 7.486091 | 8.193633 | 7.435864 | 7.071166 | 8.44891 | 7.252264 | 6.549131 | 7.588198 | 6.155022 | 2.9149 | high |
| GSM437238 | 7.9225 | 0 | 7.341442 | 6.471022 | 7.117078 | 6.486064 | 7.491756 | 7.317183 | 8.547889 | 7.379152 | 5.978931 | 7.760576 | 5.009608 | 2.848196 | low |
| GSM437239 | 0.413333333 | 1 | 6.980837 | 6.110802 | 8.879055 | 7.027149 | 7.726413 | 7.637858 | 8.264014 | 7.585204 | 7.75688 | 8.160992 | 5.759097 | 3.060483 | high |
| GSM437240 | 2.4075 | 1 | 7.242508 | 6.423583 | 7.483507 | 6.378894 | 7.626875 | 7.286584 | 7.86632 | 7.523268 | 7.32616 | 7.423471 | 5.009247 | 2.849996 | low |
| GSM437241 | 0.270833333 | 1 | 7.165453 | 6.483101 | 7.915991 | 7.660091 | 7.755537 | 7.771959 | 8.192587 | 7.509056 | 6.819034 | 8.108541 | 6.233623 | 3.071988 | high |
| GSM437243 | 1.564166667 | 1 | 6.64468 | 6.133209 | 8.332926 | 6.425317 | 7.637984 | 7.613835 | 8.186654 | 7.553637 | 7.407987 | 7.91621 | 5.207368 | 3.008443 | high |
| GSM437244 | 4.810833333 | 1 | 7.30016 | 5.986078 | 7.555747 | 6.302862 | 7.671943 | 7.146269 | 8.257992 | 7.422024 | 6.729358 | 7.806276 | 4.804467 | 2.856566 | low |
| GSM437245 | 1.199166667 | 1 | 7.189088 | 6.339152 | 7.872825 | 6.758755 | 7.478409 | 7.480185 | 8.352843 | 7.252598 | 6.717796 | 8.038468 | 5.277983 | 2.911063 | high |
| GSM437246 | 0.426666667 | 1 | 6.863913 | 6.399323 | 7.855365 | 6.825177 | 7.680533 | 7.173884 | 8.030621 | 7.488923 | 7.306406 | 7.427358 | 8.416276 | 3.162002 | high |
| GSM437247 | 0.415833333 | 1 | 6.964436 | 6.36372 | 7.675983 | 6.168081 | 7.379704 | 6.958429 | 8.402508 | 7.406994 | 7.232106 | 7.933782 | 4.837398 | 2.883222 | low |
| GSM437248 | 2.764166667 | 1 | 7.056629 | 6.150613 | 7.885538 | 6.640302 | 7.704404 | 7.483137 | 8.414613 | 7.637227 | 7.784642 | 7.681417 | 6.759972 | 3.066768 | high |
| GSM437249 | 4.210833333 | 1 | 7.049092 | 6.257114 | 7.760049 | 7.610556 | 7.535207 | 7.291155 | 8.038314 | 7.42949 | 7.035898 | 7.468196 | 9.515269 | 3.185328 | high |
| GSM437250 | 0.476666667 | 1 | 6.941745 | 6.45895 | 8.386789 | 8.518946 | 7.52794 | 7.413672 | 8.652466 | 7.515069 | 6.052715 | 7.883852 | 6.654107 | 3.083738 | high |
| GSM437251 | 0.668333333 | 1 | 8.453561 | 6.290145 | 7.299767 | 9.382128 | 6.871266 | 7.191014 | 8.503712 | 7.350871 | 6.988174 | 7.879226 | 5.427219 | 2.643297 | low |
| GSM437252 | 2.835 | 1 | 7.31771 | 6.786269 | 7.689656 | 6.520847 | 7.607597 | 7.299176 | 7.457026 | 7.3803 | 5.853853 | 8.16133 | 4.84909 | 2.87428 | low |
| GSM437253 | 0.12 | 1 | 7.117465 | 6.466284 | 7.585983 | 7.068205 | 7.283959 | 7.734846 | 8.791132 | 7.966577 | 8.393302 | 7.778713 | 5.18534 | 2.929163 | high |
| GSM437254 | 3.079166667 | 1 | 6.959367 | 8.45139 | 6.892093 | 7.030037 | 7.773253 | 7.157883 | 7.615102 | 7.337948 | 7.240828 | 7.393416 | 6.984087 | 3.130411 | high |
| GSM437255 | 5.0625 | 0 | 7.602446 | 6.625722 | 6.886053 | 6.828179 | 7.387965 | 7.273911 | 8.736652 | 7.052422 | 5.90298 | 7.421026 | 4.823082 | 2.747134 | low |
| GSM437256 | 3.12 | 1 | 7.183586 | 6.63152 | 7.43387 | 6.604778 | 7.750556 | 7.276358 | 7.86965 | 6.932345 | 6.630114 | 7.660212 | 5.900376 | 2.946625 | high |
| GSM437257 | 1.9225 | 1 | 6.891076 | 6.224871 | 7.091003 | 8.053928 | 7.251146 | 6.965026 | 8.306534 | 7.355633 | 8.047802 | 7.834046 | 6.73481 | 3.007684 | high |
| GSM437258 | 1.654166667 | 1 | 7.695402 | 6.563488 | 7.379555 | 6.028617 | 7.372995 | 7.112624 | 7.968029 | 7.566646 | 7.846093 | 8.069484 | 4.656348 | 2.783008 | low |
| GSM437259 | 2.654166667 | 0 | 7.714895 | 6.165339 | 7.115436 | 6.778141 | 7.425814 | 7.476894 | 8.0274 | 7.141233 | 5.946307 | 7.846792 | 4.862216 | 2.739661 | low |
| GSM437260 | 0.7175 | 1 | 7.23565 | 6.413785 | 7.611033 | 8.805422 | 7.451607 | 7.333373 | 9.665299 | 7.454581 | 7.965635 | 7.344747 | 5.113002 | 2.926105 | high |
| GSM437261 | 3.5225 | 0 | 7.362583 | 7.162342 | 7.661312 | 5.661509 | 7.28404 | 7.144714 | 7.574999 | 6.448164 | 7.165883 | 7.597081 | 4.634317 | 2.716116 | low |
| GSM437262 | 1.270833333 | 1 | 7.178366 | 6.206903 | 8.390283 | 7.581233 | 7.511945 | 7.851558 | 7.997517 | 7.350957 | 7.591722 | 8.121293 | 5.289955 | 2.939914 | high |
| GSM437263 | 1.213333333 | 1 | 6.900737 | 7.466619 | 8.642846 | 7.840893 | 7.459316 | 8.15106 | 8.209124 | 7.376856 | 8.338441 | 7.970971 | 5.711582 | 3.066645 | high |
| GSM437264 | 0.83 | 1 | 6.961174 | 6.265014 | 7.504832 | 6.365182 | 7.381619 | 7.396332 | 7.061832 | 7.254373 | 5.86443 | 7.585413 | 5.637405 | 2.829688 | low |
| GSM437265 | 0.745 | 1 | 7.857884 | 6.432458 | 8.193008 | 8.281696 | 7.264709 | 7.134667 | 7.865365 | 7.282301 | 7.233917 | 7.782343 | 9.600563 | 3.058797 | high |
| GSM437266 | 2.7225 | 0 | 7.485767 | 6.592124 | 7.473109 | 7.887797 | 7.458811 | 7.4354 | 8.45264 | 7.285151 | 6.577236 | 8.107013 | 5.484316 | 2.913703 | high |
| GSM437267 | 0.1725 | 1 | 7.356207 | 6.412627 | 7.55955 | 7.615809 | 7.455079 | 7.212782 | 7.208085 | 7.13358 | 6.543354 | 7.927582 | 7.029403 | 2.949365 | high |
| GSM437268 | 1.898333333 | 1 | 7.090432 | 6.498224 | 7.546917 | 6.371615 | 7.441821 | 6.858638 | 8.49286 | 6.619122 | 6.73025 | 8.311484 | 4.724603 | 2.877347 | low |
| GSM437269 | 1.8625 | 0 | 7.062643 | 6.166449 | 7.767594 | 6.106078 | 7.030284 | 6.898654 | 7.241097 | 7.397889 | 8.098439 | 7.59207 | 4.806748 | 2.714539 | low |
| GSM437271 | 5.326027398 | 0 | 9.453449 | 6.484832 | 7.233977 | 6.934247 | 7.200506 | 6.782841 | 7.670964 | 7.579631 | 7.330822 | 7.827159 | 5.386145 | 2.514822 | low |
| GSM437272 | 3.857534247 | 1 | 7.668509 | 7.425341 | 7.402157 | 7.369309 | 7.381602 | 7.317873 | 8.327244 | 7.883213 | 6.199411 | 7.613187 | 5.342995 | 2.860707 | low |
| GSM437273 | 5.002739726 | 0 | 7.20942 | 6.41581 | 7.414513 | 6.833345 | 7.389616 | 6.980698 | 7.726695 | 7.969005 | 7.187523 | 7.732186 | 5.355601 | 2.873352 | low |
| GSM437275 | 5.002739726 | 0 | 7.068698 | 6.501958 | 6.976765 | 7.436582 | 7.163533 | 6.923971 | 7.262825 | 7.273022 | 7.412433 | 7.709102 | 5.500748 | 2.816225 | low |
| GSM437276 | 5.046575343 | 0 | 7.711509 | 6.293552 | 7.130604 | 7.397293 | 7.804432 | 6.73426 | 7.354209 | 7.55899 | 5.953166 | 7.983585 | 5.519372 | 2.874014 | low |
| GSM437277 | 4.756164383 | 0 | 9.443996 | 6.379713 | 6.90003 | 6.689737 | 7.115711 | 6.995537 | 7.590987 | 7.059203 | 7.332271 | 7.558037 | 5.388795 | 2.439311 | low |
| GSM437278 | 4.654794521 | 0 | 7.36696 | 6.485229 | 7.608832 | 6.628686 | 8.032132 | 6.845894 | 7.853205 | 7.546679 | 6.231992 | 8.419649 | 5.430821 | 3.03085 | high |
| GSM437279 | 5.405479452 | 0 | 7.986391 | 6.553501 | 7.001309 | 7.144557 | 7.351072 | 6.889095 | 8.155717 | 7.274405 | 7.988204 | 7.66263 | 5.024432 | 2.730753 | low |
| GSM437280 | 4.561643836 | 0 | 7.253798 | 6.448744 | 6.996013 | 6.829744 | 7.61602 | 7.247534 | 7.600931 | 7.721928 | 6.286444 | 7.972443 | 5.383236 | 2.913863 | high |
| GSM437283 | 4.980821918 | 0 | 7.908767 | 7.371703 | 6.69796 | 7.360881 | 7.428766 | 7.555361 | 8.339487 | 6.918892 | 6.432728 | 7.864977 | 5.075845 | 2.799317 | low |
| GSM437285 | 4.542465753 | 0 | 7.249539 | 6.472273 | 6.941843 | 7.01282 | 8.088687 | 7.707655 | 6.835037 | 7.006804 | 6.445544 | 7.90322 | 5.014223 | 2.926823 | high |
| GSM437297 | 0.652054795 | 1 | 7.433452 | 6.36625 | 7.676243 | 7.526424 | 7.444532 | 8.206653 | 7.871797 | 7.366563 | 6.886677 | 7.44424 | 5.447851 | 2.829303 | low |
| GSM437299 | 5.671232877 | 0 | 7.879097 | 6.450557 | 7.79215 | 7.023738 | 7.176007 | 7.205947 | 8.201208 | 6.941477 | 7.622828 | 7.669775 | 5.195275 | 2.707065 | low |
| GSM437300 | 4.178082192 | 0 | 7.113427 | 6.160638 | 7.699717 | 6.923382 | 7.514916 | 7.295363 | 8.282335 | 7.191541 | 5.913049 | 7.758262 | 5.125329 | 2.867887 | low |
| GSM437301 | 5.904109589 | 0 | 6.736638 | 7.952083 | 9.562402 | 6.893452 | 7.736209 | 7.52626 | 8.207312 | 7.37132 | 7.029121 | 8.138422 | 5.074227 | 3.11163 | high |
| GSM437302 | 6.191780822 | 0 | 7.08435 | 6.436498 | 7.54905 | 6.907795 | 7.38089 | 7.642131 | 7.925067 | 6.980136 | 7.304034 | 7.735515 | 5.114346 | 2.848729 | low |
| GSM437303 | 3.871232877 | 0 | 7.070643 | 6.104206 | 8.128973 | 6.897838 | 7.310661 | 7.32887 | 7.975777 | 7.05175 | 8.360479 | 7.577486 | 5.544597 | 2.853687 | low |
| GSM437304 | 6.397260274 | 0 | 9.907582 | 7.254771 | 7.603203 | 7.245061 | 7.29096 | 7.67746 | 9.02671 | 6.691241 | 6.700645 | 7.752734 | 5.43965 | 2.526722 | low |
| GSM437305 | 1.950684932 | 1 | 7.367314 | 6.763899 | 7.517305 | 6.734597 | 7.463099 | 7.907115 | 8.857302 | 7.295379 | 7.303799 | 7.905225 | 5.32994 | 2.924456 | high |
| GSM437306 | 1.802739726 | 1 | 8.86388 | 6.832786 | 7.731202 | 6.903621 | 7.481375 | 7.265175 | 8.82421 | 7.327274 | 6.438905 | 8.282588 | 5.201124 | 2.735629 | low |
| GSM437307 | 2.75890411 | 1 | 6.880437 | 7.021522 | 8.084246 | 6.885036 | 7.330163 | 7.435397 | 8.779043 | 7.262715 | 7.684675 | 8.061085 | 5.533207 | 3.006495 | high |
| GSM437308 | 5.764383562 | 0 | 6.7113 | 6.169908 | 7.754788 | 6.934595 | 7.564424 | 7.52626 | 8.214292 | 7.381541 | 8.845036 | 8.382517 | 5.374334 | 3.054769 | high |
| GSM437309 | 3.915068493 | 0 | 7.945038 | 6.46566 | 7.865062 | 6.570121 | 7.445182 | 7.352306 | 8.235728 | 7.44792 | 7.636916 | 8.014516 | 5.695785 | 2.841326 | low |
| GSM437312 | 3.389041096 | 0 | 7.624903 | 6.53402 | 7.363391 | 6.968861 | 7.235665 | 6.995942 | 9.013125 | 7.337776 | 6.890415 | 7.674604 | 5.25165 | 2.7998 | low |
| GSM437313 | 3.394520548 | 0 | 7.236052 | 6.323697 | 8.0197 | 7.742083 | 7.499006 | 7.148743 | 8.55719 | 7.408843 | 6.947486 | 7.54941 | 5.524286 | 2.906746 | high |
| GSM437314 | 1.153424658 | 1 | 7.014649 | 6.837973 | 8.701304 | 7.059218 | 7.655744 | 7.506777 | 7.361189 | 7.71024 | 6.95504 | 8.095503 | 5.211253 | 2.986578 | high |
| GSM437315 | 5.871232877 | 0 | 7.394214 | 6.296656 | 8.754298 | 8.124166 | 7.681657 | 7.372914 | 8.906176 | 8.017134 | 6.938932 | 9.138667 | 5.537405 | 3.126629 | high |
| GSM437316 | 3 | 1 | 7.378098 | 6.5041 | 7.897596 | 6.820853 | 7.211045 | 7.666294 | 8.732859 | 6.912803 | 8.877679 | 7.385583 | 9.2348 | 3.078264 | high |
| GSM437317 | 0.128767123 | 1 | 7.126035 | 6.423498 | 8.186627 | 7.702478 | 7.779651 | 8.026862 | 9.531505 | 7.309253 | 6.998011 | 8.086493 | 5.420604 | 3.074717 | high |
| GSM437318 | 1.471232877 | 1 | 6.798528 | 6.018545 | 8.305918 | 6.902909 | 7.732648 | 7.443704 | 8.463504 | 7.824322 | 7.523504 | 8.186812 | 5.395398 | 3.06914 | high |
| GSM437319 | 1.054794521 | 1 | 7.06832 | 6.422143 | 8.91405 | 7.09584 | 7.752525 | 7.35417 | 9.074921 | 7.74848 | 8.58791 | 8.352135 | 5.515622 | 3.117738 | high |
| GSM437320 | 0.575342466 | 1 | 6.958455 | 6.86866 | 7.86535 | 7.379232 | 7.62977 | 7.16333 | 9.240811 | 7.866259 | 7.530568 | 7.99209 | 5.097356 | 3.059485 | high |
| GSM437321 | 2.709589041 | 1 | 6.913646 | 6.909636 | 7.451302 | 7.003095 | 7.241376 | 7.299525 | 8.313026 | 7.061847 | 7.59916 | 7.481245 | 5.821949 | 2.910336 | high |
| GSM437322 | 1.345205479 | 1 | 6.549005 | 6.129508 | 8.210198 | 7.303286 | 7.570397 | 7.413367 | 8.169907 | 7.283062 | 7.016771 | 7.709484 | 5.346543 | 2.989383 | high |
| GSM437324 | 3.452054794 | 0 | 7.447815 | 6.469547 | 7.732967 | 7.240467 | 7.6491 | 7.287959 | 8.314324 | 7.0736 | 6.116996 | 7.757343 | 5.308597 | 2.878281 | low |

**Table S12 TIDE scores of 348 patients in TCGA-COAD cohort.**

| id | HRI riskScore | HRI risk | Responder | TIDE score | Dysfunction | Exclusion |
| --- | --- | --- | --- | --- | --- | --- |
| TCGA-G4-6294 | 0.960518409 | low | TRUE | -0.29 | -0.92 | -0.29 |
| TCGA-AZ-4313 | 0.758631429 | low | FALSE | 0.83 | 0.56 | 0.83 |
| TCGA-CA-5256 | 1.042768242 | low | FALSE | 0.87 | -1.61 | 0.87 |
| TCGA-G4-6317 | 1.517006502 | high | FALSE | 0.74 | -1.43 | 0.74 |
| TCGA-AA-3488 | 1.14579034 | low | FALSE | 0.44 | 0.22 | 0.44 |
| TCGA-A6-5661 | 0.707578444 | low | TRUE | -0.7 | 1.33 | -0.7 |
| TCGA-G4-6307 | 1.155582402 | low | FALSE | 0.56 | -0.08 | 0.56 |
| TCGA-A6-5656 | 0.866989285 | low | TRUE | -0.86 | -0.67 | -0.86 |
| TCGA-DM-A28M | 1.065694501 | low | FALSE | 0.09 | -1.11 | 0.09 |
| TCGA-AZ-4614 | 1.567360972 | high | FALSE | 0.54 | -0.32 | 0.54 |
| TCGA-QG-A5YX | 0.399194717 | low | TRUE | -0.86 | -1.06 | -0.86 |
| TCGA-A6-6650 | 0.759490484 | low | TRUE | -0.51 | -1.34 | -0.51 |
| TCGA-NH-A50T | 1.124103189 | low | FALSE | 0.95 | -1.62 | 0.95 |
| TCGA-CM-5862 | 1.399004522 | high | FALSE | 0.4 | -1.72 | 0.4 |
| TCGA-DM-A28G | 1.162787229 | low | TRUE | -0.11 | -0.24 | -0.11 |
| TCGA-5M-AATE | 1.504162702 | high | FALSE | 0.28 | -0.73 | 0.28 |
| TCGA-DM-A28H | 1.346389173 | low | FALSE | 0.7 | -1.83 | 0.7 |
| TCGA-CM-4747 | 1.453051642 | high | FALSE | 0.23 | 1.68 | 0.23 |
| TCGA-AZ-4315 | 1.184507448 | low | FALSE | 0.36 | -0.65 | 0.36 |
| TCGA-A6-2672 | 1.048158821 | low | TRUE | -1.33 | 0.93 | -1.33 |
| TCGA-AA-A01I | 0.994580174 | low | TRUE | -0.41 | -1.34 | -0.41 |
| TCGA-AA-A01Z | 1.461017946 | high | FALSE | 0.01 | 0.83 | 0.01 |
| TCGA-AA-3519 | 0.799140097 | low | TRUE | -0.35 | 0.62 | -0.35 |
| TCGA-A6-5659 | 0.751167181 | low | TRUE | -0.72 | -0.31 | -0.72 |
| TCGA-DM-A0X9 | 1.390017091 | high | FALSE | 0.01 | -0.9 | 0.01 |
| TCGA-F4-6808 | 1.099468174 | low | FALSE | 0.26 | -0.31 | 0.26 |
| TCGA-AA-A017 | 1.259796938 | low | TRUE | -0.39 | 1.13 | -0.39 |
| TCGA-AA-A01X | 1.449670366 | high | FALSE | 0.31 | 1.3 | 0.31 |
| TCGA-D5-6532 | 1.263579757 | low | FALSE | 0.02 | -1.32 | 0.02 |
| TCGA-DM-A0XD | 1.606027371 | high | FALSE | 0.33 | 0.52 | 0.33 |
| TCGA-AA-3844 | 0.851690837 | low | TRUE | -0.17 | -0.7 | -0.17 |
| TCGA-AY-A71X | 1.057913749 | low | TRUE | -0.44 | -0.15 | -0.44 |
| TCGA-DM-A28C | 1.103385369 | low | TRUE | -0.22 | -0.42 | -0.22 |
| TCGA-AA-3560 | 0.412812879 | low | FALSE | 0.36 | 0.3 | 0.36 |
| TCGA-G4-6304 | 0.645378417 | low | FALSE | 0.03 | -0.28 | 0.03 |
| TCGA-CM-5864 | 1.276617948 | low | FALSE | 0.06 | -1.89 | 0.06 |
| TCGA-AA-A01V | 1.41512531 | high | TRUE | -0.54 | 1.54 | -0.54 |
| TCGA-AZ-6599 | 0.812712721 | low | TRUE | -0.37 | 0.07 | -0.37 |
| TCGA-AA-A01Q | 1.136409305 | low | TRUE | -0.88 | 1.19 | -0.88 |
| TCGA-AY-A54L | 1.393370344 | high | TRUE | -0.03 | -1.56 | -0.03 |
| TCGA-AD-6888 | 1.077035335 | low | FALSE | 0.11 | -0.34 | 0.11 |
| TCGA-AA-3514 | 1.544180691 | high | FALSE | 0.44 | 1.64 | 0.44 |
| TCGA-SS-A7HO | 1.587029691 | high | FALSE | 0.69 | -0.64 | 0.69 |
| TCGA-AA-A00L | 0.986183612 | low | TRUE | -0.43 | 0.17 | -0.43 |
| TCGA-AA-A01S | 1.485066593 | high | FALSE | 0.17 | 0.67 | 0.17 |
| TCGA-AA-A02K | 1.28152898 | low | FALSE | 0.13 | 0.14 | 0.13 |
| TCGA-DM-A1D9 | 1.359885417 | high | FALSE | 0.65 | -1.3 | 0.65 |
| TCGA-A6-2683 | 1.696521541 | high | TRUE | -0.26 | 1.43 | -0.26 |
| TCGA-DM-A1DA | 1.774064581 | high | FALSE | 0.47 | -0.89 | 0.47 |
| TCGA-DM-A28E | 0.997761216 | low | TRUE | -0.24 | -1.04 | -0.24 |
| TCGA-4N-A93T | 1.189417728 | low | TRUE | -0.64 | 0.35 | -0.64 |
| TCGA-D5-6538 | 1.356201604 | low | TRUE | -0.06 | -0.86 | -0.06 |
| TCGA-AA-A02Y | 0.720509743 | low | TRUE | -1.02 | 0.3 | -1.02 |
| TCGA-AZ-6608 | 1.465870773 | high | FALSE | 0.37 | -1.19 | 0.37 |
| TCGA-AA-A01T | 0.850715773 | low | TRUE | -0.15 | 1.67 | -0.15 |
| TCGA-AA-A03J | 1.126403596 | low | TRUE | -0.5 | 2.3 | -0.5 |
| TCGA-AA-A004 | 1.231154525 | low | TRUE | -0.25 | 1.77 | -0.25 |
| TCGA-AZ-5407 | 1.185040053 | low | TRUE | -0.16 | -0.9 | -0.16 |
| TCGA-AA-3548 | 1.329648045 | low | TRUE | -0.08 | 1.24 | -0.08 |
| TCGA-DM-A1DB | 1.096771107 | low | TRUE | -0.04 | -1.02 | -0.04 |
| TCGA-AA-A01F | 1.115475207 | low | TRUE | -0.53 | 0.9 | -0.53 |
| TCGA-D5-5540 | 1.617503374 | high | FALSE | 1.19 | -2.44 | 1.19 |
| TCGA-AA-3861 | 0.765905378 | low | TRUE | -0.86 | -0.27 | -0.86 |
| TCGA-AA-3531 | 1.309175537 | low | TRUE | -0.01 | 0.89 | -0.01 |
| TCGA-AA-3561 | 1.093529856 | low | FALSE | 0.31 | -1.51 | 0.31 |
| TCGA-CM-4744 | 1.516039482 | high | FALSE | 0.31 | -0.28 | 0.31 |
| TCGA-AA-3941 | 1.587297594 | high | TRUE | -0.07 | -0.41 | -0.07 |
| TCGA-A6-6652 | 1.384439941 | high | FALSE | 0.02 | -0.33 | 0.02 |
| TCGA-DM-A1HA | 1.869054555 | high | FALSE | 0.19 | 0 | 0.19 |
| TCGA-AA-3542 | 1.212962556 | low | FALSE | 0.71 | 0.51 | 0.71 |
| TCGA-QL-A97D | 0.909799143 | low | TRUE | -1.22 | -0.25 | -1.22 |
| TCGA-DM-A1D4 | 1.045833068 | low | TRUE | -0.61 | -0.59 | -0.61 |
| TCGA-RU-A8FL | 0.986313252 | low | FALSE | 0.16 | -1.1 | 0.16 |
| TCGA-AZ-4308 | 1.057385912 | low | FALSE | 0.74 | 1.53 | 0.74 |
| TCGA-AA-3972 | 0.716635511 | low | FALSE | 0.11 | -0.84 | 0.11 |
| TCGA-AA-3848 | 1.41873792 | high | TRUE | -0.3 | 1.6 | -0.3 |
| TCGA-AA-3667 | 1.345466888 | low | TRUE | -0.05 | 1.61 | -0.05 |
| TCGA-AA-A00W | 1.015458969 | low | TRUE | -0.73 | 0.23 | -0.73 |
| TCGA-CM-4746 | 1.316859454 | low | FALSE | 0.43 | 0.14 | 0.43 |
| TCGA-AA-3502 | 1.054855729 | low | TRUE | -0.2 | 0.14 | -0.2 |
| TCGA-DM-A1D0 | 1.062852816 | low | TRUE | -0.01 | -0.94 | -0.01 |
| TCGA-NH-A8F7 | 1.1790161 | low | TRUE | -0.4 | -0.11 | -0.4 |
| TCGA-A6-3807 | 1.643126361 | high | FALSE | 0.15 | 0.01 | 0.15 |
| TCGA-AA-3846 | 0.586841654 | low | TRUE | -0.7 | -1.14 | -0.7 |
| TCGA-QG-A5Z2 | 0.481028044 | low | TRUE | -1.49 | 0.18 | -1.49 |
| TCGA-CM-5341 | 1.442986818 | high | FALSE | 0.02 | -1.17 | 0.02 |
| TCGA-AA-3662 | 1.247662689 | low | TRUE | -0.79 | 0.45 | -0.79 |
| TCGA-A6-2678 | 0.831964969 | low | TRUE | -0.16 | -1.43 | -0.16 |
| TCGA-AA-3976 | 1.199812762 | low | FALSE | 0.95 | -1.44 | 0.95 |
| TCGA-CA-5797 | 1.527819056 | high | FALSE | 0.5 | -0.82 | 0.5 |
| TCGA-AA-A02H | 1.613809449 | high | FALSE | 0.16 | -0.04 | 0.16 |
| TCGA-AA-3862 | 0.764049846 | low | TRUE | -0.63 | -0.7 | -0.63 |
| TCGA-CA-6716 | 1.718216864 | high | FALSE | 0.63 | -0.97 | 0.63 |
| TCGA-D5-6535 | 1.558057211 | high | TRUE | -0.99 | -0.06 | -0.99 |
| TCGA-A6-6137 | 1.114818801 | low | TRUE | -1.51 | 0.22 | -1.51 |
| TCGA-AA-3986 | 0.897560863 | low | TRUE | -1.62 | 1.48 | -1.62 |
| TCGA-CM-6165 | 1.347927845 | low | TRUE | -0.26 | -0.96 | -0.26 |
| TCGA-CM-4750 | 1.694847548 | high | FALSE | 0.74 | 0.63 | 0.74 |
| TCGA-AA-3520 | 1.359411054 | high | FALSE | 0.73 | -0.38 | 0.73 |
| TCGA-G4-6625 | 0.844550089 | low | TRUE | -1.96 | 1.71 | -1.96 |
| TCGA-AA-A02E | 1.664562024 | high | TRUE | -0.06 | 0.18 | -0.06 |
| TCGA-AA-3710 | 1.546144464 | high | FALSE | 1.06 | 1.06 | -2.15 |
| TCGA-G4-6320 | 1.129221379 | low | FALSE | 0.01 | 0.22 | 0.01 |
| TCGA-AA-A00Z | 0.828047881 | low | TRUE | -0.01 | 0.11 | -0.01 |
| TCGA-CM-6674 | 1.505711751 | high | FALSE | 0.61 | -0.12 | 0.61 |
| TCGA-A6-2680 | 1.23317194 | low | TRUE | -0.23 | 1.49 | -0.23 |
| TCGA-AA-3930 | 1.597977299 | high | TRUE | -0.38 | -0.99 | -0.38 |
| TCGA-AA-3982 | 1.085069292 | low | TRUE | -0.55 | -0.1 | -0.55 |
| TCGA-AA-3510 | 1.030800341 | low | TRUE | -0.58 | 0.89 | -0.58 |
| TCGA-G4-6310 | 1.749725255 | high | FALSE | 1.1 | -0.24 | 1.1 |
| TCGA-AA-A00K | 1.497764955 | high | TRUE | -0.12 | 0.82 | -0.12 |
| TCGA-G4-6588 | 1.477659396 | high | FALSE | 0.31 | -0.31 | 0.31 |
| TCGA-CK-5912 | 1.262593741 | low | FALSE | 0 | -1.38 | 0 |
| TCGA-CM-5344 | 0.968572667 | low | FALSE | 1.39 | 0.04 | 1.39 |
| TCGA-AA-3831 | 1.131780062 | low | TRUE | -0.46 | 0.45 | -0.46 |
| TCGA-D5-6541 | 1.109337074 | low | TRUE | -0.68 | 0.19 | -0.68 |
| TCGA-A6-4107 | 1.251476061 | low | FALSE | 0.3 | 0.37 | 0.3 |
| TCGA-AA-3875 | 1.097215343 | low | TRUE | -0.55 | -0.5 | -0.55 |
| TCGA-G4-6295 | 1.590097814 | high | TRUE | -0.52 | -0.52 | -0.11 |
| TCGA-DM-A28F | 1.631041069 | high | TRUE | -0.09 | -0.86 | -0.09 |
| TCGA-CM-4743 | 1.616133812 | high | TRUE | -0.21 | 0.45 | -0.21 |
| TCGA-AA-A00U | 1.539360219 | high | FALSE | 0.87 | -0.72 | 0.87 |
| TCGA-CM-5861 | 2.035645503 | high | FALSE | 0.6 | -0.84 | 0.6 |
| TCGA-CK-4948 | 2.002206588 | high | FALSE | 1.43 | 0.16 | 1.43 |
| TCGA-AA-3968 | 0.863413755 | low | FALSE | 0.41 | -0.66 | 0.41 |
| TCGA-D5-6530 | 0.941455207 | low | TRUE | -1.59 | 1.2 | -1.59 |
| TCGA-G4-6315 | 0.823757275 | low | FALSE | 0.37 | -1.51 | 0.37 |
| TCGA-AA-3562 | 0.954534524 | low | FALSE | 0.33 | -0.01 | 0.33 |
| TCGA-AA-A010 | 1.270538037 | low | TRUE | -0.25 | -0.26 | -0.25 |
| TCGA-AA-3952 | 1.558320779 | high | FALSE | 0.36 | 0.07 | 0.36 |
| TCGA-AA-3532 | 0.857095815 | low | TRUE | -0.98 | 0.29 | -0.98 |
| TCGA-AA-3989 | 1.542839483 | high | TRUE | -0.59 | 1.73 | -0.59 |
| TCGA-AA-3530 | 1.375605775 | high | TRUE | -0.51 | -1.17 | -0.51 |
| TCGA-NH-A5IV | 1.297001633 | low | FALSE | 0.24 | 0.24 | -0.73 |
| TCGA-CK-4947 | 1.109796022 | low | TRUE | -0.65 | -0.59 | -0.65 |
| TCGA-AA-3492 | 1.679522483 | high | TRUE | -0.1 | 0.07 | -0.1 |
| TCGA-AZ-4682 | 0.641839591 | low | FALSE | 0.44 | -1.53 | 0.44 |
| TCGA-G4-6293 | 1.326166171 | low | TRUE | -1.23 | 1.38 | -1.23 |
| TCGA-AA-3552 | 0.868750806 | low | TRUE | -0.88 | 1.27 | -0.88 |
| TCGA-CM-6166 | 1.912227636 | high | FALSE | 1.49 | -1.86 | 1.49 |
| TCGA-AA-3496 | 1.598174126 | high | TRUE | -0.48 | 0.57 | -0.48 |
| TCGA-AY-A8YK | 0.529197066 | low | TRUE | -0.58 | 0.22 | -0.58 |
| TCGA-CM-6677 | 1.387421841 | high | FALSE | 0.91 | -0.68 | 0.91 |
| TCGA-A6-2677 | 1.141065506 | low | TRUE | -0.75 | -0.97 | -0.75 |
| TCGA-AA-3544 | 0.925610842 | low | TRUE | -0.52 | 1.4 | -0.52 |
| TCGA-A6-A567 | 1.507021871 | high | FALSE | 0.26 | -0.54 | 0.26 |
| TCGA-AA-3867 | 1.554091718 | high | FALSE | 0.98 | 0.28 | 0.98 |
| TCGA-NH-A50V | 1.461654894 | high | TRUE | -0.35 | 0.58 | -0.35 |
| TCGA-AA-A01C | 1.586009284 | high | FALSE | 1.01 | 1.1 | 1.01 |
| TCGA-AA-A00O | 1.588133965 | high | TRUE | -0.27 | 0.85 | -0.27 |
| TCGA-AA-A00D | 1.667989766 | high | TRUE | -1.4 | 1.08 | -1.4 |
| TCGA-AA-3522 | 0.744097377 | low | TRUE | -0.63 | -1.22 | -0.63 |
| TCGA-AA-3979 | 1.325699601 | low | FALSE | 0.23 | -1.6 | 0.23 |
| TCGA-A6-5667 | 1.748805347 | high | FALSE | 0.87 | -1.03 | 0.87 |
| TCGA-AA-3842 | 1.613496268 | high | FALSE | 0.42 | -1.11 | 0.42 |
| TCGA-A6-A5ZU | 1.333331391 | low | TRUE | -0.69 | 1.36 | -0.69 |
| TCGA-AA-3511 | 0.772741281 | low | FALSE | 0.35 | 0.5 | 0.35 |
| TCGA-NH-A8F8 | 1.694518063 | high | TRUE | -0.21 | 0.56 | -0.21 |
| TCGA-AA-3973 | 1.38197467 | high | FALSE | 0.24 | -0.33 | 0.24 |
| TCGA-AA-3680 | 1.190170919 | low | TRUE | -0.91 | -0.02 | -0.91 |
| TCGA-QG-A5YW | 0.171816894 | low | TRUE | -0.54 | 0.41 | -0.54 |
| TCGA-AY-A69D | 1.1232216 | low | TRUE | -0.1 | -0.81 | -0.1 |
| TCGA-AA-3673 | 1.613821056 | high | FALSE | 0.22 | -0.48 | 0.22 |
| TCGA-AA-3855 | 1.061217359 | low | TRUE | -0.45 | 1.06 | -0.45 |
| TCGA-CM-6172 | 0.831599089 | low | TRUE | -0.16 | -0.96 | -0.16 |
| TCGA-AA-3971 | 0.235871817 | low | TRUE | -1.22 | 0.98 | -1.22 |
| TCGA-AA-3666 | 1.164816066 | low | TRUE | -0.16 | -1.22 | -0.16 |
| TCGA-D5-6537 | 1.29091006 | low | FALSE | 0.69 | -1.09 | 0.69 |
| TCGA-G4-6321 | 0.381082175 | low | TRUE | -1.55 | 1.27 | -1.55 |
| TCGA-T9-A92H | 1.070730837 | low | TRUE | -0.07 | -0.85 | -0.07 |
| TCGA-AD-6965 | 1.551865609 | high | FALSE | 0.49 | -1.05 | 0.49 |
| TCGA-A6-6140 | 0.643558393 | low | TRUE | -0.4 | -0.41 | -0.4 |
| TCGA-AA-3553 | 1.434267056 | high | TRUE | -0.82 | -0.82 | 0.4 |
| TCGA-A6-A56B | 1.776498632 | high | FALSE | 0.74 | -0.44 | 0.74 |
| TCGA-AA-A00E | 1.511881477 | high | TRUE | -0.63 | 0.44 | -0.63 |
| TCGA-AA-3956 | 1.074077781 | low | TRUE | -0.61 | -0.05 | -0.61 |
| TCGA-AA-3713 | 0.787625277 | low | TRUE | -1.28 | 0.16 | -1.28 |
| TCGA-AA-3955 | 0.958109105 | low | FALSE | 0.91 | -2.17 | 0.91 |
| TCGA-AZ-6598 | 1.765348189 | high | TRUE | -0.1 | -0.04 | -0.1 |
| TCGA-CM-6163 | 0.463324856 | low | TRUE | -0.55 | -0.7 | -0.55 |
| TCGA-AA-3869 | 1.507483164 | high | TRUE | -0.39 | -0.74 | -0.39 |
| TCGA-AA-3538 | 0.95438468 | low | FALSE | 0.28 | -0.93 | 0.28 |
| TCGA-AA-3494 | 1.061559664 | low | FALSE | 0.36 | -0.41 | 0.36 |
| TCGA-AA-3851 | 1.400292454 | high | TRUE | -0.23 | -0.3 | -0.23 |
| TCGA-A6-5665 | 1.392562074 | high | TRUE | -1 | 0.5 | -1 |
| TCGA-AA-3509 | 0.813000475 | low | TRUE | -0.2 | -1.75 | -0.2 |
| TCGA-AA-3970 | 0.762667692 | low | TRUE | -0.62 | -0.79 | -0.62 |
| TCGA-CK-5914 | 1.405352878 | high | TRUE | -1.77 | -1.77 | 0.25 |
| TCGA-A6-5666 | 1.180217416 | low | FALSE | 0.53 | -2.08 | 0.53 |
| TCGA-CM-6171 | 1.281071495 | low | TRUE | -0.43 | -0.33 | -0.43 |
| TCGA-G4-6309 | 1.024268831 | low | FALSE | 0.44 | -0.42 | 0.44 |
| TCGA-AA-3697 | 0.924131862 | low | TRUE | -0.4 | -0.76 | -0.4 |
| TCGA-G4-6586 | 0.848484048 | low | FALSE | 0.52 | 0.52 | -1.01 |
| TCGA-AZ-4616 | 2.033107106 | high | FALSE | 0.16 | 0.16 | 0.53 |
| TCGA-AA-A02J | 0.927576831 | low | FALSE | 0.59 | -0.89 | 0.59 |
| TCGA-NH-A6GB | 1.344943076 | low | TRUE | -0.25 | 0.09 | -0.25 |
| TCGA-CK-6747 | 1.169142379 | low | FALSE | 0.16 | -0.21 | 0.16 |
| TCGA-AD-A5EK | 1.447496858 | high | FALSE | 0.38 | -0.71 | 0.38 |
| TCGA-AA-3506 | 1.474600032 | high | TRUE | -0.21 | 1.86 | -0.21 |
| TCGA-G4-6298 | 1.972650277 | high | FALSE | 1.55 | -0.3 | 1.55 |
| TCGA-AA-3678 | 0.904219097 | low | TRUE | -0.69 | -0.07 | -0.69 |
| TCGA-F4-6806 | 1.164283752 | low | TRUE | -0.29 | 0.14 | -0.29 |
| TCGA-AA-3812 | 1.049959171 | low | TRUE | -0.02 | 2.04 | -0.02 |
| TCGA-AA-3815 | 1.200761402 | low | FALSE | 1.06 | 1.06 | -1.52 |
| TCGA-CA-6715 | 1.723115201 | high | FALSE | 0.66 | -1.56 | 0.66 |
| TCGA-NH-A6GA | 1.756728479 | high | TRUE | -0.07 | -0.32 | -0.07 |
| TCGA-AD-6889 | 1.404400411 | high | FALSE | 0.31 | -0.23 | 0.31 |
| TCGA-AA-A029 | 1.7535557 | high | FALSE | 0.11 | 0.67 | 0.11 |
| TCGA-AA-3655 | 1.204648922 | low | FALSE | 0.18 | 0.02 | 0.18 |
| TCGA-CM-6678 | 1.28202568 | low | FALSE | 0.73 | -0.2 | 0.73 |
| TCGA-AA-3660 | 0.645524962 | low | FALSE | 0.09 | -0.38 | 0.09 |
| TCGA-DM-A0XF | 1.53672616 | high | FALSE | 0.76 | -1.21 | 0.76 |
| TCGA-QG-A5Z1 | 1.344167613 | low | TRUE | -0.11 | -0.19 | -0.11 |
| TCGA-AA-3526 | 1.545943415 | high | FALSE | 0.37 | -1.28 | 0.37 |
| TCGA-CA-5255 | 1.121071818 | low | TRUE | -0.32 | -0.01 | -0.32 |
| TCGA-5M-AAT4 | 1.80053832 | high | FALSE | 0.79 | -0.98 | 0.79 |
| TCGA-AA-3864 | 1.187302954 | low | FALSE | 0.6 | -1.72 | 0.6 |
| TCGA-CM-6161 | 1.531954676 | high | TRUE | -0.52 | -0.52 | 0.05 |
| TCGA-AA-3819 | 0.556263219 | low | TRUE | -0.08 | -0.17 | -0.08 |
| TCGA-AA-3685 | 0.944827198 | low | FALSE | 0.08 | -0.41 | 0.08 |
| TCGA-AA-3664 | 1.253296478 | low | TRUE | -0.33 | -1.1 | -0.33 |
| TCGA-A6-6648 | 0.30501932 | low | TRUE | -0.15 | -1.48 | -0.15 |
| TCGA-AZ-4684 | 1.23753889 | low | FALSE | 0.36 | 0.66 | 0.36 |
| TCGA-AA-A00A | 1.342696588 | low | TRUE | -0.39 | 0.54 | -0.39 |
| TCGA-CM-5349 | 1.91796872 | high | FALSE | 0.36 | -0.73 | 0.36 |
| TCGA-AA-3534 | 1.159991991 | low | FALSE | 0.32 | -0.45 | 0.32 |
| TCGA-AA-3663 | 1.563334384 | high | FALSE | 0.21 | -1.21 | 0.21 |
| TCGA-AA-3549 | 1.041411892 | low | TRUE | -0.02 | 0.67 | -0.02 |
| TCGA-G4-6626 | 1.456250568 | high | FALSE | 0.62 | -0.71 | 0.62 |
| TCGA-AY-6386 | 1.101891273 | low | TRUE | -0.52 | 0.12 | -0.52 |
| TCGA-AU-3779 | 1.49151178 | high | TRUE | -0.33 | 0.3 | -0.33 |
| TCGA-AA-3517 | 1.056678682 | low | TRUE | -0.42 | 0.75 | -0.42 |
| TCGA-AA-3518 | 1.514054999 | high | TRUE | -0.42 | 0.49 | -0.42 |
| TCGA-CM-4752 | 0.797692848 | low | TRUE | -0.17 | 0.08 | -0.17 |
| TCGA-A6-5660 | 1.482558928 | high | FALSE | 0.55 | -1.88 | 0.55 |
| TCGA-CM-5868 | 1.542622672 | high | FALSE | 1.46 | -1.6 | 1.46 |
| TCGA-CM-6164 | 1.636280736 | high | FALSE | 0.27 | -0.28 | 0.27 |
| TCGA-AZ-6606 | 1.239845356 | low | TRUE | -0.11 | -0.73 | -0.11 |
| TCGA-QG-A5YV | 1.28356054 | low | FALSE | 0.35 | -1.38 | 0.35 |
| TCGA-AA-3688 | 1.197491666 | low | FALSE | 0.02 | -1.15 | 0.02 |
| TCGA-AA-3980 | 1.265356388 | low | TRUE | -0.46 | -0.98 | -0.46 |
| TCGA-AA-3525 | 1.278826367 | low | FALSE | 0.36 | 0.22 | 0.36 |
| TCGA-AA-3495 | 1.030519925 | low | TRUE | -0.23 | -0.84 | -0.23 |
| TCGA-D5-6531 | 1.788813674 | high | TRUE | -0.32 | 1 | -0.32 |
| TCGA-CA-5254 | 1.096391562 | low | FALSE | 0.47 | -0.4 | 0.47 |
| TCGA-AA-3696 | 1.374788429 | high | FALSE | 0.23 | -0.12 | 0.23 |
| TCGA-A6-5662 | 1.815096396 | high | FALSE | 0.46 | -0.82 | 0.46 |
| TCGA-AA-3975 | 0.69818677 | low | TRUE | -0.67 | 1.44 | -0.67 |
| TCGA-F4-6460 | 1.556497858 | high | FALSE | 0.36 | 0.89 | 0.36 |
| TCGA-AA-3858 | 1.181640039 | low | FALSE | 0.87 | 0.29 | 0.87 |
| TCGA-AA-3679 | 1.354989725 | low | FALSE | 0.3 | -0.05 | 0.3 |
| TCGA-AY-5543 | 0.820310222 | low | TRUE | -0.57 | -0.91 | -0.57 |
| TCGA-AA-A00F | 0.610905733 | low | FALSE | 0.27 | 1.29 | 0.27 |
| TCGA-AA-A01K | 1.918871914 | high | FALSE | 0.47 | 0.48 | 0.47 |
| TCGA-D5-6932 | 1.790533841 | high | FALSE | 0.25 | 0.52 | 0.25 |
| TCGA-AA-3675 | 1.34296173 | low | FALSE | 0.54 | -1.44 | 0.54 |
| TCGA-AA-3681 | 1.178136567 | low | TRUE | -0.94 | 0.4 | -0.94 |
| TCGA-AA-A00Q | 1.288914372 | low | TRUE | -0.37 | 0.81 | -0.37 |
| TCGA-A6-6141 | 1.252220043 | low | TRUE | -0.96 | 1.45 | -0.96 |
| TCGA-AA-3939 | 1.446744274 | high | FALSE | 0.15 | -1.11 | 0.15 |
| TCGA-CM-6170 | 0.92393199 | low | TRUE | -0.13 | -0.38 | -0.13 |
| TCGA-AA-A02W | 1.431237449 | high | FALSE | 0.61 | -1.63 | 0.61 |
| TCGA-AA-3524 | 1.140771577 | low | FALSE | 0.69 | -1.72 | 0.69 |
| TCGA-AA-3841 | 1.077755314 | low | TRUE | -0.1 | 2.01 | -0.1 |
| TCGA-F4-6703 | 1.273174786 | low | FALSE | 1.26 | 1.26 | -0.24 |
| TCGA-F4-6461 | 1.478986388 | high | FALSE | 0.02 | 0.69 | 0.02 |
| TCGA-G4-6303 | 1.769851549 | high | FALSE | 0.08 | -0.72 | 0.08 |
| TCGA-CM-6675 | 1.765592776 | high | FALSE | 0.16 | -0.63 | 0.16 |
| TCGA-A6-5664 | 1.849180173 | high | FALSE | 0.57 | 0.89 | 0.57 |
| TCGA-A6-4105 | 1.782182511 | high | TRUE | -0.08 | -0.08 | 0.29 |
| TCGA-AA-3860 | 1.133139533 | low | TRUE | -0.04 | 0.45 | -0.04 |
| TCGA-D5-6920 | 0.450358922 | low | TRUE | -1.16 | 0.78 | -1.16 |
| TCGA-A6-6138 | 1.749083378 | high | TRUE | -1.54 | 0.67 | -1.54 |
| TCGA-A6-2685 | 1.544736565 | high | FALSE | 0.23 | 2.01 | 0.23 |
| TCGA-CK-5916 | 1.719879807 | high | TRUE | -0.22 | -0.57 | -0.22 |
| TCGA-AA-3833 | 1.502991336 | high | TRUE | -0.05 | 1.56 | -0.05 |
| TCGA-G4-6314 | 2.086068728 | high | FALSE | 2.22 | -0.07 | 2.22 |
| TCGA-CA-6718 | 2.390901127 | high | TRUE | -0.9 | 0.2 | -0.9 |
| TCGA-CM-6680 | 1.368923816 | high | TRUE | -0.45 | 0.34 | -0.45 |
| TCGA-A6-6142 | 2.335646017 | high | FALSE | 1.3 | 0.82 | 1.3 |
| TCGA-AD-6895 | 2.422957993 | high | FALSE | 0.95 | 0.95 | -0.25 |
| TCGA-F4-6807 | 2.227365537 | high | FALSE | 0.73 | 0.66 | 0.73 |
| TCGA-D5-6529 | 1.674536356 | high | TRUE | -0.49 | 1.38 | -0.49 |
| TCGA-CM-6679 | 1.350866994 | low | FALSE | 0.89 | 0.68 | 0.89 |
| TCGA-AZ-6607 | 2.300134291 | high | FALSE | 1.68 | 0.78 | 1.68 |
| TCGA-A6-3810 | 1.68503755 | high | FALSE | 0.07 | -0.25 | 0.07 |
| TCGA-A6-2675 | 0.918653024 | low | TRUE | -0.65 | 1.05 | -0.65 |
| TCGA-AA-3554 | 1.855598115 | high | TRUE | -1.41 | 1.56 | -1.41 |
| TCGA-D5-5538 | 1.579772764 | high | TRUE | -1.09 | -0.32 | -1.09 |
| TCGA-AA-A01P | 1.561951703 | high | FALSE | 1.91 | 1.91 | -1.29 |
| TCGA-AZ-5403 | 1.097732358 | low | FALSE | 1.39 | -1.78 | 1.39 |
| TCGA-G4-6299 | 1.5581624 | high | FALSE | 0.32 | 0.26 | 0.32 |
| TCGA-CK-5913 | 1.616328921 | high | FALSE | 0.07 | -0.42 | 0.07 |
| TCGA-AZ-6605 | 1.500884826 | high | FALSE | 1.18 | 1.06 | 1.18 |
| TCGA-CM-4751 | 1.192442688 | low | TRUE | -0.64 | 1.03 | -0.64 |
| TCGA-G4-6627 | 1.420544058 | high | TRUE | -0.67 | 1.05 | -0.67 |
| TCGA-CM-6676 | 1.89626145 | high | FALSE | 1.3 | -0.89 | 1.3 |
| TCGA-CA-6719 | 1.9225872 | high | FALSE | 0.43 | -0.09 | 0.43 |
| TCGA-AZ-4615 | 1.562899809 | high | TRUE | -0.23 | -0.23 | -0.74 |
| TCGA-A6-5657 | 2.163917136 | high | FALSE | 1.23 | 0.74 | 1.23 |
| TCGA-D5-6923 | 1.837235859 | high | FALSE | 2.18 | -0.44 | 2.18 |
| TCGA-D5-6922 | 1.123379339 | low | FALSE | 0.54 | -0.39 | 0.54 |
| TCGA-AA-3870 | 2.181268237 | high | FALSE | 0.4 | 1.69 | 0.4 |
| TCGA-CM-6167 | 2.084309953 | high | FALSE | 1.34 | 0.3 | 1.34 |
| TCGA-CM-5348 | 1.96584229 | high | TRUE | -0.29 | 0.9 | -0.29 |
| TCGA-A6-2671 | 2.053135474 | high | FALSE | 0.72 | 1.1 | 0.72 |
| TCGA-G4-6628 | 1.377165535 | high | FALSE | 0.46 | 0.46 | -0.82 |
| TCGA-AZ-6600 | 1.745491941 | high | FALSE | 0.06 | 0.31 | 0.06 |
| TCGA-D5-6898 | 1.86814759 | high | FALSE | 0.37 | -0.81 | 0.37 |
| TCGA-AA-3489 | 2.103154906 | high | FALSE | 1.37 | 1.37 | -0.28 |
| TCGA-D5-6931 | 1.504020661 | high | TRUE | -0.15 | 0.23 | -0.15 |
| TCGA-D5-6927 | 1.834335953 | high | FALSE | 0.19 | 0.19 | -0.35 |
| TCGA-A6-2676 | 1.842085552 | high | TRUE | -0.53 | 0.28 | -0.53 |
| TCGA-F4-6570 | 1.635142442 | high | TRUE | -1.17 | 1.42 | -1.17 |
| TCGA-CM-6168 | 1.67669436 | high | FALSE | 0.77 | -0.03 | 0.77 |
| TCGA-A6-6654 | 1.909652365 | high | TRUE | -0.26 | 1.51 | -0.26 |
| TCGA-A6-2682 | 1.783681868 | high | FALSE | 0.59 | 0.58 | 0.59 |
| TCGA-A6-6782 | 1.691381884 | high | TRUE | -0.22 | 1 | -0.22 |
| TCGA-CM-5860 | 1.682745649 | high | FALSE | 1.47 | -1.47 | 1.47 |
| TCGA-A6-6653 | 1.424720915 | high | FALSE | 0.37 | 0.03 | 0.37 |
| TCGA-A6-3808 | 2.011273298 | high | FALSE | 0.77 | 0.08 | 0.77 |
| TCGA-A6-6651 | 2.049076152 | high | FALSE | 1.24 | 1.24 | 0.54 |
| TCGA-F4-6809 | 1.86282964 | high | FALSE | 0.18 | 1.07 | 0.18 |
| TCGA-CM-6169 | 1.636304705 | high | FALSE | 0.28 | 0.28 | -0.83 |
| TCGA-F4-6855 | 1.79453824 | high | FALSE | 1.91 | -0.3 | 1.91 |
| TCGA-D5-6536 | 2.005220523 | high | FALSE | 0.19 | 0.23 | 0.19 |
| TCGA-A6-6649 | 1.10502636 | low | TRUE | -0.19 | 0.6 | -0.19 |
| TCGA-G4-6311 | 1.64981715 | high | FALSE | 0.05 | -0.19 | 0.05 |
| TCGA-G4-6297 | 1.588489903 | high | FALSE | 0.72 | 0.17 | 0.72 |
| TCGA-5M-AAT6 | 1.992301187 | high | FALSE | 0.23 | 0.33 | 0.23 |
| TCGA-D5-6926 | 1.842037401 | high | FALSE | 0.43 | -0.16 | 0.43 |
| TCGA-D5-6929 | 1.796004505 | high | FALSE | 0.63 | -0.17 | 0.63 |
| TCGA-F4-6569 | 2.030714117 | high | FALSE | 2.06 | 0.93 | 2.06 |
| TCGA-A6-2684 | 1.494251257 | high | FALSE | 0.07 | 1.99 | 0.07 |
| TCGA-A6-2681 | 1.678321021 | high | FALSE | 0.26 | 1.5 | 0.26 |
| TCGA-D5-5541 | 1.619853545 | high | TRUE | -0.11 | -1.53 | -0.11 |
| TCGA-A6-2686 | 1.995395551 | high | FALSE | 0.56 | 0.56 | -1.07 |
| TCGA-D5-6924 | 1.653098566 | high | FALSE | 0.16 | 0.11 | 0.16 |
| TCGA-AY-4070 | 2.579868438 | high | FALSE | 0.55 | 0.68 | 0.55 |
| TCGA-AA-A02R | 1.506607199 | high | TRUE | -0.85 | 1.24 | -0.85 |
| TCGA-F4-6805 | 1.677818968 | high | FALSE | 1.14 | -0.03 | 1.14 |
| TCGA-AZ-6601 | 1.928217411 | high | FALSE | 0.68 | 0.68 | -1.46 |
| TCGA-AU-6004 | 1.614598739 | high | TRUE | -1.05 | 0.91 | -1.05 |
| TCGA-DM-A28A | 1.997465787 | high | FALSE | 0.87 | -0.96 | 0.87 |
| TCGA-AA-3866 | 1.594510879 | high | TRUE | -1.09 | -0.03 | -1.09 |
| TCGA-AZ-4323 | 2.068250877 | high | TRUE | -0.51 | 1.44 | -0.51 |
| TCGA-AD-6548 | 1.15195874 | low | TRUE | -0.11 | -0.11 | -0.1 |
| TCGA-F4-6459 | 2.010225716 | high | FALSE | 1.03 | -0.13 | 1.03 |

**Table S13 TIDE scores of 210 patients in GSE17538.**

| id | HRI riskScore | HRI risk | Responder | TIDE score | Dysfunction | Exclusion |
| --- | --- | --- | --- | --- | --- | --- |
| GSM437093 | 2.707230315 | low | TRUE | -0.97 | 0.89 | -0.97 |
| GSM437094 | 2.774663152 | low | TRUE | -0.92 | -0.18 | -0.92 |
| GSM437095 | 2.726193899 | low | TRUE | -0.7 | -0.42 | -0.7 |
| GSM437096 | 2.666111099 | low | FALSE | 0.21 | -0.37 | 0.21 |
| GSM437097 | 2.882299719 | low | TRUE | -0.17 | -1.05 | -0.17 |
| GSM437098 | 2.897650684 | high | FALSE | 0 | 0.22 | 0 |
| GSM437099 | 2.836306359 | low | TRUE | -1.51 | -0.17 | -1.51 |
| GSM437100 | 2.852316674 | low | TRUE | -0.07 | 0.51 | -0.07 |
| GSM437101 | 3.014230694 | high | TRUE | -0.36 | -1.08 | -0.36 |
| GSM437102 | 2.934660576 | high | FALSE | 0.47 | -0.02 | 0.47 |
| GSM437103 | 2.628141756 | low | TRUE | -0.33 | 0.51 | -0.33 |
| GSM437104 | 2.730723536 | low | TRUE | -1.31 | 0.61 | -1.31 |
| GSM437105 | 2.765203 | low | TRUE | -1.29 | 0.53 | -1.29 |
| GSM437106 | 2.668349216 | low | FALSE | 0.01 | -0.24 | 0.01 |
| GSM437107 | 2.751492126 | low | TRUE | -0.54 | -0.05 | -0.54 |
| GSM437108 | 2.778321611 | low | TRUE | -0.54 | -0.2 | -0.54 |
| GSM437109 | 3.085353702 | high | FALSE | 0.77 | 0.77 | -0.98 |
| GSM437110 | 2.902859093 | high | FALSE | 0.4 | 0.4 | -0.88 |
| GSM437111 | 3.044785437 | high | FALSE | 0.01 | 1.11 | 0.01 |
| GSM437112 | 2.771557513 | low | TRUE | -0.62 | -0.77 | -0.62 |
| GSM437113 | 2.798057729 | low | TRUE | -0.54 | 0.72 | -0.54 |
| GSM437114 | 2.846279213 | low | TRUE | -0.2 | -0.29 | -0.2 |
| GSM437115 | 3.054206544 | high | TRUE | -0.09 | -0.01 | -0.09 |
| GSM437116 | 2.902558437 | high | TRUE | -0.39 | 0.3 | -0.39 |
| GSM437117 | 2.934703897 | high | FALSE | 1.25 | -0.51 | 1.25 |
| GSM437118 | 3.109978693 | high | FALSE | 0.42 | 0.42 | -0.95 |
| GSM437119 | 3.199517277 | high | FALSE | 2.08 | 0.14 | 2.08 |
| GSM437120 | 2.793992555 | low | FALSE | 1.03 | 0.02 | 1.03 |
| GSM437121 | 2.842295041 | low | TRUE | -0.2 | -0.2 | 0.04 |
| GSM437122 | 2.838019331 | low | FALSE | 1.43 | -1.28 | 1.43 |
| GSM437123 | 2.522018433 | low | TRUE | -0.11 | -0.11 | -0.11 |
| GSM437124 | 2.955226823 | high | FALSE | 0.73 | -0.46 | 0.73 |
| GSM437125 | 3.066462493 | high | FALSE | 0.19 | 0.19 | 0.19 |
| GSM437126 | 2.876560736 | low | TRUE | -0.27 | -0.27 | -1 |
| GSM437127 | 2.814585253 | low | FALSE | 0.17 | 0.6 | 0.17 |
| GSM437128 | 2.978116694 | high | FALSE | 0.49 | -1.02 | 0.49 |
| GSM437129 | 2.850740801 | low | TRUE | -0.38 | -0.57 | -0.38 |
| GSM437130 | 3.005904533 | high | FALSE | 1.65 | 0.14 | 1.65 |
| GSM437131 | 2.760215879 | low | TRUE | -0.67 | 0.89 | -0.67 |
| GSM437132 | 2.906271647 | high | FALSE | 1.4 | -1.05 | 1.4 |
| GSM437133 | 2.990049386 | high | FALSE | 0.87 | 0.87 | -0.21 |
| GSM437134 | 3.017287814 | high | FALSE | 0.31 | 1.31 | 0.31 |
| GSM437135 | 3.089290813 | high | FALSE | 1.3 | 0.72 | 1.3 |
| GSM437136 | 2.965312424 | high | FALSE | 0.23 | -0.06 | 0.23 |
| GSM437137 | 2.862567262 | low | FALSE | 0.75 | -0.78 | 0.75 |
| GSM437138 | 2.83031095 | low | FALSE | 0.6 | -0.22 | 0.6 |
| GSM437139 | 2.847410606 | low | FALSE | 0.31 | -0.83 | 0.31 |
| GSM437140 | 2.772953866 | low | TRUE | -0.59 | -0.66 | -0.59 |
| GSM437141 | 2.921958747 | high | TRUE | -0.49 | -0.49 | -0.61 |
| GSM437142 | 2.852362684 | low | TRUE | -0.57 | -0.6 | -0.57 |
| GSM437143 | 2.590258436 | low | TRUE | -1.47 | -0.04 | -1.47 |
| GSM437144 | 2.748486028 | low | FALSE | 1.1 | 0.08 | 1.1 |
| GSM437145 | 3.022803385 | high | FALSE | 1.23 | 1.23 | -0.85 |
| GSM437146 | 2.657917478 | low | TRUE | -1.34 | 0.04 | -1.34 |
| GSM437147 | 3.040633036 | high | TRUE | -0.49 | -0.49 | -0.44 |
| GSM437148 | 2.344328883 | low | TRUE | -0.94 | -0.37 | -0.94 |
| GSM437149 | 2.898854439 | high | FALSE | 0.12 | 0.23 | 0.12 |
| GSM437150 | 2.92837356 | high | TRUE | -0.9 | 0.92 | -0.9 |
| GSM437151 | 2.68265579 | low | TRUE | -0.76 | 0.35 | -0.76 |
| GSM437152 | 2.91935868 | high | FALSE | 1.64 | 1.64 | -0.96 |
| GSM437153 | 2.753259337 | low | TRUE | -1.04 | 1.01 | -1.04 |
| GSM437154 | 2.813966797 | low | TRUE | -0.93 | 0.82 | -0.93 |
| GSM437155 | 2.957514926 | high | FALSE | 1.01 | 1.01 | -0.67 |
| GSM437156 | 2.922476324 | high | FALSE | 0.77 | 0.77 | -1.39 |
| GSM437157 | 2.827988148 | low | FALSE | 0.46 | 0.2 | 0.46 |
| GSM437158 | 2.865457555 | low | FALSE | 0.39 | -0.77 | 0.39 |
| GSM437159 | 2.844714898 | low | FALSE | 0.1 | 0.41 | 0.1 |
| GSM437160 | 2.849068696 | low | TRUE | -0.95 | -0.51 | -0.95 |
| GSM437161 | 2.870215389 | low | TRUE | -0.37 | 0.25 | -0.37 |
| GSM437162 | 2.866825192 | low | TRUE | -0.45 | -0.07 | -0.45 |
| GSM437163 | 2.974087314 | high | TRUE | -0.27 | 1.55 | -0.27 |
| GSM437164 | 2.829559643 | low | FALSE | 0.13 | -0.01 | 0.13 |
| GSM437165 | 2.590079744 | low | TRUE | -1.23 | -0.45 | -1.23 |
| GSM437166 | 2.772206463 | low | FALSE | 0.63 | 0.27 | 0.63 |
| GSM437167 | 2.848628022 | low | TRUE | -0.73 | -0.05 | -0.73 |
| GSM437168 | 2.964065417 | high | TRUE | -1.32 | -0.63 | -1.32 |
| GSM437169 | 2.946588823 | high | TRUE | -0.86 | -0.14 | -0.86 |
| GSM437170 | 2.89239034 | low | FALSE | 0.67 | -0.16 | 0.67 |
| GSM437171 | 2.770503054 | low | FALSE | 0.28 | -0.07 | 0.28 |
| GSM437172 | 2.890668211 | low | FALSE | 0.06 | -0.82 | 0.06 |
| GSM437173 | 2.998726656 | high | FALSE | 1.2 | -0.28 | 1.2 |
| GSM437174 | 3.034287422 | high | FALSE | 1.3 | -0.52 | 1.3 |
| GSM437175 | 3.145602484 | high | FALSE | 1.21 | 0.5 | 1.21 |
| GSM437176 | 3.083701131 | high | TRUE | -0.13 | -0.36 | -0.13 |
| GSM437177 | 3.00995723 | high | FALSE | 1.24 | -0.91 | 1.24 |
| GSM437178 | 2.889095209 | low | TRUE | -0.21 | 0.46 | -0.21 |
| GSM437179 | 2.865470739 | low | FALSE | 0.24 | -0.78 | 0.24 |
| GSM437180 | 2.88186617 | low | FALSE | 0.56 | 0.22 | 0.56 |
| GSM437181 | 2.913110704 | high | FALSE | 0.84 | 0.95 | 0.84 |
| GSM437182 | 2.998382789 | high | FALSE | 0.66 | -0.46 | 0.66 |
| GSM437183 | 2.825078632 | low | FALSE | 0.31 | -0.86 | 0.31 |
| GSM437184 | 3.074969951 | high | TRUE | -0.15 | -0.31 | -0.15 |
| GSM437185 | 2.890256972 | low | TRUE | -0.63 | 0.81 | -0.63 |
| GSM437186 | 2.89831118 | high | FALSE | 1.17 | -1.65 | 1.17 |
| GSM437187 | 2.69475091 | low | TRUE | -0.03 | 0.63 | -0.03 |
| GSM437188 | 2.838361445 | low | FALSE | 0.33 | -0.88 | 0.33 |
| GSM437189 | 2.933488184 | high | FALSE | 0.17 | 0.14 | 0.17 |
| GSM437190 | 3.027116084 | high | FALSE | 1.67 | -0.41 | 1.67 |
| GSM437191 | 2.803742361 | low | FALSE | 0.14 | 0.08 | 0.14 |
| GSM437192 | 2.843115396 | low | FALSE | 1.6 | -0.53 | 1.6 |
| GSM437193 | 2.844603178 | low | TRUE | -0.14 | -1.25 | -0.14 |
| GSM437194 | 2.596281281 | low | FALSE | 0.2 | -0.51 | 0.2 |
| GSM437195 | 3.027302302 | high | FALSE | 0.5 | -0.75 | 0.5 |
| GSM437196 | 2.927972423 | high | FALSE | 0.28 | -0.81 | 0.28 |
| GSM437197 | 2.760338194 | low | FALSE | 0.9 | 0.35 | 0.9 |
| GSM437198 | 2.90298071 | high | FALSE | 1.28 | -0.61 | 1.28 |
| GSM437199 | 2.956124951 | high | FALSE | 0.55 | 0.81 | 0.55 |
| GSM437200 | 3.084357328 | high | FALSE | 2.13 | -0.22 | 2.13 |
| GSM437201 | 3.014535446 | high | FALSE | 1.43 | 0.74 | 1.43 |
| GSM437202 | 2.996420351 | high | FALSE | 1.01 | -0.66 | 1.01 |
| GSM437203 | 2.970119708 | high | TRUE | -1.14 | 0.42 | -1.14 |
| GSM437204 | 3.23098095 | high | FALSE | 1.48 | 0.31 | 1.48 |
| GSM437205 | 2.855963623 | low | FALSE | 0.13 | 1.51 | 0.13 |
| GSM437206 | 2.923050379 | high | FALSE | 1.55 | -1.5 | 1.55 |
| GSM437207 | 2.885379832 | low | FALSE | 0.93 | -0.88 | 0.93 |
| GSM437208 | 2.780767128 | low | FALSE | 0.04 | -0.1 | 0.04 |
| GSM437209 | 3.109551756 | high | FALSE | 1.79 | 1.79 | -1.69 |
| GSM437210 | 2.921147528 | high | FALSE | 0.47 | 0.25 | 0.47 |
| GSM437211 | 2.997393182 | high | TRUE | -0.01 | -0.36 | -0.01 |
| GSM437212 | 3.046521164 | high | FALSE | 0.01 | 0.01 | -0.83 |
| GSM437213 | 2.975663198 | high | TRUE | -0.5 | -0.22 | -0.5 |
| GSM437214 | 2.723753482 | low | FALSE | 0.47 | 0.33 | 0.47 |
| GSM437215 | 3.084446934 | high | TRUE | -0.58 | 1.05 | -0.58 |
| GSM437216 | 2.30912949 | low | TRUE | -0.07 | -1.18 | -0.07 |
| GSM437217 | 2.747578634 | low | FALSE | 0.09 | -0.21 | 0.09 |
| GSM437218 | 3.023637787 | high | FALSE | 0.46 | -1 | 0.46 |
| GSM437219 | 2.817291286 | low | TRUE | -1.1 | -0.15 | -1.1 |
| GSM437220 | 2.658404904 | low | TRUE | -1.32 | -0.1 | -1.32 |
| GSM437221 | 2.926562856 | high | FALSE | 0.11 | -0.45 | 0.11 |
| GSM437222 | 2.269954109 | low | TRUE | -0.01 | 0.22 | -0.01 |
| GSM437223 | 3.034854747 | high | FALSE | 0.56 | 0.06 | 0.56 |
| GSM437225 | 3.134589407 | high | TRUE | -0.02 | 0.29 | -0.02 |
| GSM437226 | 2.844250732 | low | TRUE | -0.36 | 1.38 | -0.36 |
| GSM437227 | 2.940219671 | high | TRUE | -0.97 | -0.36 | -0.97 |
| GSM437228 | 2.861680407 | low | TRUE | -0.44 | -0.96 | -0.44 |
| GSM437229 | 3.020184102 | high | TRUE | -1 | 0.66 | -1 |
| GSM437230 | 2.613153405 | low | TRUE | -0.31 | 0.04 | -0.31 |
| GSM437231 | 2.865164389 | low | FALSE | 0.63 | -0.88 | 0.63 |
| GSM437232 | 2.906969858 | high | TRUE | -0.57 | 0.82 | -0.57 |
| GSM437233 | 3.059273742 | high | FALSE | 0.71 | -0.48 | 0.71 |
| GSM437234 | 2.894080025 | high | FALSE | 1.41 | -0.47 | 1.41 |
| GSM437235 | 3.124670498 | high | FALSE | 2.37 | -0.38 | 2.37 |
| GSM437236 | 2.975024756 | high | FALSE | 0.36 | -1.07 | 0.36 |
| GSM437237 | 2.914900127 | high | TRUE | -0.16 | -0.84 | -0.16 |
| GSM437238 | 2.848195858 | low | FALSE | 0.47 | -0.53 | 0.47 |
| GSM437239 | 3.06048337 | high | FALSE | 0.86 | -0.67 | 0.86 |
| GSM437240 | 2.849996381 | low | FALSE | 0.9 | -0.4 | 0.9 |
| GSM437241 | 3.071987912 | high | FALSE | 1.35 | -0.28 | 1.35 |
| GSM437243 | 3.008442926 | high | FALSE | 0.8 | -0.49 | 0.8 |
| GSM437244 | 2.856566222 | low | FALSE | 1.33 | -1.27 | 1.33 |
| GSM437245 | 2.911062824 | high | FALSE | 0.24 | -0.47 | 0.24 |
| GSM437246 | 3.162002273 | high | FALSE | 0.18 | -0.64 | 0.18 |
| GSM437247 | 2.883222294 | low | FALSE | 1.16 | -0.97 | 1.16 |
| GSM437248 | 3.066768012 | high | FALSE | 0.27 | -0.75 | 0.27 |
| GSM437249 | 3.185327767 | high | FALSE | 0.77 | -1.2 | 0.77 |
| GSM437250 | 3.083737645 | high | FALSE | 0.53 | 0.29 | 0.53 |
| GSM437251 | 2.643296758 | low | FALSE | 0.6 | 0.55 | 0.6 |
| GSM437252 | 2.874279664 | low | TRUE | -1.23 | 0.94 | -1.23 |
| GSM437253 | 2.929163161 | high | FALSE | 0.24 | -0.73 | 0.24 |
| GSM437254 | 3.130410627 | high | FALSE | 0.21 | 0.21 | -0.06 |
| GSM437255 | 2.747133583 | low | TRUE | -1.77 | 0.21 | -1.77 |
| GSM437256 | 2.946624601 | high | TRUE | -0.9 | 0.03 | -0.9 |
| GSM437257 | 3.007683745 | high | FALSE | 0.79 | -0.51 | 0.79 |
| GSM437258 | 2.783007738 | low | TRUE | -1.01 | 0.02 | -1.01 |
| GSM437259 | 2.739660922 | low | TRUE | -0.27 | -0.24 | -0.27 |
| GSM437260 | 2.926104779 | high | TRUE | -0.53 | -0.2 | -0.53 |
| GSM437261 | 2.716115589 | low | FALSE | 0.35 | 0.35 | -1.38 |
| GSM437262 | 2.939914404 | high | FALSE | 0.96 | 0.12 | 0.96 |
| GSM437263 | 3.066645072 | high | FALSE | 0.85 | -0.8 | 0.85 |
| GSM437264 | 2.829688099 | low | TRUE | -0.07 | -1.09 | -0.07 |
| GSM437265 | 3.058796575 | high | FALSE | 1.02 | 0.12 | 1.02 |
| GSM437266 | 2.91370303 | high | TRUE | -0.82 | 0.84 | -0.82 |
| GSM437267 | 2.949365095 | high | FALSE | 0.24 | -0.1 | 0.24 |
| GSM437268 | 2.877346928 | low | FALSE | 0.02 | -0.09 | 0.02 |
| GSM437269 | 2.714539237 | low | FALSE | 0.64 | -1.1 | 0.64 |
| GSM437271 | 2.514821638 | low | FALSE | 0.16 | -0.59 | 0.16 |
| GSM437272 | 2.860706614 | low | TRUE | -0.96 | 1.11 | -0.96 |
| GSM437273 | 2.873352071 | low | FALSE | 0.44 | -0.64 | 0.44 |
| GSM437275 | 2.816224628 | low | TRUE | -0.49 | -0.71 | -0.49 |
| GSM437276 | 2.874014277 | low | FALSE | 1.31 | 0.23 | 1.31 |
| GSM437277 | 2.439310656 | low | FALSE | 0.27 | -1.12 | 0.27 |
| GSM437278 | 3.030850062 | high | FALSE | 0.1 | 0.87 | 0.1 |
| GSM437279 | 2.730752878 | low | FALSE | 0.72 | -0.3 | 0.72 |
| GSM437280 | 2.913862584 | high | FALSE | 0.77 | 0.2 | 0.77 |
| GSM437283 | 2.799317357 | low | TRUE | -1.73 | 0.87 | -1.73 |
| GSM437285 | 2.9268225 | high | TRUE | -1.19 | 0.91 | -1.19 |
| GSM437297 | 2.82930285 | low | FALSE | 0.67 | 0.67 | -1.4 |
| GSM437299 | 2.707065169 | low | TRUE | -0.17 | -0.17 | -0.61 |
| GSM437300 | 2.867886892 | low | FALSE | 0.43 | -0.59 | 0.43 |
| GSM437301 | 3.111630011 | high | FALSE | 1.32 | 0.15 | 1.32 |
| GSM437302 | 2.848729472 | low | TRUE | -0.04 | -1.01 | -0.04 |
| GSM437303 | 2.853687053 | low | TRUE | -0.01 | -0.26 | -0.01 |
| GSM437304 | 2.526722293 | low | TRUE | -0.85 | 0.87 | -0.85 |
| GSM437305 | 2.924456425 | high | FALSE | 1.27 | -0.4 | 1.27 |
| GSM437306 | 2.735628585 | low | TRUE | -0.29 | 1.43 | -0.29 |
| GSM437307 | 3.006494603 | high | FALSE | 0.74 | 0.74 | -0.57 |
| GSM437308 | 3.054769159 | high | FALSE | 0.39 | 0.7 | 0.39 |
| GSM437309 | 2.841325716 | low | FALSE | 1.03 | 1.03 | -0.54 |
| GSM437312 | 2.799799536 | low | TRUE | -0.25 | -0.88 | -0.25 |
| GSM437313 | 2.906746487 | high | TRUE | -0.38 | -0.38 | -0.09 |
| GSM437314 | 2.98657827 | high | FALSE | 1.26 | 1.26 | -0.53 |
| GSM437315 | 3.126628605 | high | FALSE | 1.36 | 1.36 | 0.57 |
| GSM437316 | 3.078263553 | high | FALSE | 0.62 | -1.61 | 0.62 |
| GSM437317 | 3.074717103 | high | FALSE | 0.93 | 0.12 | 0.93 |
| GSM437318 | 3.069140378 | high | FALSE | 1.17 | -0.67 | 1.17 |
| GSM437319 | 3.117737583 | high | FALSE | 0.36 | -0.36 | 0.36 |
| GSM437320 | 3.059484898 | high | TRUE | -1.16 | 1.08 | -1.16 |
| GSM437321 | 2.910336 | high | TRUE | -1.46 | 0.47 | -1.46 |
| GSM437322 | 2.989383474 | high | TRUE | -0.21 | 0.23 | -0.21 |
| GSM437324 | 2.878281438 | low | TRUE | -0.39 | 0.34 | -0.39 |
